# Status of human rights violations and trauma among North Korean defectors: a cross-sectional study

**DOI:** 10.12771/emj.2025.00367

**Published:** 2025-04-10

**Authors:** So Hee Lee, Won Woong Lee, Haewoo Lee, Jin Yong Jun, Jin-Won Noh

**Affiliations:** 1 Department of Psychiatry, National Medical Center, Seoul, Korea; 2 Department of Social Welfare, Catholic Kwandong University, Gangneung, Korea; 3 Department of Psychiatry, Kangwon National University Hospital, Chuncheon, Korea; 4 Department of Psychiatry, Ulsan University Hospital, Ulsan, Korea; 5 Division of Health Administration, Yonsei University, Wonju, Korea

**Keywords:** Democratic People’s Republic of Korea, Health policy, Human rights, Post-traumatic stress disorders, Social integration

## Abstract

**Purpose:**

This study aimed to identify the types of human rights violations and the associated psychological trauma experienced by North Korean defectors. It also examined the impact of trauma on the defectors’ interpersonal relationships, employment, and overall quality of life, while evaluating existing psychological support policies to suggest potential improvements.

**Methods:**

A multidisciplinary research team conducted an observational survey and in-depth interviews with approximately 300 North Korean defectors residing in South Korea from June to September 2017. Standardized measurement tools, including the Post-Traumatic Stress Disorder (PTSD) Checklist (PCL-5), Patient Health Questionnaire-9 (PHQ-9), Generalized Anxiety Disorder Scale-7 (GAD-7), and Short Form-8 Health Survey (SF-8), were employed. Statistical analyses consisted of frequency analysis, cross-tabulation, factor analysis, and logistic regression.

**Results:**

The findings revealed a high prevalence of human rights violations, such as public executions (82%), forced self-criticism (82.3%), and severe starvation or illness (62.7%). Additionally, there were elevated rates of PTSD (56%), severe depression (28.3%), anxiety (25%), and insomnia (23.3%). Defectors who resided in China before entering South Korea reported significantly worse mental health outcomes and a lower quality of life. Moreover, trauma was strongly and negatively correlated with social adjustment, interpersonal relationships, employment stability, and overall well-being.

**Conclusion:**

An urgent revision of existing policies is needed to incorporate specialized, trauma-informed care infrastructures within medical institutions. Furthermore, broad societal education to reduce stigma and enhance integration efforts is essential to effectively support the psychological well-being and social integration of North Korean defectors.

## Introduction

### Background

North Korean defectors are known to experience trauma-related disorders, such as post-traumatic stress disorder (PTSD), following their harrowing experiences [1-4]. Previous research has documented various forms of discrimination and human rights violations, notably among North Korean women, during the defection and resettlement processes in North Korea, third countries, and South Korea. It has also been suggested that the psychological and physical trauma encountered during defection and stays in third countries plays a major role in the social adjustment difficulties faced by North Korean defectors in South Korea [5,6].

### Objectives

This study aimed to identify the specific human rights violations experienced by North Korean defectors at different stages—while in North Korea, during the defection process, and after resettlement in South Korea—and to examine the associated psychological trauma. It also analyzes how trauma affects the defectors’ interpersonal relationships, workplace experiences, and daily lives, exploring its influence on social adaptation in South Korea. Furthermore, the study evaluates current psychological support policies for North Korean defectors, identifies deficiencies, and proposes targeted improvements.

## Methods

### Ethics statement

This study was conducted in accordance with ethical guidelines for research involving human participants. The study protocol was approved by the Institutional Review Board (IRB) of the National Medical Center, Seoul, Korea (IRB no., H-1704-077-002) and all procedures were performed in compliance with the Declaration of Helsinki.

The original Korean report is available at Supplement 1.

### Study design

This cross-sectional observational survey was designed to investigate the human rights violations and trauma experiences of North Korean defectors. The study was conducted in accordance with the STROBE (Strengthening the Reporting of Observational Studies in Epidemiology) statement available at: https://www.strobe-statement.org/.

### Setting

A multidisciplinary research team was established, consisting of psychiatrists experienced in treating North Korean defectors and conducting related research, experts in North Korean human rights, and public health PhD holders, was assembled. The team operated as a collaborative, multi-institutional unit to ensure a comprehensive and specialized approach to the study.

### Participants

Survey data were collected from approximately 300 North Korean defectors who had entered South Korea from June to September 2017. The gender distribution was structured at approximately a 7:3 ratio between women and men, reflecting the demographic profile of North Korean defectors in South Korea. Sampling was aimed to match the population distribution in terms of age, gender, and residential area (metropolitan cities and provinces). Recruitment was carried out using snowball sampling and through Hana Centers, organizations related to North Korean defectors, and counseling centers to ensure a representative sample.

### Variables

Outcome variables comprised the survey questions measuring human rights violations, trauma experiences, mental health, psychological state, and quality of life.

### Data sources/measurement

#### Establishment of survey tools for the study

A human rights violation assessment form was developed and combined with standardized tools to construct survey questions that measured diverse aspects including trauma experiences, mental health, psychological state, quality of life, and demographic factors (Table 1).

#### Development of an in-depth interview tool

Approximately 10 key questions were selected for in-depth interviews with North Korean defectors. The domains and corresponding items were systematically determined, and specific questions were finalized during researcher meetings and through expert consultations.

#### Implementation of the survey and in-depth interviews

A trauma expert with extensive experience in treating North Korean defectors administered the survey to approximately 300 North Korean defectors upon their entry to South Korea, and conducted in-depth interviews with around 20 participants. Sampling for the survey utilized snowball sampling and promotional efforts through Hana Centers, organizations supporting North Korean defectors, and counseling centers.

### Bias

Due to the nature of snowball sampling, the generalizability of this study’s findings is limited.

### Study size

Assuming a 2-tailed test with a significance level (α) of 0.05, the minimum required sample size for testing mean differences between 2 independent, normally distributed groups (e.g., gender differences) was estimated based on effect size. For a small effect size (0.2), approximately 1,833 participants would be needed; for a medium effect size (0.5), around 295 participants; and for a large effect size (0.8), about 117 participants would be required. Similarly, for independent groups with non-normal distributions, approximately 1,919, 309, and 122 participants would be needed for small, medium, and large effect sizes, respectively.

### Statistical methods

The collected survey responses were coded and analyzed using frequency analysis, cross-tabulation analysis, factor analysis, and logistic regression to address the research objectives. In-depth interview data were examined to identify “key domains” and “core ideas,” which were later reviewed and categorized by the research team until a consensus was reached.

All statistical analyses were conducted using Stata/MP 17.0 software (Stata Corp.).

## Results

### Participants

Out of the 300 respondents, 245 (81.67%) were women and 55 (18.33%) were men, with an average age of 52.87±15.90 years. Regarding their North Korean residential origins, the majority (181 individuals, 60.32%) were from Hamgyong Province. A large proportion (258 respondents, 85.66%), reported having held employment (including small-scale trading). The most common marital status in North Korea was “married” (85 individuals, 28.33%).

Regarding defection characteristics, 135 defectors (44.99%) initially left North Korea during the 2000s. The majority (157 individuals, 52.34%) obtained South Korean citizenship in the 2010s. After defection, 181 respondents (60.33%) resided in China, with an average stay of 6.21±4.65 years, while 115 individuals (38.34%) entered South Korea directly without transiting a third country. Among those who stayed in China, approximately 40% experienced arrest by Chinese authorities, 33% were repatriated to North Korea, and about 35% were detained in labor camps or detention facilities.

Regarding post-arrival experiences in South Korea, 147 individuals (49.00%) reported having employment experience, of which only 36 (24.49%) remained employed at the time of the survey. Among those previously employed in South Korea, the primary reasons for job loss were health issues (69 individuals, 60.53%), followed by stress and adaptation difficulties (17 individuals, 14.91%). Concerning marital status after arriving in South Korea—considered separately from North Korean marital status—188 respondents (62.67%) reported being unmarried. Among married respondents, 65 individuals (58.04%) were married to fellow North Korean defectors. Lastly, a majority (251 individuals, 83.67%) still reported having family members remaining in North Korea.

### Status of human rights violation trauma experiences

#### Human rights violation experiences

**Fig. 1.**
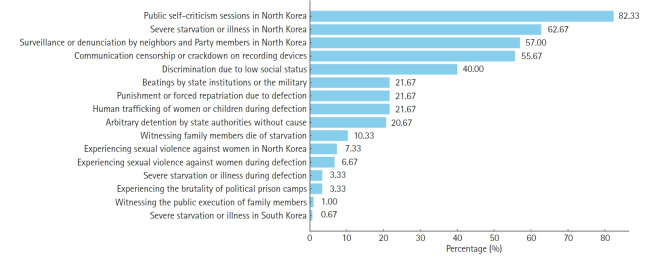
Prevalence of human rights violations experienced by North Korean defectors.

The most frequently reported human rights violations included being compelled to participate in public self-criticism sessions in North Korea (247 individuals, 82.33%), experiencing severe starvation or illness (188 individuals, 62.67%), and suffering from surveillance or denunciation by neighbors and Party members (171 individuals, 57.00%). Other severe violations included human trafficking of women or children during defection (65 individuals, 21.67%; specifically, 26.53% among women), sexual violence against women in North Korea (22 individuals, 9.0% of women), and witnessing public executions (246 individuals, 82.0%) (Fig. 1).

#### Trauma experiences

**Fig. 2.**
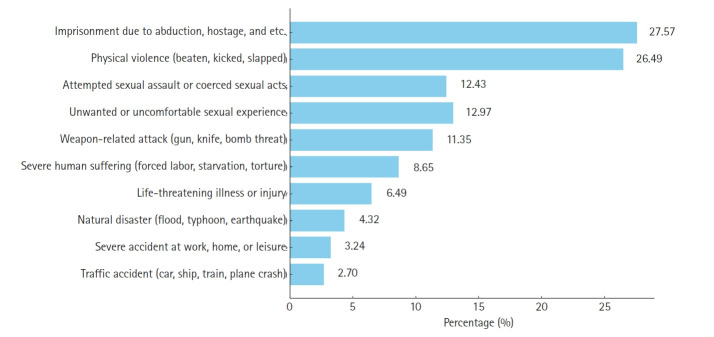
Prevalence of trauma experiences during defection.

The most common traumatic events in North Korea involved extreme human suffering, including forced labor, chronic starvation or food shortages, persistent homelessness, and torture (192 individuals, 64.00%). This was followed by physical violence such as assaults, beatings, slaps, or kicks (139 individuals, 46.33%) and by natural disasters like floods, typhoons, storms, or earthquakes (133 individuals, 44.33%). During defection, the most frequent traumatic experiences included imprisonment due to abduction, kidnapping, hostage situations, or being taken as a prisoner of war (51 individuals, 27.57%); physical violence including assaults, beatings, or slaps (49 individuals, 26.49%); and attempted sexual assault or coerced sexual acts through force or threat (23 individuals, 12.43%) (Fig. 2).

In South Korea, the primary traumatic experiences reported were traffic accidents involving cars, ships, trains, or airplane crashes (31 individuals, 10.33%), followed by life-threatening illness or injury (13 individuals, 4.33%).

### Mental health and quality of life status

Based on total scores from the PTSD Checklist (PCL-5), 168 individuals (56.00%) scored within a range indicating a need for clinical attention (scores of 33 or higher). PTSD levels were significantly higher among defectors under 60 years old compared to those aged 60 or older, and those who resided in China exhibited markedly higher PTSD symptoms than those who entered South Korea directly.

According to the Patient Health Questionnaire (PHQ-9), participants were classified as follows: normal (scores ≤4) for 62 individuals (20.67%), mild depression (scores between 5 and 9) for 57 individuals (19.00%), moderate depression (scores between 10 and 19) for 96 individuals (32.00%), and severe depression (scores between 20 and 27) for 85 individuals (28.33%).

Using the Korean version of the Suicidal Tendency Scale (K-MINI) to assess suicidal tendencies, 184 individuals (61.33%) were categorized as having a low suicide risk (scores of 5 or lower), 46 individuals (15.33%) showed moderate risk (scores between 6 and 9), and 70 individuals (23.33%) were identified as having a high suicide risk (scores between 10 and 19).

For anxiety, as assessed by the Generalized Anxiety Disorder Scale (GAD-7), 109 individuals (36.33%) scored within the normal range (scores of 4 or lower), 71 individuals (23.67%) had moderate anxiety (scores between 10 and 14), and 75 individuals (25.00%) exhibited severe anxiety (scores of 15 or higher).

Finally, based on the Insomnia Severity Index (ISI), 80 individuals (26.67%) were within the normal range (scores of 7 or lower), 71 individuals (23.67%) had mild insomnia (scores between 8 and 14), 79 individuals (26.33%) exhibited moderate insomnia (scores between 15 and 21), and 70 individuals (23.33%) were classified as having severe insomnia (scores between 22 and 28).

### The impact of human rights violation trauma on mental health, quality of life, and employment

#### Differences based on defection, entry process, and length of resettlement

Individuals who had lived in China prior to arriving in South Korea exhibited significantly higher levels of depression, suicidal tendencies, and PTSD compared to those who entered South Korea directly. Although the severity of insomnia did not differ significantly between the 2 groups, those who resided in China reported marginally but significantly lower quality of life and, notably, a higher employment rate. There were no significant differences in PTSD, depression, suicidal tendencies, or insomnia based on the length of resettlement in South Korea, suggesting that these mental health issues do not naturally resolve over time.

#### Differences based on experiences of human rights violations

Defectors who directly experienced or witnessed severe human rights violations—including severe starvation or illness, brutality in political prison camps, public executions, punishment for defection attempts, and sexual violence against women in North Korea—displayed significantly higher levels of PTSD symptoms. However, experiencing sexual violence during defection did not significantly alter PTSD levels between groups. In contrast, experiences of human trafficking of women or children during defection were associated with markedly higher PTSD symptoms

Quality of life was significantly lower among individuals who had personally experienced or witnessed public executions, punishments for defection attempts, sexual violence in North Korea, and human trafficking during the defection period.

Paradoxically, employment rates were higher among those who experienced or witnessed human trafficking during defection, suggesting that residence in China might act as a confounding factor influencing employment status.

#### Differences in mental health based on trauma experiences

An analysis comparing PTSD symptom levels revealed that individuals who directly experienced traumatic events—such as physical violence (attacks, being struck by hands, fists, or clubs), assaults involving weapons (guns, knives, or explosives), life-threatening illness or injury, or natural disasters (floods, typhoons, storms, or earthquakes) in North Korea or during defection—displayed significantly higher PTSD levels than those who did not have such experiences.

Additionally, experiences unique to North Korea, such as witnessing severe injury or death caused by oneself, being imprisoned through kidnapping or hostage-taking, or witnessing sudden violent deaths (murders or suicides), were also significantly associated with elevated PTSD symptoms.

Moreover, during defection, individuals who experienced severe human suffering (such as forced labor, prolonged starvation, chronic homelessness, or torture) demonstrated significantly higher PTSD symptoms compared to those without such exposures.

In South Korea, only experiences involving serious accidents in work, home, or recreational contexts resulted in significantly higher PTSD symptoms compared to those who did not encounter such events.

#### Differences in quality of life and employment according to trauma experiences

A comparative analysis between groups who directly experienced traumatic events and those who did not revealed significant differences in quality of life. In North Korea and during defection, individuals who directly experienced natural disasters, physical violence, weapon-related attacks, or life-threatening illness or injury scored significantly lower in quality of life than those without these experiences.

Furthermore, in North Korea, respondents who experienced sexual violence, unwanted or uncomfortable sexual encounters, or imprisonment, as well as those who witnessed sudden violent deaths, or serious injury or death caused by themselves, also reported significantly lower quality of life. During defection, severe human suffering further contributed to a reduced quality of life. In South Korea, only those who experienced severe accidents at work, home, or during leisure activities reported significantly lower quality-of-life levels.

Trauma experiences in North Korea did not significantly affect employment status. Interestingly, during defection, experiences such as natural disasters, physical violence, imprisonment, and life-threatening illness or injury were associated with higher employment rates, suggesting that residence in China was a confounding variable influencing employment outcomes. Similarly, in South Korea, individuals who experienced traffic accidents involving cars, ships, trains, or airplane crashes also had higher employment rates, indicating that employment status was more likely a causal factor than a consequence of these traumatic events.

### Analysis of in-depth interviews with North Korean defectors

#### Experiences of trauma from human rights violations

Respondents recounted various traumatic experiences, primarily involving severe starvation, illness, and death in North Korea; brutality in political prison camps; witnessing public executions; physical violence by state institutions or the military; discrimination due to low social status (*songbun*); enforced public self-criticism sessions; and pervasive surveillance through communication censorship or crackdowns on recording devices. Traumatic experiences encountered during the defection process were also documented.

#### Impact of human rights violation trauma on settlement in South Korea

Respondents described the negative impact of trauma on their interpersonal relationships. Many reported feelings of fear around others, distrust, heightened anger or aggression, and a tendency to avoid social interactions. In the workplace, they cited physical pain, reluctance to participate in company social events, and fear of going outside—factors that often hindered sustained employment. In daily life, defectors frequently experienced trauma-related symptoms, including nightmares, insomnia, anxiety, depression, headaches, fatigue, intrusive recollections of traumatic events, avoidance behaviors, hyperarousal, and persistent negative moods and cognition.

### Suggestions for supporting the psychological stability of North Korean defectors

Respondents proposed a range of measures to support their psychological stability. These included providing financial assistance, protection against discrimination and negative perceptions in South Korean workplaces and society, and ensuring stable access to medical care. They also stressed the importance of reducing the stigma associated with psychiatric treatment through targeted awareness campaigns. In addition, respondents emphasized the need for practical and personalized education programs that offer tangible support aligned with individual capabilities after graduating from Hanawon, the official Settlement Support Center for North Korean Defectors. Upon entering South Korea, defectors are required to stay at Hanawon for 3 months, during which they receive education on South Korean society, culture, and practical skills to facilitate integration.

## Discussion

### Key results

Among 300 North Korean defectors, 81.7% were women, and many experienced severe trauma, including starvation, public executions, physical violence, and trafficking. PTSD, severe depression, and anxiety were prevalent—especially among younger defectors and those who had resided in China. Human rights violations were significantly correlated with increased PTSD symptoms and diminished quality of life. Notably, employment rates were paradoxically higher among those with severe traumatic experiences during defection. Respondents also highlighted persistent psychological distress after arriving in South Korea.

### Interpretation

**Fig. 3.**
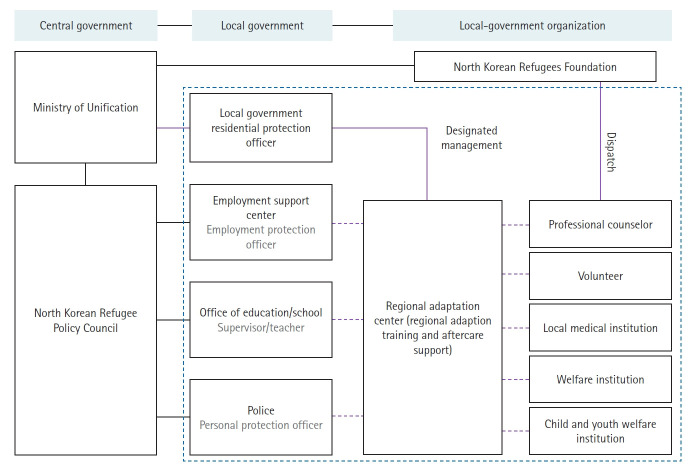
Overview of the current settlement support system for North Korean defectors (adapted from the Ministry of Unification, Korea).

At Hanawon, the primary health services offered to newly arrived North Korean defectors consist of psychiatric care, with mental health specialists conducting initial evaluations and treatments. In addition to this, the current support system provides financial assistance for mental health-related medical services and access to professional counselors specialized in mental health issues. This integrated approach is intended to facilitate defectors’ psychological adjustment and overall well-being (Fig. 3).

The findings clearly demonstrate that North Korean defectors experience high rates of human rights violations and trauma, which lead to serious mental health issues such as PTSD. These problems severely diminish their quality of life, adversely affecting interpersonal relationships, work experiences, and daily routines.

However, the current support system—especially through Hanawon and general mental health services—is insufficient. Given the severity and complexity of the trauma faced by these individuals, merely providing financial assistance or basic counseling is inadequate. The existing public healthcare services are not adequately equipped to address severe trauma-related disorders. Therefore, establishing a specialized trauma treatment infrastructure that can deliver comprehensive care and support is urgently needed.

### Recommendations

#### Establishing medical institution-based trauma treatment centers for North Korean defectors

Given the profound mental health and trauma-related challenges faced by North Korean defectors, it is recommended to establish specialized trauma treatment centers within national and public medical institutions nationwide. Initially, these centers could operate as pilot projects and later evolve into central hub facilities, ensuring comprehensive and systematic treatment and support services. This approach would leverage existing public healthcare infrastructure to facilitate early identification, treatment, and ongoing support, ultimately improving defectors’ mental health, quality of life, and integration into South Korean society.

#### Entrusting medical consultation services for North Korean defectors to relevant healthcare institutions

Instead of relying solely on private organizations, the Ministry of Unification should consider entrusting medical consultation services for North Korean defectors directly to healthcare institutions experienced in providing treatment and support to this population [7]. Furthermore, deploying mental health professionals to these institutions could establish a more structured support system, allowing for continuous and systematic management of healthcare resources. This coordinated, integrated approach overseen by the Ministry of Unification would enhance the efficiency and effectiveness of long-term psychological and medical care for defectors.

#### Customized support system tailored to entry pathways, gender, and life stages

A support system tailored to the diverse needs of North Korean defectors should account for differences in entry pathways, gender, and specific life stages [8]. Defectors who previously resided in China face unique challenges, including psychological distress from being separated from spouses and children, as well as severe post-traumatic stress from experiences such as arrest by Chinese authorities and forced repatriation to North Korea. Although some may have achieved economic adaptation during their stay in China, the emotional and psychological difficulties necessitate specialized, trauma-informed care.

Considering gender differences, female North Korean defectors experience significantly higher rates of sexual violence and human trafficking compared to other refugee groups. Therefore, specialized mental health support that addresses issues such as depression, low self-esteem, and trauma recovery is essential. A comprehensive psychological treatment and counseling system involving mental health professionals must be established to effectively manage severe trauma.

Additionally, support measures must consider life-cycle characteristics. Elderly defectors often face severe isolation, loneliness, and guilt related to family separation, whereas younger defectors frequently struggle with adapting to the South Korean education and social systems. Sustainable, mid- to long-term support programs tailored to address the unique challenges at each life stage are necessary. Examples include isolation and emotional distress among older defectors, and educational and social integration challenges among defector youth.

#### Legal foundation establishment

To establish a solid legal basis for addressing the trauma experienced by North Korean defectors, it is recommended to amend the existing Act on the Protection and Settlement Support for North Korean Defectors. The amendment should specifically mandate the establishment and operation of trauma treatment centers dedicated to North Korean defectors. This legal foundation would ensure structured, long-term psychological support and treatment, which is essential for their successful integration into South Korean society.

### Limitations

This study utilized snowball sampling rather than randomized sampling; as a result, it is difficult to generalize the findings to the entire population of defectors.

### Conclusion

To overcome prejudice and negative perceptions toward North Korean defectors, it is crucial to shift policy approaches from separation and minimal support to one of localization and integration. Efforts should focus on raising awareness within South Korean society to reduce stigma and discrimination. Specifically, the current support policies—predominantly managed by the Ministry of Unification—must be revised to establish specialized infrastructure capable of professionally addressing the lingering effects of trauma from human rights violations. Collaboration with relevant ministries, such as the Ministry of Health and Welfare and the Ministry of Gender Equality and Family, is necessary to develop integrated systems that offer specialized, trauma-informed care. This comprehensive, sustainable support is essential for facilitating the effective societal integration of North Korean defectors.

## Figures and Tables

**Table 1.** Survey tools

Evaluation items	Tools
Socio-demographic domain	Motivation for defection, year of defection, defection route, year of entry into South Korea, marital status, medical history, etc.
Human rights violations and PTSD	
Human rights violation questionnaire	Human rights violation questionnaire developed specifically for North Korean defectors
Traumatic event list	Life Events Checklist (LEC-5)
Post-traumatic symptoms	PTSD Checklist-5 (PCL-5)
Depression	Patient Health Questionnaire-9 (PHQ-9)
Anxiety	7-item Anxiety Scale (Generalized Anxiety Disorder Scale, GAD-7)
Suicide risk	Korean version of the Suicidal Tendency Scale (K-MINI)
Insomnia	Insomnia Severity Index (ISI)
Quality of life	Short Form-8 Health Survey (SF-8)
Alcohol and smoking habits	Alcohol Use Disorders Identification Test (AUDIT-C); smoking assessment questions

PTSD, post-traumatic stress disorder.

## Supplementary materials

Supplementary files are available from Harvard Dataverse: https://doi.org/10.7910/DVN/ZJ4QP3

Supplement 1. Original Korean report of this article.
