# Supplementary material for: Status of human rights violations and trauma among North Korean defectors: a cross-sectional study
Source: Ewha Med J. 2025 Apr 10;48(2):e28. doi: 10.12771/emj.2025.00367 (PMC12277490; doi:10.12771/emj.2025.00367)
Supplement: Supplementary file 1 — Supplement 1. Original Korean report of this article. [file emj-2025-00367-Supplementary-1.pdf]

발 간 등 록 번 호
11-1620000-000664-01

2017년 인권상황 실태조사
연구용역 보고서

북한이탈주민 인권피해 트라우마 실태조사

국가인권위원회
Nation Human Rights Commission of Korea

북한이탈주민
인권피해 트라우마 실태조사

2017년도 인권상황 실태조사 연구용역보고서를 제출합니다.

2017.10.27.

연구수행기관 **국립중앙의료원**

책임연구원 **이소희** (국립중앙의료원)

공동연구원 **이원웅** (가톨릭관동대학교)

이해우 (서울의료원)

전진용 (국립정신건강센터)

노진원 (을지대학교)

연구보조원 **한우리** (국립중앙의료원)

이 보고서는 연구용역수행기관의 결과물로서,
국가인권위원회의 입장과 다를 수 있습니다.

연 구 요 약

1. 연구의 필요성

○ 탈북자들은 외상 후 스트레스 장애(Posttraumatic Stress Disorder, 이하 PTSD)와 같이 트라우마를 경험한 후 나타나는 질환과 연관되어 있는 것으로 알려져 있다.

○ 선행연구에서 북한여성들이 북한, 제3국, 한국 등 탈북과 정착과정에서 경험하는 다양한 차별과 인권침해 경로들을 보고하고 있다.

○ 또한 탈북과정 및 제3국 체류 과정에서 발생한 심리 및 신체적 외상 요인들이 북한이탈주민의 남한사회 부적응을 초래하는 주요한 요인이라는 지적이 있었다.

2. 연구의 목적

○ 재북 시, 탈북기간, 국내 입국 후로 구분하여 북한이탈주민이 각 단계별 겪었던 인권침해 유형과 그에 따른 심리적 트라우마와의 연관성을 파악하고자 한다.

○ 트라우마가 북한이탈주민의 대인관계, 직장생활, 기타 일상생활에서 나타나는 영향을 분석하고, 트라우마가 북한이탈주민의 사회적응에 미치는 영향을 분석하고자 한다.

○ 북한이탈주민 심리지원 정책에 대한 문제점을 분석하고 개선방안을 제시하고자 한다.

3. 연구방법

○ **실태 조사를 위한 설문도구 확립**

인권침해 조사표를 개발하였고, 트라우마 경험, 정신건강, 심리, 삶의 질, 인구사회학적 요인 등 다양한 영역의 결과를 측정할 수 있는 표준화된 도구를 사용하여 설문 문항을 구성하였다.

○ **심층 면접 도구 확립**

북한이탈주민을 대상으로 심층면접을 시행할 때 사용할 질문 문항을 약 10개 내외로 선정하였다. 영역 및 항목을 구조적으로 결정하고 구체적 질문 내용을 선정하여 연구자 회의 및 전문가 자문을 통하여 검토 후 확정하였다.

○ **실태조사 및 심층면접 실시**

북한이탈주민 진료 경험이 풍부한 트라우마 전문가가 남한에 입국한 북한이탈주민 약300명을 대상으로 설문 자료를 수집하였고, 약 20명을 대상으로 심층면접을 시행하였다. 실태조사를 위한 북한이탈주민의 표집은 스노우볼 샘플링과 하나센터, 북한이탈주민 관련 단체 및 상담실 등 기관 홍보를 통하여 이루어졌다.

○ **자료 분석**

조사된 설문지는 코딩 후, 빈도 분석, 교차분석, 요인분석, 로지스틱 회귀분석 등을 이용하여 연구목적에 따른 결과를 도출하였다. 심층면접 자료는 연구자들에 의해 ‘영역’과 ‘핵심 아이디어’가 도출되었고, 회의에서 검토 후 합의점에 이르면 범주화하여 결과를 도출하였다.

○ **북한이탈주민 지원 정책과 심리지원제도 문제점 분석 및 대안 도출**

북한이탈주민 정착지원에 관한 법률, 사회보장법, 정신건강증진 및 정신질환자 복지서비스 지원에 관한 법률 등 관련법령의 정신건강 지원제도를 검토하여 하나원, 지역적응센터 등에서 실시되고 있는 북한이탈주민 정신건강 및 심리치료 프로그램에 대한 문제점을 도출하고 개선 방안을 제시하였다.

4. 선행 연구 고찰

○ **해외 난민과 이주민 연구**에 의하면 이주 전 트라우마 경험과 이주관련 상실, 이별, 트라우마가 복합적으로 작용하고 이주 후 빈곤 및 문화동화문제 등으로 정신건강 문제가 높은 것으로 알려져 있다.

- 여기에는 PTSD, 불안, 우울, 신체화장애, 물질남용 등이 있다.
- 치료법으로는 약물요법 외에도 인지행동요법과 노출요법이 효과적으로 나타났고, 트라우마에 중점을 둔 요법(trauma-focused therapy, TFT)과 다양한 모드의 개입(multimodal intervention)이 제안되고 있다.

○ **북한이탈주민은** 탈북전 및 탈북기간 중 노동착취, 인신매매, 성폭력, 강제송환, 교화소 수감 등 인권침해 및 극심한 트라우마를 경험하는 것으로 보고되고 있다.

- 그로인해 PTSD(30~50%), 우울, 불안 등 정신건강문제에 시달리고 있고, 신체질환 공존율이 높다. 이들에게는 심리적 지원만이 아닌 의료적 차원에서 통합적으로 정신건강에 대한 개입이 필요함을 알 수 있다.
- PTSD는 만성적 경과를 밟을 경우 다른 정신질환과의 공존이 높아지고, 사회적 기능 저하 및 삶의 질 저하와 연관되는 것으로 알려져 있다.

○ **해외 트라우마센터 사례 검토 연구**에 의하면, 주로 국가적 재난에 대응하여 출동하거나 트라우마 후유증 치료를 지원하기 위하여 트라우마 센터를 운영하고 있다.

- 일본의 경우, 재난응급정신의료지원팀(Disaster Psychiatry Assistant Team)을 구성하여 재난 시 법적 근거에 의하여 조직화된 정신의료팀이 정부 지원을 받아 출동하여 일정기간 기능을 수행하는 형태이다.
- 미국의 경우 적십자사를 중심으로 민간 자원봉사조직이 상설화 운영되고 있고, 재난정신지원단(Disaster Psychiatry Outreach)같은 민간 정신건강전문가 자원봉사조직 등이 활동하고 있다.

5. 연구 결과

○ 일반적인 특성

- 총 300명 중에 여성 245명(81.67%), 남성 55명(18.33%)으로 구성되었고 평균 연령은 52.87±15.90세이었다.

- **북한에서의 특성**

  주요 거주지는 함경도 거주자가 181명(60.32%)으로 가장 많았다. 북한에서 직업이 '있음'으로 응답한 경우가 258명(85.66%)이었다(장사 포함). 북한에서의 결혼 상태는 '기혼'이 85명(28.33%)으로 가장 많았다.

- **탈북 관련 특성**

  최초탈북년도는 2000년대 탈북자가 135명(44.99%)으로 가장 많았고, 한국 국적 취득년도는 2010년대 취득자가 157명(52.34%)으로 가장 많았다.

  탈북 후 중국 체류가 181명(60.33%)으로 가장 많았고, 평균 체류 기간은 6.21±4.65년으로 나타났으며 제3국을 거치지 않고 남한에 바로 입국한 경우가 115명(38.34%)이었다.

  남한 입국 전 중국에 체류한 탈북민 중 약 40%는 공안에 체포된 경험이 있었고, 약 33%는 북송을 경험하였으며, 약 35%는 교화소 및 구치소 수감을 경험한 것으로 나타났다.

- **남한 입국 후 특성**

  북한이탈주민의 남한 입국 후 취업 경험에 따라서는 '예'가 147명(49.00%)이었으며, 그 중 현재 취업 지속여부는 '예'가 36명(24.49%)이었다. 남한 입국 후 취업 경험이 있었던 북한이탈주민이 직장을 그만둔 이유는 응답 건수 중 건강상의 이유가 69명(60.53%), 스트레스와 부적응이 17명(14.91%) 순이었다.

  북한이탈주민의 남한 입국 후 혼인 관련 상태에 대해서는 북한에서 결혼 여부와 무관하게 남한에서만의 경험만을 위주로 조사하였다. '미혼'이 188명(62.67%)으로 가장 많았고, 기혼의 경우 배우자는 '탈북민'이 65명(58.04%)으로 가장 많았으며, 북한에 남아 있는 가족이 있는지 여부에 대해서는 "예"인 경우가 251명(83.67%)으로 더 많았다.

○ 인권침해 트라우마 경험 실태

- 인권침해 경험

  북한이탈주민이 경험한 다빈도 인권침해 사건은 다음과 같다. 북한에서 공개적인 자아비판을 직접 경험한 경우가 247명(82.33%)으로 가장 많았고, 북한에서 심한 굶주림 또는 질병을 직접 경험한 경우가 188명(62.67%), 북한에서 이웃과 당원의 감시와 고발을 직접 경험한 경우가 171명(57.00%) 등의 순으로 나타났다.

  그 밖에 심각한 인권침해 경험으로는 탈북기간 중 여성 또는 아동 인신매매를 직접 경험한 경우가 65명(21.67%, 여성 중 26.53%), 북한에서 여성에 대한 성폭력을 직접 경험한 경우가 22명(여성 중 9.0%), 공개처형 목격이 246명(82.0%)으로 분석되었다.

- 트라우마 경험

  북한에서 직접 겪은 사건은 강제적인 노동이나 지속적인 굶주림 또는 식량부족, 지속적인 노숙 상태, 고문과 같은 심각한 인간적 고난이 192명(64.00%)에 달해 가장 많았고, 그다음으로는 공격, 가격(손, 주먹, 몽둥이로 공격), 따귀를 맞음, 또는 발로 채이거나 두들겨 맞는 등의 신체폭력 경험이 139명(46.33%)으로 나타났으며, 홍수나 태풍, 폭풍, 지진과 같은 자연재난 133명(44.33%) 순으로 나타났다.

  탈북 기간에 직접 겪은 사건은 납치, 유괴, 인질 및 전쟁 포로 등으로 인해 감금된 경험이 51명(27.57%)에 달해 가장 많았으며, 그다음으로는 공격, 가격(손, 주먹, 몽둥이로 공격), 따귀를 맞음, 또는 발로 채이거나 두들겨 맞는 등의 신체폭력 경험이 49명(26.49%), 성폭행 시도 또는 완력이나 위협 하에 성적 행위 경험 23명(12.43%) 순으로 나타났다.

  남한에서 직접 겪은 사건으로는 자동차 사고, 선박 사고, 기차 사고 및 비행기 추락과 같은 교통사고 경험 31명(10.33%), 목숨이 좌우될 정도의 질병이나 부상 경험 13명(4.33%) 순이었다.

○ 정신 건강, 삶의 질 현황

- 외상 후 스트레스 장애

PTSD checklist (PCL-5) 총점을 기준으로 구분한 결과, 주의요망(33점 이상)이 168명(56.00%) 으로 나타났다. 연령에 따라서는 60대 미만이 이상에 비해 높은 PTSD 수준이 높았고, 중국에 거주 경험자가 남한 직행의 경우보다 유의하게 PTSD 수준이 높았다.

- 우울

Patient Health Questionnaire (PHQ-9) 총점을 기준으로 구분한 결과, 정상(4점 이하) 62명(20.67%), 경도 우울(5점 이상 ~ 9점 이하) 57명(19.00%), 중등도 우울(10점 이상 ~ 19점 이하) 96명(32.00%), 고도 우울(20점 이상 ~ 27점 이하) 85명(28.33%)으로 각각 나타났다.

- 자살 경향성

K-MINI 총점을 기준으로 구분한 결과, '낮은 자살 위험성(5점 이하)' 184명(61.33%), 중간 정도의 자살 위험성(6점 이상 ~ 9점 이하)' 46명(15.33%), '높은 자살 위험성(10점 이상 ~ 19점 이하)' 70명(23.33%) 으로 각각 나타났다.

- 불안

Generalized Anxiety Disorder Scale 7-item (GAD-7)총점을 기준으로 구분한 결과, 정상(4점 이하) 109명(36.33%), 경도(5점 이상 ~ 9점 이하), 중등도(10점 이상 ~ 14점 이하) 71명(23.67%), 고도(15점 이상) 75명(25.00%)으로 각각 나타났다.

- 불면

Insomnia Severity Index (ISI) 총점을 기준으로 구분한 결과, 정상(7점 이하) 80명(26.67%), 경도(8점 이상 ~ 14점 이하) 71명(23.67%), 중등도(15점 이상 ~ 21점 이하) 79명(26.33%), 고도(22점 이상 ~ 28점 이하)가 70명(23.33%) 으로 각각 나타났다.

○ 인권침해 트라우마가 정신건강, 삶의 질 및 취업에 미치는 영향

- **탈북 및 입국 과정과 정착기간에 따른 차이**

  중국 거주경험자가 남한 직행자에 비해 유의하게 더 높은 우울 수준, 더 높은 자살경향성 수준, 더 높은 PTSD 수준을 나타냈었고 불면증은 정도는 유의한 차이는 아니었다. 삶의 질은 중국 거주경험자가 한계적으로 유의하게 낮았고, 취업자 비율은 중국 거주경험자가 높았다. 정착기간에 따른 PTSD, 우울, 자살경향성, 불면증 차이는 없어서 시간이 흐른다고 해결되는 문제는 아님을 시사한다.

- **인권침해 경험에 따른 차이**

  인권침해 피해를 직접 경험하거나 가족이 경험하거나 직접 목격한 집단과 그렇지 않은 집단을 비교하였을 때 북한에서 '심한 굶주림 또는 질병을 경험', '정치범 수용소의 잔혹한 경험', '공개처형', '탈북으로 인한 처벌', '여성에 대한 성폭력' 경험을 했을 때 유의하게 높은 PTSD 증상 수준을 나타내었다. 다만, 탈북 기간 중 '여성에 대한 성폭력' 경험은 집단 간 PTSD 증상 수준의 유의한 차이는 아니었다. 하지만. 탈북 기간 중 '여성 또는 아동 인신 매매'은 집단 간 PTSD 증상 정도의 유의한 차이가 있었다.

  삶의 질에 있어서는 '공개처형', '탈북으로 인한 처벌', '북한에서의 성폭력', '탈북 중 인신매매' 경험을 직접 경험 혹은 목격한 경우 유의하게 낮은 것으로 나타났다.

  취업 여부는 '탈북 중 인신매매'를 경험하거나 목격한 경우 오히려 취업자가 더 많아 중국 거주가 교란변수로 작용했을 것으로 보인다.

- **트라우마 경험에 따른 정신건강 차이**

  트라우마 사건 별로 트라우마를 직접 경험한 경우와 그렇지 않은 경우 집단 간 차이를 분석하였다. 트라우마를 북한에서 경험한 경우와 탈북기간에 경험한 경우에 공통적으로, 공격, 가격(손, 주먹, 몽둥이로 공격), 따귀를 맞음, 또는 발로 채이거나 두들겨 맞는 등의 신체폭력', '총이나 칼에 맞거나, 칼, 총, 폭탄으로 위협 당하는 등과 같이 무기에 의한 공격', '목숨이 좌우될 정도의 질병이나 부상', '홍수나 태풍, 폭풍, 지진과 같은 자연재난'을 경험한 집단이 그렇지 않은 집단에 비해 각각 유의하게 더 높은 PTSD 증상 수준을 나타내었다.

추가적으로 북한에서는 '나 자신 때문에 발생했던 다른 사람의 심각한 부상, 상해 또는 사망을 목격', '납치, 유괴, 인질 및 전쟁 포로 등으로 인한 감금', '타인이 살인이나 자살 등의 급작스러운 변사를 당하는 것을 목격'을 경험한 집단에서도 그렇지 않은 집단에 비해 각각 유의하게 더 높은 PTSD 증상 수준을 나타내었다.

또한 탈북기간 동안에는, '강제적인 노동이나 지속적인 굶주림 또는 식량 부족, 지속적인 노숙 상태, 고문과 같은 심각한 인간적 고난'을 경험한 집단도 그렇지 않은 집단에 비해 각각 유의하게 더 높은 PTSD 증상 수준을 나타내었다.

남한에서 경험한 집단이 그렇지 않은 집단에 비해 각각 유의하게 더 높은 PTSD 증상 수준을 나타내었던 트라우마 경험은, '직장이나 집 또는 여가 활동 중 심각한 사고' 만으로 밝혀졌다.

- **트라우마 경험에 따른 삶의 질, 취업 차이**

트라우마 사건 별로 트라우마를 직접 경험한 경우와 그렇지 않은 경우 집단 간 차이를 분석하였다. 북한과 탈북 기간 공통적으로 '자연재난', '신체폭력', '무기로 공격당함', '목숨이 좌우될 정도의 질병이나 부상'을 직접 경험한 경우가 그렇지 않은 경우 보다 삶의 질이 유의하게 낮게 나타났다.

추가적으로 북한에서는 '성폭력', '기타 원하지 않거나 불편한 성적 경험 공격', '감금', '급작스러운 변사 목격', '나 때문에 발생했던 다른 사람의 심각한 부상, 상해 또는 사망 목격'을 직접 경험한 경우 삶의 질이 유의하게 낮았다.

한편, 탈북기간에는 추가적으로 '심각한 인간적 고난'을 경험한 경우 삶의 질 유의하게 낮았다. 남한에서는 직접 경험했을 때 유의하게 삶의 질이 낮은 것은 '직장이나 집 또는 여가 활동 중 심각한 사고'만으로 나타났다.

북한에서의 트라우마 경험은 취업 유무에 유의한 영향을 주지 못하였고 탈북기간의 '자연재난', '신체폭력', '감금', '목숨이 좌우될 정도의 질병이나 부상'을 경험한 경우 오히려 취업자 비율이 높았는데 중국 거주가 교란 변수로 작용한 것으로 해석된다. 남한에서는 '자동차 사고, 선박 사고, 기차 사고 및 비행기 추락과 같은 교통사고' 경험이 있는 경우 오히려 취업자 비율이 높았는데 이는 취업 경험이 결과라기보다 원인으로 해석된다.

○ 북한이탈주민 심층면접 내용 분석 결과

- **인권침해 트라우마 경험**

  주로 북한에서의 '심한 굶주림 또는 질병, 죽음', '정치범 수용소 잔혹성', '공개 처형', '국가기관/군대에서 매질', '토대가 나쁘다는 이유로 인한 차별', '공개적인 자아비판', '통신 검열 또는 록화기 단속' 관련 다양한 경험과 탈북 기간 동안의 경험이 기술되었다.

- **인권침해 트라우마 경험이 한국정착에 미치는 영향**

  대인관계 영역에서 '사람이 무섭다.', '사람을 의심한다', '욱해서 싸운다.', '사람을 피한다.'는 등의 부정적 영향을 호소하였고, 직장 생활 영역에서는 '온몸이 아프다', '회식 자리를 가기 싫다.', '바깥에 나가는게 두려워 일을 할 수 없다'는 등의 부정적 영향을 호소하였다. 일상생활에 악몽, 불면, 불안, 우울, 두통, 무기력 등을 포함한 트라우마 관련 재경험, 특정 상황 회피 반응, 과각성, 부정적 기분 인지 등 다양한 후유 증상을 호소하였다.

- **탈북민의 심리적 안정을 돕기 위한 제안**

  금전적 지원, 남한 회사나 사회에서 탈북민을 향한 차별과 부정적 시선으로부터 보호 받을 수 있는 방법, 병원 진료를 안정적으로 받을 수 있는 의료지원, 정신과 치료에 대한 편견이나 불이익이 없으면 좋겠고 홍보가 필요, 하나원 졸업 후 실제 사회생활을 하면서 받을 수 있는 개개인의 능력에 맞고 실질적 도움이 되는 교육 등 다양한 의견을 제안하였다.

6. 정책적 제언

○ 현행제도

- 하나원에서는 보건의료 서비스 내에서 정신건강의학과 전문의를 주축으로 하는 입소 탈북민의 정신건강 초기 평가와 상담 진료 서비스가 이루어지고 있다.
- 현행 북한이탈주민의 정착지원제도는 정신건강 관련하여서는 의료비 지원과 '정신건강 검사 등 전문적 서비스를 제공'할 수 있는 전문상담사 제도가 있다.

○ 문제점

- 스노우볼샘플링 특성 상 이번 조사 결과를 일반화하기 어렵다는 제한점은 있지만, 북한이탈주민은 인권침해 및 트라우마 경험율이 높고 그로인한 PTSD 등 정신건강 문제가 심각하여, 삶의 질을 저하시키며, 후유증으로 대인관계, 직장생활, 일상생활에 부정적 영향에 시달리고 있어 이에 대한 대책이 필요하다.
- 그러나, 현재 국내 의료환경을 고려하였을 때 단순한 의료비 지원만으로는 건강의료보험 및 의료급여 체계에서 심각한 복합 PTSD를 치료하기에 역부족이다. 즉, 환자가 의뢰되었을 때 그들을 제대로 치료할 수 있는 트라우마 치료 인프라 구축이 시급하다.
- 한편, 현행 전문상담사 제도는 애초에 정신건강에 대한 전문적 치료 역할을 할 수 있는 인력이 아니고, 의료기간 내 의료상담실은 민간에게 위탁하여 운영되고 있어 비효율적이다.

○ 제언

- **의료기관 기반 북한이탈주민 트라우마 치료 센터 구축**

  심각한 인권침해 트라우마 경험과 그로인한 정신적 신체적 어려움을 겪고 있는 북한이탈주민에게 적절한 치료 서비스를 제공하기 위해서는 전국 국공립 의료기관이 있으므로 의료기관 기반 북한이탈주민 전문 트라우마 치료센터를 구축하여 시범 사업을 시행한 후 거점 센터로 확대 운영하는 것을 검토할 수 있다.

  북한이탈주민을 우리 사회의 보현적인 사회안전망, 사회복지 빛 보건의료 시스템 속에 편입하되 그들을 위한 특화된 서비스를 추가로 지원하는 것이 그들의 사회 적응과 통합을 돕기위해 바람직할 것이다.

- **북한이탈주민 의료상담실을 해당 의료기관에 위탁**

  통일부는 탈북민 의료 상담실 운영을 민간에게만 위탁할 것이 아니라 탈북민 치료 지원을 하고 있는 의료기관에서 운영할 수 있도록 위탁하는 것이 더 합리적이고 효율적일 것이다.

  다른 대안으로 통일부차원에서 보건복지부에 정신건강 및 보건의료지원 사업을 위탁함으로써 보건복지부가 탈북민 진료사업을 하는 의료기관에 전문 인력을 배치하고 하나원에서 지역 사회로 연속적이고 체계적으로 보건의료자원을 활용하여 관리할 수도 있다.

- **맞춤형 지원체제: 유입경로별, 성별, 생애주기별**

  유입경로별 특성 고려: 중국체류를 거쳐서 유입되는 탈북민의 경우 어느 정도 자본주의사회의 제도와 남한사회에 대한 정보를 가지고 있다는 이점이 있는 반면, 중국에 두고 온 남편이나 자식들과의 관계로 인한 정신적 어려움, 중국체류 기간 중에 중국공안에 체포되어 북한으로 강제북송을 겪은 북한이탈주민의 경우 상당한 정도의 정신적 외상 후 스트레스 증상에 시달리고 있는 점 등을 고려할 때 중국체류를 거쳐서 입국하는 북한이탈주민에 대한 세밀한 검사와 지속적인 정신건강 관리가 필요하다.

  성별 특성 고려: 북한이탈주민 여성들은 인신매매, 성폭력 등에 노출되는 빈도수가 다른 지역 난민여성보다 심각한 상태인 점 등을 감안한다면 북한이탈주민 가운데 여성들에 대한 맞춤형 정신건강지원체제가 특히 필요하다. 즉, 일반적인 우울이나 자존감 회복을 위한 상시지원체제 이외에 심각한 인권피해, 특히 여성들에 대한 젠더폭력의 피해자들에 대한 정신과 전문의, 성폭력 상담 전문가, 여성인권 전문가를 포함한 체계적이고 전문적인 진단 및 치료, 상담시스템 구축이 시급하다.

  생애주기별 특성 고려: 북한이탈주민 고령층의 심각한 고립감과 외로움, 죄책감, 북한이탈 청소년들의 남한의 교육과 사회제도 부적응, 북한이탈주민 여성의 높은 가정폭력 경험 수준, 이혼과 중혼 등으로 인한 심한 스트레스, 북한에 두고 온 가족에 대한 죄책감 등을 고려한 대상별 북한이탈주민의 다양한 욕구체계에 대응하는 지속가능한 중장기 지원제도 구축이 필요하다.

- **정착지원법 개정(안)**

  북한이탈주민의 트라우마 후유증을 해결하기 위해서는 법적 기반을 마련하기 위해 현행 '북한이탈주민의 보호 및 정착지원에 관한 법률'내용 가운데 '북한이탈주민을 위한 트라우마 치료센터 설치 운영'을 위한 조항 삽입이 필요하다.

- **분리/지원정책에서 지역화/통합정책으로: 편견과 부정적 시선 극복을 위한 남한사회의 인식개선, 사회통합노력**

  북한이탈주민을 주변화하지 않기 위해서는 무엇보다 일반 국민들을 대상으로 하는 보편적인 차별철폐교육, 인권교육, 인식개선 노력이 광범위하게 설계되어 시행되어야 한다.

  이를 위해서는 현재 통일부(중앙정부)가 주도하는 북한이탈주민 지원제도를 수정하여 그들의 인권침해 트라우마 후유증을 전문적으로 치료해줄 수 있는 특화된 인프라를 구축하고, 보건복지부, 여성가족부, 행정안전부와의 협치체제 구축에 대한 치밀한 연구와 거시적인 정책조정 등 관련법의 개정이나 수정이 필요하다.

목차

〈제 1장〉 서 론

1. 연구의 필요성 ········ 1
2. 연구목적 ········ 3

〈제 2장〉 연구 내용 및 방법

1. 사전 설계 및 실태조사 계획 ········ 4
2. 선행 연구 고찰 ········ 5
3. 실태조사 설문 조사 및 심층 면접 프로토콜 확립 ········ 6
4. 실태조사 설문 자료 수집 ········ 10
5. 실태조사 심층면접 시행 ········ 16
6. 자료 취합 및 자료 분석 ········ 17
7. 북한이탈주민 지원 정책과 심리지원제도 문제점 분석 및 대안 도출 ······ 18
8. 최종결과보고서 작성 ········ 18

〈제 3장〉 선행 연구 고찰

1. 난민과 이주민의 트라우마 경험과 스트레스 ········ 19
2. 북한이탈주민의 인권침해 트라우마 경험 ········ 25
3. 북한이탈주민의 정신건강 및 사회적응 ········ 31
4. 해외 트라우마센터 사례 ········ 39

<제 4장> 연구결과

1. 연구참여자의 일반적 특성 ········ 43
2. 인권침해 트라우마 경험 실태 ········ 59
3. 정신건강, 삶의 질 및 건강행태 현황 ········ 71
4. 인권침해 트라우마가 정신건강, 삶의 질 및 취업에 미치는 영향 ········ 101
5. 심층 면접 내용 분석 결과 ········ 130
6. 소결 ········ 137

<제 5장> 정책적 제언

1. 현행제도 ········ 140
2. 문제점 ········ 148
3. 정책대안 ········ 154
4. 결론 : 분리/지원정책에서 지역화/통합정책으로 ········ 163

참 고 문 헌 ········ 165
색 인 ········ 178

<부록 1> 인권침해 조사표 ········ 171
<부록 2> 스트레스 사건 목록 (Life Events Checklist, LEC-5) ········ 172
<부록 3> 탈북민 트라우마 평가 설문지 ········ 173

표목차

<표 2-1> 실태조사 설문조사 도구 ···· 8

<표 2-2> 심층면접 도구 ···· 9

<표 3-1> 탈북여성의 공간별 인권침해 목록 ···· 30

<표 3-2> 외상 후 스트레스증후군과 사회적 기능 저하의 연관성과 관련한 연구 ···· 37

<표 4-1> 북한이탈주민의 성별 특성 ···· 43

<표 4-2> 북한이탈주민의 출생연도 특성: 요약 통계량 ···· 44

<표 4-3> 북한이탈주민의 출생연도 특성 ···· 44

<표 4-4> 북한이탈주민의 연령 특성 ···· 44

<표 4-5> 북한이탈주민의 북한생활 당시 거주지 특성 ···· 45

<표 4-6> 북한이탈주민의 북한생활 당시 직업 특성 ···· 46

<표 4-7> 북한이탈주민의 북한생활 당시 결혼상태 특성 ···· 46

<표 4-8> 북한이탈주민의 최초탈북년도 특성 ···· 47

<표 4-9> 북한이탈주민의 한국 국적 취득년도 특성 ···· 48

<표 4-10> 북한이탈주민의 탈북기간 중 제3거주국가 특성 ···· 48

<표 4-11> 북한이탈주민의 제3국 체류기간 특성 ···· 48

<표 4-12> 북한이탈주민의 탈북기간 중 중국공안체포경험 특성 ···· 49

<표 4-13> 북한이탈주민의 탈북기간 중 북송경험 특성 ···· 49

<표 4-14> 북한이탈주민의 탈북기간 중 교화소 및 구치소 수감경험 특성 ···· 50

<표 4-15> 북한이탈주민의 남한에서 취업한 경험 특성 ···· 51

<표 4-16> 북한이탈주민의 남한에서 취업한 유형 특성 ···· 51

<표 4-17> 북한이탈주민의 남한에서 취업 후 지속여부 특성 ································ 52
<표 4-18> 북한이탈주민의 남한에서 취업한 직장을 그만둔 이유 특성 ·········· 52
<표 4-19> 북한이탈주민의 남한에서 혼인 관련 상태 특성 ·································· 53
<표 4-20> 북한이탈주민의 남한에서 결혼한 배우자 출신 특성 ··························· 54
<표 4-21> 북한이탈주민의 북한잔류가족여부 특성 ··· 54
<표 4-22> 북한이탈주민의 북한잔류가족과의 관계 특성 ······································ 55
<표 4-23> 북한이탈주민의 북한잔류가족 수 특성 ·· 55
<표 4-24> 북한이탈주민의 남한에서 진단받은 질병 진단 당치 치료 여부 특성 ······· 56
<표 4-25> 북한이탈주민의 남한에서 진단받은 질병의 치료지속여부 특성 ····· 56
<표 4-26> 북한이탈주민의 남한에서 진단받은 질병의 치료를 지속하지 못한 이유 ···· 57
<표 4-27> 북한이탈주민의 남한에서 겪은 문화적응 스트레스 특성 ··················· 58
<표 4-28> 북한이탈주민이 경험한 인권침해 경험 특성: 요약 통계량 ············ 59
<표 4-29> 북한이탈주민이 경험한 인권침해 경험 특성 ·· 60
<표 4-30> 북한이탈주민의 북한 생활 당시 경험한 스트레스 사건: 요약 통계량 ······· 62
<표 4-31> 북한이탈주민의 북한 생활 당시 경험한 스트레스 사건 ···················· 63
<표 4-32> 북한이탈주민의 제3국 거주 탈북 기간 중 경험한 스트레스 사건: 요약 통계량 ··· 65
<표 4-33> 북한이탈주민의 제3국 거주한 탈북 기간 중 경험한 스트레스 사건 ···· 66
<표 4-34> 북한이탈주민의 남한에서 경험한 스트레스 사건: 요약 통계량 ······ 68
<표 4-35> 북한이탈주민의 남한에서 경험한 스트레스 사건 ································ 69
<표 4-36> 북한이탈주민의 PCL-5 총점 특성: 요약 통계량 ································ 71
<표 4-37> 북한이탈주민의 PCL-5 총점 특성 ·· 71
<표 4-38> 북한이탈주민의 PCL-5 군별 특성 ·· 73
<표 4-39> 북한이탈주민의 PCL-5 문항별 특성 ··· 76
<표 4-40> 북한이탈주민의 성별에 따른 PCL-5 총점 특성 ································ 79

<표 4-41> 북한이탈주민의 연령대에 따른 PCL-5 총점 특성 ······ 79
<표 4-42> 북한이탈주민의 북한 생활당시 거주지에 따른 PCL-5 총점 특성 ······ 80
<표 4-43> 남한입국과정 차이에 따른 북한이탈주민의 외상 후 스트레스장애 특성 ······ 80
<표 4-44> 북한이탈주민의 PHQ-9 총점 특성: 요약 통계량 ······ 81
<표 4-45> 북한이탈주민의 PHQ-9 총점 특성 ······ 81
<표 4-46> 북한이탈주민의 PHQ-9 군별 특성 ······ 82
<표 4-47> 북한이탈주민의 PHQ-9 문항별 특성 ······ 84
<표 4-48> 북한이탈주민의 K-MINI 총점 특성: 요약 통계량 ······ 85
<표 4-49> 북한이탈주민의 K-MINI 총점 특성 ······ 85
<표 4-50> 북한이탈주민의 K-MINI 군별 특성 ······ 86
<표 4-51> 북한이탈주민의 K-MINI 문항별 특성 ······ 87
<표 4-52> 북한이탈주민의 GAD-7 총점 특성: 요약 통계량 ······ 88
<표 4-53> 북한이탈주민의 GAD-7 총점 특성 ······ 88
<표 4-54> 북한이탈주민의 GAD-7 군별 특성 ······ 89
<표 4-55> 북한이탈주민의 GAD-7 문항별 특성 ······ 90
<표 4-56> 북한이탈주민의 ISI 총점 특성: 요약 통계량 ······ 91
<표 4-57> 북한이탈주민의 ISI 총점 특성 ······ 91
<표 4-58> 북한이탈주민의 ISI 군별 특성 ······ 92
<표 4-59> 북한이탈주민의 ISI 문항별 특성 ······ 93
<표 4-60> 북한이탈주민의 SF-8 총점 특성: 요약 통계량 ······ 94
<표 4-61> 북한이탈주민의 SF-8 총점 특성 ······ 94
<표 4-62> 북한이탈주민의 SF-8 문항별 특성 ······ 97
<표 4-63> 북한이탈주민의 흡연 특성: 요약 통계량 ······ 98
<표 4-64> 북한이탈주민의 흡연 특성 ······ 98

〈표 4-65〉 북한이탈주민의 음주 특성 ······ 99

〈표 4-66〉 북한이탈주민의 AUDIT-C 점수에 따른 임상군 분포 특성 ······ 100

〈표 4-67〉 남한입국과정 차이에 따른 북한이탈주민의 외상 후 스트레스장애 특성 ······ 101

〈표 4-68〉 남한입국과정 차이에 따른 북한이탈주민의 우울 특성 ······ 102

〈표 4-69〉 남한입국과정 차이에 따른 북한이탈주민의 자살경향성 특성 ······ 102

〈표 4-70〉 남한입국과정 차이에 따른 북한이탈주민의 불면증 심각도 특성 ······ 103

〈표 4-71〉 정착기간과 정신건강문제 간의 연관성 특성 ······ 103

〈표 4-72〉 남한입국과정 차이에 따른 북한이탈주민의 삶의 질 특성 ······ 104

〈표 4-73〉 남한입국과정 차이에 따른 북한이탈주민의 취업 특성 ······ 104

〈표 4-74〉 심한 굶주림 또는 질병경험 차이에 따른 북한이탈주민의 외상 후 스트레스 증상 ······ 106

〈표 4-75〉 정치범수용소의 잔혹성 경험 차이에 따른 북한이탈주민의 외상 후 스트레스 증상 ······ 106

〈표 4-76〉 공개처형 관련 경험 차이에 따른 북한이탈주민의 외상 후 스트레스 증상 ······ 107

〈표 4-77〉 탈북으로 인한 처벌 관련 경험 차이에 따른 북한이탈주민의 외상 후 스트레스 증상 ······ 108

〈표 4-78〉 북한에서 여성에 대한 성폭력관련 경험 차이에 따른 북한이탈주민의 외상 후 ······ 108

〈표 4-79〉 탈북과정 중 여성에 대한 성폭력관련 경험 차이에 따른 북한이탈주민의 외상 후 스트레스 증상 ······ 109

〈표 4-80〉 탈북과정 중 여성 또는 아동 인신매매 관련 경험 차이에 따른 북한이탈주민의 외상 후 스트레스 증상 ······ 109

〈표 4-81〉 인권침해경험 경험 차이에 따른 북한이탈주민의 삶의 질 특성 ··· 111

〈표 4-82〉 인권침해경험 경험 차이에 따른 북한이탈주민의 취업여부 특성 ······ 112

〈표 4-83〉 북한에서 겪은 트라우마 경험 차이에 따른 북한이탈주민의 외상 후 스트레스 증상 ······ 115

<표 4-84> 북한에서 겪은 트라우마 경험 차이에 따른 북한이탈주민의 삶의 질 특성 ······ 117
<표 4-85> 북한에서 겪은 트라우마 경험 차이에 따른 북한이탈주민의 취업여부 특성 ······ 118
<표 4-86> 탈북 기간 동안 겪은 트라우마 경험 차이에 따른 북한이탈주민의 외상 후 스트레스 증상 ······ 120
<표 4-87> 탈북 기간 동안 겪은 트라우마 경험 차이에 따른 북한이탈주민의 삶의 질 특성 ······ 122
<표 4-88> 탈북 기간 동안 겪은 트라우마 경험 차이에 따른 북한이탈주민의 취업여부 특성 ······ 124
<표 4-89> 남한에서 겪은 트라우마 경험 차이에 따른 북한이탈주민의 외상 후 스트레스 증상 ······ 126
<표 4-90> 남한에서 겪은 트라우마 경험 차이에 따른 북한이탈주민의 삶의 질 특성 ······ 127
<표 4-91> 남한에서 겪은 트라우마 경험 차이에 따른 북한이탈주민의 취업여부 특성 ······ 129
<표 4-92> 심층 면접 참여자의 일반적 특성 ······ 130
<표 5-1> 2017년 의료비지원 대비표 ······ 144
<표 5-2> 남북하나재단 공공의료체계 의료비 지원 ······ 145
<표 5-3> 북한이탈주민 의료지원 협약병원 현황 (17.8.3 기준): 정신건강의학과 진료 과목 운영 병원 ······ 146
<표 5-4> 하나원 교육생 진료현황 통계 ······ 149
<표 5-5> 전문상담사 채용기준 ······ 153
<표 5-6> 서울의료원 북한이탈주민 치과진료 사업 ······ 156

그림목차

〈그림 3-1〉 Percentage of those with income drop between years 2002 and 2010 by chronic health condition* (*including heart disease, diabetes, depression, anxiety, lung disease, and asthma) : among two rescue and recovery worker subgroups ········· 36

〈그림 5-1〉 통일부의 현행 정착지원제도 개요: 북한이탈주민 정착지원 체계도 ········· 142

제1장 서 론

1. 연구의 필요성

북한이탈주민은 1990년대 이후 본격적으로 입국하기 시작하여 그 증가세를 지속하여왔으며, 2016년 12월말 누적 입국자 기준 3만 명을 넘어섰다(통일부 홈페이지[1]). 구체적으로, 여성이 21,410명으로 남성이 902명인 것을 고려하면 전체 북한이탈주민 30,212명 중 여성이 차지하는 비율은 71%에 달한다.

북한이탈주민 진료 센터 개설 이후(2006. 01. 12.~ 2016. 02. 29.) 약 4,000명의 실인원을 진료한 국립중앙의료원의 진료 현황 조사에 따르면 정신건강의학과 진료 건수 및 다빈도 질환이 상위 5위안에 드는 것으로 나타났다(국립중앙의료원, 2017). 외래 연인원으로 살펴본 다빈도 진료과는 정형외과가 6,127(10.4%)로 가장 많았으며, 그다음으로는 정신건강의학과(10.3%), 산부인과(9.9%), 소화기내과(8.3%), 신경과(8.2%) 순이었다. 입원 연인원상에서는 정형외과가 5,281(13.4%)로 가장 많았으며, 그다음은 신경과(12.1%), 정신건강의학과(9.9%), 산부인과(9.0%), 한방내과(6.0%) 순이었다. 국립중앙의료원에 내원한 4,027명에서 가장 많은 진단명은 두통(7.6%)이었으며, 그다음으로는 등통증(4.1%), 위염(3.3%), 복통(3.3%), 우울(2.6%) 순이었다. 특히 주진단이 외상 후 스트레스(Posttraumatic Stress Disorder, PTSD)인 환자의 정신건강의학과 평균진료횟수가 17.0회로 없는 환자의 9.3회 보다 더 많았고 평균 의료비 지출도 PTSD가 있는 환자의 경우 54,512원으로 없는 환자의 44,819원 보다 더 높았다.

탈북자들은 외상 후 스트레스 장애(Posttraumatic Stress Disorder, PTSD)와 같이 트라우마를 경험한 후 나타나는 질환과 연관되어 있는 것으로 알려져 있다. 중국에 있는 탈북자들 170명의 정신건강 상태를 조사하였을 때 PTSD 유병율은 약 56%로 높은 편이었고, 불안(90%)과 우울(81%)도 더욱 높게 나타난 바 있다(Lee et al., 2001). 국내 입국 후 시행한 연구로는 하나원 입소자를 대상으로 한 연구에 의하면 1999년에 입국한 95명 연구 대상자의 27.3%가 PTSD를 겪는 것으로 나타났다

1) 북한이탈주민의 국내 입국 현황(2016)
http://www.unikorea.go.kr/content.do?cmsid=3099

(강성록, 2000). 하나원과 지역사회 탈북민을 대상으로 한 PTSD 유병율 연구 결과로는 302명 중 완전(full) PTSD가 26.15%이고, 부분(partial) PTSD는 50.66%로 보고되었다(김현아, 윤여상, 한선영, 2007). 1년이 미만의 지역 사회 탈북민 200명에서는 PTSD가 약 30%로 추정되는데, 여성이 남성보다 유의하게 높게 높은 것으로 알려져 있다(Jeon et al., 2005). 한편 정신건강의학과를 방문한 북한이탈주민 임상군을 대상으로 한 연구에 따르면 53%가 PTSD로 진단되어 매우 높게 나타났다(한나영 등, 2015).

이처럼 북한이탈주민의 심각한 정신건강 실태는 개인적 요인보다는 사회적으로 초래된 인권 침해의 결과로 추정된다. 따라서 인권침해 유형과 북한이탈주민의 트라우마 실태의 과학적 연관성을 제시할 필요성이 제기되고 있다. 국가인권위원회 탈북여성 인권실태에 관한 용역보고서에서 박순성 등(2009)은 북한여성들이 북한, 제3국, 한국 등 탈북과 정착과정에서 경험하는 다양한 차별과 인권침해 경로들을 보고하고 있다. 또한 탈북과정 및 제3국 체류 과정에서 발생한 심리 및 신체적 외상 요인들이 북한이탈주민의 남한사회 부적응을 초래하는 주요한 요인이라는 선행연구의 지적이 있다(조영아, 전우택, 2005).

따라서, 실태조사를 통하여 북한이탈주민의 인권침해 유형에 따른 트라우마를 객관적으로 파악하고 북한이탈주민의 정신건강을 지원할 수 있는 다양한 정책적 방안을 제시할 필요성이 제기되었다. 선행연구를 통해서 볼 때 현재 정부 차원에서 실행되고 있는 북한이탈주민 정신건강 지원 시스템의 효율성을 분석하고 심리적 외상요인의 대상별 및 범주별 맞춤형 프로그램을 적극적으로 도입할 필요성도 제기되었다.

2. 연구목적

가. 북한이탈주민 인권침해 유형에 따른 트라우마 현황 파악

재북 시, 탈북기간, 국내 입국 후로 구분하여 각 단계별 겪었던 인권침해 유형과 그에 따른 심리적 트라우마 관련 질환과의 연관성을 파악하고자 하였다. 인권침해 유형을 분류하기 위하여 인권피해사례 기록 및 분석 프로그램인 HURIDOCS의 WinEvsys 및 Martus와 국내 북한인권정보센터의 <NKDB 통합인권 DB> 북한인권 침해분류항목을 준용하여 분석하였다.

나. 트라우마가 북한이탈주민의 한국사회 적응과정에 미치는 영향 파악

트라우마가 북한이탈주민의 정신건강, 대인관계, 직장생활, 기타 일상생활에서 나타나는 영향을 분석하고, 트라우마가 북한이탈주민의 사회적응에 미치는 영향을 분석하고자 하였다.

다. 북한이탈주민 심리지원 프로그램의 문제점 분석 및 개선방안 제시

북한이탈주민정착지원에 관한법률, 사회보장법, 정신건강증진 및 정신질환자 복지서비스 지원에 관한 법률 등 관련법령의 정신건강 지원제도를 검토하고, 하나원, 지역적응센터 등에서 실시되고 있는 북한이탈주민 정신건강 및 심리치료 프로그램에 대한 문제점을 도출하였다. 이를 바탕으로 북한이탈주민 심리지원 정책에 대한 개선방안을 제시하고자 하였다.

제2장 연구 내용 및 방법

1. 사전 설계 및 실태조사 계획

가. 연구팀 구성

북한이탈주민 진료 경험 및 북한이탈주민 관련 연구 경험이 풍부한 정신건강의학과 전문의, 북한인권 전문가, 보건학 박사로 구성된 다기관 연구팀을 구성하였다.

나. 자문단 구성

하나원 및 하나센터 실무 전문가, 인권관련 전문가, 북한학 박사, 트라우마관련 전문 학회와 협업 체계 구축하였다.

다. 발주처(국가인권위원회)와 유기적 의사소통 관계 유지

발주처		연구팀		협업・자문단
국가인권위원회	→<br>←	북한이탈주민 인권 침해 및 트라우마실태 연구팀	→<br>←	한국트라우마스트레스학회(KSTSS)<br>외상 후 스트레스 장애 연구회

2. 선행 연구 고찰

기존 북한이탈주민 관련 자료(인권 피해, 트라우마 경험 관련 현황 및 정신심리사회적 영향) 및 해외 이주민 및 난민 관련 인권 피해 트라우마 자료를 수집하여 검토하였다. 즉, 기존 국내외 이주민 및 난민 조사 연구 리뷰, 기존 국내외 북한이탈주민 전수 실태 조사 및 사회 조사 리뷰, 북한이탈주민 트라우마 관련 연구 리뷰를 시행하였다.

문헌고찰은 국내외 인문・사회・과학 논문, 서적, 펍메드(Pubmed) 와 ISI Web of Science, KISS 등의 검색엔진을 이용, 적절한 키워드를 사용하여 문헌을 검색하였다. 초록만을 가지고 2인 이상의 연구원이 중복 검증을 통해서 연구 목표에 적합한 문헌인지 확인하였다. 간추려진 문헌들을 바탕으로 체계적 문헌 검토를 실시하였고, 연구 목적에 직・간접적으로 관련된 문헌자료를 추가로 검색하여 이들에 대한 문헌 연구를 진행하였다.

3. 실태조사 설문 조사 및 심층 면접 프로토콜 확립

가. 설문조사 도구 확립

1) 설문문항 선정

선행 연구 검토 결과, 북한이탈주민 면담 경험 및 현장에서 사용되는 트라우마 심리검사 도구 검토 결과를 바탕으로 재북 시, 탈북기간, 국내 입국 후로 구분하여 설문 도구를 개발하였다. 북한이탈주민 인권 침해 종류와 심각성을 반영할 수 있는 설문 문항과 트라우마 경험, 정신・심리적 상태와 사회정착에 필요한 기능 및 삶의 질 정도를 측정할 수 있는 표준화된 척도를 사용하였다.

2) 문항 검토

연구자 회의 및 전문가 자문를 통하여 선정된 문항을 검토하였다.

3) 문항 확정

북한이탈주민 2명을 대상으로 시행한 후 설문 문항을 최종적으로 확정하였다.

나. 인권침해 조사표

제22차 인권이사회 결의(2013.3.21)를 통해 설립된 북한인권 조사위원회(UN Commission of Inquiry on human rights in the Democratic People´s Republic of Korea, COI)는 1년간의 활동을 거쳐 2014년 2월 17일 서면보고서(written report)를 제출하고, 제25차 인권이사회(2014.3.17)에서 보고서를 최종 발표하였다. 유엔<2014 북한COI보고서>는 북한인권침해의 중요한 내용으로서 다음 9가지 항목을 적시하고 있다.

그 밖에도 성별에 기초한(gender-based) 인권침해, 특히 여성에 대한 폭력문제에 대해서 별도의 관심을 두었고, 위의 내용에 기초하여 인권침해 설문항목을 간추려 정리하였다(부록 1. 참조).

1. 식량권 침해
2. 정치범수용소 관련 모든 인권 침해 사항
3. 고문 및 비인간적 대우
4. 자의적 체포 및 구금
5. 각종 차별, 특히 기본적 인권과 자유에 대한 조직적인 박탈 및 침해 속에 이루어진 차별
6. 표현의 자유 침해
7. 생명권 침해
8. 이동의 자유 침해
9 .외국인 납치를 포함한 강제실종

다. 트라우마 경험 조사표

심리적 트라우마란, 일상적 스트레스 사건을 넘어서서, 생명이나 신체적 보전을 위협받거나 끔찍한 장면을 목격함으로써 심각한 정신적 충격을 받아 압도될만한 경험을 의미한다. 이 연구에서는 심리적 트라우마를 측정하기 위해서 일반적으로 많이 사용하는 도구인 Life Events Checklist (LEC-5)를 사용하였다(부록 2. 참조).

라. 인권침해・트라우마의 정신사회심리적 영향을 측정하는 설문 도구

표준화된 도구를 사용하여 인권침해나 트라우마 경험의 정신, 심리, 삶의 질 등 다양한 영역의 결과를 측정함으로써 신뢰도와 타당도를 확보하고자 하였다(부록 3 참조). 결과 예후에 영향을 줄 수 있는 인구사회학적 요인 등에 대한 설문 문항도 포함하여 설문지를 구성하였다. 전반적 설문 도구는 아래 표와 같다.

〈표 2-1〉 실태조사 설문조사 도구

평가항목		측정도구
인구사회학적 영역		탈북동기, 탈북연도, 탈북경로, 입국연도, 결혼상태, 질환력 등
인권침해 및 외상 후 스트레스 장애	인권침해조사표	북한이탈주민용 인권침해 조사표 개발
	트라우마 사건 목록	생활사건 조사표(Life Events Checklist, LEC-5)
	트라우마 후 증상	PTSD 체크리스트(PTSD check List-5, PCL-5)
우울		건강질문지-9(Patient Health Questionnaire-9, PHQ-9)
불안		불안척도-7(7 items anxiety scale, GAD-7)
자살위험		자살경향성 척도(K-MINI)
불면		불면증 심각도 지수(Insomnia Severity Index, ISI)
삶의 질		건강관련 삶의 질 평가(Short Form-8 Health survey, SF-8)
음주 및 흡연		The Alcohol Use Disorders Identification Test (AUDIT-C), 흡연 평가 문항

마. 심층면접 도구

인권침해트라우마를 경험한 북한이탈주민을 대상으로 심층면접을 시행할 때 사용할 질문 문항을 약 10개 내외로 선정하였다. 영역 및 항목을 구조적으로 결정하고 구체적 질문 내용을 선정하여 연구자 회의 및 전문가 자문을 통하여 검토 후 확정하였다.

〈표 2-2〉 심층면접 도구

대분류	소분류	내용 (심층면담을 통해 얻어짐)
인권침해 유형과 그에 따른 트라우마	북한에서	생명권, 개인의 존엄성 및 자유권(고문 및 폭행, 성적 학대 등), 식량권, 건강권, 교육권, 거주 및 주거권, 결혼과 가정에 대한 권리, 재생산권, 신념 및 표현의 자유, 재산권, 집회 및 결사권, 노동권, 참정권, 피의자와구금자의 권리, 기타(숙청, 개인프라이버시 침해)
	중국 등 제3국	
	한국 입국 후	
트라우마가 한국사회 정착에 미치는 영향	대인관계	
	직장생활	
	일상생활	
북한이탈주민 심리지원 정책 개선 방안	법제도 차원	
	정착지원 프로그램	
	심리지원 프로그램	

4. 실태조사 설문 자료 수집

가. 설문 자료 수집

설문 자료의 수집은 북한이탈주민 진료 경험이 풍부한 트라우마 전문가가 직접 시행하였다. 인권침해조사표 및 트라우마 목록 설문은 조사자에 따라 자료의 양과 질에 차이가 날 수 있는 영역이다. 또한 트라우마 전문가가 안정된 분위기에서 설문을 진행하는 것은 특히 과거 인권침해관련 경험과 트라우마 경험을 진술하면서 회상하는 과정에서 이차피해를 줄이는데 필요한 것으로 보였으므로, 전문가가 설문 자료 수집을 실시하였다.

나. 설문 자료 수집 대상

설문 자료의 수집은 남한에 입국한 북한이탈주민 약300명을 대상으로 실시하였다. 남녀 비율은 우리나라 북한이탈주민 입국 현황을 반영하여 7:3에 가깝게 구성하였다.

다. 표집방법

북한이탈주민 추정 결과를 바탕으로 연령, 성별, 거주지역(광역시와 도 단위)을 기준으로 모집단 비율에 맞추어 목표 표본을 수집하고 스노우볼 샘플링과 하나센터, 북한이탈주민 관련 단체 및 상담실 등 기관 홍보를 통하여 북한이탈주민을 표집하였다.

라. 표본크기 산출에 고려되어야 할 사항

검정력과 표본크기를 구하는 데는 여러 고려할 사항들이 많지만 그 중 중요하게 고려되어야 할 사항들은 제1종 오류와 제2종 오류, 효과크기(effect size) 등이다. 통계 방법마다 다른 공식을 가지고 표본크기를 계산해야 하므로 각자의 연구에서 어떤 변수를 주요 결과 변수(primary endpoint)로 하여 어떤 통계 방법을 사용하여 분석할 것인지 연구 계획단계에서 잘 계획되어야 한다.

가설검정은 표본에 기초하여 의사 결정이 이루어지기 때문에 오류가 발생할 가능성이 항상 존재한다. 이러한 오류에는 제1종 오류와 제2종 오류가 존재한다. 제1종 오류는 효과가 없는데도 있다고 할 확률이고 제2종 오류는 효과가 있는데도 없다고 할 확률이다. 그러므로 검정력은 효과가 있을 때 있다고 할 확률이며, 대개 제1종 오류는 0.05, 검정력은 0.8의 고정된 값을 사용한다. 보건통계에서는 0.95값을 권장하고 있다.

마. 효과크기 (effect size)

효과크기가 클수록 검정력은 커지지만 표본크기는 작아진다. 반대로 효과크기가 작다면 그 작은 차이를 유의하게 발견하기 위해서 표본크기는 커져야 한다. 효과크기는 선행논문이나 예비연구(pilot study)에서 주어진 평균, 표준편차, 비율 등을 통해 추정할 수 있다.

바. 개략적인 추정치

다음은 G*Power 3.1.9.2를 이용하여 표본수를 추정한 결과임. 국내 탈북 이주민 중 여성의 비율이 약 70%로 추산된 바 있기에 남녀 집단 표본의 분포 비율(Allocation ratio)을 3:7로 설정하였다. 모든 추정에서 검정력은 0.95로 설정하였고, 효과크기는 cohen이 제시한 기준값을 적용하였다. 이 지침은 유용한 시작점으로 활용되고 있다.

사. difference between two independent means (t-tests)

양측검정, 유의수준 0.05를 가정하여 효과크기별로 상호독립이며 정규분포하는 두 집단의 평균차(성별 차이)를 검정하기 위한 최소 필요 표본수를 추정한 결과, 작은 효과크기(0.2)의 경우 약 1,833명, 중간의 효과크기(0.5)의 경우 약 295명, 큰 효과크기(0.8)의 경우 약 117명으로 추정되었다.

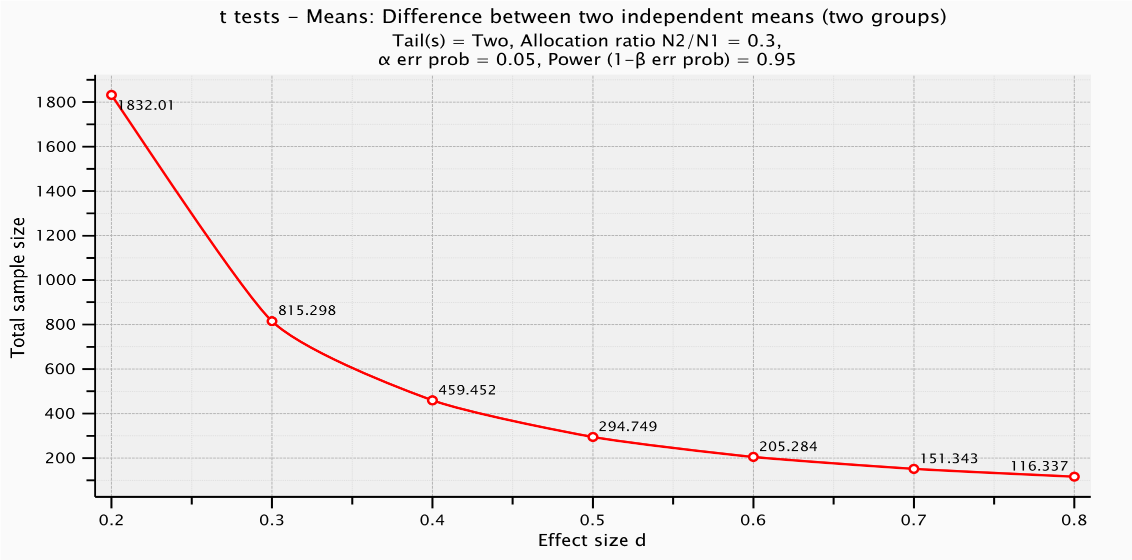

effect size	estimated sample size	effect size	estimated sample size
0.20 (small)	1832.01	0.51	283.379
0.21	1661.87	0.52	272.659
0.22	1514.39	0.53	262.540
0.23	1385.73	0.54	252.978
0.24	1272.82	0.55	243.933
0.25	1173.18	0.56	235.368
0.26	1084.82	0.57	227.250
0.27	1006.09	0.58	219.548
0.28	935.644	0.59	212.235
0.29	872.360	0.60	205.284
0.30	815.298	0.61	198.672
0.31	763.669	0.62	192.378
0.32	716.805	0.63	186.381
0.33	674.136	0.64	180.663
0.34	635.176	0.65	175.207
0.35	599.507	0.66	169.997
0.36	566.770	0.67	165.019
0.37	536.651	0.68	160.259
0.38	508.878	0.69	155.704
0.39	483.215	0.70	151.343
0.40	459.452	0.71	147.165
0.41	437.406	0.72	143.160
0.42	416.917	0.73	139.319
0.43	397.840	0.74	135.632
0.44	380.049	0.75	132.092
0.45	363.431	0.76	128.691
0.46	347.885	0.77	125.421
0.47	333.321	0.78	122.276
0.48	319.657	0.79	119.250
0.49	306.822	0.80 (large)	116.337
0.50 (medium)	294.749		

아. Wilcoxon-Mann-Whitney test of a difference between two independent means (U-test)

양측검정, 유의수준 0.05를 가정하여 효과크기별로 상호독립이지만 정규분포하지 않는 두 집단의 평균차(성별 차이)를 검정하기 위한 최소 필요 표본수를 추정한 결과, 작은 효과크기(0.2)의 경우 약 1,919명, 중간의 효과크기(0.5)의 경우 약 309명, 큰 효과크기(0.8)의 경우 약 122명으로 추정되었다.

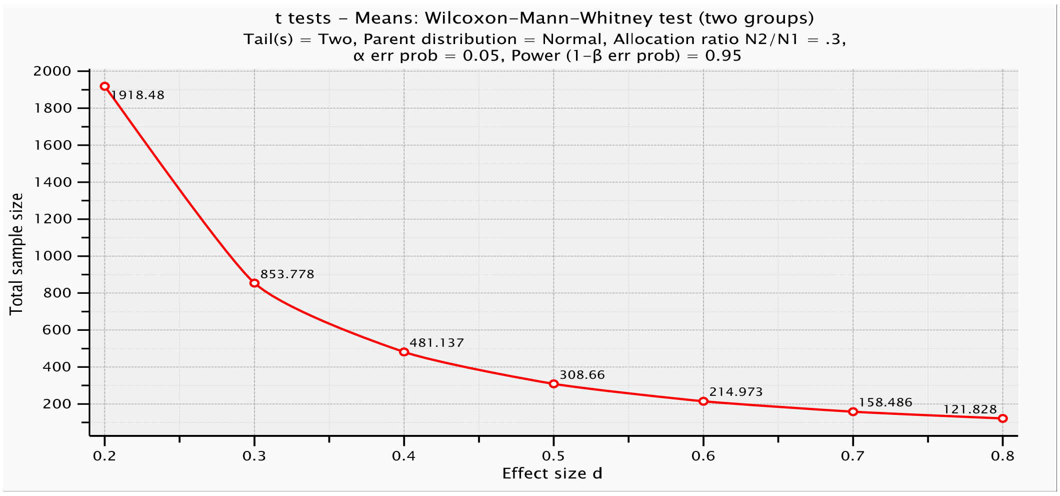

effect size	estimated sample size	effect size	estimated sample size
0.20 (small)	1918.480	0.51	296.754
0.21	1740.300	0.52	285.528
0.22	1585.870	0.53	274.931
0.23	1451.140	0.54	264.918
0.24	1332.890	0.55	255.446
0.25	1228.550	0.56	246.477
0.26	1136.020	0.57	237.976
0.27	1053.570	0.58	229.910
0.28	979.804	0.59	222.252
0.29	913.533	0.60	214.973
0.30	853.778	0.61	208.049
0.31	799.713	0.62	201.458
0.32	750.636	0.63	195.178
0.33	705.953	0.64	189.190
0.34	665.154	0.65	183.477
0.35	627.803	0.66	178.021
0.36	593.520	0.67	172.808
0.37	561.980	0.68	167.823
0.38	532.896	0.69	163.053
0.39	506.021	0.70	158.486
0.40	481.137	0.71	154.111
0.41	458.051	0.72	149.917
0.42	436.594	0.73	145.894
0.43	416.617	0.74	142.034
0.44	397.986	0.75	138.326
0.45	380.584	0.76	134.764
0.46	364.304	0.77	131.341
0.47	349.053	0.78	128.047
0.48	334.744	0.79	124.879
0.49	321.303	0.80 (large)	121.828
0.50 (medium)	308.660		

5. 실태조사 심층면접 시행

가. 연구윤리위원회의 승인

연구 대상자의 윤리적 측면을 준수하기 위하여 전반적인 연구 프로토콜은 연구윤리위원회의 승인을 받은 후 시행하였다. 실태조사 심층면접 실시 이전에 대상자가 연구에 대해 충분한 설명을 듣고 참여 의지를 자발적으로 결정하도록 하였다. 면접 내용은 녹취를 기본으로 하되 거부하는 자는 요약본을 기록하였으며, 면접 장소는 국립중앙의료원, 서울의료원, 하나센터 내소를 원칙으로 하였다.

나. 실태조사 심층면접의 대상

실태조사 면접은 다양한 목소리를 듣는 것이 목적이며, 면접을 기록하여 내용을 분석하는 것이 연구 진행 중에 이루어져 주제와 내용이 포화되어 더 이상 의미있는 새로운 내용이 나오지 않는 시점에 이르면 질적 분석 연구 자료의 추가를 중단하게 되므로 연구 시작 시점에 표본 크기를 결정할 수 없다(통상 최대치를 30명으로 설정한다.).

다. 실태조사 심층면접의 수집 방법

심층면접은 약 30~60분의 1회 면접으로 실시되었다. 객관식 답변은 없고 면접의 틀만 정해져 있는 반구조화된 면접으로 진행하였다. 경험의 본질적인 구조의 타당성을 연구 대상자로부터 도출하여 주제를 확인하였다. 훈련받은 정신건강의학과 전공의, 임상심리사들에 의하여 면접을 실시하였고, 연구 대상자가 심리적으로 안정된 분위기에서 면접에 임할 수 있도록 하였다. 면접을 담당한 전공의, 임상심리사들이 면접 내용의 축어록 작성 및 코딩에 참여하였다.

라. 실태조사 심층면접의 중지 및 탈락 기준

연구 대상자는 면접 도중 응답을 거부하거나 중단할 권리를 가진다.

6. 자료 취합 및 자료 분석

가. 설문 조사의 양적 통계 분석 방법

조사된 설문지는 미리 작성된 코딩가이드 및 코딩 입력폼에 의해 기록되었으며, 빈도분석, 교차분석, 요인분석, 로지스틱 회귀분석 등을 이용하여 연구목적에 따른 결과를 도출하였다. 구체적으로 설문조사 분석은 변수들의 서술적 분석을 통해 빈도 분포와 이 변수들에 사용된 통계적 기술을 위한 기본적인 가정을 충족하는지를 검증하였다. 그 후 피어슨 상관관계분석이나 카이스퀘어 분석을 이용하여 변수들 사이의 상관관계를 검토하였다. 본 연구에서는 묘사적 연구, 다수준 분석을 이용한 다중 회귀분석, 그리고 로지스틱 분석이 사용되었으며, 통합의료의 국가 간 형태를 나타내기 위해서 요인분석을 사용하였다.

결측값(missing data)의 경우는 SAS 10.1에서 제공되는 다중 대치법(multiple imputation)을 사용하였다. 결과의 안정성과 타당성(validity)를 파악하기 위해 extreme bound analysis (Deravi, Hegjiand Moberly 1990; Leamer 1978; Leamer 1982; Leamer 1983)를 STATA do file을 사용해서 실행하였고, jackknife test를 STATA 12를 이용하여 수동으로 실행하였다.

나. 심층 면접 내용 분석 방법

이 연구는 통계적 가설을 설정하지 않고, 내용 분석과 의미 추출을 목적으로 한다. 심층면접 자료는 인터뷰를 시행한 스크립트 요약본으로 구성되어 있고 연구자들은 ‘영역’과 ‘핵심 아이디어’를 도출하고, 영역과 핵심 아이디어는 연구자 회의에서 검토 후 합의점에 이르면 범주화하여 결과를 도출하였다.

7. 북한이탈주민 지원 정책과 심리지원제도 문제점 분석 및 대안 도출

북한이탈주민 관련 정책을 분석하고 정책의 문제점 도출 및 개선 방안을 제시하였다. 북한이탈주민정착지원에 관한법률, 사회보장법, 정신건강증진 및 정신질환자 복지서비스 지원에 관한 법률 등 관련법령의 정신건강 지원제도 검토하였다. 이를 바탕으로 하나원, 지역적응센터 등에서 실시되고 있는 북한이탈주민 정신건강 및 심리치료 프로그램에 대한 문제점을 도출하였다.

8. 최종결과보고서 작성

다각도로 얻어진 실태와 관련된 결과 자료를 취합하여 최종 결과보고서를 작성하였다. 설문 조사 분석 및 심층 면접한 자료 분석 결과를 토대로 북한이탈주민이 경험하는 트라우마의 특성을 토론하였다. 북한이탈주민 관련 정책을 분석하고 정책의 문제점 도출 및 개선 방안을 제시하였다. 전문가와의 집단 토론을 통하여 의견을 수렴하여 결과를 분석하였다.

제3장 선행 연구 고찰

세계적으로 5천 1백 2천만 명의 사람들이 강제 이주 되었고(2013년 말 기준), 그 중 1천 6백 7천만 명이 난민(refugees)이며, 1백 2천만 명은 망명신청자(asylum-seekers)로 추산되고 있다(UNHCR, 2014).

1. 난민과 이주민의 트라우마 경험과 스트레스

가. 이주 전 및 이주 관련 스트레스

이주를 하게 된 직접적 동기인 고향에서 경험한 외상적 사건(전쟁, 고문, 테러, 자연재해, 기근)과 거기에 더하여 친인척 관계망의 상실 및 가족 간 이별과 이동 중 경험하는 목숨을 위협하는 사건들(강을 건너는 것, 뗏목 전복, 타인의 사망 목격)이 복합적으로 발생할 수 있다. 그 과정에서 아이들은 부모나 보호자 자신이 압도되고 아이들의 정서적 요구에 신경을 써줄 수 없다는 점에서 더욱 취약하게 된다.

나. 이주 후 스트레스

이주민은 빈곤에서 벗어나기 위하여 온 경우가 많기 때문에 교육과 직업 기술 수준이 낮은 편이고 임대료가 낮은 지역에 거주하게 된다. 그래서 개인 사생활이 보호되기 어려운 인구 밀집 지역에 폭력, 범죄 위험에 노출된 안전하지 않은 생활환경 속에서 생존해야 하는 경우가 많고 탈피하기 어려운 빈곤의 사슬에 놓이게 된다.

다. 이주민에서 문화와 문화 동화(acculturation) 문제

아동은 만 3,4세만 되어도 인종 및 문화의 차이를 처음 인식하기 시작하고 만 4-8세가 되면 인종적 오리엔테이션이 발달하여 청소년기말이 되면 인종적 정체감을 자신의 정체감의 일부로 공고화 하게 된다. 인류학과 사회과학에서는 새로운 문화에 가장 효율적이고 건강하게 적응하는 방법을 동화를 통하여서라고 하는데 그렇게 되었다 할지라도 상실감과 개인 정체감의 위협을 느끼게 된다. margination은 새로운 언어, 관습, 가치관, 믿음에 동화되지 못한 현상이라 할 수 있는데 노인 이주민이나 추방자에서 흔하다. 2세대 청소년은 잘 동화되고 부모세대는 그렇지 못하며 가족 갈등, 품행장애나 물질 남용에 취약해진다.

라. 이주민과 난민의 정신건강

난민과 이주민은 외상적 경험과 스트레스에 직면하기 때문에 정신 건강에 취약하여 우울장애, PTSD, 불안장애의 높은 유병률을 보인다(Pumariega, Rothe, & Pumariega, 2005). 그 유병율은 외상적 경험의 시기, 노출 기간과 강도가 심리적 반응에 영향을 줄 수 있다. 증상과 장애의 정도에 영향을 주는 그 밖에 위험 인자로는 가난, 교육, 실업, 낮은 자존감, 신체적 건강문제 등이 있다. 소아청소년은 가족, 또래, 주변 사람들, 환경의 반응이 어떠한지에 의해 영향을 받는다. 이민자에서 성인에 비해 청소년은 증상이 적고 사회적 기능은 좋다. 이러한 결과는 발달학적인 어떤 시기는 비교적 탄력적임을 의미한다. 이민 2세대에서는 소아청소년에게 보다 만성적 스트레스 위험이 증가할 수 있다. 정신건강 문제는 인지율과 치료율이 낮아서 이민자의 성공적 정착과 기능을 방해할 수 있다. 외상적 경험자는 예외이지만 노인이 정신건강 문제가 가장 높다. 문화적 유연성 부족, 가족 및 이웃과의 고립, 건강이 위험 인자이기 때문이다.

마. 난민과 이주민에서 PTSD와 공존질환 유병율 및 관련 요인

난민과 이주민을 대상으로 실시된 정신건강관련 메타 분석 연구 결과에 의하면 우울 유병율은 노동이주민의 20%, 난민의 44%이고, 불안 유병율은 노동 이주민의 21%, 난민의 40%로 나타나 난민의 불안 우울 유병율이 더 높을 것으로 추정할

수 있다(Lindert, Von Ehrenstein, Priebe, Mielck, & Brähler, 2009).

난민들은 폭력 등의 트라우마 경험에 노출된 경우 PTSD를 비롯한 다양한 정신건강문제가 심각할 수 있는데 연구 대상의 특성, 측정 도구, 평가 방법에 따라 유병율은 다르게 보고되고 있다. 또한 정신 건강 문제의 위험요소에 대한 평가를 할 때는 이주 전 경험, 이주 도중 경험, 이주 후 경험 관련 스트레스 인자와 외상적 사건을 모두 평가하는 것이 중요하다.

전쟁 난민 관련 장기 연구(Bogic, Njoku, & Priebe, 2015)에 의하면 정신질환 유병율은 PTSD 4.4-86%, 우울증 2.3-80%, 불안장애 20.3-88%로 나타나서, 연구의 방법 및 임상적 요인에 따라 다양한데, 전형적으로 20% 이상의 유병율로 추정되고 있다. 전쟁 난민에서 정신 질환 유병율은 출신 국가 및 정착 국가의 영향을 받는다. 즉, 유고슬라비아 및 캄보디아에서 온 경우 및 미국에 정착한 경우 가장 높게 나타난다. 또한, 이주 전 외상적 경험 노출과 이주 후 스트레스 노출이 관련 요인으로 일관성 있게 보고되고 있다.

PTSD 및 우울증 정도의 예측인자를 알기 위해 688명의 난민 및 망명신청자를 대상으로 분석한 결과에 의하면 난민 신분의 결여 및 노출된 외상 사건의 수가 PTSD 및 우울증 증상의 정도와 유의하게 관련이 있었다(Knipscheer, Sleijpen, Mooren, Ter Heide, & der Aa, 2015).

스위스 정신과 외래를 방문한 외상적 경험에 노출된 난민과 망명자 대상 연구(Spiller et al., 2016)에 의하면 PTSD 증상의 정도는 신체화(somatization) 및 폭발적 분노(explosive anger)와 유의한 관련이 있었다. 여러 외상적 경험을 겪고 노르웨이에 정착한 난민 외래 환자를 대상으로 실시된 조사(Teodorescu et al., 2015)에 따르면, 만성 통증이 66%에서 있어 일반인구 유병율(19%)의 세배를 넘었고, 88%는 PTSD 진단이 내려졌다. 가장 흔한 만성 통증 부위는 머리(80%), 가슴(74%), 팔/다리(66%), 등허리(62%)로 나타났으며, 특히 여성이 남성보다 더 많은 부위에 만성 통증을 호소하였고, PTSD와 만성 통증의 공존율이 57%에 달하였다.

정신증이나 양극성 장애가 없지만 트라우마 경험을 한 난민에게서 PTSD와 우울증에 합병증으로 정신병적 증상이 발표된 바 있다(Shevlin et al., 2016). 이러한 현상들은 트라우마 경험을 한 다른 인구에서 관찰되어 PTSD의 정신병적 아형의 존재가 있다는 논란으로 이끌어지기도 하였으나 아직은 증거가 분명하지 않다.

이주 후 사회 문화 적응도 PTSD 심각도에 영향을 주는 것으로 여겨지고 있다. 스위스 연구(Schick et al., 2016)에 의하면 사회 동화(integration)의 어려움은 교육, 비자 상태와 같은 사회 인구학적 지표보다는 심리적인 증상과 밀접한 관련이 있는 것으로 나타났다. 보스니아 난민을 대상으로 한 연구(Kartal & Kiropoulos,

2016)에 의하면, 호주에 정착한 난민은 외상적 사건 노출이 PTSD, 불안, 우울의 심각도를 예측하였지만, 오스트리아에 정착한 난민은 나이, 성별, 외상적 사건 노출을 통제한 후에도 문화 동화(acculturative) 스트레스가 PTSD, 불안 증상의 심각도를 예측하는 것으로 나타났다. 이 결과는 난민의 정신 건강을 평가할 때 이주한 사회에 적응의 영향을 고려해야 함을 의미한다.

다양한 이주 인구에서 외상적 경험과 관련된 질환의 이주 후 예측 인자로는 취업상태, 경제적 어려움, 언어의 능숙함, 사회적 지지가 있는 것으로 보고되고 있다. 고문이나 전쟁을 경험한 후 관련 정서 질환이나 PTSD를 겪고 있는 환자를 대상으로 진행된 연구(Buhman et al., 2013)에 의하면 사회적 고립(연락, 배우자 및 자녀와 동거 여부)이 PTSD 과각성 증상 및 통증과 관련이 있었다.

탈출 전 전쟁 및 인권침해(human rights violations, HRVs)와 관련된 잠재적 트라우마 경험(potentially traumatic experiences, PTEs)을 한 성인 난민을 대상으로 아동기 시절 부정적 혹은 잠재적 트라우마 경험을 조사한 연구(Opaas & Varvin, 2015)에 의하면 아동기 시절에 경험한 부정적 사건들도 주요 인자이다. 즉, 탈출 전 전쟁 및 인권 침해 관련 경험은 재경험 증상과만 연관된 반면, 아동기 PTEs는 PTSD의 각성, 회피 증상 및 삶의 질과 유의한 관련이 있는 것으로 나타났다. 이 연구(Opaas & Varvin, 2015)에서는 관련성이 드러난 아동기 부정적 경험으로는 상실이나 질병보다 가족 폭력, 외부적 폭력이 특히 정신 건강 증상을 증가시키고 삶의 질을 감소시키는 것으로 분석되었다. 이 결과는 전쟁 및 인권 침해 관련 경험을 한 성인 난민을 연구하거나 치료할 때 아동기 시절에 경험한 부정적 사건들의 중요성도 고려해야 함을 시사한다.

바. PTSD 평가, 치료 및 효과 관련 요인

난민 대상 PTSD 치료 효과에 대한 연구는 매우 제한적인 편이라서 어떤 치료법이 가장 효과적인지 아직 분명한 결론은 내지 못하고 있으나 몇 가지 접근법이 전망이 있을 것으로 보고되고 있다(Buhmann et al., 2015). 먼저, 인지행동요법과 노출요법이 몇 개의 연구에서 효과적인 것으로 나타난 바 있다. 또한, 문화적으로 맞게 변형된 인지행동요법이 캄보디아인 난민에서 성공적으로 수행되었다. 그리고 서술적 노출 요법(narrative exposure therapy)이 수단, 소말리아, 르완다 출신 난민에서 약간의 성공을 거둔 것으로 연구되었다.

장기간 반복적인 외상적 사건을 경험한 생존자에게 발견되는 공존질환을 동

반한 심각한 PTSD(복합성 PTSD)는 세 가지 단계의 치료 모델을 환자 상태에 맞게 차례대로 권고하고 있다. 첫 번째 단계는 안정화와 일상생활 기술 강화에 초점을 두어야 한다. 환자의 안전성과 자원을 호전시키고 기분을 알아차리고 표현하는 능력을 발달시키고, 대인관계에서 유능감을 증가시키는 데 목적을 두고 개입한다. 두 번째 시기에는 외상적 기억을 구조적 방법으로 돌아보고 재평가한다. 이 시기에는 예를 들어 CBT, EMDR, NET와 같은 근거에 기반을 둔 외상적 경험 치료들이 적용될 수 있다. 세 번째 시기에는 치료로 인해 얻는 것들을 공고히 하고 환자로 하여금 치료에서 공동체 속 생활으로 이행을 목표로 한다.

난민의 정신건강 개입 분야에서는 외상에 중점을 둔 요법(trauma-focused therapy, TFT)과 다양한 모드의 개입(multimodal intervention)이라는 상반된 접근이 시도되고 있는데, 전자는 PTSD 증상에 목표를 두고 외상적 경험을 다루는 방법이고 후자는 현재 당면한 여러가지 문제, 예를 들어 심리사회학적 어려움, 심리학적 기능, 신체적 건강, 문화동화 문제를 다루는 방법이다. 외상에 중점을 둔 요법은 여러 연구에서 효과가 밝혀져 있고, 다양한 모드의 개입에 대해서는 아직 그렇게 정립되어 있지 못하지만 정신병리나 장애가 심하고 만성적인 난치성 난민에게 시도되어 왔다. 그래서 장기간 반복적으로 외상적 경험에 노출된 난민들의 정신건강 후유증, 즉 심각한 PTSD를 평가하고 이해하는데 있어서, 근거 기반 TFT에 다양한 모드의 개입을 병합한 통합적 맥락에 맞는 모델(Integrative Contextual Model)이 도움이 될 것으로 제안된 바 있다(Boris Drožđek, 2015).

난민 캠프에서 시리아 출신 난민들에게 안구운동 탈감각 및 재처리(Eye movement desensitization and reprocessing, EMDR) 요법을 시행한 예비 연구 결과(Acarturk et al., 2015) 그 개입방법이 PTSD 및 우울증에 효과가 있을 것으로 시사된 바 있다. 즉, 29명의 대상자를 EMDR 치료군(15명), 아무런 치료도 받지 않고 대기하는 대조군(14명)에 할당하고, 4주간 추적 완료한 결과 치료군이 대조군에 비해 PTSD 증상 및 우울 증상 점수가 유의하게 호전된 것으로 나타났다.

코펜하겐 정착 난민을 위한 정신과 트라우마 클리닉에서는 약 6개월간 약물요법 및 인지행동요법으로 구성된 매뉴얼을 따라 개입을 시행하였다(Buhmann et al., 2015a). 즉, 첫 방문 시 제 1 -2주간 의사와 사회사업가의 평가를 시행한 이후, 제 3-8주는 일주일에 한 번씩 진료를 통한 약물요법 및 정신교육(psycho-education)을 진행하고, 제9 -24주째는 진료를 한 달에 한 번씩 지속하면서 심리학자에 의한 인지행동요법(45분)을 일주일에 한 번씩 병행하였다. 그 결과 PTSD, 우울, 불안 증상, 삶의 질, 기능 수준의 유의한 호전이 있었던 것으로 보고되었다. 이때 사용된 인지행동요법은 노출요법, 마음챙김(mindfulness)요법, 수용과 전념 요법(acceptance

and commitment therapy) 요소들을 결합한 구조화된 형태이어서 트라우마 경험을 한 난민 환자 군도 구조화된 인지행동요법을 잘 수행할 수 있음을 제시하였다.

트라우마 사건에 노출된 난민들의 PTSD 치료 결과에 대한 예측인자로는 이주 후 생활 어려움 척도(the Post-migration Living Difficulty scale, PMLD)와 개입 전 정신 건강 증상, 공공 재정 지원 등이 있었다(Buhmann et al., 2015b). 덴마크에서 195명 대상으로 6,7개월 치료 연구(Buhmann et al., 2016)에서 취업 상태가 유의한 인자로 나타난 바 있다.

2. 북한이탈주민의 인권침해 트라우마 경험

가. 북한이탈주민의 사회 인구학적 특성

1990년대 중반부터 국내 입국 탈북자는 점차 증가하여 현재 약 3만 명에 이른다(2016년 6월말 입국자 기준). 그 중 여성이 약 70%를 차지하고 연령별로 20대(8,236명)와 30대(8,535명)가 가장 높은 비율을 차지하고 있다. 재북 직업별로는 '무직부양'이 13,999명이고 '봉사분야'가 1,119명으로 가장 많고, 재북 학력별로 비교하면 중학교(고등중)(20,315명) 및 인민학교(소학교)(1,943명) 졸업 혹은 중퇴자가 주류를 이룬다(통일부 홈페이지).

탈북 후 입국까지의 과정은 다양한데, 주로 제3국에서 불법 체류자 형태로 거주하거나 기술자로서 파견 근무하다가 남한으로 넘어오는 경우가 많지만, 최근에는 이미 남한에 입국한 가족의 도움을 받아 북한에서 남한으로 직행하는 사례도 증가하였다.

나. 탈북 동기

탈북한 이유에 대해서는 식량 부족, 정치적 억압, 가난에서 벗어나고자 등 다양한데 시대의 흐름에 따라 변화하는 경향이 있다. 1990년대 중・후반 '고난의 행군' 시기에는 북한이 경제적으로 극도의 어려움을 겪은 시기이므로 당시 탈북한 사람들을 면담해보면 '먹을 것 부족'과 '아파도 치료를 못 받아서'가 탈북 동기의 대부분이었다(Lee et al., 2001). 그러나 2002년 이후로는 배고픔은 많은 이유 중 하나일 뿐이고 지위 박탈, 기회 결여에 대한 좌절, 출신 성분에 기인한 정치적 피해, 이미 탈북한 사람들과 비슷한 환경에서 생활하고자 등으로 다양해지고 있다(Pokempner, Baik, & Jendrzejczyk, 2002).

다. 탈북 전 트라우마 경험

1999년경 먹을 것을 찾아서 중국으로 탈북한 사람들을 대상으로 트라우마 경험을 조사하였을 때 '먹을 것과 마실 것 부족'(93%)과 '아픈데 의학적 치료를 받지 못함'(89%) 이 가장 높게 나타난 바 있다(Lee et al., 2001). 2000년 이후 하나원 입소자들을 대상으로 시행한 연구에 의하면 재북 시절 경험한 트라우마 경험은, '굶어 죽는 사람을 목격', '공개 처형을 목격', '먹을 것이 부족하여 생명을 위협 당함', '교화소나 감옥으로 보내짐 ', '심하게 구타당함', '예고 없이 강압적으로 가족과 이별' 등이 상위 비율을 기록하였다(Jeon et al., 2008).

국가인권위원회 보고서(2009)에 따르면 북한 내에서 국제기구에 대한 접근 제한, 고문과 기타 형태의 학대, 공개처형 등이 자행되고 있으며 납치 및 강제실종, 표현의 자유, 이동의 자유, 식량권에 대한 침해가 나타나고 있으며 유엔인권기구 등은 이러한 인권침해에 대한 우려를 표명하고 권고사항을 제시하고 있다.

또한 2012 북한인권 침해신고센터 1주년 보고회 자료를 참고하면, 정치범 수용소와 교화소에서 이루어진 학대 및 노예화, 구타, 고문, 성폭력, 강제 낙태, 살해, 기타 비인도적 행위 및 조사과정에서의 고문피해 등이 신고 사례로 보고되었다.

라. 탈북 기간 동안의 트라우마 경험

탈북과정에 따라 북한이탈주민들의 트라우마 경험은 매우 다른 것으로 나타나고 있다. 특히 제3국을 통해서 탈북하는 경우 신분불안과 체포위험, 강제북송 등에 따른 극심한 스트레스를 경험하는 것으로 나타나고 있다.

탈북자들은 제3국에서 불법체류자이기 때문에 심각한 폭력에 노출되거나 범죄에 희생되어도 보호 받지 못하는 생활을 하게 되고 공안에 잡혀 북송되기도 한다. 재중 탈북 여성 대상의 연구에 의하면 체포(44%), 추방(34%), 인신매매(24%), 강간(20%), 매춘(9%) 등을 경험한다고 보고되었다(Kang 2006).

'좋은벗들'은 1998년 재중 탈북자조사 결과 탈북자들은 신체의 자유나 노동과 생계활동의 권리, 교육과 의료혜택의 권리 추구가 어렵다고 평가하였다. 특히 노동력 착취나 여성들의 인신매매, 성매매 등 각종 인권문제가 발생하고 있다고 실태를 평가하고 있다(좋은벗들, 1999).

이금순(1999)은 탈북자문제 해결 방안에서 재중탈북자 실태에 대해서도 인신매매, 노동착취, 강제송환, 건강파괴 등으로 세분하여 살펴보기 시작하고 있다. 건강

파괴에 대해서도 탈북어린이의 경우 성장발육 뿐만 아니라 정신적 피폐도 나타나고 있다고 지적하고 있다.

윤여상(2004)은 재외탈북자의 인권실태에 대해 신분 불안정, 고용불안, 저임금, 공권력의 횡포, 성을 매개로 한 협박·폭력 및 성산업 유입, 유인 혹은 강제적 인신매매, 강제송환의 심각성을 지적하고 있다.

한편, 국내 입국한 하나원 입소자들을 대상으로 2004년경 조사한 바에 의하면 ‘발각될까 봐 두려워 숨어있음’, ‘생명이 위협될 정도로 음식과 물이 부족’,’국경에서 북한 경비병의 검열’, ‘동반 탈북하던 사람과 헤어짐’,’국경에서 중국 경비병에게 체포’ 등을 많이 경험하는 것으로 나타났다(Jeon et al., 2008).

위의 연구를 통해서 볼 때 제3국을 거쳐서 입국하는 탈북자들의 경우에는 강제송환으로 인한 외상경험과 불안감이 PTSD 증상의 중요한 요인으로 지적되고 있다. 재중탈북자들은 체포된 지역 파출소→지역 감옥→국경 구류장을 거쳐 북한으로 넘어가는데 국경 근방에 위치한 북한 보위부를 거쳐 도 집결소, 교화소(단련대)에 수감되어 처벌을 받게 된다. 정치적 이유로 탈북하였거나 중국내에서 행적에 문제가 있으면 정치범수용소에 수감된다. 인신매매에 관련되었거나 정치범으로 간주되면 공개처형을 당한 사례도 다수 보고되고 있다. 보위부에 끌려가게 되면 먼저 알몸수색, 소지품 검사, 에이즈 검사를 받고 수감되는데, 인적사항, 도강시기 및 횟수, 도강이후 행적(한국인 접촉, 기독교 접촉, 한국행 시도 여부, 한국영상물 시청여부, 인신매매 피해여부)들을 조사하고 있다.

보위부 조사에서는 최소 5회 이상의 자술서를 똑같이 반복해서 작성해야 하는데 이때 자술서 내용에 차이가 있으면 심각한 폭행과 고문을 당하게 된다(Jeon et al., 2008). 일반적으로 북한의 수감시설에서는 강제노동, 배고픔, 간수들의 폭행에 시달려야 한다. 2005년 개정된 북한형법에서는 비법월경죄(탈북죄)에 대해 교화 3년형 이상을 명문화하고 있으나 정치적 이유로 탈북했거나 탈북이후 중국에서 한국인 또는 현지 교회와 접촉하지 않은 경우에는 보위부나 도(道)집결소에서 뇌물을 쓰면 풀려나는 경우도 있다. 즉 교회, 언론, 한국 정보기관과 연류된 증거가 없을 경우 청진에 위치한 함경북도 도(道)집결소에서는 인민폐 3천 위안 이상이면 ‘병보석 가석방’으로 내보내주고, 인민폐 6천 위안 이상이면 집결소에서 사건기록 문건 자체를 폐기시키고 몰래 석방시켜주는 경우가 있었다. 하지만 뇌물을 전혀 쓰지 않는 재범은 교화 3년~5년 형에 처해지고 있는 것이 현실이다.

대부분의 탈북자들은 최종적으로 한국으로 들어오지만 이들이 중국에 체류하는 동안 겪게 되는 끔찍한 인권피해는 고스란히 정신적 육체적 상처로 남게 된다. 결국 이들의 정신적 육체적 트라우마에 대한 치유비용은 고스란히 우리 사회의 몫

으로 남겨지게 된다. 탈북자의 국내통합을 위해서도 중국내에서 자행되는 심각한 인권침해와 특히 강제송환으로 인하여 발생하는 인권문제에 대한 우리 정부와 시민사회의 노력이 있어야 하며 그러한 노력에 대한 탈북자 사회의 올바른 평가가 있어야 할 것이다.

마. 성별 인권침해 및 트라우마 경험의 차이

선행연구에 의하면 여성들의 경우 일반적으로 남성보다 PTSD 유병률이 직행보다 높게 나타나고 있다(김현아, 2004). 이러한 현상의 배후에는 여성이 가지는 정신적 민감성 등 여러 가지 생물학적, 환경적 요인이 있으나 탈북자 가운데 높은 여성비율과 중국내 탈북여성에 대한 흡수요인이 있다는 점을 주목해야 할 것이다. 특히 재중 탈북여성의 경우 상당수가 인신매매의 피해자로 우려되고 있다.

인신매매는 “위협 혹은 폭력이나 기타의 강제력, 납치, 사기, 유인, 피해자의 취약한 지위 활용, 타인에 대한 통제력을 가진 자의 동의를 얻어내기 위해 금전 혹은 혜택을 주고받는 행위 등에 의해 ‘착취’의 목적으로 사람을 모집, 운송, 이전, 은신, 접수하는 행위”를 의미한다. 여기서 착취(exploitation)란 “매춘 및 성적 착취, 강제노동 및 서비스, 노예상태에 준하는 행태 및 노예제, 장기절제” 등을 포함한 개념이다.

인신매매가 단순한 밀입국과 다른 점은 불법적인 국경이동 주선 후에도 지속적인 착취가 이루어진다는 점이다. 중국 내 북한여성에 대한 잠재적인 수요로 인해 탈북여성에 대한 인신매매 현상은 지금도 끊이지 않고 있다. 탈북 여성들은 주로 매매혼의 형태로 거래되고 있으나, 매춘이나 성적대상을 전제로 매매되는 경우도 있다. 2006년 한국의 한 시민단체가 중국에 체류 중인 가임기 탈북여성 127명에 대한 실태조사를 벌인 결과 74명(58%)가 ‘인신매매를 당해본 경험이 있다’고 답했으며 실제로는 더 많은 탈북여성이 인신매매를 당했던 것으로 추정되고 있다(박인호, 2008).

유엔 북한인권 COI 최종 보고서(UNHCR, 2014)에 의하면 북한 내 여성인권문제도 심각한 것으로 지적되고 있다. 특히 가부장적인 사회적 환경 내에서 만성적인 경제난 때문에 여성들의 경제 활동이 증가함에 따라 북한여성들은 가정과 경제활동 주체로서 이중적인 부담을 지고 살아가야 한다. 이 과정에서 북한여성 들은 경제적 활동 뿐 아니라, 정치적, 사회적 영역에서도 차별과 인권침해 요인들에 노출되게 된다. 탈북여성의 탈북 및 정착과정에서의 인권침해 실태조사(국가인권위원회, 2009)

에 의하면, 탈북여성의 공간별 인권침해 현황은 <표 3-1>과 같이 요약된다.

한편 중국내 탈북 남성들은 구직난뿐만 아니라 임금체불 및 노동력 착취라는 이중고에 시달리고 있다. 중국 농촌에서 일하는 탈북 남성들의 경우 사실상 임금은 거의 못 받으며 숙식만 제공받거나 숙식 제공 없는 저임금(일당 인민폐 5~10위안)에 종사하고 있다. 탈북자라는 이유로 약속된 임금을 못 받는 경우가 많아 탈북남성의 노동의욕을 감소시키는 주요한 원인으로 손꼽히고 있다.

〈표 3-1〉 탈북여성의 공간별 인권침해 목록

공간	공간의 특징	인권상황 또는 인권침해 패턴
북한	• 1990년대 이후 ‘경제난의 일상화’와 결합하면서 인권침해의 유형・규모・방식이 다양화・확대・구조화 됨<br>• 정치적 지배구조와 성별 위계구조의 결합에 따른 여성의 ‘이등국민’ 대우<br>• 남성중심 분배체계에 따른 여성의 지위 하락과 생계 책임 전담<br>• 남성중심의 이데올로기와 가족주의적 국가관에 따른 여성의 종속화	• 1990년대 경제난 이후 국가와 남성의 차별과 폭력, 동원과 착취에 노출된 북한 여성<br>• 교육과 노동 영역에서 나타나는 불평등한 기회와 계급적 대물림<br>• 경제난 이후 가족을 생계를 위해 시장에서의 경제활동에 내몰리는 여성<br>• 여성의 시장 내 생계활동에 기생하고 여성들을 성적으로 착취하는 남성과 관료<br>• 경제난 이후 더욱 열악해진 모성 보호 조치와 가정 내 폭력
중국 및 제3국	• 난민 인정을 받지 못하는 불법 입국자・체류자<br>• 색출・단속・강제송환의 위험 상존<br>• 준비되지 못한 불법 이주에 따른 열악한 생계 전략	• 국적 없는 삶: 신분증을 만들 수 없는 생활, 공안의 추격과 타인에 대한 경계, 일할 수 있는 권리의 박탈, 불법체류자의 지위를 악용하는 브로커의 인권 침탈<br>• 일상생활의 대인관계와 경제활동에서 정체성 부정과 인권의 박탈: 비인간적 대우, 자아정체성 부정, 의식주 등 기본 생존권 박탈, 언어폭력<br>• 젠더와 트라우마: 모성권의 침탈, 성폭력, 매매혼, 인신매매
한국	• 남한 정부의 정착・교육・취업 지원 제도와 ‘국민 만들기’<br>• ‘인적 자본’을 갖추지 못한 열등한 소수자<br>• ‘못 살고 적대적인 나라에서 온’ 소수자<br>• 육체적・정신적 상처를 안고 있는 삶	• ‘국민 만들기’ 과정에서의 인권상황: 조사과정에서의 낙인과 상처, 트라우마에 대한 치료・지원의 부재, 차별을 재생산하는 적응교육, 수동적 존재로 만드는 길들이기 식의 정부 지원제도<br>• 일상에서의 차별과 배제: 모르는 게 너무 많은 사람들, 사회에 짐이 되는 사람들, 믿을 수 없는 사람들, 아픈 데가 많은 사람들, 마음은 항상 이산가족에 가 있는 상황, 가부장적 성역할의 지속

출처: 2009년도 국가인권위원회 북한인권 실태조사 연구용역 보고서(탈북여성의 탈북 및 정착과정에서의 인권침해 실태조사)

3. 북한이탈주민의 정신건강 및 사회적응

가. 인권침해 트라우마 경험과 북한이탈주민의 정신건강

탈북자들은 불안, 우울, 외상 후 스트레스 장애(Posttraumatic stress disorder, PTSD)와 같은 주요 정신 질환과 연관되어 있는 것으로 알려져 있다. 중국에 있는 탈북자들 170명의 정신건강 상태를 조사하였을 때 PTSD 유병율은 약 56%로 높은 편이었고, 불안(90%)과 우울(81%)도 더욱 높게 나타난 바 있다(Lee et al., 2001).

국내 입국 후 시행한 연구로는 하나원 입소자를 대상으로 한 연구에 의하면 1999년에 입국한 95명 연구 대상자의 27.3%가 PTSD를 겪는 것으로 나타났다(강성록, 2000). 하나원과 지역사회 탈북민을 대상으로 한 PTSD 유병율 연구(김현아, 윤여상, 한선영, 2007) 결과로는 302명 중 완전(full) PTSD가 26.15%이고, 부분(partial) PTSD는 50.66%로 보고되었다. 1년 미만의 지역 사회 탈북민 200명에서는 PTSD가 약 30%로 추정되는데, 여성이 남성보다 유의하게 높은 것으로 알려져 있다(Jeon, 2005). 장기간 추적 연구(홍창형 등, 2006)한 결과에 의하면, 3년 추적 결과 부분(partial) PTSD는 31.8%에서 5.3%으로 감소하였고, 완전(full) PTSD는 27.2% 에서 4.0%으로 감소하여 처음에 부분 혹은 완전 PTSD로 진단되었던 사람들의 88.8%가 회복되는 양상을 보였다고 한다. 이중 3년간 PTSD가 만성적으로 지속되었던 사람들은 모두 여성들이었다. 그러나 탈북민들의 PTSD 경과 및 예후에 대해서는 체계적인 추적 연구가 필요한 상황이다. 한편 정신건강의학과를 방문한 북한이탈주민 임상군을 대상으로 한 연구(한나영 등, 2015)에 따르면 53%가 PTSD로 진단되어 매우 높게 나타났다. 우울 증상은 남한에 거주한지 1년 이상 지난 북한이탈주민을 대상으로 연구한 결과에서 남성의 33.1%와 여성의 36.1%에서 임상적 수준을 나타내었다고 한다(Jeon et al., 2009).

북한이탈주민(593명)과 남한 원주민(2,373명)과의 정신건강을 비교한 연구 결과에 의하면, 북한이탈주민의 우울, 불안, 자살 사고가 유의하게 높음을 알 수 있었다(Shin, Lee, & Park, 2016). 선행연구에 의하면 북한이탈주민들의 PTSD증상은 남한정착 이후 많이 완화되어지는 것으로 조사되지만, 우울수준과 불안수준은 유의하게 높아지는 것으로 나타나고 있다. 그러나 이들의 외상경험 자체는 PTSD증상이나 우울, 불안증상과 모두 긴밀한 관계성을 나타내고 있어 외상경험이 북한이탈주민의 정신건강 상에 있어서 매우 중요한 요인임을 지적하고 있다(전우택, 김연희, 조성아, 2010). 이러한 외상경험 가운데 구금, 고문 등 폭력피해나 성폭력 등 중대한 인권침해 경험과 친밀한 관계의 상실 등은 북한이탈주민의 정신건강에 매우 심각한 영향

을 미치는 요인으로 조사되고 있다.

우울과 불안 이외에 북한이탈주민들에게 있어서 나타나는 또 하나의 임상적 특징은 높은 신체화(Somatization) 경향이다. 이들은 주관적 건강에 대한 호소로 심리적 문제를 대체하는 경향성이 높게 나타나고 있으며 MMPI에 있어서 Hs척도가 유의하게 높게 나타나고 있다(김현아, 전명남, 2003). 따라서 이들은 정신적 문제를 신체적 증상으로 인식하여 정신과 진료를 회피하고 일반 병원을 전전하여 적절한 치료시기를 놓치는 경우가 발생하고 있다(이영렬, 2014).

탈북자를 대상으로 한 정신건강 연구 결과는 다양하게 나타나는데 그 이유에 대해서는 몇 가지가 제시되고 있다(김연희, 전우택, 조영아, 2010). 첫째, 연구에 참여한 대상자의 차이가 유병율에 영향을 준다. 즉, 하나원 입소자, 지역사회 거주자, 재외공관, 제3국에 거주자 등 상황에 따라 차이가 날 수 있다. 둘째, 표집 방법의 문제이다. 북한이탈주민에 대한 공개적인 접촉이 어렵고, 연구자가 개인적으로 알고 있는 기관을 중심으로 설문을 하는 편의적 방법을 사용하다 보니, 연구 결과를 일반화하는데 한계가 있고, 지역적 차이나 입국 년도에 따르는 차이와 같이 정책적인 유용성을 가질 수 있는 세밀한 분석을 하는 것이 어렵다. 셋째, 연구에 따라서 다양한 정신건강 척도가 사용된다는 점이다. 즉, 기존에 남한 일반인들을 대상으로 타당화가 이루어졌거나 북한이탈주민을 대상으로 사용된 적 있는 척도들을 자신의 연구에 맞게 수정하여 사용하다 보니, 다른 연구와 비교가 어렵다는 단점을 가지고 있다.

북한이탈주민의 PTSD 등 정신건강문제의 관련 요인으로는 북한이나 탈북 과정에서 겪는 트라우마 경험, 제3국에서의 장기간 체류, 남북한의 문화적 차이, 남한 정착 후의 자립의 문제 등 다양한 여건들이 고려되고 있다.

PTSD 진단은 질병 및 죽음 관련 트라우마 그리고 폭력관련 트라우마와 유의한 관련이 있는 것으로 나타났다는 결과가 있는 반면(김현아, 윤여상, 한선영, 2007), 탈북 과정 중 트라우마 사건을 '육체적 트라우마', '발각 및 체포와 연관된 트라우마', '가족과 연관된 트라우마', '배신과 연관된 트라우마' 4개의 요인으로 나누었던 연구에 의하면 그 중 '가족과 연관된 트라우마'가 PTSD와 통계적으로 유의한 관련성이 있었다고 보고되었다(홍창형 등, 2006). 이 연구에서 북한이탈주민(199명)에서 감정표현불능(alexithymia) 정도가 경험한 트라우마와 PTSD 의 관련성을 조정하는 것으로 나타났다. 즉, 감정표현불능 정도가 높은 경우 트라우마 경험 수가 많을수록 PTSD 정도가 높았고, 감정표현불능 정도가 낮은 경우는 그렇지 않은 것으로 분석되었다. 그래서 트라우마를 많이 경험할수록 감정을 인지하고 표현하는 것이 PTSD 증상을 줄이는데 필요함을 알 수 있다.

이처럼 여러 연구들에서 북한이탈주민들이 북한내와 탈북과정에서 겪은 심각한 인권침해, 트라우마 경험이 남한입국이후 PTSD증상과 삶의 질에 부정적인 영향을 미친다고 보고하고 있다. 즉 이러한 외상경험 요인은 우울과 불안 등 심리적 증상으로 지속되며 적응과정에 부정적인 변인으로 작용하고 있다. 북한이탈주민들의 외상경험 중에 공권력에 의한 폭력, 강제북송, 성폭력 등 극심한 인권침해는 매우 중대하고 심각한 요인으로 나타나고 있다는 점을 확인할 수 있다.

북한이탈주민을 대상으로 한국형 마음 챙김에 기반한 스트레스 감소 프로그램(Korean Mindfulness-Based Stress Reduction, K-MBSR)의 효과성을 검증한 연구(최현옥, 2011) 결과는 13명을 K-MBSR 치료집단에 6명, 통제집단에 7명을 할당하여, 치료집단은 총 8회기에 걸쳐 K-MBSR 프로그램을 실시한 것이 있다. 프로그램 종료 후 사후 검사가 실시되었고, 4주 후 추적 검사가 이루어졌다. 그 결과, K-MBSR 프로그램에 참여한 북한이탈주민 집단이 통제집단에 비해 경험회피의 한 수준인 수용행동능력이 유의하게 감소되었다. 그러나 PTSD 증상인 재경험 증상, 회피・마비 증상, 과각성 증상과 경험회피의 다른 수준인 사고억제 및 수치심에서는 유의한 변화가 나타나지 않았다.

정신과 전문의 김병창(2010), 이영렬(2014) 등은 북한이탈주민의 정신건강과 건강한 적응을 위한 통합적인 정신건강 및 보건의료 정책을 모색할 시점이라는 점을 지적하고 있다. 김병창(2010)은 하나원의 개별 정신과 진료기록과 심리검사 데이터의 지역 정신건강 지원체계와의 공유를 제안하고 있으며 특히 이영렬(2014)은 하나원 근무경험자를 포함하여 북한이탈주민 치료경험이 풍부한 정신과 전문의 2인과 북한이탈주민 3인에 대한 심층면접을 통해서 다음과 같은 구체적인 통합 정신건강 지원체계안을 제시하고 있다.

❍ 첫째 보건복지부와 국립정신병원을 중심으로 하는 북한이탈주민 정신건강 중점클리닉 및 특성화된 조사-연구-치료 통합시스템 구축한다.

❍ 둘째 5개 국립정신병원, 31개 하나센터, 185개 지역 정신건강증진센터, 중앙정신보건 사업지원단 간에 “조기발견-진료의뢰” 연계시스템 구축한다.

❍ 셋째 북한이탈주민 적응지원 및 통일이후를 대비한 북한이탈주민 정신보건인력 양성한다.

하현선(2016)은 북한이탈주민 보호 및 정착지원사업을 평가하는 보고서를 통해서 현행 전문상담사 제도의 미비점과 한계점을 적실하게 지적하고 있다. 특히 전문상담사제도의 채용기준이나 능력 면에서 전문성이 떨어지고 운용제도 면에서도

행정적 혼선과 유기적인 협조체제가 부족한 점을 지적하고 있다.

정신건강의학과를 방문한 북한이탈주민의 81.8%가 공존 신체 질환이 있는 것으로 나타나 이들에게 단순한 심리적 지원이 아닌 의료적 차원에서 통합적으로 정신건강에 대한 개입이 필요함을 알 수 있다. 선행연구를 통해서 남다른 외상경험과 특히 극심한 인권침해를 경험한 북한이탈주민의 정신건강 문제가 매우 심각한 상태라는 점은 널리 인정되고 있다. 북한이탈주민의 남한사회 정착과 통합을 위해서 정신건강을 유지하고 적절한 정신의학적 치료를 받을 수 있는 지원체제를 정비하고 개선하는 과제는 매우 시급하고 중요한 사안이다.

나. PTSD 등 정신건강과 북한이탈주민의 사회적응

적응이란 개인이 환경과의 상호작용 속에서 기능을 원활히 수행해 나가는 과정을 의미하는데, 이때의 기능이란 신체적, 심리적, 사회적 차원의 기능이 모두 포함된다. 이러한 기능과 적응능력들은 서로 분리되어 있는 것이 아니라, 서로 상호작용을 하면서, 개인의 적응과정에 영향을 준다. 사회적 적응은 직업적 기능을 포함하여, 남한사회에서의 소속감, 자신이 일상생활에서 관련된 일들을 함께 잘 처리 할 수 있는 능력들을 의미한다. 정정애(2013)는 통일정책연구의 논문에서 탈북동기에 따른 남한사회의 심리적 적응이 달라진다고 하였다. 이러한 북한이탈주민의 심리적 특성에서 불안은 중요한 특징 중 하나이며, 외상 후 스트레스 장애와 같은 불안은 체험할수록, 남한환경에 대한 환경적 적응과 사회적 적응에 어려움을 겪게 됨을 알 수 있다. 이와 관련된 구체적 연구 결과들은 아래 <표 3-2>와 같다

남한 사회에 정착하여 일상생활을 하고 있는 북한이탈주민 중 정신과적 문제를 호소하며 정신건강의학과를 방문한 임상군을 대상으로 연구한 결과에 의하면 PTSD군이 Non-PTSD군에 비해, 삶의 질이 유의하게 낮았고 남한 사회에서의 적응 곤란이 유의하게 높은 것으로 나타났다. 특히 PTSD군에서는 '남한 사람들에게 무시당하는 느낌 혹은 경험'이 삶의 질 중 심리적 영역 및 사회적 영역과 각각 유의한 부적 상관관계가 있는 것으로 나타나 PTSD가 심리적, 대인관계, 사회적응 영역에 유기적 영향을 미칠 수 있음을 보고하였다. 그리고 PTSD가 있을 경우, 결혼 만족도가 낮고 사회 적응 정도가 낮은 것으로 알려져 있다.

특히 외상 후 스트레스 장애와 직업적 손실과 관련된 연구는 최근 9.11테러 이후 장기적 결과에 대한 연구들이 발표되고 있으며 이에 대한 결과로는 9.11테러 당시 World Trade Center에서 구호 등의 작업을 했던 사람들 중 PTSD의 공존질환이 있을 경우, 은퇴나, 실직의 위험도가 높았으며, 만성적으로 PTSD를 경험할수록 위험도가 높음을 알 수 있었다(표3-2).

2001년 9월 11일 세계무역센터의 테러이후, 정신건강과 관련된 여러 연구들이 발표 되었다. Niles et al.(2011)등은 NYC의 소방관의 9.11테러 이후, 7년 동안의 accidental disability 은퇴의 47%가 9/11 연관된 손상이나, 질병으로 인한 것이라고 하였다. 자신 스스로 인식하는 나쁜 건강상태와 조기 퇴직 또는 실직과는 연관이 높으며, 이러한 연관성은 유럽, 호주를 포함하여, 미국 등 다양한 나라에서 관찰되었다. 일반군과 생존 암환자의 경우, 우울증, 불안, 여러 정신건강문제들이 조기 퇴직이나, 실직과 연관이 높다는 연구들이 있었다. 뿐만 아니라, 다른 신체적 질환과

함께 PTSD가 동반되어 있는 경우, PTSD가 동반되지 않는 경우보다, 수입이 감소하는 결과를 보였다(그림3-1).

최근 북한이탈주민의 탈북원인으로 경제적 이유와 삶의 질을 의미있게 생각하는 하는 경우가 늘어나고 있다. 지금까지 여러 연구에서 PTSD가 급성기 뿐만 아니라 만성적으로 심리적 사회적 기능에 부정적 영향을 준다는 것을 알 수 있었다. PTSD는 만성적 경과를 밟을 경우, 우울증 등의 다른 정신질환과의 공존이 높아지며 이는 더욱 사회적 기능의 적응에 영향을 주고, 삶의 질을 저하시키게 된다. 궁극적으로 북한이탈주민의 초기정착단계에서의 지원에서 경제적 지원 외에 탈북과정, 재북시기에서의 인권적 문제 및 외상으로 인한 여러 정신건강의 문제에 대한 의료적이고 전문적 접근이 후기 정착에서의 사회적 적응 및 심리적 적응을 돕는데 중요함을 알 수 있다. 향후, 사회적 적응을 통해, 북한이탈주민들은 남한사회에서의 소속감을 느끼고, 신체적 심리적 건강을 유지하며, 삶의 질을 높일 수 있다고 생각된다.

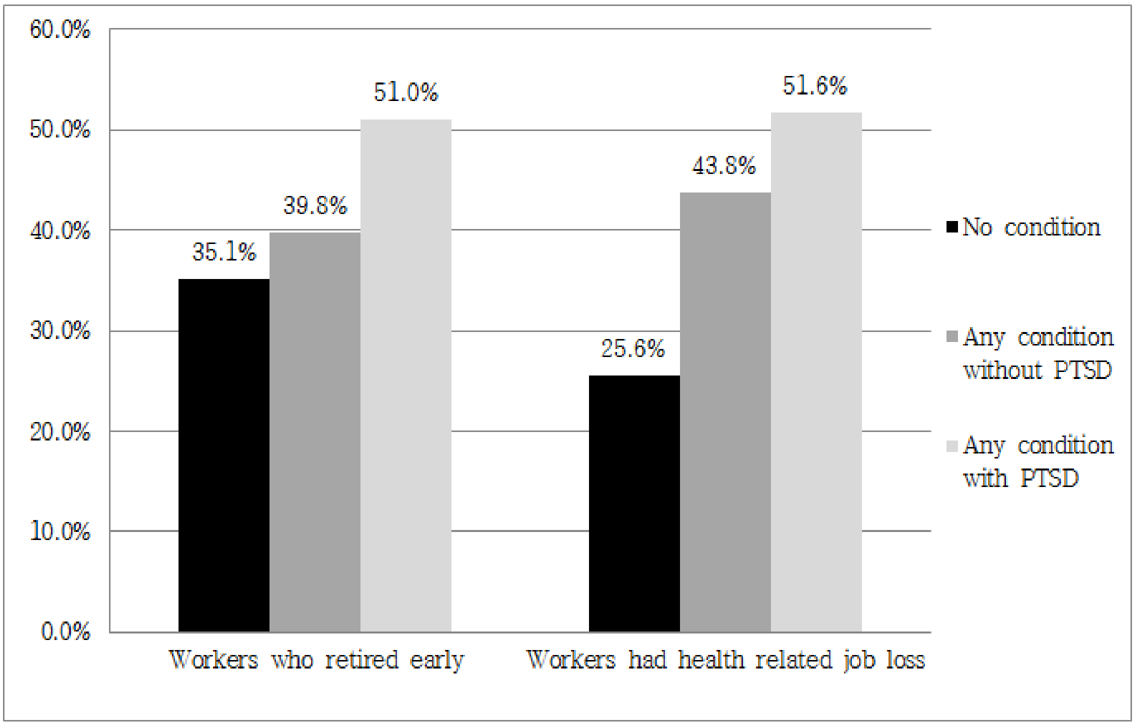

<그림 3-1> Percentage of those with income drop between years 2002 and 2010 by chronic health condition*(*including heart disease, diabetes, depression, anxiety, lung disease, and asthma) : among two rescue and recovery worker subgroups.

〈표 3-2〉 외상 후 스트레스증후군과 사회적 기능 저하의 연관성과 관련한 연구

저 자	발행년도	제목	목적	대상	방법	결과
**Yu et al.**	2016	Impact of 9/11-Related Chronic Conditions and PTSD Comorbidity on Early Retirement and Job Loss Among World Trade Center Disaster Rescue and Recovery Workers.	세계무역센터 9.11 테러 이후 60 세 이전에 다양한 직종의 구조 구호 활동을 한 workers 에서 은퇴를 하거나 , 실직을 함으로써 발생하는 장기적 경제적 영향력에 대한 연구	The WTC Health Registry surveys 에 참여한 workers (7,662명)	Logistic regression models examine the association of 9/11-related health and labor force exit	PTSD 의 공존유병이 있는 경우, 이른 은퇴나 실직과 연관성이 높았으며 만성적으로 PTSD 를 경험하고 있는 사람들일수록, 이른 실직과 은퇴의 위험도가 높았음.
**Stellman et al.**	2008	Enduring Mental Health Morbidity and Social Function Impairment in World Trade Center Rescue, Recovery, and Cleanup Workers :The Psychological Dimension of an Environmental Health Disaster	세계무역센터 WTC 의 종사자 코호트에서 9.11 테러 이후 , 61 개월간의 정신건강문제 , 사회적 기능의 저하 , 정신질환의 공존율을 파악하기 위함	A large cohort of WTC rescue, recovery, and cleanup workers (10,132명)	Self-administered mental health questionnaire (10 to 61 month after)	정신건강문제 유병률<br>PTSD : 11.1%<br>probable depression: 8.8%<br>probable panic disorder: 5.0%<br>substantial stress reaction : 62%<br>PTSD point prevalence : 13.5% -> 9.7%<br>(declined over the 5 years of observation)<br>정신질환 및 정신건강문제가 공존할수록, 사회적 기능의 저하가 광범위하게, 지속적으로 있을 가능성이 높다.
**Ainamani et al.**	2017	PTSD symptom severity relates to cognitive and psycho-social dysfunctioning - a study with Congolese refugees in Uganda	PTSD 의 중등도가 높을 수록 , 실행기능 , 작업기억 (working memory), 일상생활의 사회적 기능의 저하와 연관성이 있을 것임	323 congolese refugees (mean age: 31.3years)	1. Exposure to traumatic events<br>2. PTSD symptom severity (PTSD symptom scale interview)	PTSD 의 중증도는 사회심리적 기능의 저하와 연관성이 있으며,<br>외상과 관련된 정신건강문제는 향후 빈곤의 가능성을 높인다고 생각할 수 있음.

					3. Executive functioning (Tower of London)<br>4. Working memory performance (Corsi block tapping task)<br>5. Psycho-social dysfunctioning (Luo functioning scale)	
**Shona C Fang et al.**	2015	Psychosocial Functioning and Health-Related Quality of Life Associated with Posttraumatic Stress Disorder in Male and Female Iraq and Afghanistan War Veterans: The VALOR Registry	PTSD 와 관련된 심리 사회적 기능의 저하 및 삶의 질 저하에서의 성별 차이에 대한 연구	1,530 United States Iraq and Afghanistan war veterans	1. the Inventory of Psychosocial Functioning (IPF)<br>2. Mental and physical HRQOL with the Veterans RAND 12-item Health Survey (VR-12) Mental and Physical Component Summary scores	PTSD 가 있는 군은 , 사회심리적 기능의 손상이 더 심했음. 이는 성별의 차이가 없었음.<br>PTSD 가 있는 군은 좀 더 낮은 정신건강부분의 삶의 질 저하 점수를 보였고, 이는 성별차이가 없었음.<br>신체적 삶의 질 저하는 점수는 여성에서 관찰됨 .
**Christin M. Ogle et al.**	2013	The Impact of the Developmental Timing of Trauma Exposure on PTSD Symptoms and Psychosocial Functioning Among Older Adults	외상사건에 노출의 부정적 결과가 외상을 경험한 시점(어린시절 부터 노년기까지)과 연관성이 있는지 여부 .	12th wave (2008 -2010) of the University of North Carolina Alumni Heart Study (UNCAHS), 지역사회거주 노인(1,995명)	1. The traumatic life events Q.<br>2. The PTSD check List-Stressor specific Version<br>3. The Subjective Happiness scale<br>4. Social Support and Coping ability scale	어린시절 외상적 경험을 할수록 심한 PTSD 증상을 나타내며, 주관적 행복감을 덜 느낌.<br>어린시절 외상적 경험을 할수록 심리사회적 기능의 저하, 심리적 대처전략의 저하와 연관성이 있음.

4. 해외 트라우마센터 사례

참고로 해외 트라우마 센터 사례를 검토해 보면 주로 국가적 재난에 대응하여 출동하거나 트라우마 후유증 치료를 지원하기 위하여 트라우마 센터를 운영하고 있다. 일본의 경우, 재난응급정신의료지원팀(Disaster Psychiatry Assistant Team)을 구성하여 재난 시 법적 근거에 의하여 조직화된 정신의료팀이 정부 지원을 받아 출동하여 일정기간 기능을 수행하는 형태이고 미국의 경우 적십자사를 중심으로 민간 자원봉사조직이 상설화 운영되고 있고, 재난정신지원단(Disaster Psychiatry Outreach) 같은 민간정신건강 전문가 자원봉사 조직 등이 활동하고 있다. 보건복지부(2014)에서 수행한 '해외 방문 현황 조사를 통한 정신건강트라우마센터 모형 및 운영체계 개발'용역 연구 결과가 아래에 정리되어 있다.

가. 일본

1) 재해시마음의정보지원센터(災害時こころの情報支援センター, National information center of disaster mental health)

가) 배경

- 2011년 3월 11일 일본 도호쿠(東北) 지방에서 발생한 동일본대지진 후 처음으로 대규모의 <마음의 케어팀(心のケアチーム)>을 피해지역에 파견하였다.
- 후생노동성[1]의 책임하에 3,000명 이상의 정신건강의학과 의사, 간호사, 임상심리사, 사회복지사, 행정직원이 지원활동을 전개하였다.
- 피해받지 않은 지역에서 피해지역을 지원하는 개념으로, 동경도의 경우 주요피해지역인 이와테현에 1년간 62개팀 총 699명을 파견하여 1년간 지원을 유지하였다.
- 동일본대지진 경험 후 국가적 규모로 재난정신건강체계를 설립하여 재난에 대해 체계적이고 적극적으로 대응해야 할 필요성이 제기되었다.

나) 조직 및 예산

- 독립행정법인 국립정신・신경의료연구센터(独立行政法人 国立精神・神経医療研究センター, National Center of Neurology and Psychiatry (NCNP)[2]에 설립되었다. NCNP의 1년 예산은 한화로 약 1,659억이며, 재해시마음의 정보지원센터는 이중 20억원 정도를 사용하고 있다. 별도로 위생노동성에서 동일본대지진 피해지역에 18억엔(한화로 180억)을 지원하여 마음의 케어센터를 운영한다.

1) 우리나라의 보건복지부에 해당
2) 우리나라의 국립정신건강연구원에 해당

다) 인력

- 정신건강의학과 전문의 요시하루 김(金吉晴)을 센터장으로 4개의 섹션 치프를 두고 있으며, 6명의 정신건강의학과 전문의와 임상심리사, 간호사 팀으로 구성된다.
- PTSD 및 외상 전문 연구원이 10명 이상 근무하도록 규정되어 있다.

라) 기능

- 정신건강서비스를 총괄하고, 온라인 시스템으로 재해정신보건의료정보 지원시스템(災害精神保健医療情報支援システム, Disaster Mental Health Information Support System(DMHISS))을 구축하여 전국적인 개입과 실적을 관리한다.
- 재난파견정신의료팀(災害派遣精神医療チーム, Disaster Psychiatric Assistance Team(DPAT)을 2013년과 2014년에 걸쳐 법제화하여 이들의 활동을 지원하고 안전을 보장하고 있다. 자연재해가 많은 일본에서는 재해 시 의료 및 복지기관의 기능이 마비되므로 이러한 파견의료팀이 재난지원에 핵심적인 역할을 수행한다. 현장의 사례관리는 보건소의 보건사(정신보건간호사)가 담당하며, 전체 콘트롤타워의 감독하에 주로 일본적십자가 자원봉사를 진행한다.
- 본 기관 설립 이후 이와테 등 주요피해지역에 위치한 정신보건복지센터[3]에 트라우마센터가 설립되었다.

2) 효고현마음의 케어센터(兵庫県こころのケアセンター)

가) 배경

- 1995년 1월 17일 발생한 한신-아와지 대지진을 계기로, 재난 생존자 및 유가족들에 대한 지속적이고 전문적인 개입의 필요성이 제기되었다.

나) 조직 및 예산

- <공익재단법인 효고 재난기념 21세기 연구기구(公益財団法人ひょうご震災記念 21世紀研究機構)>의 관리 감독을 받는 독립법인의 형태로 운영된다.
- 총예산은 19억이며, 효고현에서 전체 예산의 90%를 지원한다.

다) 인력

- 정신건강의학과 전문의 3명(센터장 1인, 부센터장 1인 포함), 임상심리사 4명, 정신보건간호사 1명(상담실장), 정신보건사회복지사 2명, 행정직원 10명이 근무한다.

라) 기능

- 기존의료 및 복지체계에서 감당할 수 없는 트라우마 환자에 대한 회당 2-3시간 이상의 충분한 진료(약물치료 포함)와 상담서비스를 제공하고 있다.

3) 우리나라의 정신건강증진센터에 해당

나. 미국

1) 세계무역센터건강프로그램(World Trade Center Health Program, WTCHP)

가) 배경

- 사고 직후인 2001년 9월 12일 부터 2004년 12월 30일 까지 자유프로젝트라는 이름의 위기상담프로그램과 심리지원이 시행되었다. 서비스를 위해 연방재난관리청(FEMA)으로부터 1억5천5백만 달러를 지원 받아 120만 뉴욕 시민에게 위기상담 및 대중교육 서비스를 지원하기 위해 시행되었다.
- 9/11 사고 당시 해당지역에서 구조, 복구 및 정리 작업한 요원 및 자원자, 그리고 해당지역의 거주한 생존자의 9/11연관 건강문제에 의료지원을 해주는 세계무역센터건강프로그램(WTCHP)은 자드로가법안(Zadroga Act)에 의해 2011년 1월에 제정되었다.
- 2011년 자드로가법안의 통과 이후 42억 달러의 예산으로 장기적 치료 제공한다.
- 조직 및 예산
- 총괄적인 업무는 보건복지부(HHS)의 질병관리본부 내의 국립산업안전보건연구원(NIOSH) 에서 관장한다.

나) 인력

- 관련 사업에 대해 민간용역방식으로 진행된다.
- 과학기술위원회를 통해 17명의 자문위원이 대상자의 범위와 연구관련 사항을 조언한다.

다) 기능

- 건강등록의 경우 현재(14. 7. 31) 뉴욕시에서만 51,674명이 등록하였고, 모든 등록자의 신체건강 및 정신건강 검진을 20년 이상 추적관찰 할 예정이다.
- 2014년 현재 치료프로그램에 7,735명(2013년 8월부터 2014년 8월까지 1,164명 등록)이 등록되었고, 이중 40% 정도에서 정신건강의학과적 개입이 필요하고 60%이상에서 PTSD, 불안 및 우울 증상을 보였다.

다. 호주

1) 호주 외상후 정신건강센터(The Australian Centre for Posttraumatic Mental Health, ACPMH)

가) 배경

- 1917년 재향군인과 유가족을 위한 송환위원회가 시초이다. 1995년 보훈처 산하에 국립 전쟁 관련 PTSD 센터(the National Centre for War-Related PTSD)가 설립되면서 PTSD에 대한 지원이 시작되었다.

- 2000년 7월, PTSD에 대한 지원 대상을 재향 군인 외에 정신적 외상을 입은 일반 시민으로 확대하면서 호주 외상후 정신건강센터(The Australian Centre for Posttraumatic Mental Health, 이하 ACPMH)로 개편되었다.
- 국가의 재정적 지원 및 인적 참여를 통한 안정성, 비영리기구(NPO)의 독립성과 유연성, 그리고 정신의학 전문가의 주도적 참여를 통한 전문성 확보 등이 ACPMH의 특징이라고 할 수 있다.

나) 조직 및 예산

- 보훈처 및 방위군, 맬버른 대학교가 공동으로 운영위원을 구성하는 비영리기구(Not-for Profit Organization, NPO)로서의 독립적인 법적 지위를 가지고 있다.
- 운영 및 재정적 지원의 상당부분을 보훈처와 방위성 등 정부기관에서 담당하고 있으며, 실질적인 운영은 맬버른 대학교 소속 정신건강의학과 전문의를 중심으로 전문가 집단이 담당하고 있다.
- 한화 기준으로 연간 50여 억원의 예산을 집행하고 있다. 수입은 상당부분 보훈처 지원금 및 정부 정책자문, 연구 용역 수입, 교육훈련 수입 등으로 충당하고 있다.

다) 인력

- 보훈처, 방위군 및 맬버른 대를 대표하는 운영위원회 8명과 약 35명의 관리, 실무진으로 구성된다.
- 실무진은 정신건강의학과 의사를 비롯한 연구직 상당수와 소수의 행정, 회계, 전산직 직원으로 구성된다.

라) 기능

- 정책자문 및 지침 개발: 보훈처와 방위군뿐만 아니라 다양한 서비스 제공자, 정책 입안자, 지방 정부, 연구기관, 기타 유관단체에 전문적인 자문을 제공하고 또한 각종 치료 프로그램과 매뉴얼, 가이드라인을 개발, 제공한다.
- 교육 훈련: 전문가 집단을 위한 전국적인 컨퍼런스 및 워크샵, 세미나를 개최하고 있다. 각 주 정부 및 서비스 요구자의 수요에 맞추어 교육 프로그램을 제공한다. 각종 책자와 유인물, 시청각자료, 관련 웹사이트 등을 제작, 배포한다.
- 연구: ACPMH의 핵심기능으로서 직접 연구 및 연구과제 용역을 통해서 다양한 형태의 PTSD관련 연구를 수행하고 있으며 매년 수십편의 관련 논문을 발표한다.

제4장 연구결과

1. 연구참여자의 일반적 특성

가. 성별 및 연령

북한이탈주민의 일반적 특성은 성별과 출생연도로 구성되어 있으며, 각 특성별 분석 결과는 다음과 같다.

1) 성별

여성이 245명(81.67%)으로 대부분을 차지하였으며, 남성은 55명(18.33%)으로 구성되었다.

<표 4-1> 북한이탈주민의 성별 특성

요인	구분	빈도(회)	백분율(%)
성별	남	55	18.33
	여	245	81.67
	합계	300	100.00

2) 연령

북한이탈주민의 연령은 평균 52.87±15.90세로, 최고령 출생자는 1930년생, 최연소 출생자는 1998년생으로 나타났다. 평균 출생연도는 1964.13±15.90년생이었다.

1960년대 출생자가 79명(26.33%)으로 가장 많았으며 그다음으로는 1940년대 출생자(58명, 19.33%), 1970년대 출생자(58명, 19.33%) 등의 순으로 나타났다. 연령대별로 살펴보면 50대가 75명(25.00%)으로 가장 많았으며, 그다음으로 40대(68명, 22.67%), 70대(50명, 16.67%), 60대(41명, 13.67%) 등의 순으로 나타났다.

〈표 4-2〉 북한이탈주민의 출생연도 특성: 요약 통계량

요인	구분	평균	표준편차
출생연도	min=1930; max=1998	1964.13	15.90
연령	min=19.00; max=87.00	52.87	15.90

〈표 4-3〉 북한이탈주민의 출생연도 특성

요인	구분	빈도(회)	백분율(%)
출생연도	1930년대	13	4.33
	1940년대	58	19.33
	1950년대	39	13.00
	1960년대	79	26.33
	1970년대	58	19.33
	1980년대	29	9.67
	1990년대	24	8.00
	합계	300	100.00

〈표 4-4〉 북한이탈주민의 연령 특성

요인	구분	빈도(회)	백분율(%)
연령	20대 이하	30	10.00
	30대	28	9.33
	40대	68	22.67
	50대	75	25.00
	60대	41	13.67
	70대	50	16.67
	80대	8	2.67
	합계	300	100.00

나. 인구사회학적 특성 : 북한에서

북한이탈주민의 북한 생활 당시 인구사회학적 특성은 주요 거주지, 직업 및 결혼상태로 구성되어 있으며, 각 특성별 분석 결과는 다음과 같다.

1) 거주지

주요 거주지에 따라서는 함경도 거주자가 181명(60.32%)으로 가장 많았으며, 그다음으로는 양강도 거주자가 66명(22.00%), 평안도 거주자가 37명(12.34%), 황해도 거주자가 11명(3.67%), 자강도 거주자가 3명(1.00%), 강원도 거주자가 2명(0.67%) 순으로 각각 나타났다.

〈표 4-5〉 북한이탈주민의 북한생활 당시 거주지 특성

요인	구분	빈도(회)	백분율(%)
주요 거주지	함경도	181	60.32
	양강도	66	22.00
	평안도	37	12.34
	황해도	11	3.67
	자강도	3	1.00
	강원도	2	0.67
	합계	300	100.00

2) 직업

북한에서의 직업에 따라서는 ‘있음’이 258명(85.66%)으로 ‘없음’이라 응답한 64명(21.33%) 보다 많았다. ‘있음’의 응답 중에서 ‘장사’는 42명(14.00%, 300명 중; 16.28%, 직업 ‘있음’ 중)이었다.

<표 4-6> 북한이탈주민의 북한생활 당시 직업 특성

요인	구분	빈도(회)	백분율(%)
직업	있음	258	85.66
	없음	64	21.33
	합계	300	100.00

3) 결혼상태

북한에서의 결혼상태에 따라서는 ‘기혼’이 85명(28.33%)으로 가장 많았으며, 그다음으로는 ‘사별’이 83명(27.67%), ‘미혼’이 75명(25.00%), ‘이혼’이 44명(14.67%), ‘별거’가 7명(2.33%), ‘동거’가 5명(1.67%), ‘기타’가 1명(0.33%) 순으로 각각 나타났다.

<표 4-7> 북한이탈주민의 북한생활 당시 결혼상태 특성

요인	구분	빈도(회)	백분율(%)
결혼상태	기혼	85	28.33
	미혼	75	25.00
	동거	5	1.67
	이혼	44	14.67
	별거	7	2.33
	사별	83	27.67
	기타	1	0.33
	합계	300	100.00

다. 탈북 관련 특성

북한이탈주민의 탈북 관련 특성은 최초탈북년도, 하나원 퇴소년도, 제3거주국가, 중국공안체포경험, 북송경험, 교화소 또는 구치소 수감경험으로 구성되어 있으며, 각 특성별 분석 결과는 다음과 같다.

1) 최초탈북년도

최초탈북년도에 따라서는 2006~2010년대 탈북자가 79명(26.33%)으로 가장 많았으며, 그다음으로는 2011~2015년대 탈북자(77명, 25.67%), 1996~2000년대 탈북자(73명, 24.33%), 2001~2005년대 탈북자(58명, 19.33%) 등의 순으로 나타났다.

<표 4-8> 북한이탈주민의 최초탈북년도 특성

요인	구분	빈도(회)	백분율(%)
최초탈북년도	1975~1980	1	0.33
	1986~1990	1	0.33
	1991~1995	1	0.33
	1996~2000	73	24.33
	2001~2005	58	19.33
	2006~2010	79	26.33
	2011~2015	77	25.67
	2016~2017	10	3.33
	합계	300	100.00

2) 한국 국적 취득년도

한국 국적 취득년도에 따라서는 2006~2010년대 취득자가 119명(39.67%)으로 가장 많았으며, 그다음으로는 2011~2015년대 취득자(94명, 31.33%), 2016~2017년대 취득자 (48명, 16.00%), 2001~2005년대 취득자(35명, 11.67%) 등의 순으로 나타났다.

<표 4-9> 북한이탈주민의 한국 국적 취득년도 특성

요인	구분	빈도(회)	백분율(%)
한국 국적 취득년도	1991~1995	1	0.33
	1996~2000	3	1.00
	2001~2005	35	11.67
	2006~2010	119	39.67
	2011~2015	94	31.33
	2016~2017	48	16.00
	합계	300	100.00

3) 제3거주국가

탈북 후 체류하였던 제3거주국가에 따라서는 중국이 181명(60.33%)으로 가장 많았으며, 그다음으로는 제3국을 거치지 않고 남한에 바로 입국한 경우가 115명(38.34%)으로 나타났다. 러시아를 경유한 경우는 4명(1.33%)으로 나타났다.

<표 4-10> 북한이탈주민의 탈북기간 중 제3거주국가 특성

요인	구분	빈도(회)	백분율(%)
제3거주국가	직행	115	38.34
	중국	181	60.33
	러시아	4	1.33
	합계	300	100.00

4) 제3국 체류기간

탈북한 후 제3국에 체류한 경우 제3국 체류기간은 평균 6.21±4.65년으로 나타났으며 최소 0년에서 최대 19년 사이에 분포하였다.

<표 4-11> 북한이탈주민의 제3국 체류기간 특성

요인	구분	평균	표준편차
제3국 체류기간(년)	min=0.00; max=19.00	6.21	4.65

5) 중국공안체포경험

남한 입국 전 중국에 체류한 탈북민 중 공안체포경험은 ‘없음’이 105명(58.01%)으로 나타났으며, 나머지 약 40%는 최소 1회 이상 공안에 체포된 경험이 있는 것으로 나타났다. 체포횟수는 1회 체포된 경우가 41명(22.65%), 2회 체포된 경우가 19명(10.50%), 3회 체포된 경우가 7명(3.87%) 등으로 나타났다.

<표 4-12> 북한이탈주민의 탈북기간 중 중국공안체포경험 특성

요인	구분	빈도(회)	백분율(%)
중국공안체포경험(회)	없음	105	58.01
	1	41	22.65
	2	19	10.50
	3	7	3.87
	4	5	2.76
	5	3	1.66
	7	1	0.55
	합계(중국 경유자)	181	100.00

6) 북송경험

남한 입국 전 중국에 체류한 탈북민 중 북송경험 여부는 ‘없음’이 122명(67.40%)으로 나타났으며, 나머지 약 33%는 북송을 경험한 것으로 나타났다. 북송경험 횟수는 1회 경험자가 36명(19.89%), 2회 경험자가 13명(7.18%) 등으로 나타났다.

<표 4-13> 북한이탈주민의 탈북기간 중 북송경험 특성

요인	구분	빈도(회)	백분율(%)
북송경험(회)	없음	122	67.40
	1	36	19.89
	2	13	7.18
	3	3	1.66
	4	3	1.66

요인	구분	빈도(회)	백분율(%)
	5	3	1.66
	7	1	0.55
	합계(중국 경유자)	181	100.00

7) 교화소 및 구치소 수감경험

남한 입국 전 중국에 체류한 탈북민 중 교화소 및 구치소 수감경험 여부는 '없음'이 119명(65.75%)으로 나타났으며, 나머지 약 35%는 교화소 및 구치소 수감을 경험한 것으로 나타났다. 그 횟수를 살펴보면 1회 경험자가 43명(23.76%), 2회 경험자가 10명(5.52%) 등으로 나타났다.

〈표 4-14〉 북한이탈주민의 탈북기간 중 교화소 및 구치소 수감경험 특성

요인	구분	빈도(회)	백분율(%)
교화소 및 구치소 수감경험(회)	없음	119	65.75
	1	43	23.76
	2	10	5.52
	3	4	2.21
	4	1	0.55
	5	3	1.66
	7	1	0.55
	합계(중국 경유자)	181	100.00

라. 인구사회학적 특성 : 남한에서

북한이탈주민의 남한 입국 후 인구사회학적 특성은 취업경험, 남한결혼상태, 북한잔류가족, 진단받은 질병 및 문화적응 스트레스로 구성되어 있으며, 각 특성별 분석 결과는 다음과 같다.

1) 취업경험

북한이탈주민의 남한 입국 후 취업 경험에 따라서는 '예'가 147명(49.00%)이었으며, '아니오'는 153명(51.00%)으로 나타났다.

〈표 4-15〉 북한이탈주민의 남한에서 취업한 경험 특성

요인	구분	빈도(회)	백분율(%)
취업경험	예	147	49.00
	아니오	153	51.00
	합계	300	100.00

남한 입국 후 취업 경험이 있었던 북한이탈주민의 취업 유형을 구체적으로 살펴보면 식당 또는 공장 등에서 근로하는 노동자가 108명(73.47%)으로 가장 많았으며, 그다음으로는 경리 또는 회사원과 같은 사무원이 29명(19.73%), 교사 또는 회계사와 같은 전문직이 4명(2.72%), 농업 또는 버섯 채취 등에 종사하는 농장원이 2명(1.36%), 예술단과 같은 예술 · 체육 직군이 1명(0.68%)으로 각각 나타났다.

〈표 4-16〉 북한이탈주민의 남한에서 취업한 유형 특성

요인	구분	빈도(회)	백분율(%)
취업경험	노동자	108	73.47
	농장원	2	1.36
	사무원	29	19.73
	전문직	4	2.72
	예술 · 체육	1	0.68
	결측	3	1.00
	합계(취업경험 있음)	147	100.00

2) 현재 취업 지속여부

남한 입국 후 취업 경험이 있었던 북한이탈주민의 현재 취업 지속여부에 따라서는 ‘예’가 36명(24.49%)이었으며, ‘아니오’는 111명(75.51%)으로 나타났다.

<표 4-17> 북한이탈주민의 남한에서 취업 후 지속여부 특성

<table>
<tr><th>요인</th><th>구분</th><th>빈도(회)</th><th>백분율(%)</th></tr>
<tr><td rowspan="3">취업경험</td><td>예</td><td>36</td><td>24.49</td></tr>
<tr><td>아니오</td><td>111</td><td>75.51</td></tr>
<tr><td>합계(취업경험 있음)</td><td>147</td><td>100.00</td></tr>
</table>

3) 직장을 그만둔 이유

남한 입국 후 취업 경험이 있었던 북한이탈주민이 취업하였던 직장을 그만둔 이유에 응답한 111명의 응답을 구체적으로 살펴보면 건강상의 이유가 69명(60.53%)으로 가장 많았으며, 그다음으로는 스트레스와 부적응이 17명(14.91%), 정년 혹은 퇴직이 7명(6.14%), 결혼, 육아, 간병 등의 이유가 6명(5.26%), 실업 혹은 구직 중인 경우가 6명(5.26%), 기타 그 외의 사유가 6명(5.26%), 학교/학원에 재학/재원 중인 경우가 3명(2.63%) 순으로 각각 나타났다.

<표 4-18> 북한이탈주민의 남한에서 취업한 직장을 그만둔 이유 특성

<table>
<tr><th>요인</th><th>구분</th><th>빈도(회)</th><th>백분율(%)</th></tr>
<tr><td rowspan="8">직장을 그만둔 이유</td><td>건강상의 이유</td><td>69</td><td>60.53</td></tr>
<tr><td>결혼, 육아, 간병 등의 이유</td><td>6</td><td>5.26</td></tr>
<tr><td>정년 혹은 퇴직 상태임</td><td>7</td><td>6.14</td></tr>
<tr><td>스트레스와 부적응</td><td>17</td><td>14.91</td></tr>
<tr><td>실업 혹은 구직 중임</td><td>6</td><td>5.26</td></tr>
<tr><td>학교/학원에 재학/재원 중임</td><td>3</td><td>2.63</td></tr>
<tr><td>기타</td><td>6</td><td>5.26</td></tr>
<tr><td>전체 응답 수(중복응답허용)</td><td>147건(111명 중)</td><td>100.00</td></tr>
</table>

4) 남한에서 혼인 관련 경험

북한이탈주민의 남한 입국 후 혼인 관련 경험에 대해서는 북한에서 결혼 상태 유무와 관련 없이 남한에서의 경험 위주로 조사 분석하였다. 그 결과'미혼'이 188명(62.67%)으로 가장 많았으며, 그다음으로는 '결혼'이 61명(20.33%), '이혼'이 25명(8.33%), '동거'가 14명(4.67%), '사별'이 6명(2.00%), '기타'가 3명(1.00%), '별거'가 2명(0.67%) 순으로 각각 나타났다.

<표 4-19> 북한이탈주민의 남한에서 혼인 관련 상태 특성

요인	구분	빈도(회)	백분율(%)
남한에서 혼인 관련 상태*	미혼**	188	62.67
	기혼	61	20.33
	이혼	25	8.33
	동거	14	4.67
	사별	6	2.00
	기타	3	1.00
	별거	2	0.67
	결측	1	0.33
	합계	300	100.00

*남한에서의 혼인 관련 상태만을 조사하였음.

**북한에서 기혼자라 할지라도 남한에서 결혼한 상태가 아니면 미혼으로 응답한 결과임.

5) 배우자 출신

배우자 출신에 따라서는 ‘탈북민’이 65명(58.04%)으로 가장 많았으며, 그다음으로는 ‘조선족’이 17명(15.18%), ‘남한’이 14명(12.50%), ‘한족’이 5명(4.46%), ‘기타’가 4명(3.57%) 순으로 각각 나타났다.

<표 4-20> 북한이탈주민의 남한에서 결혼한 배우자 출신 특성

요인	구분	빈도(회)	백분율(%)
배우자출신	탈북민	65	58.04
	조선족	17	15.18
	한족	5	4.46
	남한	14	12.50
	기타	4	3.57
	결측	7	6.25
	합계(미혼 제외)	112	100.00

6) 북한잔류가족

북한이탈주민의 북한잔류가족 여부에 따라서는 ‘예’가 251명(83.67%)으로 더 많았으며, ‘아니오’는 49명(16.33%)으로 나타났다.

<표 4-21> 북한이탈주민의 북한잔류가족여부 특성

요인	구분	빈도(회)	백분율(%)
북한잔류가족여부	예	251	83.67
	아니오	49	16.33
	합계	300	100.00

7) 북한잔류가족과의 관계

북한이탈주민의 북한잔류가족과의 관계에 응답한 251명의 응답을 구체적으로 살펴보면 ‘형제’가 154명(35.65%)로 가장 많았으며, 그다음으로는 ‘자녀’가 133명(30.79%), ‘부모’가 101명(23.38%), ‘기타’가 58명(13.43%), ‘손자녀’가 7명(1.62%)의 순으로 나타났다.

<표 4-22> 북한이탈주민의 북한잔류가족과의 관계 특성

요인	구분	빈도(회)	백분율(%)
북한잔류가족과의 관계	형제	154	35.65
	자녀	133	30.79
	손자녀	7	1.62
	부모	101	23.38
	기타	37	13.43
	전체 응답 수(중복응답허용)	453건(251명 중)	100.00

8) 북한잔류가족 수

<표 4-23> 북한이탈주민의 북한잔류가족 수 특성

요인	구분	빈도(회)	백분율(%)
북한잔류가족(명)	1	52	20.72
	2	71	28.29
	3	53	21.12
	4	31	12.35
	5	21	8.37
	6	7	2.79
	7	3	1.20
	8	2	0.80
	9	2	0.80
	10	7	2.79
	15	2	0.80
	합계(북한잔류가족: 있음)	251	100.00

북한에 잔류가족이 있다고 응답한 북한이탈주민의 북한잔류가족 수에 따라서는 ‘2명 잔류’가 71명(28.29%)으로 가장 많았으며, 그다음으로는 ‘3명 잔류’가 53명(21.12%), ‘1명 잔류’가 52명(20.72%) 등의 순으로 각각 나타났다

9) 질병진단 당시 치료 여부

남한에서 진단받은 질병이 있는 북한이탈주민의 질병 진단당시 치료 여부에 따라서는 ‘예’가 224명(95.73%)으로 나타나 대다수를 차지하였으며, ‘아니오’는 10명(4.27%)으로 나타났다.

<표 4-24> 북한이탈주민의 남한에서 진단받은 질병 진단 당치 치료 여부 특성

요인	구분	빈도(회)	백분율(%)
진단당시치료 여부	예	224	95.73
	아니오	10	4.27
	합계(‘진단받은 질병이 있음’ 중)	234	100.00

10) 진단받은 질병의 치료지속 여부

남한에서 진단받은 질병이 있는 북한이탈주민의 진단받은 질병의 치료지속 여부에 따라서는 ‘예’가 203명(86.75%)으로 나타나 대다수를 차지하였으며, ‘아니오’는 31명(13.25%)으로 나타났다.

<표 4-25> 북한이탈주민의 남한에서 진단받은 질병의 치료지속여부 특성

요인	구분	빈도(회)	백분율(%)
진단받은 질병의 치료지속 여부	예	203	86.75
	아니오	31	13.25
	합계(‘진단받은 질병이 있음’ 중)	234	100.00

11) 진단받은 질병의 치료를 지속하지 못한 이유

남한에서 진단받은 질병이 있는 북한이탈주민이 진단받은 질병 치료를 지속하지 못한 이유에 따라서는 ‘증상이 가벼워서’가 17명(54.84%)으로 가장 많았으며, 그다음으로는 ‘내가 갈 수 있는 시간에 병의원 등이 문을 열지 않아서’가 3명(9.68%), ‘경제적인 이유로’가 2명(6.45%), ‘기타’가 9명(29.03%)의 순으로 각각 나타났다. ‘기타’ 응답 중 주요 내용을 살펴보면, ‘치료가 불가능한 질환이어서’, ‘효과가 별로 없어서’, ‘자의로 치료 중단’ 등이 있었다.

〈표 4-26〉 북한이탈주민의 남한에서 진단받은 질병의 치료를 지속하지 못한 이유

요인	구분	빈도(회)	백분율(%)
진단받은 질병의 치료를 지속하지 못한 이유	증상이 가벼워서	17	54.84
	내가 갈 수 있는 시간에 병의원 등이 문을 열지 않아서	3	9.68
	경제적인 이유로	2	6.45
	기타	9	29.03
	합계(‘치료를 지속하지 못함’ 중)	31	100.00

12) 문화적응 스트레스

문화적응 스트레스는 객관형 응답 4문항과 그렇게 응답한 구체적인 사유 및 주관식 응답 1문항으로 구성되어 있다.

문화적응 스트레스 1번 문항(남한 언어 이해 어려움)에 따라서는 ‘조금어려움’이 147명(49.00%)으로 가장 많았으며, 그다음으로는 ‘매우어려움’이 76명(25.33%), ‘전혀없음’이 73명(24.33%) 순으로 나타났다.

문화적응 스트레스 2번 문항(남한 사람들과 어울리는데 어려움)에 따라서는 ‘조금어려움’이 127명(42.33%)으로 가장 많았으며, 그다음으로는 ‘전혀없음’이 83명(27.67%), ‘매우어려움’이 83명(27.67%) 순으로 나타났다.

문화적응 스트레스 3번 문항(남한 사람들에게 무시당하는 느낌, 경험)에 따라서는 ‘조금어려움’이 117명(39.00%)으로 가장 많았으며, 그다음으로는 ‘전혀없음’이 115명(38.33%), ‘매우어려움’이 63명(21.00%) 순으로 나타났다.

문화적응 스트레스 4번 문항(이웃과 어울리기, 도움 주고받기 어려움)에 따라서는 ‘전혀없음’이 125명(41.67%)으로 가장 많았으며, 그다음으로는 ‘조금어려움’이 96명(32.00%), ‘매우어려움’이 72명(24.00%) 순으로 각각 나타났다.

문화적응 스트레스 5번 문항(그 외 남한에서 살면서 힘든 점)에 따라서는 ‘경제적 문제’가 22명(7.33%)으로 가장 많았으며, 그다음으로는 ‘편견’이 10명(3.33%), ‘외로움’이 7명(2.33%), ‘건강’이 7명(2.33%), ‘학업’이 6명(2.00%), ‘언어’가 6명(2.00%), ‘자녀양육’이 3명(1.00%) 순으로 각각 나타났다.

〈표 4-27〉 북한이탈주민의 남한에서 겪은 문화적응 스트레스 특성

요인	구분	빈도(회)	백분율(%)
1. 남한 언어 이해 어려움	매우어려움	76	25.33
	조금어려움	147	49.00
	전혀 없음	73	24.33
	결측	4	1.33
2. 남한 사람들과 어울리는데 어려움	매우어려움	83	27.67
	조금어려움	127	42.33
	전혀 없음	83	27.67
	결측	7	2.34
3. 남한 사람들에게 무시당하는 느낌, 경험	매우어려움	63	21.00
	조금어려움	117	39.00
	전혀 없음	115	38.33
	결측	5	1.67
4. 이웃과 어울리기, 도움 주고받기 어려움	매우어려움	72	24.00
	조금어려움	96	32.00
	전혀 없음	125	41.67
	결측	7	2.34
5. 그 외 남한에서 살면서 힘든 점	경제적 문제	22	7.33
	편견	10	3.33
	외로움	7	2.33
	건강	7	2.33
	학업	6	2.00
	언어(말하기)	6	2.00
	자녀양육	3	1.00
	결측	239	79.67
합계		300	100.00

2. 인권침해 트라우마 경험 실태

가. 인권침해 경험

북한이탈주민의 인권침해에 대한 본인 또는 가족의 겪음, 주변 사람들이 겪는 것을 목격 하였거나, 소문을 들은 것에 대한 경험 여부를 평가하기 위하여 인권침해 조사표를 이용하였다. 점검문항은 총 13개의 다양한 인권침해 사건으로 구성되어 있으며, 각 사건 특성별 필요에 따라 북한 생활 당시와 탈북과정 중 및 남한 입국 후의 세 시점에서 각각 회상 평가하였다.

'전혀 겪거나 들은 바 없다'에 0점 ~ '내가 직접 당하였다'를 4점을 순서대로 부여하여 개인별로 합산한 인권침해 조사표 점수는 평균 29.78±7.40점이었으며 0~53점 사이에 분포하였다.

〈표 4-28〉 북한이탈주민이 경험한 인권침해 경험 특성: 요약 통계량

요인	구분	평균	표준편차
인권침해 조사표 점수	min=0; max=53	29.78	7.40

북한이탈주민이 경험한 인권침해 사건을 다빈도순으로 살펴보면 공개적인 자아비판을 직접 경험한 경우가 247명(82.33%)으로 가장 많았으며, 그다음으로는 북한에서 심한 굶주림 또는 질병을 직접 경험한 경우가 188명(62.67%), 북한에서 이웃과 당원의 감시와 고발을 직접 경험한 경우가 171명(57.00%), 북한에서 통신검열 또는 록화기 단속을 직접 경험한 경우가 167명(55.67%), 북한에서 토대가 나쁘다는 이유로 인한 차별을 직접 경험한 경우가 120명(40.00%), 북한에서 국가기관 또는 군대에서 매질을 직접 경험한 경우가 65명(21.67%), 북한에서 탈북으로 인한 처벌 또는 강제송환을 직접 경험한 경우가 65명(21.67%), 탈북기간 중 여성 또는 아동 인신매매를 직접 경험한 경우가 65명(21.67%), 북한에서 잘못 없이 국가기관에 끌려가는 것을 직접 경험한 경우가 62명(20.67%), 북한에서 가족들이 굶주림으로 인한 죽음을 경험한 경우가 31명(10.33%), 북한에서 여성에 대한 성폭력을 직접 경험한 경우가 22명(7.33%), 탈북기간 중 여성에 대한 성폭력을 직접 경험한 경우가 20명(6.67%), 탈북기간 중 심한 굶주림 또는 질병을 직접 경험한 경우가 10명(3.33%), 북한에서 정치범수용소의 잔혹성을 직접 경험한 경우가 10명(3.33%), 북한에서 가족들이 공개

처형을 당하는 것을 경험한 경우가 3명(1.00%), 남한에서 심한 굶주림 또는 질병을 직접 경험한 경우가 2명(0.67%) 순으로 각각 나타났다. 남한에서 여성에 대한 성폭력에 대한 소문을 들은 경우는 18명(6.00%)으로 나타났다.

<표 4-29> 북한이탈주민이 경험한 인권침해 경험 특성

요인	구분	빈도(회)	백분율(%)
심한 굶주림 또는 질병: 북한에서	내가 직접 당하였다	188	62.67
	가족들이 당하는 것을 경험하였다	12	4.00
	주변사람들이 당하는 것을 목격하였다	68	22.67
	소문을 들었다	17	5.67
	전혀 겪거나 들은 바 없다	15	5.00
심한 굶주림 또는 질병: 탈북기간 중	내가 직접 당하였다	10	3.33
	소문을 들었다	3	1.00
	전혀 겪거나 들은 바 없다	287	95.67
심한 굶주림 또는 질병: 남한에서	내가 직접 당하였다	2	0.67
	소문을 들었다	3	1.00
	전혀 겪거나 들은 바 없다	295	98.33
정치범수용소의 잔혹성: 북한에서	내가 직접 당하였다	10	3.33
	가족들이 당하는 것을 경험하였다	23	7.67
	주변사람들이 당하는 것을 목격하였다	46	15.33
	소문을 들었다	197	65.67
	전혀 겪거나 들은 바 없다	24	8.00
공개처형: 북한에서	가족들이 당하는 것을 경험하였다	3	1.00
	주변사람들이 당하는 것을 목격하였다	243	81.00
	소문을 들었다	44	14.67
	전혀 겪거나 들은 바 없다	10	3.33
국가기관/군대에서 매질: 북한에서	내가 직접 당하였다	65	21.67
	가족들이 당하는 것을 경험하였다	28	9.33
	주변사람들이 당하는 것을 목격하였다	50	16.67
	소문을 들었다	108	36.00
	전혀 겪거나 들은 바 없다	49	16.33
잘못없이 국가기관에 끌려감: 북한에서	내가 직접 당하였다	62	20.67
	가족들이 당하는 것을 경험하였다	34	11.33
	주변사람들이 당하는 것을 목격하였다	52	17.33
	소문을 들었다	88	29.33
	전혀 겪거나 들은 바 없다	64	21.33

요인	구분	빈도(회)	백분율(%)
이웃과 당원의 감시와 고발: 북한에서	내가 직접 당하였다	171	57.00
	가족들이 당하는 것을 경험하였다	15	5.00
	주변사람들이 당하는 것을 목격하였다	38	12.67
	소문을 들었다	48	16.00
	전혀 겪거나 들은 바 없다	28	9.33
토대가 나쁘다는 이유로 인한 차별: 북한에서	내가 직접 당하였다	120	40.00
	가족들이 당하는 것을 경험하였다	21	7.00
	주변사람들이 당하는 것을 목격하였다	78	26.00
	소문을 들었다	48	16.00
	전혀 겪거나 들은 바 없다	33	11.00
공개적인 자아비판: 북한에서	내가 직접 당하였다	247	82.33
	가족들이 당하는 것을 경험하였다	3	1.00
	주변사람들이 당하는 것을 목격하였다	23	7.67
	소문을 들었다	17	5.67
	전혀 겪거나 들은 바 없다	10	3.33
통신검열 또는 록화기 단속: 북한에서	내가 직접 당하였다	167	55.67
	가족들이 당하는 것을 경험하였다	10	3.33
	주변사람들이 당하는 것을 목격하였다	30	10.00
	소문을 들었다	35	11.67
	전혀 겪거나 들은 바 없다	58	19.33
굶주림으로 인한 죽음: 북한에서	가족들이 당하는 것을 경험하였다	31	10.33
	주변사람들이 당하는 것을 목격하였다	207	69.00
	소문을 들었다	48	16.00
	전혀 겪거나 들은 바 없다	14	4.67
탈북으로 인한 처벌(강제송환): 북한 또는 탈북기간 중	내가 직접 당하였다	65	21.67
	가족들이 당하는 것을 경험하였다	14	4.67
	주변사람들이 당하는 것을 목격하였다	85	28.33
	소문을 들었다	120	40.00
	전혀 겪거나 들은 바 없다	16	5.33
여성에 대한 성폭력: 북한에서	내가 직접 당하였다	22	7.33(여성의 9.0)
	가족들이 당하는 것을 경험하였다	2	0.67
	주변사람들이 당하는 것을 목격하였다	30	10.00
	소문을 들었다	105	35.00
	전혀 겪거나 들은 바 없다	141	47.00
여성에 대한 성폭력: 탈북기간 중	내가 직접 당하였다	20	6.67(여성의 8.16)
	가족들이 당하는 것을 경험하였다	1	0.33
	주변사람들이 당하는 것을 목격하였다	4	1.33
	소문을 들었다	36	12.00
	전혀 겪거나 들은 바 없다	239	79.66

요인	구분	빈도(회)	백분율(%)
여성에 대한 성폭력: 남한에서	소문을 들었다	18	6.00
	전혀 겪거나 들은 바 없다	282	94.00
여성 또는 아동 인신매매: 탈북기간 중	내가 직접 당하였다	65	21.67(여성의 26.53)
	가족들이 당하는 것을 경험하였다	7	2.33
	주변사람들이 당하는 것을 목격하였다	58	19.33
	소문을 들었다	134	44.67
	전혀 겪거나 들은 바 없다	36	12.00
합계		300	100.00

* 복수의 경험이 있는 경우, 가장 강도가 높은 응답을 선택하도록 함.

나. 트라우마 경험

북한이탈주민의 스트레스 사건별 경험 또는 목격 여부를 평가하기 위하여 생활사건점검표 (Life Events Checklist for DSM-5, LEC-5)를 이용하였다. 점검문항은 자연재난 및 화재 또는 폭발, 교통사고, 직장이나 집 또는 여가 활동 중 심각한 사고, 독성 물질에 노출, 신체폭력, 무기로 공격당함, 성폭력, 기타 원하지 않거나 불편한 성적 경험, 전투나 전쟁터에 노출, 감금, 목숨이 좌우될 정도의 질병이나 부상, 심각한 인간적 고난, 급작스러운 변사, 급작스러운 사고사, 나 자신 때문에 발생했던 다른 사람의 심각한 부상, 상해 또는 사망, 그 밖의 매우 심각한 스트레스 사건이나 경험과 같은 총 17개의 다양한 스트레스 사건으로 구성되어 있으며, 북한 생활 당시와 탈북과정 중 및 남한 입국 후의 세 시점에서 각각 회상 평가하였다. 각 특성별 분석 결과는 다음과 같다.

1) 북한에서

<표 4-30> 북한이탈주민의 북한 생활 당시 경험한 스트레스 사건: 요약 통계량

요인	구분	평균	표준편차
스트레스 사건 경험 수	min=0; max=11	3.45	2.34

북한이탈주민이 북한 거주 당시 경험 또는 목격한 스트레스 사건들은 평균 3.45±2.34개였으며, 0~11개 사이에 분포하였다. 이를 각 스트레스 사건 유형별로 살펴본 결과는 다음과 같다. 강제적인 노동이나 지속적인 굶주림 또는 식량 부족, 지

속적인 노숙 상태, 고문과 같은 심각한 인간적 고난을 직접 겪은 경우가 192명(64.00%)에 달해 가장 많았고, 타인의 겪음을 목격한 경우는 53명(17.67%)으로 나타났다.

그다음으로는 공격, 가격(손, 주먹, 몽둥이로 공격), 따귀를 맞음, 또는 발로 채이거나 두들겨 맞는 등의 신체폭력을 직접 겪은 경우가 139명(46.33%)으로 나타났고, 타인의 겪음을 목격한 경우는 50명(16.67%)으로 나타났다.

홍수나 태풍, 폭풍, 지진과 같은 자연재난을 직접 겪은 경우는 133명(44.33%)이었으며, 타인의 겪음을 목격한 경우는 76명(25.33%)으로 나타났다.

타인이 살인이나 자살 등의 급작스러운 변사를 당하는 것을 목격한 경우는 105명(35.00%)이었으며, 급작스러운 사고사를 목격한 경우는 99명(33.00%)으로 나타났다.

목숨이 좌우될 정도의 질병이나 부상을 직접 겪은 경우는 75명(25.00%)이었으며, 타인의 겪음을 목격한 경우는 26명(8.67%)으로 나타났다.

<표 4-31> 북한이탈주민의 북한 생활 당시 경험한 스트레스 사건

요인	구분	빈도(회)	백분율(%)
1. 자연재난	직접겪음	133	44.33
	타인겪음목격	76	25.33
	경험 안함	91	30.33
2. 화재 또는 폭발	직접겪음	40	13.33
	타인겪음목격	95	31.67
	경험 안함	165	55.00
3. 교통사고	직접겪음	36	12.00
	타인겪음목격	78	26.00
	경험 안함	186	62.00
4. 직장, 집, 또는 여가 활동 중 심각한 사고	직접겪음	18	6.00
	타인겪음목격	13	4.33
	경험 안함	269	89.67
5. 독성 물질에 노출	직접겪음	9	3.00
	타인겪음목격	6	2.00
	경험 안함	285	95.00
6. 신체폭력	직접겪음	139	46.33
	타인겪음목격	50	16.67
	경험 안함	111	37.00
7. 무기로 공격당함	직접겪음	37	12.33
	타인겪음목격	36	12.00
	경험 안함	227	75.67

요인	구분	빈도(회)	백분율(%)
8. 성폭력	직접겪음	17	5.67
	타인겪음목격	20	6.67
	경험 안함	263	87.67
9. 기타 원하지 않거나 불편한 성적 경험	직접겪음	16	5.33
	타인겪음목격	8	2.67
	경험 안함	276	92.00
10. 전투나 전쟁터에 노출	직접겪음	12	4.00
	타인겪음목격	7	2.33
	경험 안함	281	93.67
11. 감금	직접겪음	72	24.00
	타인겪음목격	24	8.00
	경험 안함	204	68.00
12. 목숨이 좌우될 정도의 질병이나 부상	직접겪음	75	25.00
	타인겪음목격	26	8.67
	경험 안함	199	66.33
13. 심각한 인간적 고난	직접겪음	192	64.00
	타인겪음목격	53	17.67
	경험 안함	55	18.33
14. 급작스러운 변사	타인겪음목격	105	35.00
	경험 안함	195	65.00
15. 급작스러운 사고사	타인겪음목격	99	33.00
	경험 안함	201	67.00
16. 나 자신 때문에 발생했던 다른 사람의 심각한 부상, 상해 또는 사망	직접겪음	2	0.67
	타인겪음목격	3	1.00
	경험 안함	295	98.33
17. 그 밖의 매우 심각한 스트레스 사건이나 경험	직접겪음	33	11.00
	타인겪음목격	6	2.00
	경험 안함	261	87.00
합계		300	100.00

* 복수의 경험이 있는 경우, 가장 강도가 높은 응답을 선택하도록 함.

납치, 유괴, 인질 및 전쟁 포로 등으로 인해 감금된 경험에 따라서는 직접 겪은 경우가 72명(24.00%)이었으며, 타인의 겪음을 목격한 경우는 24명(8.00%)으로 나타났다.

총이나 칼에 맞거나, 칼, 총, 폭탄으로 위협 당하는 등과 같이 무기에 의한 공격을 직접 겪은 경우는 37명(12.33%)이었으며, 타인의 겪음을 목격한 경우는 36명(12.00%)으로 나타났다.

자동차 사고, 선박 사고, 기차 사고 및 비행기 추락과 같은 교통사고를 직접

겪은 경우는 36명(12.00%)이었으며, 타인의 겪음을 목격한 경우는 78명(26.00%)으로 나타났다.

직장이나 집 또는 여가 활동 중 심각한 사고를 직접 겪은 경우는 18명(6.00%)이었으며, 타인의 겪음을 목격한 경우는 13명(4.33%)으로 나타났다.

성폭행 시도 또는 완력이나 위협 하에 성적 행위를 직접 겪은 경우는 17명(5.67%)이었으며, 타인의 겪음을 목격한 경우는 20명(6.67%)으로 나타났다.

기타 원하지 않거나 불편한 성적 경험을 직접 겪은 경우는 16명(5.33%)이었으며, 타인의 겪음을 목격한 경우는 8명(2.67%)으로 나타났다.

전투나 전쟁터에 노출되는 상황을 직접 겪은 경우는 12명(4.00%)이었으며, 타인의 겪음을 목격한 경우는 7명(2.33%)으로 나타났다.

독성 물질에 노출되는 상황을 직접 겪은 경우는 9명(3.00%)이었으며, 타인의 겪음을 목격한 경우는 6명(2.00%)으로 나타났다.

나 자신 때문에 발생했던 다른 사람의 심각한 부상, 상해 또는 사망을 직접 겪은 경우는 2명(0.67%)이었으며, 타인의 겪음을 목격한 경우는 3명(1.00%)으로 나타났다.

제시된 16유형 외의 매우 심각한 스트레스 사건을 직접 경험한 경우는 33명(11.00%)으로 나타났으며, 타인의 겪음을 목격한 경우는 6명(2.00%)으로 나타났다.

2) 탈북 기간 중

제3국에 거주하다가 남한에 입국한 북한이탈주민이 탈북 기간 동안 경험 또는 목격한 스트레스 사건들은 평균 0.25±0.72개였으며, 0~6개 사이에 분포하였다. 이를 각 스트레스 사건 유형별로 살펴본 결과는 다음과 같다.

〈표 4-32〉 북한이탈주민의 제3국 거주 탈북 기간 중 경험한 스트레스 사건: 요약 통계량

요인	구분	평균	표준편차
스트레스 사건 경험 수	min=0; max=6	0.25	0.72

납치, 유괴, 인질 및 전쟁 포로 등으로 인해 감금된 경험에 따라서는 직접 겪은 경우가 51명(27.57%)에 달해 가장 많았으며, 타인의 겪음을 목격한 경우는 3명(1.62%)으로 나타났다.

그다음으로는 공격, 가격(손, 주먹, 몽둥이로 공격), 따귀를 맞음, 또는 발로

채이거나 두들겨 맞는 등의 신체폭력을 직접 겪은 경우가 49명(26.49%)으로 나타났고, 타인의 겪음을 목격한 경우는 6명(3.24%)으로 나타났다.

성폭행 시도 또는 완력이나 위협 하에 성적 행위를 직접 겪은 경우는 23명(12.43%)이었으며, 타인의 겪음을 목격한 경우는 4명(2.16%)으로 나타났다.

기타 원하지 않거나 불편한 성적 경험을 직접 겪은 경우는 24명(12.97%)이었으며, 타인의 겪음을 목격한 경우는 4명(2.16%)으로 나타났다.

총이나 칼에 맞거나, 칼, 총, 폭탄으로 위협 당하는 등과 같이 무기에 의한 공격을 직접 겪은 경우는 21명(11.35%)이었으며, 타인의 겪음을 목격한 경우는 3명(1.62%)으로 나타났다.

강제적인 노동이나 지속적인 굶주림 또는 식량 부족, 지속적인 노숙 상태, 고문과 같은 심각한 인간적 고난을 직접 겪은 경우는 16명(8.65%)명이었으며, 타인의 겪음을 목격한 경우는 1명(0.54%)으로 나타났다.

목숨이 좌우될 정도의 질병이나 부상을 직접 겪은 경우는 12명(6.49%)이었으며, 타인의 겪음을 목격한 경우는 2명(1.08%)으로 나타났다.

홍수나 태풍, 폭풍, 지진과 같은 자연재난을 직접 겪은 경우는 8명(4.32%)이었으며, 타인의 겪음을 목격한 경우는 3명(1.62%)으로 나타났다.

직장이나 집 또는 여가 활동 중 심각한 사고를 직접 겪은 경우는 6명(3.24%)이었으며, 타인의 겪음을 목격한 경우는 2명(1.08%)으로 나타났다.

자동차 사고, 선박 사고, 기차 사고 및 비행기 추락과 같은 교통사고를 직접 겪은 경우는 5명(2.70%)이었으며, 타인의 겪음을 목격한 경우는 14명(7.57%)으로 나타났다.

<표 4-33> 북한이탈주민의 제3국 거주한 탈북 기간 중 경험한 스트레스 사건

요인	구분	빈도(회)	백분율(%)
1. 자연재난	직접겪음	8	4.32
	타인겪음목격	3	1.62
	경험 안함	174	94.05
2. 화재 또는 폭발	직접겪음	2	1.08
	타인겪음목격	3	1.62
	경험 안함	180	97.3
3. 교통사고	직접겪음	5	2.70
	타인겪음목격	14	7.57
	경험 안함	166	89.73
4. 직장, 집, 또는 여가 활동 중 심각한 사고	직접겪음	6	3.24
	타인겪음목격	2	1.08
	경험 안함	177	95.68

요인	구분	빈도(회)	백분율(%)
5. 독성 물질에 노출	타인겪음목격	1	0.54
	경험 안함	184	99.46
6. 신체폭력	직접겪음	49	26.49
	타인겪음목격	6	3.24
	경험 안함	130	70.27
7. 무기로 공격당함	직접겪음	21	11.35
	타인겪음목격	3	1.62
	경험 안함	161	87.03
8. 성폭력	직접겪음	23	12.43
	타인겪음목격	4	2.16
	경험 안함	158	85.41
9. 기타 원하지 않거나 불편한 성적 경험	직접겪음	24	12.97
	타인겪음목격	4	2.16
	경험 안함	157	84.86
10. 전투나 전쟁터에 노출	경험 안함	185	100.00
11. 감금	직접겪음	51	27.57
	타인겪음목격	3	1.62
	경험 안함	131	70.81
12. 목숨이 좌우될 정도의 질병이나 부상	직접겪음	12	6.49
	타인겪음목격	2	1.08
	경험 안함	171	92.43
13. 심각한 인간적 고난	직접겪음	16	8.65
	타인겪음목격	1	0.54
	경험 안함	168	90.81
14. 급작스러운 변사	타인겪음목격	4	2.16
	경험 안함	181	97.84
15. 급작스러운 사고사	타인겪음목격	7	3.78
	경험 안함	178	96.22
16. 나 자신 때문에 발생했던 다른 사람의 심각한 부상, 상해 또는 사망	직접겪음	1	0.54
	경험 안함	184	99.46
17. 그 밖의 매우 심각한 스트레스 사건이나 경험	직접겪음	21	11.35
	경험 안함	164	88.65
	합계	185	100.00

화재 또는 폭발을 직접 겪은 경우는 2명(1.08%)이었으며, 타인의 겪음을 목격한 경우는 3명(1.62%)으로 나타났다.

타인이 독성 물질에 노출되는 상황을 겪음을 목격한 경우는 1명(0.54%)으로 나타났다.

제시된 16유형 외의 매우 심각한 스트레스 사건을 직접 경험한 경우는 21명(11.35%)으로 나타났다.

전투나 전쟁터에 노출되는 상황을 직접 또는 간접적으로 겪은 경우는 보고되지 않았다.

3) 남한에서

북한이탈주민이 남한에서 경험 또는 목격한 스트레스 사건들은 평균 0.14±0.46개였으며, 0~3개 사이에 분포하였다. 이를 각 스트레스 사건 유형별로 살펴본 결과는 다음과 같다.

〈표 4-34〉 북한이탈주민의 남한에서 경험한 스트레스 사건: 요약 통계량

요인	구분	평균	표준편차
스트레스 사건 경험 수	min=0; max=3	0.14	0.46

제시된 16유형 외의 매우 심각한 스트레스 사건을 직접 경험한 경우는 37명(12.33%)으로 가장 많았으며, 타인의 겪음을 목격한 경우는 3명(1.00%)으로 나타났다.

그다음으로는 자동차 사고, 선박 사고, 기차 사고 및 비행기 추락과 같은 교통사고를 직접 겪은 경우는 31명(10.33%)이었으며, 타인의 겪음을 목격한 경우는 15명(5.00%)으로 나타났다.

목숨이 좌우될 정도의 질병이나 부상을 직접 겪은 경우는 13명(4.33%)이었으며, 타인의 겪음을 목격한 경우는 2명(0.67%)으로 나타났다.

공격, 가격(손, 주먹, 몽둥이로 공격), 따귀를 맞음, 또는 발로 채이거나 두들겨 맞는 등의 신체폭력을 직접 겪은 경우가 8명(2.67%)으로 나타났고, 타인의 겪음을 목격한 경우는 5명(1.67%)으로 나타났다.

홍수나 태풍, 폭풍, 지진과 같은 자연재난을 직접 겪은 경우는 7명(2.33%)이었으며, 타인의 겪음을 목격한 경우는 5명(1.67%)으로 나타났다.

직장이나 집 또는 여가 활동 중 심각한 사고를 직접 겪은 경우는 4명(1.33%)이었으며, 타인의 겪음을 목격한 경우는 3명(1.00%)으로 나타났다.

총이나 칼에 맞거나, 칼, 총, 폭탄으로 위협 당하는 등과 같이 무기에 의한 공격을 직접 겪은 경우는 4명(1.33%)으로 나타났다.

기타 원하지 않거나 불편한 성적 경험을 직접 겪은 경우는 3명(1.00%)이었으며, 타인의 겪음을 목격한 경우는 1명(0.33%)으로 나타났다.

강제적인 노동이나 지속적인 굶주림 또는 식량 부족, 지속적인 노숙 상태, 고문과 같은 심각한 인간적 고난을 직접 겪은 경우는 1명(0.33%)로 나타났다.

화재 또는 폭발을 직접 겪은 경우는 1명(0.33%)으로 나타났다.

성폭행 시도 또는 완력이나 위협 하에 성적 행위를 직접 겪은 경우는 1명

(0.33%)으로 나타났다.

나 자신 때문에 발생했던 다른 사람의 심각한 부상, 상해 또는 사망을 직접 겪은 경우는 1명(0.33%)으로 나타났다.

타인이 살인이나 자살 등의 급작스러운 변사를 당하는 것을 목격한 경우는 5명(1.67%)이었으며, 급작스러운 사고사를 목격한 경우는 3명(1.00%)으로 나타났다.

전투나 전쟁터에 노출되는 상황 또는 납치, 유괴, 인질 및 전쟁 포로 등으로 인해 감금된 경험이나 독성 물질에 노출되는 상황을 직접 또는 간접적으로 겪은 경우는 보고되지 않았다.

〈표 4-35〉 북한이탈주민의 남한에서 경험한 스트레스 사건

요인	구분	빈도(회)	백분율(%)
1. 자연재난	직접겪음	7	2.33
	타인겪음목격	5	1.67
	경험 안함	288	96.00
2. 화재 또는 폭발	직접겪음	1	0.33
	타인겪음목격	8	2.67
	경험 안함	291	97.00
3. 교통사고	직접겪음	31	10.33
	타인겪음목격	15	5.00
	경험 안함	254	84.67
4. 직장, 집, 또는 여가 활동 중 심각한 사고	직접겪음	4	1.33
	타인겪음목격	3	1.00
	경험 안함	293	97.67
5. 독성 물질에 노출	경험 안함	300	100.00
6. 신체폭력	직접겪음	8	2.67
	타인겪음목격	5	1.67
	경험 안함	287	95.67
7. 무기로 공격당함	직접겪음	4	1.33
	경험 안함	296	98.67
8. 성폭력	경험 안함	300	100.00

요인	구분	빈도(회)	백분율(%)
9. 기타 원하지 않거나 불편한 성적 경험	직접겪음	3	1.00
	타인겪음목격	1	0.33
	경험 안함	296	98.67
10. 전투나 전쟁터에 노출	경험 안함	300	100.00
11. 감금	경험 안함	300	100.00
12. 목숨이 좌우될 정도의 질병이나 부상	직접겪음	13	4.33
	타인겪음목격	2	0.67
	경험 안함	285	95.00
13. 심각한 인간적 고난	직접겪음	1	0.33
	경험 안함	299	99.67
14. 급작스러운 변사	타인겪음목격	5	1.67
	경험 안함	295	98.33
15. 급작스러운 사고사	타인겪음목격	3	1.00
	경험 안함	297	99.00
16. 나 자신 때문에 발생했던 다른 사람의 심각한 부상, 상해 또는 사망	직접겪음	1	0.33
	경험 안함	299	99.67
17. 그 밖의 매우 심각한 스트레스 사건이나 경험	직접겪음	37	12.33
	타인겪음목격	3	1.00
	경험 안함	260	86.67
합계		300	100.00

3. 정신건강, 삶의 질 및 건강행태 현황

가. 외상 후 스트레스장애

북한이탈주민의 외상 후 스트레스장애정도를 평가하기 위하여 PTSD checklist (PCL-5)를 이용하였다. 총점 평균은 35.85±22.92점이었고, 최저 점수는 0점이었으며, 최고 점수는 80점으로 나타났다.

〈표 4-36〉 북한이탈주민의 PCL-5 총점 특성: 요약 통계량

요인	구분	평균	표준편차
PCL-5 총점	min=0.00; max=80.00	35.85	22.92

〈표 4-37〉 북한이탈주민의 PCL-5 총점 특성

요인	구분	빈도(회)	백분율(%)
PCL-5 총점	0	13	4.33
	1	3	1.00
	2	6	2.00
	3	4	1.33
	4	4	1.33
	5	2	0.67
	6	7	2.33
	7	4	1.33
	8	3	1.00
	9	6	2.00
	10	10	3.33
	11	3	1.00
	13	4	1.33
	14	4	1.33
	15	6	2.00
	16	3	1.00
	17	5	1.67
	18	6	2.00
	19	2	0.67
	20	2	0.67
	21	6	2.00
	22	1	0.33
	23	3	1.00

요인	구분	빈도(회)	백분율(%)
	24	5	1.67
	25	4	1.33
	26	5	1.67
	27	2	0.67
	28	2	0.67
	29	1	0.33
	30	1	0.33
	31	7	2.33
	33	3	1.00
	34	2	0.67
	35	4	1.33
	36	2	0.67
	37	7	2.33
	38	5	1.67
	39	4	1.33
	41	6	2.00
	42	7	2.33
	43	2	0.67
	44	3	1.00
	45	4	1.33
	46	1	0.33
	47	4	1.33
	48	3	1.00
	49	3	1.00
	50	2	0.67
	51	6	2.00
	52	3	1.00
	53	7	2.33
	54	5	1.67
	55	6	2.00
	56	4	1.33
	57	7	2.33
	58	7	2.33
	59	2	0.67
	60	5	1.67
	61	5	1.67
	62	6	2.00
	63	3	1.00
	64	7	2.33
	65	3	1.00

요인	구분	빈도(회)	백분율(%)
	66	2	0.67
	67	3	1.00
	68	3	1.00
	69	4	1.33
	70	1	0.33
	71	1	0.33
	73	4	1.33
	74	1	0.33
	75	1	0.33
	76	2	0.67
	77	2	0.67
	78	2	0.67
	79	1	0.33
	80	1	0.33
	합계	300	100.00

PCL-5 총점을 기준으로 구분한 결과, ‘정상(32점 이하)’군에 속한 대상자가 132명(44.00%)으로 가장 많았으며, 그다음으로는 ‘주의 요망(33점 이상)’군이 168명(56.00%)순으로 각각 나타났다.

<표 4-38> 북한이탈주민의 PCL-5 군별 특성

요인	구분	빈도(회)	백분율(%)
PCL-5 구분	정상(32점 이하)	132	44.00
	주의 요망(33점 이상)	168	56.00
	합계	300	100.00

PCL-5 1번 문항(그 스트레스 경험에 대한 괴롭고 원하지 않는 기억이 반복적으로 떠오른다)에 따라서는 ‘약간’이 81명(27.00%)으로 가장 많았으며, 그다음으로 ‘많이’가 69명(23.00%), ‘매우 많이’가 56명(18.67%), ‘보통’이 50명(16.67%), ‘전혀 아님’ 44명(14.67%) 순으로 각각 나타났다

PCL-5 2번 문항(그 스트레스 경험과 관련된 악몽을 반복해서 꾼다)에 따라서는 ‘전혀 아님’이 73명(24.33%)으로 가장 많았으며, 그다음으로 ‘많이’가 69명(23.00%), ‘약간’이 66명(22.00%), ‘보통’이 48명(16.00%), ‘매우 많이’가 44명(14.67%) 순으로 각각 나타났다.

PCL-5 3번 문항(갑자기 그 스트레스 경험을 실제로 다시 체험하는 것처럼 느끼거나 행동하게 된다)에 따라서는 '전혀 아님'이 99명(33.00%)으로 가장 많았으며, 그다음으로 '약간'이 76명(25.33%), '보통'이 50명(16.67%), '많이'가 48명(16.00%), '매우 많이'가 27명(9.00%) 순으로 각각 나타났다.

PCL-5 4번 문항(무엇인가 그 스트레스 경험을 떠오르게 하면 마음이 매우 힘들어진다)에 따라서는 '많이'가 81명(27.00%)으로 가장 많았으며, 그다음으로 '매우 많이'가 67명(22.33%), '약간'이 60명(20.00%), '전혀 아님'이 46명(15.33%), '보통'이 46명(15.33%) 순으로 각각 나타났다.

PCL-5 5번 문항(무엇인가 그 스트레스 경험을 떠오르게 하면 강렬한 신체 증상이 나타난다)에 따라서는 '많이'가 76명(25.33%)으로 가장 많았으며, 그다음으로 '전혀 아님'이 70명(23.33%), '매우 많이'가 65명(21.67%), '약간'이 48명(16.00%), '보통'이 41명(13.67%) 순으로 각각 나타났다.

PCL-5 6번 문항(그 스트레스 경험과 관련된 기억, 생각 혹은 감정에서 벗어나려고 한다)에 따라서는 '많이'가 84명(28.00%)으로 가장 많았으며, 그다음으로 '약간'이 69명(23.00%), '매우 많이'가 58명(19.33%), '보통'이 45명(15.00%), '전혀 아님'이 44명(14.67%) 순으로 각각 나타났다.

PCL-5 7번 문항(그 스트레스 경험을 떠오르게 하는 어떤 외부의 자극을 피한다)에 따라서는 '많이'가 79명(26.33%)으로 가장 많았으며, 그다음으로 '전혀 아님'이 61명(20.33%), '보통'이 59명(19.67%), '약간'이 55명(18.33%), '매우 많이'가 46명(15.33%) 순으로 각각 나타났다.

PCL-5 8번 문항(그 스트레스 경험과 관련된 어떤 중요한 부분을 기억해내기 어렵다)에 따라서는 '전혀 아님'이 104명(34.67%)으로 가장 많았으며, 그다음으로 '약간'이 56명(18.67%), '많이'가 54명(18.00%), '보통'이 53명(17.67%), '매우 많이'가 33명(11.00%) 순으로 각각 나타났다.

PCL-5 9번 문항(나 자신, 다른 사람, 혹은 세상에 대해 절대로 안 된다고 생각하는 신념을 가지고 있다)에 따라서는 '전혀 아님'이 100명(33.33%)으로 가장 많았으며, 그다음으로 '많이'가 59명(19.67%), '약간'이 56명(18.67%), '보통'이 55명(18.33%), '매우 많이'가 30명(10.00%) 순으로 각각 나타났다.

PCL-5 10번 문항(그 스트레스 경험이나 그 이후에 일어난 일들에 대해 나 자신이나 누군가를 탓한다)에 따라서는 '전혀 아님'이 90명(30.00%)으로 가장 많았으며, 그다음으로 '약간'이 74명(24.67%), '많이'가 59명(19.67%), '보통'이 53명(17.67%), '매우 많이'가 24명(8.00%) 순으로 각각 나타났다.

PCL-5 11번 문항(두려움, 공포, 분노, 죄책감, 수치심과 같은 강력한 부정적인

감정을 가지고 있다)에 따라서는 ‘전혀 아님’이 94명(31.33%)으로 가장 많았으며, 그다음으로 ‘약간’이 57명(19.00%), ‘많이’가 56명(18.67%), ‘보통’이 54명(18.00%), ‘매우 많이’가 39명(13.00%) 순으로 각각 나타났다.

PCL-5 12번 문항(예전에는 즐거운 마음으로 하던 일이 이제는 다 재미없어졌다)에 따라서는 ‘전혀 아님’이 88명(29.33%)으로 가장 많았으며, 그다음으로 ‘많이’가 63명(21.00%), ‘약간’이 53명(17.67%), ‘매우 많이’가 52명(17.33%), ‘보통’이 44명(14.67%) 순으로 각각 나타났다.

PCL-5 13번 문항(다른 사람들과의 관계가 멀어지거나 끊어졌다고 느낀다)에 따라서는 ‘전혀 아님’이 105명(35.00%)으로 가장 많았으며, 그다음으로 ‘많이’가 58명(19.33%), ‘약간’이 50명(16.67%), ‘매우 많이’가 44명(14.67%), ‘보통’이 43명(14.33%) 순으로 각각 나타났다.

PCL-5 14번 문항(긍정적인 감정을 느끼는데 어려움이 있다)에 따라서는 ‘전혀 아님’이 87명(29.00%)으로 가장 많았으며, 그다음으로 ‘약간’이 61명(20.33%), ‘보통’이 55명(18.33%), ‘많이’가 50명(16.67%), ‘매우 많이’가 47명(15.67%) 순으로 각각 나타났다.

PCL-5 15번 문항(신경질, 분노폭발 혹은 공격적인 행동을 한다)에 따라서는 ‘전혀 아님’이 83명(27.67%)으로 가장 많았으며, 그다음으로 ‘약간’이 74명(24.67%), ‘많이’가 59명(19.67%), ‘보통’이 43명(14.33%), ‘매우 많이’가 41명(13.67%) 순으로 각각 나타났다.

PCL-5 16번 문항(매우 위험한 행동을 하거나 자신에게 해가 될 수 있는 행동을 한다)에 따라서는 ‘전혀 아님’이 158명(52.67%)으로 가장 많았으며, 그다음으로 ‘보통’이 56명(18.67%), ‘약간’이 43명(14.33%), ‘많이’가 31명(10.33%), ‘매우 많이’가 12명(4.00%) 순으로 각각 나타났다.

PCL-5 17번 문항(매우 예민하게 늘 주변을 살피고 경계하게 된다)에 따라서는 ‘전혀 아님’이 91명(30.33%)으로 가장 많았으며, 그다음으로 ‘약간’이 72명(24.00%), ‘많이’가 56명(18.67%), ‘매우 많이’가 41명(13.67%), ‘보통’이 40명(13.33%) 순으로 각각 나타났다.

PCL-5 18번 문항(작은 일에도 쉽게 깜짝깜짝 놀라게 된다)에 따라서는 ‘매우 많이’가 71명(23.67%)으로 가장 많았으며, 그다음으로 ‘전혀 아님’이 62명(20.67%), ‘약간’ 및 ‘많이’가 각각 58명(19.33%), ‘보통’이 51명(17.00%) 순으로 나타났다.

PCL-5 19번 문항(집중하기가 어렵다)에 따라서는 ‘매우 많이’가 69명(23.00%)으로 가장 많았으며, 그다음으로 ‘많이’가 68명(22.67%), ‘약간’이 63명(21.00%), ‘전혀 아님’이 52명(17.33%), ‘보통’이 48명(16.00%) 순으로 각각 나타났다.

PCL-5 20번 문항(잠들기가 어렵거나 깨지 않고 쭉 자기가 어렵다)에 따라서는 ‘매우 많이’가 108명(36.00%)으로 가장 많았으며, 그다음으로 ‘약간’이 59명(19.67%), ‘많이’가 54명(18.00%), ‘전혀 아님’이 45명(15.00%), ‘보통’이 34명(11.33%) 순으로 각각 나타났다.

PCL-5 21번 문항(왠지 나 자신의 미래가 갑자기 끝날 것 같은 느낌이 든다)에 따라서는 ‘전혀 아님’이 97명(32.33%)으로 가장 많았으며, 그다음으로 ‘매우 많이’가 60명(20.00%), ‘약간’이 49명(16.33%), ‘보통’ 및 ‘많이’가 47명(15.67%) 순으로 각각 나타났다.

<표 4-39> 북한이탈주민의 PCL-5 문항별 특성

요인	구분	빈도(회)	백분율(%)
1. 그 스트레스 경험에 대한 괴롭고 원하지 않는 기억이 반복적으로 떠오른다.	전혀 아님	44	14.67
	약간	81	27.00
	보통	50	16.67
	많이	69	23.00
	매우 많이	56	18.67
2. 그 스트레스 경험과 관련된 악몽을 반복해서 꾼다.	전혀 아님	73	24.33
	약간	66	22.00
	보통	48	16.00
	많이	69	23.00
	매우 많이	44	14.67
3. 갑자기 그 스트레스 경험을 실제로 다시 체험하는 것처럼 느끼거나 행동하게 된다.	전혀 아님	99	33.00
	약간	76	25.33
	보통	50	16.67
	많이	48	16.00
	매우 많이	27	9.00
4. 무엇인가 그 스트레스 경험을 떠오르게 하면 마음이 매우 힘들어진다	전혀 아님	46	15.33
	약간	60	20.00
	보통	46	15.33
	많이	81	27.00
	매우 많이	67	22.33
5. 무엇인가 그 스트레스 경험을 떠오르게 하면 강렬한 신체 증상이 나타난다.	전혀 아님	70	23.33
	약간	48	16.00
	보통	41	13.67
	많이	76	25.33
	매우 많이	65	21.67
6. 그 스트레스 경험과 관련된 기억, 생각 혹은 감정에서 벗어나려고 한다.	전혀 아님	44	14.67
	약간	69	23.00
	보통	45	15.00
	많이	84	28.00
	매우 많이	58	19.33

요인	구분	빈도(회)	백분율(%)
7. 그 스트레스 경험을 떠오르게 하는 어떤 외부의 자극을 피한다.	전혀 아님	61	20.33
	약간	55	18.33
	보통	59	19.67
	많이	79	26.33
	매우 많이	46	15.33
8. 그 스트레스 경험과 관련된 어떤 중요한 부분을 기억해내기 어렵다	전혀 아님	104	34.67
	약간	56	18.67
	보통	53	17.67
	많이	54	18.00
	매우 많이	33	11.00
9. 나 자신, 다른 사람, 혹은 세상에 대해 절대로 안 된다고 생각하는 신념(신심)을 가지고 있다	전혀 아님	100	33.33
	약간	56	18.67
	보통	55	18.33
	많이	59	19.67
	매우 많이	30	10.00
10. 그 스트레스 경험이나 그 이후에 일어난 일들에 대해 나 자신이나 누군가를 탓한다.	전혀 아님	90	30.00
	약간	74	24.67
	보통	53	17.67
	많이	59	19.67
	매우많이	24	8.00
11. 두려움, 공포, 분노, 죄책감, 수치심과 같은 강력한 부정적인 감정을 가지고 있다	전혀 아님	94	31.33
	약간	57	19.00
	보통	54	18.00
	많이	56	18.67
	매우 많이	39	13.00
12. 예전에는 즐거운 마음으로 하던 일이 이제는 다 재미없어졌다	전혀 아님	88	29.33
	약간	53	17.67
	보통	44	14.67
	많이	63	21.00
	매우 많이	52	17.33
13. 다른 사람들과의 관계가 멀어지거나 끊어졌다고 느낀다.	전혀 아님	105	35.00
	약간	50	16.67
	보통	43	14.33
	많이	58	19.33
	매우 많이	44	14.67
14. 긍정적인 감정을 느끼는데 어려움이 있다	전혀 아님	87	29.00
	약간	61	20.33
	보통	55	18.33
	많이	50	16.67
	매우 많이	47	15.67
15. 신경질, 분노폭발 혹은 공격적인 행동을 한다.	전혀 아님	83	27.67
	약간	74	24.67
	보통	43	14.33
	많이	59	19.67
	매우 많이	41	13.67

요인	구분	빈도(회)	백분율(%)
16. 매우 위험한 행동을 하거나 자신에게 해가 될 수 있는 행동을 한다.	전혀 아님	158	52.67
	약간	43	14.33
	보통	56	18.67
	많이	31	10.33
	매우 많이	12	4.00
17. 매우 예민하게 늘 주변을 살피고 경계하게 된다.	전혀 아님	91	30.33
	약간	72	24.00
	보통	40	13.33
	많이	56	18.67
	매우 많이	41	13.67
18. 작은 일에도 쉽게 깜짝깜짝 놀라게 된다.	전혀 아님	62	20.67
	약간	58	19.33
	보통	51	17.00
	많이	58	19.33
	매우 많이	71	23.67
19. 집중하기가 어렵다.	전혀 아님	52	17.33
	약간	63	21.00
	보통	48	16.00
	많이	68	22.67
	매우 많이	69	23.00
20. 잠들기가 어렵거나 깨지 않고 쭉 자기가 어렵다	전혀 아님	45	15.00
	약간	59	19.67
	보통	34	11.33
	많이	54	18.00
	매우 많이	108	36.00
21. 왠지 나 자신의 미래가 갑자기 끝날 것 같은 느낌이 든다.	전혀 아님	97	32.33
	약간	49	16.33
	보통	47	15.67
	많이	47	15.67
	매우 많이	60	20.00
합계		300	100.00

* 중복 응답은 허용되지 않음.

4) 성별에 따른 북한이탈주민의 외상 후 스트레스장애정도

성별에 따른 PCL-5 총점에 차이가 있는지를 분석한 결과 여성의 총점 평균은 36.79±23.04점으로 남성의 총점 평균인 31.67±22.08점에 비해 약 16%높게 나타났으나, 통계적으로 유의한 수준의 차이는 아니었다.

〈표 4-40〉 북한이탈주민의 성별에 따른 PCL-5 총점 특성

구분	평균	표준편차	95% 신뢰구간		유의확률
남성	31.67	22.08	25.70	37.64	.135
여성	36.79	23.04	33.89	39.69	

5) 연령에 따른 북한이탈주민의 외상 후 스트레스장애정도

연령대에 따른 PCL-5 총점에 차이가 있는지를 분석한 결과 60대 미만의 총점 평균은 40.68±23.02점으로 60대 이상의 총점 평균인 26.06±19.40점에 비해 약 56% 높게 나타났다. 이는 60대 미만이 60대 이상에 비해 보다 높은 외상 후 스트레스 증상 수준을 나타낼 수 있음을 시사한다.

〈표 4-41〉 북한이탈주민의 연령대에 따른 PCL-5 총점 특성

구분	평균	표준편차	유의확률
20대 이하	24.63	20.23	.000
30대	32.82	21.89	
40대	40.47	24.25	
50대	50.21	18.57	
60대	31.34	21.10	
70대	22.68	16.48	
80대	20.13	23.25	

6) 북한 생활당시 거주지에 따른 북한이탈주민의 외상 후 스트레스장애정도

북한 생활 당시 거주지에 따른 PCL-5 총점은 다음 표와 같다. PCL-5 총점을 함경도 거주자와 그 외의 지역 거주자 간에 차이가 있는지를 분석한 결과 함경도 거주자의 총점 평균은 36.53±22.43점으로 함경도 외의 지역 거주자의 총점 평균인 34.81±21.69점에 비해 약 5% 높게 나타났으나, 통계적으로 유의한 수준의 차이는

아니었다.

<표 4-42> 북한이탈주민의 북한 생활당시 거주지에 따른 PCL-5 총점 특성

구분	평균	표준편차	유의확률
강원도	36.50	43.13	.529
양강도	34.56	22.85	
자강도	13.67	10.07	
평안도	40.78	22.96	
함경도	36.53	22.43	
황해도	22.27	26.46	

7) 남한입국과정 차이에 따른 북한이탈주민의 외상 후 스트레스장애정도

남한입국과정 차이에 따른 북한이탈주민의 외상 후 스트레스장애 특성을 알아보기 위하여 외상 후 스트레스장애 체크리스트(PCL-5 with a item of PCL-IV) 총점에 차이가 있는지를 분석한 결과, 중국 경유자의 총점 평균은 38.92±23.19점으로 남한 직행자의 총점 평균인 30.48±21.69점에 비해 유의하게 약 28% 높게 나타났다. 이는 중국 경유자가 남한 직행자에 비해 더 높은 외상 후 스트레스장애 수준을 나타냄을 시사한다.

<표 4-43> 남한입국과정 차이에 따른 북한이탈주민의 외상 후 스트레스장애 특성

구분	평균	표준편차	95% 신뢰구간		유의확률
남한 직행	30.45	21.69	26.36	34.55	.002
중국 경유*	38.92	23.19	35.52	42.32	

*중국을 통해서 넘어온 수준이 아닌 1년이상 중국에 거주했을 경우를 의미

나. 우울

북한이탈주민의 우울 정도를 평가하기 위하여 건강질문지-9 (Patient Health Questionnaire, PHQ-9)를 이용하였다. 총점 평균은 13.00±8.27점이었고, 최저 점수는 0점이었으며, 최고 점수는 27점으로 나타났다.

〈표 4-44〉 북한이탈주민의 PHQ-9 총점 특성: 요약 통계량

요인	구분	평균	표준편차
PHQ-9 총점	min=0; max=27	13.00	8.27

〈표 4-45〉 북한이탈주민의 PHQ-9 총점 특성

요인	구분	빈도(회)	백분율(%)
PHQ-9 총점	0	16	5.33
	1	8	2.67
	2	14	4.67
	3	12	4.00
	4	11	3.67
	5	14	4.67
	6	11	3.67
	7	7	2.33
	8	14	4.67
	9	12	4.00
	10	8	2.67
	11	13	4.33
	12	9	3.00
	13	10	3.33
	14	7	2.33
	15	9	3.00
	16	9	3.00
	17	7	2.33
	18	13	4.33
	19	11	3.67
	20	13	4.33
	21	11	3.67
	22	10	3.33
	23	17	5.67
	24	9	3.00
	25	5	1.67
	26	3	1.00
	27	17	5.67
	합계	300	100.00

PHQ-9 총점을 기준으로 구분한 결과 정상(4점 이하)군이 62명(20.67%), 경도(5점 이상 ~ 9점 이하)군이 57명(19.00%), 중등도 우울(10점 이상 ~ 19점 이하)군에 속한 대상자가 96명(32.00%), 고도의 우울(20점 이상 ~ 27점 이하)군이 85명(28.33%) 순으로 각각 나타났다.

<표 4-46> 북한이탈주민의 PHQ-9 군별 특성

요인	구분	빈도(회)	백분율(%)
PHQ-9 구분	정상(4점 이하)	62	20.67
	경도(5점 이상 ~ 9점 이하)	57	19.00
	중등도(10점 이상 ~ 19점 이하)	96	32.00
	고도(20점 이상 ~ 27점 이하)	85	28.33
	합계	300	100.00

PHQ-9 1번 문항(어떤 일에도 관심이나 재미가 없음)에 따라서는 '없음'이 113명(37.67%)로 가장 많았으며, 그다음으로 '거의 매일'이 87명(29.00%), '2일 이상'이 57명(19.00%), '1주일 이상'이 43명(14.33%) 순으로 각각 나타났다.

PHQ-9 2번 문항(처지는 느낌, 우울감 혹은 절망감)에 따라서는 '거의 매일'이 93명(31.00%)으로 가장 많았으며, 그다음으로 '없음'이 82명(27.33%), '2일 이상'이 67명(22.33%), '1주일 이상'이 58명 (19.33%) 순으로 각각 나타났다.

PHQ-9 3번 문항(잠들기 어려움, 깨지 않고 쭉 자기 어려움, 혹은 너무 많이 잠)에 따라서는 '거의 매일'이 146명(48.67%)으로 가장 많았으며, 그다음으로 '2일 이상'이 62명(20.67%), '없음'이 60명(20.00%), '1주일 이상'이 32명(10.67%) 순으로 각각 나타났다.

PHQ-9 4번 문항(피곤함 혹은 기운 없음)에 따라서는 '거의 매일'이 140명(46.67%)으로 가장 많았으며, 그다음으로 '2일 이상'이 66명(22.00%), '없음'이 47명(15.67%), '1주일 이상'이 47명(15.67%) 순으로 각각 나타났다.

PHQ-9 5번 문항(식욕 저하 혹은 과식)에 따라서는 '거의 매일'이 82명(27.33%)으로 가장 많았으며, 그다음으로 '없음'이 79명(26.33%), '1주일 이상'이 71명(23.67%), '2일 이상'이 68명(22.67%) 순으로 각각 나타났다.

PHQ-9 6번 문항(나 자신을 못마땅하게 여김)에 따라서는 '없음'이 113명(37.67%)으로 가장 많았으며, 그다음으로 '거의 매일'이 86명(28.67%), '1주일 이상'이

52명(17.33%), ‘2일 이상’이 49명(16.33%) 순으로 각각 나타났다.

PHQ-9 7번 문항(나 자신이 실패자 같음, 혹은 나 자신이나 가족을 실망시킨 것 같음)에 따라서는 ‘없음’이 118명(39.33%)으로 가장 많았으며, 그다음으로 ‘거의 매일’이 90명(30.00%), ‘2일 이상’이 47명(15.67%), ‘1주일 이상’ 45명(15.00%) 순으로 각각 나타났다.

PHQ-9 8번 문항(신문 읽기나 텔레비전 시청과 같은 일상적인 일에도 집중하기 어려움)에 따라서는 ‘없음’이 143명(47.67%)으로 가장 많았으며, 그다음으로 ‘거의 매일’이 62명(20.67%), ‘2일 이상’이 49명(16.33%), ‘1주일 이상’이 46명(15.33%) 순으로 각각 나타났다.

PHQ-9 9번 문항(움직이는 것이나 말하는 것이 너무 느려서 남들이 알아챌 정도임)에 따라서는 ‘없음’이 163명(54.33%)으로 가장 많았으며, 그다음으로 ‘거의 매일’이 57명(19.00%), ‘2일 이상’이 41명(13.67%), ‘1주일 이상’이 39명(13.00%) 순으로 나타났다.

PHQ-9 10번 문항(위의 해당 증상 때문에 직장이나 집안일을 할 때 혹은 주변 사람들과의 관계에서 어려움을 겪은 정도)에 따라서는 ‘조금 어려웠다’가 111명(37.00%)으로 가장 많았으며, 그다음으로 ‘전혀 없었다’가 73명(24.33%)으로 가장 많았으며, 그다음으로 ‘많이 어려웠다’가 71명(23.67%), ‘아주 어려웠다’가 44명(14.67%) 순으로 나타났다.

<표 4-47> 북한이탈주민의 PHQ-9 문항별 특성

요인	구분	빈도(회)	백분율(%)
1. 어떤 일에도 관심이나 재미가 없음	없음	113	37.67
	2일이상	57	19.00
	1주이상	43	14.33
	거의매일	87	29.00
2. 처지는 느낌, 우울감 혹은 절망감	없음	82	27.33
	2일이상	67	22.33
	1주이상	58	19.33
	거의매일	93	31.00
3. 잠들기 어려움, 깨지 않고 쭉 자기 어려움, 혹은 너무 많이 잠	없음	60	20.00
	2일이상	62	20.67
	1주이상	32	10.67
	거의매일	146	48.67
4. 피곤함 혹은 기운 없음	없음	47	15.67
	2일이상	66	22.00
	1주이상	47	15.67
	거의매일	140	46.67
5. 식욕(입맛) 저하 혹은 과식	없음	79	26.33
	2일이상	68	22.67
	1주이상	71	23.67
	거의매일	82	27.33
6. 나 자신을 못마땅하게 여김	없음	113	37.67
	2일이상	49	16.33
	1주이상	52	17.33
	거의매일	86	28.67
7. 나 자신이 실패자 같음, 혹은 나 자신이나 가족을 실망시킨 것 같음	없음	118	39.33
	2일이상	47	15.67
	1주이상	45	15.00
	거의매일	90	30.00
8. 신문 읽기나 텔레비전 시청과 같은 일상적인 일에도 집중하기 어려움	없음	143	47.67
	2일이상	49	16.33
	1주이상	46	15.33
	거의매일	62	20.67
9. 움직이는 것이나 말하는 것이 너무 느려서 남들이 알아챌 정도임.	없음	163	54.33
	2일이상	41	13.67
	1주이상	39	13.00
	거의매일	57	19.00
10a. 위의 해당 증상 때문에 직장이나 집안일을 할 때 혹은 주변 사람들과의 관계에서 어려움을 겪은 정도	전혀 없었다	73	24.33
	조금 어려웠다	111	37.00
	많이 어려웠다	71	23.67
	아주 어려웠다	44	14.67
	결측	1	0.33
합계		300	100.00

* 중복 응답은 허용되지 않음.

다. 자살경향성

북한이탈주민의 자살경향성 정도를 평가하기 위하여 K-MINI를 이용하였다. 총점 평균은 6.95±10.11점이었고, 최저 점수는 0점이었으며, 최고 점수는 33점으로 나타났다.

<표 4-48> 북한이탈주민의 K-MINI 총점 특성: 요약 통계량

요인	구분	평균	표준편차
K-MINI 총점	min=0.00; max=33.00	6.95	10.11

<표 4-49> 북한이탈주민의 K-MINI 총점 특성

요인	구분	빈도(회)	백분율(%)
K-MINI 총점	0	131	43.67
	1	11	3.67
	2	4	1.33
	3	2	0.67
	4	33	11.00
	5	3	1.00
	6	16	5.33
	7	21	7.00
	8	2	0.67
	9	7	2.33
	10	5	1.67
	11	8	2.67
	12	1	0.33
	13	9	3.00
	14	1	0.33
	17	1	0.33
	19	1	0.33
	20	2	0.67
	21	4	1.33
	22	2	0.67
	23	7	2.33
	29	1	0.33
	30	1	0.33
	32	4	1.33
	33	23	7.67
	합계	300	100.00

K-MINI 총점을 기준으로 구분한 결과, '낮은 자살 위험성(5점 이하)'군에 속한 대상자가 184명(61.33%), 중간 정도의 자살 위험성(6점 이상 ~ 9점 이하)'군이 46명(15.33%), '높은 자살 위험성(10점 이상 ~ 19점 이하)'군이 70명(23.33%)으로 나타났다.

<표 4-50> 북한이탈주민의 K-MINI 군별 특성

요인	구분	빈도(회)	백분율(%)
K-MINI 구분	낮음(5점 이하)	184	61.33
	중간(6점 이상 ~ 9점 이하)	46	15.33
	높음(10점 이상 ~ 19점 이하)	70	23.33
	합계	300	100.00

K-MINI 1번 문항(지난 1개월간 당신은 차라리 죽는 것이 더 낫다고 생각하거나 죽기를 바랐던 적이 있습니까?)에 따라서는 '아니오'가 186명(62.00%)으로 더 많았으며, '예'는 114명(38.00%)으로 나타났다.

K-MINI 2번 문항(지난 1개월간 당신은 자해 -본인에게 상처를 내거나 해를 끼치는 것- 를 하고 싶었던 적이 있습니까?)에 따라서는 '아니오'가 232명(77.33%)으로 더 많았으며, '예'는 68명(22.67%)으로 나타났다.

K-MINI 3번 문항(지난 1개월간 당신은 자살에 대해서 생각했던 적이 있습니까?)에 따라서는 '아니오'가 191명(63.67%)으로 더 많았으며, '예'는 109명(36.33%)으로 나타났다.

K-MINI 4번 문항(지난 1개월간 당신은 자살을 계획했었습니까?)에 따라서는 '아니오'가 257 (85.67%)으로 더 많았으며, '예'는 43명(14.33%)으로 나타났다.

K-MINI 5번 문항(지난 1개월간 당신은: 자살을 시도했습니까?)에 따라서는 '아니오'가 266명(88.67%)으로 더 많았으며, '예'는 34명(11.33%)으로 나타났다.

K-MINI 6번 문항(일생동안 한 차례라도 자살 시도를 한 적이 있습니까?)에 따라서는 '아니오'가 193명(64.33%)으로 더 많았으며, '예'는 107명(35.67%)으로 나타났다.

〈표 4-51〉 북한이탈주민의 K-MINI 문항별 특성

요인	구분	빈도(회)	백분율(%)
1. 지난 1개월간 당신은: 차라리 죽는 것이 더 낫다고 생각하거나 죽기를 바랐던 적이 있습니까?	아니오	186	62.00
	예	114	38.00
2. 지난 1개월간 당신은: 자해 (본인에게 상처를 내는 등 해를 끼치는)를 하고 싶었던 적이 있습니까?	아니오	232	77.33
	예	68	22.67
3. 지난 1개월간 당신은: 자살에 대해서 생각했던 적이 있습니까?	아니오	191	63.67
	예	109	36.33
4. 지난 1개월간 당신은: 자살을 계획했었습니까?	아니오	257	85.67
	예	43	14.33
5. 지난 1개월간 당신은: 자살을 시도했습니까?	아니오	266	88.67
	예	34	11.33
6. 일생동안 한 차례라도 자살 시도를 한 적이 있습니까?	아니오	193	64.33
	예	107	35.67
합계		300	100.00

* 중복 응답은 허용되지 않음.

라. 불안증상

북한이탈주민의 불안장애 정도를 평가하기 위하여 Generalized Anxiety Disorder Scale 7-item (GAD-7)을 이용하였다. 총점 평균은 8.66±6.27점이었고, 최저 점수는 0점이었으며, 최고 점수는 18점으로 나타났다.

<표 4-52> 북한이탈주민의 GAD-7 총점 특성: 요약 통계량

요인	구분	평균	표준편차
GAD-7 총점	min=0.00; max=18.00	8.66	6.27

<표 4-53> 북한이탈주민의 GAD-7 총점 특성

요인	구분	빈도(회)	백분율(%)
GAD-7 총점	0	33	11.00
	1	24	8.00
	2	26	8.67
	3	13	4.33
	4	13	4.33
	5	4	1.33
	6	12	4.00
	7	4	1.33
	8	14	4.67
	9	11	3.67
	10	15	5.00
	11	14	4.67
	12	15	5.00
	13	16	5.33
	14	11	3.67
	15	15	5.00
	16	21	7.00
	17	10	3.33
	18	29	9.67
	합계	300	100.00

GAD-7 총점을 기준으로 구분한 결과, '정상(4점 이하)'군에 속한 대상자가 109명(36.33%), 경도(5점 이상 ~ 9점 이하)'군이 45명(15.00%), 중등도(10점 이상 ~ 14점 이하)'군이 71명(23.67%), 그다음으로는 '고도(15점 이상)'군이 75명(25.00%)으로 각각 나타났다.

<표 4-54> 북한이탈주민의 GAD-7 군별 특성

요인	구분	빈도(회)	백분율(%)
GAD-7 구분	정상(4점 이하)	109	36.33
	경도(5점 이상 ~ 9점 이하)	45	15.00
	중등도(10점 이상 ~ 14점 이하)	71	23.67
	고도(15점 이상)	75	25.00
	합계	300	100.00

GAD-7 1번 문항(초조, 불안, 조마조마함)에 따라서는 '없음'이 94명(31.33%)으로 가장 많았으며, 그다음으로 '거의 매일'이 83명(27.67%), '2일 이상'이 69명(23.00%), '1주일 이상'이 54명(18.00%) 순으로 각각 나타났다.

GAD-7 2번 문항(걱정하는 것을 멈추거나 조절할 수 없음)에 따라서는 '없음'이 97명(32.33%)으로 가장 많았으며, 그다음으로 '거의 매일'이 92명(30.67%), '2일 이상'이 59명(19.67%), '1주일 이상'이 52명(17.33%) 순으로 각각 나타났다.

GAD-7 3번 문항(이런저런 다양한 걱정을 너무 많이 함)에 따라서는 '거의 매일'이 124명(41.33%)으로 가장 많았으며, 그다음으로는 '2일 이상'이 77명(25.67%), '없음'이 50명(16.67%), '1주일 이상'이 49명(16.33%) 순으로 각각 나타났다.

GAD-7 4번 문항(진정하기 어려움)에 따라서는 '없음'이 100명(33.33%)으로 가장 많았으며, 그다음으로는 '거의 매일'이 85명(28.33%), '2일 이상'이 59명(19.67%), '1주일 이상'이 56명(18.67%) 순으로 각각 나타났다.

GAD-7 5번 문항(가만히 있지 못할 정도로 안절부절 못함)에 따라서는 '없음'이 111명(37.00%)으로 가장 많았으며, 그다음으로는 '2일 이상'이 68명(22.67%), '1주일 이상'이 64명(21.33%), '거의 매일'이 57명(19.00%) 순으로 각각 나타났다.

GAD-7 6번 문항(쉽게 짜증이 남)에 따라서는 '거의 매일'이 101명(33.67%)로 가장 많았으며, 그다음으로는 '2일 이상'이 80명(26.67%), '없음'이 72명(24.00%), '1주일 이상'이 47명(15.67%) 순으로 각각 나타났다.

GAD-7 7번 문항(끔찍한 일이 생길 것 같은 두려움이 생김)에 따라서는 '없음'이 137명(45.67%)으로 가장 많았으며, '거의 매일'이 63명(21.00%), '1주일 이상'이 52명(17.33%), '2일 이상'이 48명(16.00%) 순으로 각각 나타났다.

<표 4-55> 북한이탈주민의 GAD-7 문항별 특성

요인	구분	빈도(회)	백분율(%)
1. 초조, 불안, 조마조마함	없음	94	31.33
	2일 이상	69	23.00
	1주일 이상	54	18.00
	거의 매일	83	27.67
2. 걱정하는 것을 멈추거나 조절할 수 없음	없음	97	32.33
	2일 이상	59	19.67
	1주일 이상	52	17.33
	거의 매일	92	30.67
3. 이런저런 다양한 걱정을 너무 많이 함	없음	50	16.67
	2일 이상	77	25.67
	1주일 이상	49	16.33
	거의 매일	124	41.33
4. 진정하기 어려움	없음	100	33.33
	2일 이상	59	19.67
	1주일 이상	56	18.67
	거의 매일	85	28.33
5. 가만히 있지 못할 정도로 안절부절 못함	없음	111	37.00
	2일 이상	68	22.67
	1주일 이상	64	21.33
	거의 매일	57	19.00
6. 쉽게 짜증이 남	없음	72	24.00
	2일 이상	80	26.67
	1주일 이상	47	15.67
	거의 매일	101	33.67
7. 끔찍한 일이 생길 것 같은 두려움이 생김	없음	137	45.67
	2일 이상	48	16.00
	1주일 이상	52	17.33
	거의 매일	63	21.00
합계		300	100.00

* 중복 응답은 허용되지 않음.

마. 불면증

북한이탈주민의 불면증 심각성에 대한 정도를 평가하기 위하여 Insomnia Severity Index (ISI)를 이용하였다. 총점 평균은 14.29±8.02점이었고, 최저 점수는 0점이었으며, 최고 점수는 28점으로 나타났다.

<표 4-56> 북한이탈주민의 ISI 총점 특성: 요약 통계량

요인	구분	평균	표준편차
ISI 총점	min=0.00; max=28.00	14.29	8.02

<표 4-57> 북한이탈주민의 ISI 총점 특성

요인	구분	빈도(회)	백분율(%)
ISI 총점	0	5	1.67
	1	9	3.00
	2	7	2.33
	3	10	3.33
	4	15	5.00
	5	12	4.00
	6	10	3.33
	7	12	4.00
	8	10	3.33
	9	6	2.00
	10	13	4.33
	11	9	3.00
	12	12	4.00
	13	9	3.00
	14	12	4.00
	15	7	2.33
	16	10	3.33
	17	11	3.67
	18	8	2.67
	19	9	3.00
	20	21	7.00
	21	13	4.33
	22	14	4.67
	23	15	5.00
	24	8	2.67
	25	4	1.33
	26	16	5.33
	27	6	2.00
	28	7	2.33
	합계	300	100.00

ISI 총점을 기준으로 구분한 결과, ‘정상(7점 이하)’가 80명(26.67%), 경도(8점 이상 ~ 14점 이하)’가 71명(23.67%), ‘중등도(15점 이상 ~ 21점 이하)’가 79명(26.33%), 고도(22점 이상 ~ 28점 이하)’가 70명(23.33%)으로 각각 나타났다.

<표 4-58> 북한이탈주민의 ISI 군별 특성

요인	구분	빈도(회)	백분율(%)
ISI 구분	정상(7점 이하)	80	26.67
	경도(8점 이상 ~ 14점 이하)	71	23.67
	중등도(15점 이상 ~ 21점 이하)	79	26.33
	고도(22점 이상 ~ 28점 이하)	70	23.33
	합계	300	100.00

ISI 1번 문항(잠들기 어려움)에 따라서는 ‘심함’이 69명(23.00%)으로 가장 많았으며, 그다음으로는 ‘매우’가 68명(22.67%), ‘약간’이 62명(20.67%), ‘없음’이 55명(18.33%), ‘중간’이 46명(15.33%) 순으로 각각 나타났다.

ISI 2번 문항(깊은 잠을 자기 어려움)에 따라서는 ‘매우’가 77명(25.67%)으로 가장 많았으며, 그다음으로는 ‘심함’이 62명(20.67%), ‘없음’이 57명(19.00%), ‘중간’이 55명(18.33%), ‘약간’이 49명(16.33%) 순으로 각각 나타났다.

ISI 3번 문항(너무 일찍 깨는 문제)에 따라서는 ‘심함’이 67명(22.33%)으로 가장 많았으며, 그다음으로는 ‘없음’이 63명(21.00%), ‘매우’가 62명(20.67%), ‘약간’이 57명(19.00%), ‘중간’이 51명(17.00%)으로 각각 나타났다.

ISI 4번 문항(현재 당신의 수면 패턴에 대해 얼마나 만족하고 있습니까?)에 따라서는 ‘중간’이 106명(35.33%)으로 가장 많았으며, 그다음으로는 ‘불만족’이 87명(29.00%), ‘매우 불만족’이 51명(17.00%), ‘만족’이 43명(14.33%), ‘매우 만족’이 43명(4.33%) 순으로 각각 나타났다.

ISI 5번 문항(불면증이 일상생활을 어느 정도 방해한다고 생각합니까?)에 따라서는 ‘조금 방해됨’이 91명(30.33%)으로 가장 많았으며, 그다음으로는 ‘다소 방해됨’이 67명(22.33%), ‘많이 방해됨’이 56명(18.67%), ‘매우 방해됨’이 48명(16.00%), ‘전혀 방해 안됨’이 38명(12.67%) 순으로 각각 나타났다.

ISI 6번 문항(불면증으로 인해 제대로 생활을 할 수 없는 당신을 다른 사람들은 어떻게 볼 거라고 생각합니까?)에 따라서는 ‘조금 알아봄’이 85명(28.33%)으로 가장 많았으며, 그다음으로는 ‘전혀 못 알아봄’이 80명(26.67%), ‘다소 알아봄’이 52명(17.33%), ‘많이 알아봄’이 51명(17.00%), ‘매우 많이 알아봄’이 32명(10.67%) 순으로 각각 나타났다.

ISI 7번 문항(현재 불면증에 관하여 얼마나 걱정하고 있습니까?)에 따라서는

‘많이’가 76명(25.33%)으로 가장 많았으며, 그다음으로는 ‘조금’이 67명(22.33%), ‘매우 많이’가 61명(20.33%), ‘전혀’가 56명(18.67%), ‘다소’가 40명(13.33%) 순으로 각각 나타났다.

〈표 4-59〉 북한이탈주민의 ISI 문항별 특성

요인	구분	빈도(회)	백분율(%)
1. 잠들기 어려움	없음	55	18.33
	약간	62	20.67
	중간	46	15.33
	심함	69	23.00
	매우	68	22.67
2. 깊은 잠을 자기 어려움	없음	57	19.00
	약간	49	16.33
	중간	55	18.33
	심함	62	20.67
	매우	77	25.67
3. 너무 일찍 깨는 문제	없음	63	21.00
	약간	57	19.00
	중간	51	17.00
	심함	67	22.33
	매우	62	20.67
4. 현재 당신의 수면 패턴(틀, 규칙)에 대해 얼마나 만족하고 있습니까?	매우 만족	13	4.33
	만족	43	14.33
	중간	106	35.33
	불만족	87	29.00
	매우 불만족	51	17.00
5. 불면증이 일상생활을 어느 정도 방해한다고 생각합니까?	전혀 방해 안됨	38	12.67
	조금 방해됨	91	30.33
	다소 방해됨	67	22.33
	많이 방해됨	56	18.67
	매우 방해됨	48	16.00
6. 불면증으로 인해 제대로 생활을 할 수 없는 당신을 다른 사람들은 어떻게 볼 거라고 생각합니까?	전혀 못 알아봄	80	26.67
	조금 알아봄	85	28.33
	다소 알아봄	52	17.33
	많이 알아봄	51	17.00
	매우 많이 알아봄	32	10.67
7. 현재 불면증에 관하여 얼마나 걱정하고 있습니까?	전혀	56	18.67
	조금	67	22.33
	다소	40	13.33
	많이	76	25.33
	매우 많이	61	20.33
합계		300	100.00

* 중복 응답은 허용되지 않음.

바. 삶의 질

북한이탈주민의 건강관련 삶의 질을 평가하기 위하여 SF-8(Short Form-8 Health survey)를 이용하였다. 총점 평균은 26.77±8.08점이었고, 최저 점수는 1점이었으며, 최고 점수는 42점으로 나타났다

<표 4-60> 북한이탈주민의 SF-8 총점 특성: 요약 통계량

요인	구분	평균	표준편차
SF-8 총점	min=1.00; max=42.00	26.77	8.08

<표 4-61> 북한이탈주민의 SF-8 총점 특성

요인	구분	빈도(회)	백분율(%)
SF-8 총점	1	1	0.33
	8	1	0.33
	10	5	1.67
	11	2	0.67
	12	6	2.00
	13	5	1.67
	14	8	2.67
	15	7	2.33
	16	4	1.33
	17	9	3.00
	18	4	1.33
	19	6	2.00
	20	11	3.67
	21	12	4.00
	22	10	3.33
	23	7	2.33
	24	17	5.67
	25	13	4.33
	26	10	3.33
	27	12	4.00
	28	10	3.33

요인	구분	빈도(회)	백분율(%)
	29	16	5.33
	30	13	4.33
	31	12	4.00
	32	14	4.67
	33	11	3.67
	34	15	5.00
	35	7	2.33
	36	17	5.67
	37	14	4.67
	38	10	3.33
	39	4	1.33
	40	3	1.00
	41	2	0.67
	42	2	0.67
	합계	300	100.00

SF-8 1번 문항(전반적으로 지난 한 달 동안 당신의 건강 상태는 어떠하였습니까?)에 따라서는 ‘그저 그랬다’가 101명(33.67%)으로 가장 많았으며, 그다음으로는 ‘나빴다’가 86명(28.67%), ‘좋았다’가 35명(11.67%), ‘상당히 좋았다’가 12명(4.00%), ‘최고로 좋았다’가 2명(0.67%) 순으로 각각 나타났다.

SF-8 2번 문항(지난 한 달 동안 당신은 신체적인 건강 문제 때문에 일상적인 신체활동에 어느 정도의 제한을 받았습니까?)에 따라서는 ‘어느 정도 받았다’가 107명(35.67%)으로 가장 많았으며, 그다음으로는 ‘상당히 많이 받았다’가 88명(29.33%), ‘거의 받지 않았다’가 47명(15.67%), ‘전혀 받지 않았다’가 34명(11.33%), ‘신체 활동을 할 수 없었다’가 24명(8.00%) 순으로 각각 나타났다.

SF-8 3번 문항(지난 한 달 동안 당신은 신체적인 건강 때문에 집에서나 밖에서 일상적인 일을 하는데 얼마나 어려움이 있었습니까?)에 따라서는 ‘상당히 많이 있었다’가 89명(29.67%)으로 가장 많았으며, 그다음으로는 ‘조금 있었다’가 74명(24.67%), ‘어느 정도 있었다’가 64명(21.33%), ‘전혀 없었다’가 45명(15.00%), ‘일상적인 일을 할 수 없었다’가 28명(9.33%) 순으로 각각 나타났다.

SF-8 4번 문항(지난 한 달 동안 당신의 몸에는 통증이 얼마나 있었습니까?)에

따라서는 ‘심한 통증이 있었다’가 77명(25.67%)으로 가장 많았으며, 그다음으로는 ‘어느 정도의 통증이 있었다’가 61명(20.33%), ‘매우 심한 통증이 있었다’가 49명(16.33%), ‘가벼운 통증이 있었다’가 41명(13.67), ‘아주 가벼운 통증이 있었다’가 40명(13.33%), ‘전혀 없었다’가 32명(10.67%) 순으로 각각 나타났다.

SF-8 5번 문항(지난 한 달 동안 당신은 어느 정도의 활력이 있었습니까?)에 따라서는 ‘어느 정도 있었다’가 112명(37.33%)으로 가장 많았으며, 그다음으로는 ‘아주 조금 있었다’가 92명(30.67%), ‘전혀 없었다’가 49명(16.33%), ‘꽤 많이 있었다’가 35명(11.67%), ‘전혀 없었다’가 32명(10.67%), ‘굉장히 많이 있었다’가 12명(4.00%) 순으로 각각 나타났다.

SF-8 6번 문항(지난 한 달 동안 당신은 신체적인 건강 혹은 정신적인 문제 때문에 친척이나 친구들을 만나거나 모임에 참석하는데 어느 정도의 제한을 받았습니까?)에 따라서는 ‘어느 정도 받았다’가 84명(28.00%)으로 가장 많았으며, 그다음으로는 ‘전혀 받지 않았다’가 63명(21.00%), ‘거의 받지 않았다’가 54명(18.00%), ‘교제나 모임에 참석할 수 없었다’가 50명(16.67%), ‘상당히 많이 받았다’가 49명(16.33%) 순으로 각각 나타났다.

SF-8 7번 문항(지난 한 달 동안 당신은 정신적인 문제 때문에 얼마나 괴로움을 당했습니까?)에 따라서는 ‘꽤 많이 괴로웠다’가 68명(22.67%)으로 가장 많았으며, 그다음으로는 ‘아주 심하게 괴로웠다’가 61명(20.33%), ‘어느 정도 괴로웠다’가 60명(20.00%), ‘전혀 괴로움이 없었다’가 59명(19.67%), ‘아주 조금 괴로웠다’가 52명(17.33%) 순으로 각각 나타났다.

SF-8 8번 문항(지난 한 달 동안 개인적인 또는 정신적인 문제 때문에 당신이 일상적인 활동을 하는데 얼마나 지장을 받았습니까?)에 따라서는 ‘어느 정도 받았다’가 90명(30.00%)으로 가장 많이 받았으며, 그다음으로는 ‘상당히 많이 받았다’가 86명(28.67%), ‘전혀 받지 않았다’가 49명(16.33%), ‘거의 받지 않았다’가 39명(13.00%), ‘일상적인 일을 할 수 없었다’가 36명(12.00%) 순으로 각각 나타났다.

<표 4-62> 북한이탈주민의 SF-8 문항별 특성

요인	구분	빈도(회)	백분율(%)
1. 전반적으로 지난 한 달 동안 당신의 건강 상태는 어떠하였습니까?	최고로 좋았다	2	0.67
	상당히 좋았다	12	4.00
	좋았다	35	11.67
	그저 그랬다	101	33.67
	나빴다	86	28.67
	아주 나빴다	64	21.33
2. 지난 한 달 동안 당신은 신체적인 건강 문제 때문에 일상적인 신체활동에 어느 정도의 제한을 받았습니까?	전혀 받지 않았다	34	11.33
	거의 받지 않았다	47	15.67
	어느 정도 받았다	107	35.67
	상당히 많이 받았다	88	29.33
	신체 활동을 할 수 없었다	24	8.00
3. 지난 한 달 동안 당신은 신체적인 건강 때문에 집에서나 밖에서 일상적인 일을 하는데 얼마나 어려움이 있었습니까?	전혀 없었다	45	15.00
	조금 있었다	74	24.67
	어느 정도 있었다	64	21.33
	상당히 많이 있었다	89	29.67
	일상적인 일을 할 수 없었다	28	9.33
4. 지난 한 달 동안 당신의 몸에는 통증이 얼마나 있었습니까?	전혀 없었다	32	10.67
	아주 가벼운 통증이 있었다	40	13.33
	가벼운 통증이 있었다	41	13.67
	어느 정도의 통증이 있었다	61	20.33
	심한 통증이 있었다	77	25.67
	매우 심한 통증이 있었다	49	16.33
5. 지난 한 달 동안 당신은 어느 정도의 활력이 있었습니까?	굉장히 많이 있었다	12	4.00
	꽤 많이 있었다	35	11.67
	어느 정도 있었다	112	37.33
	아주 조금 있었다	92	30.67
	전혀 없었다	49	16.33
6. 지난 한 달 동안 당신은 신체적인 건강 혹은 정신적인 문제 때문에 친척이나 친구들을 만나거나 모임에 참석하는데 어느 정도의 제한을 받았습니까?	전혀 받지 않았다	63	21.00
	거의 받지 않았다	54	18.00
	어느 정도 받았다	84	28.00
	상당히 많이 받았다	49	16.33
	교제나 모임에 참석할 수 없었다	50	16.67
7. 지난 한 달 동안 당신은 정신적인 문제 때문에 얼마나 괴로움을 당했습니까?	전혀 괴로움이 없었다	59	19.67
	아주 조금 괴로웠다	52	17.33
	어느 정도 괴로웠다	60	20.00
	꽤 많이 괴로웠다	68	22.67
	아주 심하게 괴로웠다	61	20.33
8. 지난 한 달 동안 개인적인 또는 정신적인 문제 때문에 당신이 일상적인 활동을 하는데 얼마나 지장을 받았습니까?	전혀 받지 않았다	49	16.33
	거의 받지 않았다	39	13.00
	어느 정도 받았다	90	30.00
	상당히 많이 받았다	86	28.67
	일상적인 일을 할 수 없었다	36	12.00
합계		300	100.00

* 중복 응답은 허용되지 않음.

사. 건강행태

북한이탈주민의 건강행태에 관한 특성은 흡연과 음주로 구성되어 있으며, 각 특성별 분석 결과는 다음과 같다.

1) 흡연

북한이탈주민의 흡연 행태는 흡연기간(년)과 일평균 흡연량(개비)로 파악하였다. 비흡연자가 276명(92.00%)으로 대부분을 차지한 것으로 나타났다. 흡연자 24명(8.00%)의 평균 흡연기간은 평균 33.58±17.47년이었고 2~60년 사이에 분포하였다. 흡연기간 중 일평균 흡연량은 평균 12.25±5.57개비였으며, 1~20개비 사이에 분포하였다.

〈표 4-63〉 북한이탈주민의 흡연 특성: 요약 통계량

요인	구분	평균	표준편차
흡연기간(년)	min=2; max=60	33.58	17.47
일평균 흡연량(개비)	min=1; max=20	12.25	5.57

〈표 4-64〉 북한이탈주민의 흡연 특성

요인	구분	빈도(회)	백분율(%)
흡연기간(년)	비흡연	276	92.00
	2	1	0.33
	3	1	0.33
	5	1	0.33
	10	1	0.33
	20	3	1.00
	30	4	1.33
	35	2	0.67
	36	1	0.33
	40	2	0.67
	50	6	2.00
	60	2	0.67
일평균 흡연량(개비)	비흡연	276	92.00
	1	1	0.33
	5	2	0.67
	6	1	0.33
	7	1	0.33
	8	1	0.33
	10	7	2.33
	15	5	1.67
	17	1	0.33
	20	5	1.67
합계		300	100.00

2) 음주

북한이탈주민의 음주 행태를 평가하기 위하여 AUDIT-C (Alcohol Use Disorders Identification Test Consumption)를 이용하였다.

AUDIT-C 1번 문항(최근 1년간 얼마나 자주 술을 마십니까?)에 따라서는 ‘전혀안마심’이 200명(66.67%)으로 가장 많았으며, 그다음으로는 ‘월1회미만’이 43명(14.33%), ‘월2~4회’가 33명(11.00%), ‘주2~3회’가 13명(4.33%), ‘주4회이상’이 11명(3.67%) 순으로 각각 나타났다.

AUDIT-C 1번 문항에 음주를 한다고 응답(‘전혀안마심’을 제외한 나머지 응답)한 100명을 대상으로 2번 문항(술을 마시면 한 번에 몇 잔 정도 마십니까?)에 따라서는 ‘한두잔’이 54명(54.00%)으로 가장 많았으며, 그다음으로는 ‘3~4잔’이 22명(22.00%), ‘5~6잔’이 10명(10.00%), ‘7~9잔’이 8명(8.00%), ‘10잔이상’이 6명(6.00%) 순으로 각각 나타났다.

AUDIT-C 1번 문항에 음주를 한다고 응답(‘전혀안마심’을 제외한 나머지 응답)한 100명을 대상으로 3번 문항(한 번에 소주 1병 이상 혹은 맥주 4병 이상 마시는 경우는 얼마나 자주 있습니까?)에 따라서는 ‘전혀없음’이 43명(43.00%)으로 가장 많았으며, 그다음으로는 ‘월1회미만’이 24명(24.00%), ‘월1회’가 11명(11.00%), ‘주1회’가 18명(18.00%), ‘거의매일’이 4명(4.00%) 순으로 각각 나타났다.

〈표 4-65〉 북한이탈주민의 음주 특성

요인	구분	빈도(회)	백분율(%)
1. 최근 1년간 얼마나 자주 술을 마십니까?	전혀 안마심	200	66.67
	월1회 미만	43	14.33
	월2~4회	33	11.00
	주2~3회	13	4.33
	주4회이상	11	3.67
	합계	300	100.00
2. 술을 마시면 한 번에 몇 잔 정도 마십니까?	한두잔	54	54.00
	3~4잔	22	22.00
	5~6잔	10	10.00
	7~9잔	8	8.00
	10잔이상	6	6.00
	합계(음주자 중)	100	100.00
3. 한 번에 소주 1병 이상 혹은 맥주 4병 이상 마시는 경우는 얼마나 자주 있습니까?	전혀 없음	43	43.00
	월1회 미만	24	24.00
	월1회	11	11.00
	주1회	18	18.00
	거의매일	4	4.00
	합계(음주자 중)	100	100.00

AUDIT-C 점수는 0에서 12점의 범위를 가진다. 남녀별로 임상군 및 비임상군의 분포 특성을 살펴본 결과이다. 남자의 경우는 총점이 4점 이상인 경우 임상군으로 분류되며, 여자의 경우는 총점이 3점 이상인 경우 임상군으로 분류된다. 남자 55명 중 30명(54.55%)이 비임상군이며, 25명(45.45%)이 임상군으로 나타났다. 여자의 경우는 245명 중 219명(89.39)이 비임상군이며, 26명(10.61%)이 임상군으로 나타났다.

〈표 4-66〉 북한이탈주민의 AUDIT-C 점수에 따른 임상군 분포 특성

요인	구분	빈도(회)	백분율(%)
AUDIT-C 임상군(남자)	임상군	25	45.45
	비임상군	30	54.55
AUDIT-C 임상군(여자)	임상군	26	10.61
	비임상군	219	89.39
합계		300	100.00

4. 인권침해 트라우마가 정신건강, 삶의 질 및 취업에 미치는 영향

가. 탈북 및 남한입국과정과 정착기간에 따른 차이

북한에서 탈북하여 남한으로 직행한 북한이탈주민과 중국을 경유하여 남한에 들어온 북한이탈주민 간의 정신과적 척도에 대한 차이를 분석하기 위하여 집단별 분산의 분포에 따라 Student's t-test 또는 Welch's t-test를 선택적으로 수행하였다. 분석 대상 척도는 건강질문지-9(Patient Health Questionnaire, PHQ-9), 자살경향성 척도(K-MINI), 외상 후 스트레스 장애 체크리스트(PCL-5 with a item of PCL-IV) 및 불면증 심각도 척도(Insomnia Severity Index, ISI)이다. 통계적 유의성의 판별은 양측 검정을 기준으로 95% 신뢰수준에서 검정하였다.

1) 외상 후 스트레스장애

남한입국과정 차이에 따른 북한이탈주민의 외상 후 스트레스장애 특성을 알아보기 위하여 외상 후 스트레스장애 체크리스트(PCL-5 with a item of PCL-IV) 총점에 차이가 있는지를 분석한 결과, 중국 경유자의 총점 평균은 38.92±23.19점으로 남한 직행자의 총점 평균인 30.48±21.69점에 비해 유의하게 약 28% 높게 나타났다. 이는 중국 경유자가 남한 직행자에 비해 더 높은 외상 후 스트레스장애 수준을 나타냄을 시사한다.

〈표 4-67〉 남한입국과정 차이에 따른 북한이탈주민의 외상 후 스트레스장애 특성

구분	평균	표준편차	95% 신뢰구간		유의확률
남한 직행	30.45	21.69	26.36	34.55	.002
중국 경유*	38.92	23.19	35.52	42.32	

*중국을 통해서 넘어온 수준이 아닌 1년이상 중국에 거주했을 경우를 의미

2) 우울

남한입국과정 차이에 따른 북한이탈주민의 우울 특성을 알아보기 위하여 건강질문지-9 (Patient Health Questionnaire, PHQ-9) 총점에 차이가 있는지를 분석한 결과, 중국 경유자의 총점 평균은 13.88±8.39점으로 남한 직행자의 총점 평균인 11.46±8.02점에 비해 유의하게 약 21% 높게 나타났다. 이는 중국 경유자가 남한 직행자에 비해 더 높은 우울 수준을 나타냄을 시사한다.

〈표 4-68〉 남한입국과정 차이에 따른 북한이탈주민의 우울 특성

구분	평균	표준편차	95% 신뢰구간		유의확률
남한 직행	11.46	8.02	9.95	12.98	.016
중국 경유*	13.88	8.39	12.65	15.11	

*중국을 통해서 넘어온 수준이 아닌 1년이상 중국에 거주했을 경우를 의미

3) 자살경향성

남한입국과정 차이에 따른 북한이탈주민의 자살경향성 특성을 알아보기 위하여 자살경향성 척도(K-MINI) 총점에 차이가 있는지를 분석한 결과, 중국 경유자의 총점 평균은 8.48±10.96점으로 남한 직행자의 총점 평균인 4.68±8.40점에 비해 유의하게 약 2배 높게 나타났다. 이는 중국 경유자가 남한 직행자에 비해 더 높은 자살경향성 수준을 나타냄을 시사한다.

〈표 4-69〉 남한입국과정 차이에 따른 북한이탈주민의 자살경향성 특성

구분	평균	표준편차	95% 신뢰구간		유의확률
남한 직행	4.68	8.40	3.09	6.27	.001
중국 경유*	8.48	10.96	6.87	10.08	

*중국을 통해서 넘어온 수준이 아닌 1년이상 중국에 거주했을 경우를 의미

4) 불면증

남한입국과정 차이에 따른 북한이탈주민의 불면증 특성을 알아보기 위하여 불면증 심각도 척도(Insomnia Severity Index, ISI) 총점에 차이가 있는지를 분석한 결과, 중국 경유자의 총점 평균은 14.80±8.20점으로 남한 직행자의 총점 평균인 13.36±7.85점에 비해 약 11% 높게 나타났으나, 통계적으로 유의한 수준의 차이는 아니었다.

<표 4-70> 남한입국과정 차이에 따른 북한이탈주민의 불면증 심각도 특성

구분	평균	표준편차	95% 신뢰구간		유의확률
남한 직행	13.36	7.85	11.88	14.85	.145
중국 경유	14.80	8.20	13.60	16.00	

*중국을 통해서 넘어온 수준이 아닌 1년이상 중국에 거주했을 경우를 의미

5) 정착기간과 정신건강문제 간의 연관성

남한 정착기간과 정신건강문제 정도의 연관성을 알아보기 위하여 Spearman's rank correlation test를 수행하였다. 이들 요인은 정규분포하지 않는 가산자료의 형태이고 여부로 나누어지지 않으므로, 양자 간의 연관성을 평가하기 위하여 비모수 검정이 적합하다고 판단하였다. 분석 결과 우울(PHQ-9), 자살경향성(K-MINI), 외상 후 스트레스장애(PCL-5) 및 불면증(ISI)의 모든 정신건강문제 정도는 정착기간과 부적인 상관관계를 나타냈다. 이는 남한 정착기간이 길수록 정신건강문제의 심각성이 낮을 가능성이 있음을 의미한다. 다만 그 정도가 통계적으로 유의한 수준에 도달하지는 못하였다.

<표 4-71> 정착기간과 정신건강문제 간의 연관성 특성

구분	계수	유의확률
우울(PHQ-9)	-0.024	.678
자살경향성(K-MINI)	-0.002	.974
외상 후 스트레스장애(PCL-5)	-0.044	.449
불면증(ISI)	-0.028	.626

6) 삶의 질

남한입국과정 차이에 따른 북한이탈주민의 건강관련 삶의 질 특성을 알아보기 위하여 SF-8(Short Form-8 Health survey) 총점에 차이가 있는지를 분석한 결과, 중국 경유자의 총점 평균은 27.49±8.05점으로 남한 직행자의 총점 평균인 25.87±7.87점에 비해 약 6% 높게 나타났다. SF-8은 척도의 특성 상 점수가 높을수록 장애가 높음을 의미하는 것으로 삶의 질은 낮아지는 것이다. 이는 중국을 경유하여 입국한 경우 보다 낮은 삶의 질 수준을 나타낼 수 있음을 시사한다. 다만 집단 간 차이가 통계적으로 유의할 확률(p=.094)은 설정된 유의수준(p<.050) 보다는 소폭 높게 나타났으므로 한계적으로 유의(marginally significant)한 수준에서 해석하여야 한다.

<표 4-72> 남한입국과정 차이에 따른 북한이탈주민의 삶의 질 특성

구분	평균**	표준편차	95% 신뢰구간		유의확률
남한 직행	25.87	7.87	24.39	27.36	.094
중국 경유*	27.49	8.05	26.31	28.67	

*중국을 통해서 넘어온 수준이 아닌 1년이상 중국에 거주했을 경우를 의미
**점수가 높을수록 장애가 높고 삶의 질이 낮음을 의미

7) 취업

남한입국과정과 취업여부 간의 연관성을 알아보기 위하여 피어슨의 카이제곱 검정을 수행하였다. 분석 결과, 남한 직행자 110명 중 취업자는 43명(39.09%)이었다. 한편 중국 경유자 181명 중 취업자는 101명(55.80%)으로 남한 직행자에 비해 유의하게 취업자에 속한 비율이 더 많은 것으로 나타났다.

<표 4-73> 남한입국과정 차이에 따른 북한이탈주민의 취업 특성

구분	취업		미취업		합계		유의확률
	빈도(회)	백분율(%)	빈도(회)	백분율(%)	빈도(회)	백분율(%)	
직행	43	39.09	67	60.91	110	100	.006
중국*	101	55.80	80	44.20	181	100	
합계	144	49.48	147	50.52	291	100	

*중국을 통해서 넘어온 수준이 아닌 1년이상 중국에 거주했을 경우를 의미

나. 인권침해 경험에 따른 차이

1) 인권침해 경험에 따른 외상 후 스트레스 증상

북한이탈주민이 경험한 인권침해 수준에 따라 외상 후 스트레스 증상 정도에 대한 차이를 분석하기 위하여 집단별 분산의 분포에 따라 Student's t-test 또는 Welch's t-test를 선택적으로 수행하였다. 인권침해 수준은 인권침해 조사표를 이용하여 측정하였다. 이는 북한이탈주민의 인권침해에 대한 본인 또는 가족의 겪음, 주변 사람들이 겪는 것을 목격 하였거나, 소문을 들은 것에 대한 경험 여부를 평가하기 위한 것으로, 총 13개의 다양한 인권침해 사건에 대한 경험을 점검하는 문항으로 구성되어 있으며, 각 사건 특성별 필요에 따라 북한 생활 당시와 탈북과정 중 및 남한 입국 후의 세 시점에서 각각 회상 평가하였다. 응답은 '내가 직접 당하였다', '가족들이 당하는 것을 경험하였다', '주변사람들이 당하는 것을 목격하였다', '소문을 들었다', '전혀 겪거나 들은 바 없다' 중 가장 강한 강도의 경험에 답하도록 하였다. 외상 후 스트레스 증상 수준은 외상 후 스트레스 장애 체크리스트(PCL-5 with a item of PCL-IV) 도구를 이용하였으며, 통계적 유의성의 판별은 양측 검정을 기준으로 95% 신뢰수준에서 검정하였다.

가) 심한 굶주림 또는 질병 경험

북한에서 심한 굶주림 또는 질병과 관련된 경험수준의 차이에 따른 외상 후 스트레스 증상 정도의 특성을 알아보기 위하여 외상 후 스트레스 장애 체크리스트(PCL-5 with a item of PCL-IV) 총점에 차이가 있는지를 분석한 결과, '내가 직접 당하였다', '가족들이 당하는 것을 경험하였다' 또는 '주변사람들이 당하는 것을 목격하였다'라고 응답한 집단의 PCL-5 총점 평균은 36.04±22.86점으로 '소문을 들었다' 또는 '전혀 겪거나 들은 바 없다'라고 응답한 집단의 PCL-5 총점 평균인 25.76±21.29점에 비해 유의하게 약 40% 높게 나타났다. 이는 북한에서 심한 굶주림 또는 질병을 경험한 경우 보다 높은 외상 후 스트레스 증상 수준을 나타낼 수 있음을 시사한다.

〈표 4-74〉 심한 굶주림 또는 질병경험 차이에 따른 북한이탈주민의 외상 후 스트레스 증상

구분	평균	표준편차	95% 신뢰구간		유의확률
‘소문을 들음’ 이하	25.76	21.29	16.07	35.45	.047
‘주변 사람들이 당하는 것을 목격’ 이상	36.04	22.86	33.29	38.79	

나) 정치범수용소의 잔혹성

북한에서 정치범수용소의 잔혹성과 관련된 경험에 대한 수준 차이에 따른 외상 후 스트레스 증상 정도의 특성을 알아보기 위하여 외상 후 스트레스 장애 체크리스트(PCL-5 with a item of PCL-IV) 총점에 차이가 있는지를 분석한 결과, ‘내가 직접 당하였다’, ‘가족들이 당하는 것을 경험하였다’ 또는 ‘주변사람들이 당하는 것을 목격하였다’라고 응답한 집단의 PCL-5 총점 평균은 40.05±22.35점으로 ‘소문을 들었다’ 또는 ‘전혀 겪거나 들은 바 없다’라고 응답한 집단의 PCL-5 총점 평균인 34.35±22.98점에 비해 약 17% 높게 나타났다. 이는 북한에서 심한 굶주림 또는 질병을 경험한 경우 보다 높은 외상 후 스트레스 증상 수준을 나타낼 수 있음을 시사한다. 다만 집단 간 차이가 통계적으로 유의할 확률(p=.058)은 설정된 유의수준(p<.050) 보다는 소폭 높게 나타났으므로 한계적으로 유의(marginally significant)한 수준에서 해석하여야 한다.

〈표 4-75〉 정치범수용소의 잔혹성 경험 차이에 따른 북한이탈주민의 외상 후 스트레스 증상

구분	평균	표준편차	95% 신뢰구간		유의확률
‘소문을 들음’ 이하	34.35	22.98	31.31	37.40	.058
‘주변 사람들이 당하는 것을 목격’ 이상	40.05	22.35	35.05	45.06	

다) 공개처형

북한에서 공개처형 관련 경험에 대한 차이에 따른 외상 후 스트레스 증상 정도의 특성을 알아보기 위하여 외상 후 스트레스 장애 체크리스트(PCL-5 with a item of PCL-IV) 총점에 차이가 있는지를 분석한 결과, ‘내가 직접 당하였다’, ‘가족들이 당하는 것을 경험하였다’ 또는 ‘주변사람들이 당하는 것을 목격하였다’라고 응답한 집단의 PCL-5 총점 평균은 38.38±22.78점으로 ‘소문을 들었다’ 또는 ‘전혀 겪거나 들은 바 없다’라고 응답한 집단의 PCL-5 총점 평균인 24.33±20.00점에 비해 유의하게 약 58% 높게 나타났다. 이는 북한에서 공개처형과 관련된 경험을 한 경우 보다 높은 외상 후 스트레스 증상 수준을 나타낼 수 있음을 시사한다.

〈표 4-76〉 공개처형 관련 경험 차이에 따른 북한이탈주민의 외상 후 스트레스 증상

구분	평균	표준편차	95% 신뢰구간		유의확률
‘소문을 들음’ 이하	24.33	20.00	18.88	29.79	<.001
‘주변 사람들이 당하는 것을 목격’ 이상	38.38	22.78	35.52	41.24	

라) 탈북으로 인한 처벌

북한에서 강제송환 등으로 인해 탈북으로 인한 처벌과 관련 경험에 대한 차이에 따른 외상 후 스트레스 증상 정도의 특성을 알아보기 위하여 외상 후 스트레스 장애 체크리스트(PCL-5 with a item of PCL-IV) 총점에 차이가 있는지를 분석한 결과, ‘내가 직접 당하였다’, ‘가족들이 당하는 것을 경험하였다’ 또는 ‘주변사람들이 당하는 것을 목격하였다’라고 응답한 집단의 PCL-5 총점 평균은 40.18±22.80점으로 ‘소문을 들었다’ 또는 ‘전혀 겪거나 들은 바 없다’라고 응답한 집단의 PCL-5 총점 평균인 30.63±22.03점에 비해 유의하게 약 31% 높게 나타났다. 이는 북한에서 탈북으로 인한 처벌과 관련된 경험을 한 경우 보다 높은 외상 후 스트레스 증상 수준을 나타낼 수 있음을 시사한다.

<표 4-77> 탈북으로 인한 처벌 관련 경험 차이에 따른 북한이탈주민의 외상 후 스트레스 증상

구분	평균	표준편차	95% 신뢰구간		유의확률
'소문을 들음' 이하	30.63	22.03	26.90	34.37	.001
'주변 사람들이 당하는 것을 목격' 이상	40.18	22.80	36.67	43.70	

마) 여성에 대한 성폭력

여성에 대한 성폭력 관련 경험은 시점에 따라 북한 거주 당시와 탈북과정에서의 경험으로 각각 분석하였다.

북한에서 여성에 대한 성폭력 관련 경험에 대한 차이에 따른 외상 후 스트레스 증상 정도의 특성을 알아보기 위하여 외상 후 스트레스 장애 체크리스트(PCL-5 with a item of PCL-IV) 총점에 차이가 있는지를 분석한 결과, '내가 직접 당하였다', '가족들이 당하는 것을 경험하였다' 또는 '주변사람들이 당하는 것을 목격하였다'라고 응답한 집단의 PCL-5 총점 평균은 47.17±21.13점으로 '소문을 들었다' 또는 '전혀 겪거나 들은 바 없다'라고 응답한 집단의 PCL-5 총점 평균인 33.09±22.82점에 비해 유의하게 약 43% 높게 나타났다. 이는 북한에서 여성에 대한 성폭력과 관련된 경험을 한 경우 보다 높은 외상 후 스트레스 증상 수준을 나타낼 수 있음을 시사한다.

<표 4-78> 북한에서 여성에 대한 성폭력관련 경험 차이에 따른 북한이탈주민의 외상 후 스트레스 증상

구분	평균	표준편차	95% 신뢰구간		유의확률
'소문을 들음' 이하	33.09	22.82	29.88	36.29	.001
'주변 사람들이 당하는 것을 목격' 이상	47.17	21.13	41.40	52.93	

탈북기간 중에 여성에 대한 성폭력 관련 경험에 대한 차이에 따른 외상 후 스트레스 증상 정도의 특성을 알아보기 위하여 외상 후 스트레스 장애 체크리스트(PCL-5 with a item of PCL-IV) 총점에 차이가 있는지를 분석한 결과, '내가 직접 당하였다', '가족들이 당하는 것을 경험하였다' 또는 '주변사람들이 당하는 것을 목격하였다'라고 응답한 집단의 PCL-5 총점 평균은 37.84±19.96점으로 '소문을 들었

다’ 또는 ‘전혀 겪거나 들은 바 없다’라고 응답한 집단의 PCL-5 총점 평균인 32.42±23.17점에 비해 약 17% 높게 나타났으나, 통계적으로 유의한 수준의 차이는 아니었다.

〈표 4-79〉 탈북과정 중 여성에 대한 성폭력관련 경험 차이에 따른 북한이탈주민의 외상 후 스트레스 증상

구분	평균	표준편차	95% 신뢰구간		유의확률
‘소문을 들음’ 이하	32.42	23.17	28.35	36.49	.276
‘주변 사람들이 당하는 것을 목격’ 이상	37.84	19.96	29.60	46.08	

바) 여성 또는 아동 인신매매

탈북기간 중에 여성 또는 아동 인신매매와 관련된 경험의 차이에 따른 외상 후 스트레스 증상 정도의 특성을 알아보기 위하여 외상 후 스트레스 장애 체크리스트(PCL-5 with a item of PCL-IV) 총점에 차이가 있는지를 분석한 결과, ‘내가 직접 당하였다’, ‘가족들이 당하는 것을 경험하였다’ 또는 ‘주변사람들이 당하는 것을 목격하였다’라고 응답한 집단의 PCL-5 총점 평균은 41.78±23.07점으로 ‘소문을 들었다’ 또는 ‘전혀 겪거나 들은 바 없다’라고 응답한 집단의 PCL-5 총점 평균인 31.32±21.80점에 비해 유의하게 약 34% 높게 나타났다. 이는 탈북기간 중 여성 또는 아동 인신매매와 관련된 경험을 한 경우 보다 높은 외상 후 스트레스 증상 수준을 나타낼 수 있음을 시사한다.

〈표 4-80〉 탈북과정 중 여성 또는 아동 인신매매 관련 경험 차이에 따른 북한이탈주민의 외상 후 스트레스 증상

구분	평균	표준편차	95% 신뢰구간		유의확률
‘소문을 들음’ 이하	31.32	21.80	28.02	34.62	.001
‘주변 사람들이 당하는 것을 목격’ 이상	41.78	23.07	37.77	45.78	

2) 인권침해 경험에 따른 삶의 질

인권침해 경험의 차이에 따른 북한이탈주민의 건강관련 삶의 질 특성을 알아보기 위하여 SF-8(Short Form-8 Health survey) 총점에 차이가 있는지를 분석하였다. 척도의 특성 상 점수가 높은 것은 장애 수준이 높고 삶의 질은 낮음을 의미한다. 그 결과, 공개처형에 따라서는‘내가 직접 당하였다’, ‘가족들이 당하는 것을 경험하였다’ 또는 ‘주변사람들이 당하는 것을 목격하였다’라고 응답한 집단의 SF-8 총점 평균은 27.56±7.92점으로 ‘소문을 들었다’ 또는 ‘전혀 겪거나 들은 바 없다’라고 응답한 집단의 SF-8 총점 평균인 23.20±7.90점에 비해 유의하게 약 19% 높게 나타났다. 탈북으로 인한 처벌에 따라서는‘내가 직접 당하였다’, ‘가족들이 당하는 것을 경험하였다’ 또는 ‘주변사람들이 당하는 것을 목격하였다’라고 응답한 집단의 SF-8 총점 평균은 28.24±7.60점으로 ‘소문을 들었다’ 또는 ‘전혀 겪거나 들은 바 없다’라고 응답한 집단의 SF-8 총점 평균인 25.01±8.32점에 비해 유의하게 약 13% 높게 나타났다. 북한에서의 여성에 대한 성폭력에 따라서는‘내가 직접 당하였다’, ‘가족들이 당하는 것을 경험하였다’ 또는 ‘주변사람들이 당하는 것을 목격하였다’라고 응답한 집단의 SF-8 총점 평균은 30.02±6.42점으로 ‘소문을 들었다’ 또는 ‘전혀 겪거나 들은 바 없다’라고 응답한 집단의 SF-8 총점 평균인 26.35±8.57점에 비해 유의하게 약 14% 높게 나타났다. 탈북 중 여성 또는 아동 인신매매에 따라서는‘내가 직접 당하였다’, ‘가족들이 당하는 것을 경험하였다’ 또는 ‘주변사람들이 당하는 것을 목격하였다’라고 응답한 집단의 SF-8 총점 평균은 28.69±7.70점으로 ‘소문을 들었다’ 또는 ‘전혀 겪거나 들은 바 없다’라고 응답한 집단의 SF-8 총점 평균인 25.31±8.09점에 비해 유의하게 약 13% 높게 나타났다. 이는 해당 트라우마 경험자가 삶의 질이 유의하게 낮음을 의미한다. 한편 심한 굶주림 또는 질병 경험, 정치범수용소의 잔혹성, 여성에 대한 성폭력(탈북중)에 따라서는 유의한 삶의 질의 차이를 보이지 않았다.

〈표 4-81〉 인권침해경험 경험 차이에 따른 북한이탈주민의 삶의 질 특성

요인	구분	평균*	표준편차	95% 신뢰구간		유의확률
심한 굶주림 또는 질병 경험	소문들음이하	24.48	9.18	20.30	28.66	.202
	목격이상	26.83	8.03	25.86	27.79	
정치범수용소의 잔혹성	소문들음이하	26.33	8.31	25.23	27.43	.113
	목격이상	28.01	7.33	26.37	29.65	
공개처형	소문들음이하	23.20	7.90	21.05	25.36	<.001
	목격이상	27.56	7.92	26.56	28.55	
탈북으로 인한 처벌	소문들음이하	25.01	8.32	23.60	26.42	.001
	목격이상	28.24	7.60	27.07	29.41	
여성에 대한 성폭력(북한에서)	소문들음이하	26.35	8.57	25.14	27.55	.001
	목격이상	30.02	6.42	28.27	31.77	
여성에 대한 성폭력(탈북중)	소문들음이하	26.24	8.61	24.73	27.76	.729
	목격이상	26.88	6.97	24.00	29.76	
여성 또는 아동 인신매매 (탈북중)	소문들음이하	25.31	8.09	24.08	26.53	<.001
	목격이상	28.69	7.70	27.36	30.03	

*점수가 높을수록 장애가 높고 삶의 질이 낮음을 의미

3) 인권침해경험에 따른 취업 여부

인권침해경험과 취업여부 간의 연관성을 알아보기 위하여 피어슨의 카이제곱 검정을 수행하였다. 분석 결과, 탈북 중 여성 또는 아동 인신매매를 '내가 직접 당하였다', '가족들이 당하는 것을 경험하였다' 또는 '주변사람들이 당하는 것을 목격하였다'라고 응답한 집단 130명 중 취업자는 79명(60.77%)이었다. 한편 '소문을 들었다' 또는 '전혀 겪거나 들은 바 없다'라고 응답한 집단 170명 중 취업자는 68명(40.00%)로 '목격 이상' 집단이 '소문 들음 이하' 집단에 비해 유의하게 취업자에 속한 비율이 더 많은 것으로 나타났다. 심한 굶주림 또는 질병 경험, 정치범수용소의 잔혹성. 공개처형, 탈북으로 인한 처벌, 북한에서의 여성에 대한 성폭력, 탈북중 여성에 대한 성폭력에 따라서는 유의한 연관성을 나타내지 않았다.

〈표 4-82〉 인권침해경험 경험 차이에 따른 북한이탈주민의 취업여부 특성

요인	구분	취업		미취업		합계		유의확률
		빈도(회)	백분율(%)	빈도(회)	백분율(%)	빈도(회)	백분율(%)	
심한 굶주림 또는 질병 경험	소문들음이하	8	38.10	13	61.90	21	100	.368
	목격이상	133	49.63	135	50.37	268	100	
정치범수용소의 잔혹성	소문들음이하	109	49.32	112	50.68	221	100	.852
	목격이상	38	48.10	41	51.90	79	100	
공개처형	소문들음이하	21	38.89	33	61.11	54	100	.101
	목격이상	126	51.22	120	48.78	246	100	
탈북으로 인한 처벌	소문들음이하	59	43.38	77	56.62	136	100	.076
	목격이상	88	53.66	76	46.34	164	100	
여성에 대한 성폭력(북한에서)	소문들음이하	90	45.69	107	54.31	197	100	.127
	목격이상	31	57.41	23	42.59	54	100	
여성에 대한 성폭력(탈북중)	소문들음이하	61	48.03	66	51.97	127	100	.190
	목격이상	16	64.00	9	36.00	25	100	
여성 또는 아동 인신매매 (탈북중)	소문들음이하	68	40.00	102	60.00	170	100	<.001
	목격이상	79	60.77	51	39.23	130	100	
합계		147	49.00	153	51.00	300	100	

다. 트라우마 경험에 따른 차이

북한이탈주민이 북한거주 당시, 탈북과정 중, 남한 입국 후의 시점별로 각각 경험한 트라우마 수준에 따라 외상 후 스트레스 증상 정도에 대한 차이를 분석하기 위하여 집단별 분산의 분포에 따라 Student's t-test 또는 Welch's t-test를 선택적으로 수행하였다.

북한이탈주민의 트라우마 경험 여부를 평가하기 위하여 생활사건점검표 (Life Events Checklist for DSM-5, LEC-5)를 이용하였다. 점검문항은 자연재난 및 화재 또는 폭발, 교통사고, 직장이나 집 또는 여가 활동 중 심각한 사고, 독성 물질에 노출, 신체폭력, 무기로 공격당함, 성폭력, 기타 원하지 않거나 불편한 성적 경험, 전투나 전쟁터에 노출, 감금, 목숨이 좌우될 정도의 질병이나 부상, 심각한 인간적 고난, 급작스러운 변사, 급작스러운 사고사, 나 자신 때문에 발생했던 다른 사람의 심각한 부상, 상해 또는 사망, 그 밖의 매우 심각한 스트레스 사건이나 경험과 같은 총 17개의 다양한 스트레스 사건으로 구성되어 있으며, 북한 생활 당시와 탈북과정 중 및 남한 입국 후의 세 시점에서 각각 회상 평가하였다. 외상 후 스트레스 증상 정도는 외상 후 스트레스 장애 체크리스트(PCL-5 with a item of PCL-IV) 도구를 이용하였으며, 통계적 유의성의 판별은 양측 검정을 기준으로 95% 신뢰수준에서 검정하였다.

1) 북한에서 트라우마 경험에 따른 차이

북한이탈주민이 북한 거주 당시 경험한 트라우마 여부에 따른 외상 후 스트레스 증상 특성을 알아보기 위하여 외상 후 스트레스 장애 체크리스트(PCL-5 with a item of PCL-IV) 총점에 차이가 있는지를 분석하고, 이를 유의하게 차이가 컸던 순으로 살펴보면 다음과 같다.

나 자신 때문에 발생했던 다른 사람의 심각한 부상, 상해 또는 사망을 목격한 대상자의 총점 평균은 68.80±8.98점으로 비경험자의 총점 평균인 35.29±22.68점에 비해 유의하게 약 2배(95%)로 높아 가장 차이가 컸다. 그다음으로는 공격, 가격(손, 주먹, 몽둥이로 공격), 따귀를 맞음, 또는 발로 채이거나 두들겨 맞는 등의 신체폭력을 직접 겪은 대상자의 총점 평균은 41.64±22.26점으로 비경험자의 총점 평균인 30.86±22.36점에 비해 유의하게 약 35% 높게 나타났다. 총이나 칼에 맞거나, 칼, 총, 폭탄으로 위협 당하는 등과 같이 무기에 의한 공격을 직접 겪은 대상자의 총점 평균은 46.00±21.67점으로 비경험자의 총점 평균인 34.43±22.77점에 비해 유의하게

약 34% 높게 나타났다. 목숨이 좌우될 정도의 질병이나 부상을 직접 겪은 대상자의 총점 평균은 44.23±22.24점으로 비경험자의 총점 평균인 33.06±22.50점에 비해 유의하게 약 34% 높게 나타났다. 납치, 유괴, 인질 및 전쟁 포로 등으로 인한 감금을 직접 겪은 대상자의 총점 평균은 42.88±21.83점으로 비경험자의 총점 평균인 33.64±22.85점에 비해 유의하게 약 27% 높게 나타났다. 홍수나 태풍, 폭풍, 지진과 같은 자연재난을 직접 겪은 대상자의 총점 평균은 40.35±22.95 점으로 비경험자의 총점 평균인 32.28±22.32점에 비해 유의하게 약 25% 높게 나타났다. 타인이 살인이나 자살 등의 급작스러운 변사를 당하는 것을 목격한 대상자의 총점 평균은 40.28±22.59점으로 비경험자의 총점 평균인 33.47±22.80점에 비해 유의하게 약 20% 높게 나타났다. 이는 해당 트라우마 경험자가 비경험자에 비해 더 높은 외상 후 스트레스 증상 수준을 나타냄을 시사한다.

반면에, 화재 또는 폭발, 교통사고, 직장이나 집 또는 여가 활동 중 심각한 사고, 독성 물질에 노출, 기타 원하지 않거나 불편한 성적 경험, 전투나 전쟁터에 노출, 심각한 인간적 고난, 급작스러운 사고사 목격에 따라서는 트라우마 경험 여부에 따른 외상 후 스트레스 장애 체크리스트(PCL-5 with a item of PCL-IV) 총점이 통계적으로 유의한 수준의 차이를 나타내지 않았다.

<표 4-83> 북한에서 겪은 트라우마 경험 차이에 따른 북한이탈주민의 외상 후 스트레스 증상

요인	트라우마 경험	평균	표준편차	95% 신뢰구간		유의확률
1. 자연재난	아니오	32.28	22.32	28.87	35.69	.002
	예	40.35	22.95	36.41	44.28	
2. 화재 또는 폭발	아니오	35.09	22.83	32.30	37.88	.141
	예	40.83	23.17	33.42	48.23	
3. 교통사고	아니오	35.85	22.92	33.07	38.63	.992
	예	35.89	23.23	28.03	43.75	
4. 직장, 집, 또는 여가 활동 중 심각한 사고	아니오	35.45	22.94	32.77	38.14	.233
	예	42.11	22.33	31.01	53.21	
5. 독성 물질에 노출	아니오	35.60	22.91	32.95	38.24	.273
	예	44.11	23.01	26.43	61.80	
6. 신체폭력	아니오	30.86	22.36	27.38	34.34	<.001
	예	41.64	22.26	37.91	45.37	
7. 무기로 공격당함	아니오	34.43	22.77	31.66	37.19	.004
	예	46.00	21.67	38.78	53.22	
8. 성폭력	아니오	34.93	22.80	32.27	37.60	.004
	예	51.18	19.78	41.01	61.35	
9. 기타 원하지 않거나 불편한 성적 경험	아니오	35.32	23.03	32.63	38.01	.009
	예	45.25	19.16	35.04	55.46	
10. 전투나 전쟁터에 노출	아니오	36.10	22.92	33.44	38.76	.367
	예	30.00	23.13	15.30	44.70	
11. 감금	아니오	33.64	22.85	30.65	36.62	.003
	예	42.88	21.83	37.74	48.01	
12. 목숨이 좌우될 정도의 질병이나 부상	아니오	33.06	22.50	30.11	36.02	<.001
	예	44.23	22.24	39.11	49.34	
13. 심각한 인간적 고난	아니오	31.95	23.21	27.53	36.38	.027
	예	38.05	22.52	34.84	41.25	
14. 급작스러운 변사	아니오	33.47	22.80	30.25	36.69	.001
	예	40.28	22.59	35.91	44.65	
15. 급작스러운 사고사	아니오	35.43	22.83	32.26	38.61	.652
	예	36.71	23.19	32.08	41.33	
16. 나 자신 때문에 발생했던 다른 사람의 심각한 부상, 상해 또는 사망	아니오	35.29	22.68	32.70	37.89	<.001
	예	68.80	8.98	57.65	79.95	

트라우마 경험에 따른 삶의 질 차이는 다음과 같다. 북한이탈주민이 북한 거주 당시 경험한 트라우마 여부에 따른 북한이탈주민의 건강관련 삶의 질 특성을 알아보기 위하여 SF-8(Short Form-8 Health survey) 총점에 차이가 있는지를 분석하고, 이를 유의하게 차이가 컸던 순으로 살펴보면 다음과 같다. 척도의 특성 상 점수가 높을수록 장애 수준이 높고 삶의 질이 낮은 것을 의미한다. 나 자신 때문에 발생했던 다른 사람의 심각한 부상, 상해 또는 사망을 목격한 대상자의 총점 평균은 37.40±2.19점으로 비경험자의 총점 평균인 26.59±8.03점에 비해 유의하게 약 41% 높아 가장 차이가 컸다. 그다음으로는 기타 원하지 않거나 불편한 성적 경험을 직접 겪은 대상자의 총점 평균은 31.69±6.79점으로 비경험자의 총점 평균인 26.50±8.07점에 비해 유의하게 약 20% 높게 나타났다. 성폭력을 직접 겪은 대상자의 총점 평균은 31.53±6.15점으로 비경험자의 총점 평균인 26.49±8.10점에 비해 유의하게 약 19% 높게 나타났다. 총이나 칼에 맞거나, 칼, 총, 폭탄으로 위협 당하는 등과 같이 무기에 의한 공격을 직접 겪은 대상자의 총점 평균은 30.24±7.38점으로 비경험자의 총점 평균인 26.29±8.07점에 비해 유의하게 약 15% 높게 나타났다. 타인이 살인이나 자살 등의 급작스러운 변사를 당하는 것을 목격한 대상자의 총점 평균은 29.07±6.87점으로 비경험자의 총점 평균인 25.54±8.43점에 비해 유의하게 약 14% 높게 나타났다. 납치, 유괴, 인질 및 전쟁 포로 등으로 인한 감금을 직접 겪은 대상자의 총점 평균은 29.36±8.54점으로 비경험자의 총점 평균인 25.96±7.77점에 비해 유의하게 약 13% 높게 나타났다. 공격, 가격(손, 주먹, 몽둥이로 공격), 따귀를 맞음, 또는 발로 채이거나 두들겨 맞는 등의 신체폭력을 직접 겪은 대상자의 총점 평균은 28.46±7.77점으로 비경험자의 총점 평균인 25.32±8.09점에 비해 유의하게 약 12% 높게 나타났다. 홍수나 태풍, 폭풍, 지진과 같은 자연재난을 직접 겪은 대상자의 총점 평균은 28.39±7.83점으로 비경험자의 총점 평균인 25.49±8.07점에 비해 유의하게 약 11% 높게 나타났다. 목숨이 좌우될 정도의 질병이나 부상을 직접 겪은 대상자의 총점 평균은 29.01±8.03점으로 비경험자의 총점 평균인 26.03±7.98점에 비해 유의하게 약 11% 높게 나타났다. 이는 해당 트라우마 경험자가 비경험자에 비해 더 낮은 삶의 질 수준을 나타냄을 시사한다. 반면에, 화재 또는 폭발, 교통사고, 독성 물질에 노출, 전투나 전쟁터에 노출, 심각한 인간적 고난, 급작스러운 사고사 목격에 따라서는 트라우마 경험의 차이에 따른 건강관련 삶의 질 총점이 통계적으로 유의한 수준의 차이를 나타내지 않았다.

<표 4-84> 북한에서 겪은 트라우마 경험 차이에 따른 북한이탈주민의 삶의 질 특성

요인	트라우마 경험	평균*	표준 편차	95% 신뢰구간		유의 확률
1. 자연재난	아니오	25.49	8.07	24.25	26.72	.002
	예	28.39	7.83	27.05	29.73	
2. 화재 또는 폭발	아니오	26.48	7.98	25.50	27.45	.106
	예	28.70	8.55	25.96	31.44	
3. 교통사고	아니오	26.56	8.03	25.59	27.53	.218
	예	28.33	8.43	25.48	31.19	
4. 직장, 집, 또는 여가 활동 중 심각한 사고	아니오	26.73	8.00	25.79	27.66	.695
	예	27.50	9.53	22.76	32.24	
5. 독성 물질에 노출	아니오	26.66	8.01	25.74	27.59	.180
	예	30.33	10.09	22.58	38.09	
6. 신체폭력	아니오	25.32	8.09	24.06	26.58	.001
	예	28.46	7.77	27.16	29.76	
7. 무기로 공격당함	아니오	26.29	8.07	25.31	27.26	.005
	예	30.24	7.38	27.78	32.71	
8. 성폭력	아니오	26.49	8.10	25.54	27.44	.012
	예	31.53	6.15	28.37	34.69	
9. 기타 원하지 않거나 불편한 성적 경험	아니오	26.50	8.07	25.55	27.44	.012
	예	31.69	6.79	28.07	35.31	
10. 전투나 전쟁터에 노출	아니오	26.82	8.12	25.87	27.76	.655
	예	25.75	7.26	21.14	30.36	
11. 감금	아니오	25.96	7.77	24.94	26.97	.002
	예	29.36	8.54	27.35	31.37	
12. 목숨이 좌우될 정도의 질병이나 부상	아니오	26.03	7.98	24.98	27.07	.005
	예	29.01	8.03	27.17	30.86	
13. 심각한 인간적 고난	아니오	25.65	8.23	24.08	27.22	.071
	예	27.41	7.95	26.27	28.54	
14. 급작스러운 변사	아니오	25.54	8.43	24.35	26.73	<.001
	예	29.07	6.87	27.74	30.40	
15. 급작스러운 사고사	아니오	26.52	8.26	25.37	27.67	.436
	예	27.29	7.73	25.75	28.84	
16. 나 자신 때문에 발생했던 다른 사람의 심각한 부상, 상해 또는 사망	아니오	26.59	8.03	25.67	27.51	<.001
	예	37.40	2.19	34.68	40.12	

*점수가 높을수록 장애가 높고 삶의 질이 낮음을 의미

트라우마 경험에 따른 취업(취업여부)는 다음과 같다. 북한이탈주민이 북한 거주 당시 경험한 트라우마 여부와 취업여부 간의 연관성을 알아보기 위하여 피어슨의 카이제곱 검정을 수행하였다. 분석 결과, 북한 거주 당시 경험한 모든 유형의 트라우마 경험 여부는 취업여부와 유의한 연관성을 나타내지 않았다.

<표 4-85> 북한에서 겪은 트라우마 경험 차이에 따른 북한이탈주민의 취업여부 특성

요인	트라우마 경험	취업		미취업		합계		유의확률
		빈도(회)	백분율(%)	빈도(회)	백분율(%)	빈도(회)	백분율(%)	
1. 자연재난	아니오	74	44.31	93	55.69	167	100	.069
	예	73	54.89	60	45.11	133	100	
2. 화재 또는 폭발	아니오	128	49.23	132	50.77	260	100	.838
	예	19	47.50	21	52.50	40	100	
3. 교통사고	아니오	127	48.11	137	51.89	264	100	.402
	예	20	55.56	16	44.44	36	100	
4. 직장, 집, 또는 여가 활동 중 심각한 사고	아니오	136	48.23	146	51.77	282	100	.337
	예	11	61.11	7	38.89	18	100	
5. 독성 물질에 노출	아니오	142	48.80	149	51.20	291	100	.746
	예	5	55.56	4	44.44	9	100	
6. 신체폭력	아니오	78	48.45	83	51.55	161	100	.837
	예	69	49.64	70	50.36	139	100	
7. 무기로 공격당함	아니오	126	47.91	137	52.09	263	100	.313
	예	21	56.76	16	43.24	37	100	
8. 성폭력	아니오	136	48.06	147	51.94	283	100	.182
	예	11	64.71	6	35.29	17	100	
9. 기타 원하지 않거나 불편한 성적 경험	아니오	137	48.24	147	51.76	284	100	.267
	예	10	62.50	6	37.50	16	100	
10. 전투나 전쟁터에 노출	아니오	144	50.00	144	50.00	288	100	.139
	예	3	25.00	9	75.00	12	100	
11. 감금	아니오	112	49.12	116	50.88	228	100	.940
	예	35	48.61	37	51.39	72	100	
12. 목숨이 좌우될 정도의 질병이나 부상	아니오	109	48.44	116	51.56	225	100	.739
	예	38	50.67	37	49.33	75	100	
13. 심각한 인간적 고난	아니오	54	50.00	54	50.00	108	100	.795
	예	93	48.44	99	51.56	192	100	
14. 급작스러운 변사	아니오	93	47.69	102	52.31	195	100	.537
	예	54	51.43	51	48.57	105	100	
15. 급작스러운 사고사	아니오	97	48.26	104	51.74	201	100	.714
	예	50	50.51	49	49.49	99	100	
16. 나 자신 때문에 발생했던 다른 사람의 심각한 부상, 상해 또는 사망	아니오	144	48.81	151	51.19	295	100	.679
	예	3	60.00	2	40.00	5	100	
합계		147	49.00	153	51.00	300	100	

2) 탈북 기간 동안 트라우마 경험에 따른 차이

제3국을 경유하여 남한에 입국한 북한이탈주민이 탈북 기간 동안에 경험한 트라우마 여부에 따른 외상 후 스트레스 증상 특성을 알아보기 위하여 외상 후 스트레스 장애 체크리스트(PCL-5 with a item of PCL-IV) 총점에 차이가 있는지를 분석하고, 이를 유의하게 차이가 컸던 순으로 살펴보면 다음과 같다.

홍수나 태풍, 폭풍, 지진과 같은 자연재난을 직접 겪은 대상자의 총점 평균은 57.50±18.45점으로 비경험자의 총점 평균인 38.06±23.06점에 비해 유의하게 약 51% 높게 나타나 가장 차이가 컸다. 그다음으로는 총이나 칼에 맞거나, 칼, 총, 폭탄으로 위협 당하는 등과 같이 무기에 의한 공격을 직접 겪은 대상자의 총점 평균은 54.30±17.86점으로 비경험자의 총점 평균인 37.01±23.10점에 비해 유의하게 약 47% 높게 나타났다. 목숨이 좌우될 정도의 질병이나 부상을 직접 겪은 대상자의 총점 평균은 54.17±21.37점으로 비경험자의 총점 평균인 37.83±22.98점에 비해 유의하게 약 43% 높게 나타났다. 강제적인 노동이나 지속적인 굶주림 또는 식량 부족, 지속적인 노숙 상태, 고문과 같은 심각한 인간적 고난을 직접 겪은 대상자의 총점 평균은 53.20±21.79점으로 비경험자의 총점 평균인 37.63±22.93점에 비해 유의하게 약 41% 높게 나타났다. 공격, 가격(손, 주먹, 몽둥이로 공격), 따귀를 맞음, 또는 발로 채이거나 두들겨 맞는 등의 신체폭력을 직접 겪은 대상자의 총점 평균은 46.85±22.06점으로 비경험자의 총점 평균인 36.21±23.02점에 비해 유의하게 약 29% 높게 나타났다. 이는 해당 트라우마 경험자가 비경험자에 비해 더 높은 외상 후 스트레스 증상 수준을 나타냄을 시사한다.

반면에, 화재 또는 폭발, 교통사고, 직장이나 집 또는 여가 활동 중 심각한 사고, 성폭력, 기타 원하지 않거나 불편한 성적 경험, 감금, 급작스러운 변사 목격, 급작스러운 사고사 목격에 따라서는 트라우마 경험 여부에 따른 외상 후 스트레스장애 체크리스트(PCL-5 with a item of PCL-IV) 총점이 통계적으로 유의한 수준의 차이를 나타내지 않았다.

독성 물질에 노출, 전투나 전쟁터에 노출, 나 자신 때문에 발생했던 다른 사람의 심각한 부상이나 상해 또는 사망 목격의 경우에는 통계적 검정을 수행하는 데 필요한 최소한의 트라우마 경험자 수가 미달하여 분석에서 제외하였다.

〈표 4-86〉 탈북 기간 동안 겪은 트라우마 경험 차이에 따른 북한이탈주민의 외상 후 스트레스 증상

요인	트라우마 경험	평균	표준편차	95% 신뢰구간		유의확률
1. 자연재난	아니오	38.06	23.06	34.60	41.52	.020
	예	57.50	18.45	42.07	72.93	
2. 화재 또는 폭발	아니오	38.91	23.14	35.49	42.32	.947
	예	40.00	38.18	-303.07	383.07	
3. 교통사고	아니오	38.70	23.06	35.27	42.14	.466
	예	46.40	29.16	10.19	82.61	
4. 직장, 집, 또는 여가 활동 중 심각한 사고	아니오	38.64	22.97	35.23	42.05	.283
	예	51.25	33.08	-1.39	103.89	
5. 독성 물질에 노출	아니오	N/A				
	예					
6. 신체폭력	아니오	36.21	23.02	32.30	40.13	.007
	예	46.85	22.06	40.30	53.40	
7. 무기로 공격당함	아니오	37.01	23.10	33.41	40.60	.002
	예	54.30	17.86	45.94	62.66	
8. 성폭력	아니오	38.53	23.60	34.82	42.23	.553
	예	41.61	20.41	32.78	50.44	
9. 기타 원하지 않거나 불편한 성적 경험	아니오	38.38	23.64	34.66	42.11	.429
	예	42.42	20.03	33.96	50.87	
10. 전투나 전쟁터에 노출	아니오	N/A				
	예					
11. 감금	아니오	37.82	24.22	33.63	42.00	.303
	예	41.80	20.16	36.07	47.53	
12. 목숨이 좌우될 정도의 질병이나 부상	아니오	37.83	22.98	34.34	41.32	.002
	예	54.17	21.37	40.59	67.75	
13. 심각한 인간적 고난	아니오	37.63	22.93	34.11	41.14	.012
	예	53.20	21.79	41.13	65.27	
14. 급작스러운 변사	아니오	38.67	23.10	35.24	42.09	.335
	예	50.00	27.84	5.69	94.31	
15. 급작스러운 사고사	아니오	39.32	23.25	35.84	42.80	.243
	예	28.86	20.62	9.79	47.93	
16. 나 자신 때문에 발생했던 다른 사람의 심각한 부상, 상해 또는 사망	아니오	N/A				
	예					

탈북 기간 동안 겪은 트라우마 경험 차이에 따른 북한이탈주민의 삶의 질 차이는 다음과 같다. 북한이탈주민이 탈북기간 동안 경험한 트라우마 여부에 따른 북한이탈주민의 건강관련 삶의 질 특성을 알아보기 위하여 SF-8(Short Form-8 Health survey) 총점에 차이가 있는지를 분석하고, 이를 유의하게 차이가 컸던 순으로 살펴보면 다음과 같다. 척도의 특성 상 점수가 높을수록 장애 수준이 높고 삶의 질이 낮음을 의미한다. 총이나 칼에 맞거나, 칼, 총, 폭탄으로 위협 당하는 등과 같이 무기에 의한 공격을 직접 겪은 대상자의 총점 평균은 32.20±5.64점으로 비경험자의 총점 평균인 26.91±8.12점에 비해 유의하게 약 20% 높게 나타났다. 홍수나 태풍, 폭풍, 지진과 같은 자연재난을 직접 겪은 대상자 또한 총점 평균은 32.63±5.76점으로 비경험자의 총점 평균인 27.25±8.07점에 비해 유의하게 약 20% 높게 나타났다. 그다음으로는 목숨이 좌우될 정도의 질병이나 부상을 직접 겪은 대상자의 총점 평균은 32.42±6.63점으로 비경험자의 총점 평균인 27.14±8.04점에 비해 유의하게 약 19% 높게 나타났다. 강제적인 노동이나 지속적인 굶주림 또는 식량 부족, 지속적인 노숙 상태, 고문과 같은 심각한 인간적 고난을 직접 겪은 대상자의 총점 평균은 31.47±7.57점으로 비경험자의 총점 평균인 27.13±8.01점에 비해 유의하게 약 16% 높게 나타났다. 공격, 가격(손, 주먹, 몽둥이로 공격), 따귀를 맞음, 또는 발로 채이거나 두들겨 맞는 등의 신체폭력을 직접 겪은 대상자의 총점 평균은 30.07±6.63점으로 비경험자의 총점 평균인 26.61±8.31점에 비해 유의하게 약 12% 높게 나타났다. 이는 해당 트라우마 경험자가 비경험자에 비해 더 낮은 삶의 질 수준을 나타냄을 시사한다. 반면에, 화재 또는 폭발, 교통사고, 직장이나 집 또는 여가활동 중 심각한 사고, 성폭력, 기타 원하지 않거나 불편한 성적 경험, 감금, 급작스러운 변사 목격, 급작스러운 사고사 목격에 따라서는 트라우마 경험의 차이에 따른 건강관련 삶의 질 총점이 통계적으로 유의한 수준의 차이를 나타내지 않았다. 독성물질에 노출, 전투나 전쟁터에 노출, 나 자신 때문에 발생했던 다른 사람의 심각한 부상이나 상해 또는 사망 목격의 경우에는 통계적 검정을 수행하는 데 필요한 최소한의 트라우마 경험자 수가 미달하여 분석에서 제외하였다.

<표 4-87> 탈북 기간 동안 겪은 트라우마 경험 차이에 따른 북한이탈주민의 삶의 질 특성

요인	트라우마 경험	평균*	표준 편차	95% 신뢰구간		유의 확률
1. 자연재난	아니오	27.25	8.07	26.04	28.47	.065
	예	32.63	5.76	27.81	37.44	
2. 화재 또는 폭발	아니오	27.44	8.06	26.25	28.63	.427
	예	32.00	7.07	-31.53	95.53	
3. 교통사고	아니오	27.44	8.11	26.24	28.65	.632
	예	29.20	5.76	22.05	36.35	
4. 직장, 집, 또는 여가 활동 중 심각한 사고	아니오	27.45	7.95	26.27	28.63	.804
	예	29.25	13.25	8.17	50.33	
5. 독성 물질에 노출	아니오	N/A				
	예					
6. 신체폭력	아니오	26.61	8.31	25.20	28.03	.005
	예	30.07	6.63	28.09	32.04	
7. 무기로 공격당함	아니오	26.91	8.12	25.64	28.17	.001
	예	32.20	5.64	29.56	34.84	
8. 성폭력	아니오	27.49	8.15	26.21	28.77	.993
	예	27.48	7.45	24.26	30.70	
9. 기타 원하지 않거나 불편한 성적 경험	아니오	27.39	7.98	26.14	28.65	.680
	예	28.13	8.64	24.48	31.77	
10. 전투나 전쟁터에 노출	아니오	N/A				
	예					
11. 감금	아니오	27.04	8.21	25.62	28.46	.221
	예	28.68	7.54	26.54	30.82	
12. 목숨이 좌우될 정도의 질병이나 부상	아니오	27.14	8.04	25.92	28.36	.028
	예	32.42	6.63	28.21	36.63	
13. 심각한 인간적 고난	아니오	27.13	8.01	25.90	28.36	.045
	예	31.47	7.57	27.28	35.66	
14. 급작스러운 변사	아니오	27.38	7.99	26.20	28.57	.233
	예	32.25	10.50	15.54	48.96	
15. 급작스러운 사고사	아니오	27.56	7.94	26.38	28.75	.553
	예	25.71	10.98	15.56	35.87	
16. 나 자신 때문에 발생했던 다른 사람의 심각한 부상, 상해 또는 사망	아니오	N/A				
	예					

*점수가 높을수록 장애가 높고 삶의 질이 낮음을 의미

탈북 기간 동안 겪은 트라우마 경험 차이에 따른 북한이탈주민의 취업여부 차이는 다음과 같다. 북한이탈주민이 탈북기간 동안 경험한 트라우마 여부와 취업 여부 간의 연관성을 알아보기 위하여 피어슨의 카이제곱 검정을 수행하였다. 분석 결과, 탈북 중 홍수나 태풍, 폭풍, 지진과 같은 자연재난을 직접 겪은 대상자 8명 중 취업자는 7명(87.50%)으로 비경험자 292명 중 취업자 140명(47.95%)에 비해 유의하게 취업자에 해당한 비율이 더 높은 것으로 나타났다. 공격, 가격(손, 주먹, 몽둥이로 공격), 따귀를 맞음, 또는 발로 채이거나 두들겨 맞는 등의 신체폭력을 직접 겪은 대상자 50명 중 취업자는 33명(66.00%)으로 비경험자 250명 중 취업자 114명(45.60%)에 비해 유의하게 취업자에 해당한 비율이 더 높았다. 납치, 유괴, 인질 및 전쟁 포로 등으로 인한 감금을 직접 겪은 대상자 53명 중 취업자는 33명(62.26%)으로 비경험자 247명 중 취업자 114(46.15%)에 비해 유의하게 취업자에 해당한 비율이 더 높았다. 목숨이 좌우될 정도의 질병이나 부상을 직접 겪은 대상자 12명 중 취업자는 10명(83.33%)으로 비경험자 288명 중 취업자 137명(47.57%)에 비해 유의하게 취업자에 해당한 비율이 더 높았다. 화재 또는 폭발, 교통사고, 직장, 집, 또는 여가활동 중 심각한 사고, 무기로 공격당함, 성폭력, 기타 원하지 않거나 불편한 성적 경험, 심각한 인간적 고난, 급작스러운 변사, 급작스러운 사고사, 나 자신 때문에 발생했던 다른 사람의 심각한 부상, 상해 또는 사망에 따라서는 유의한 연관성을 나타내지 않았다. 독성 물질에 노출 및 전투나 전쟁터에 노출의 경우에는 통계적 검정을 수행하는 데 필요한 최소한의 트라우마 경험자 수가 미달하여 분석에서 제외하였다.

<표 4-88> 탈북 기간 동안 겪은 트라우마 경험 차이에 따른 북한이탈주민의 취업여부 특성

요인	트라우마 경험	취업		미취업		합계		유의확률
		빈도 (회)	백분율 (%)	빈도 (회)	백분율 (%)	빈도 (회)	백분율 (%)	
1. 자연재난	아니오	140	47.95	152	52.05	292	100	.033
	예	7	87.50	1	12.50	8	100	
2. 화재 또는 폭발	아니오	145	48.66	153	51.34	298	100	.239
	예	2	100.00	0	0.00	2	100	
3. 교통사고	아니오	143	48.47	152	51.53	295	100	.207
	예	4	80.00	1	20.00	5	100	
4. 직장, 집, 또는 여가 활동 중 심각한 사고	아니오	145	49.32	149	50.68	294	100	.685
	예	2	33.33	4	66.67	6	100	
5. 독성 물질에 노출	아니오	147	49.00	153	51.00	300	100	N/A
	예	N/A						
6. 신체폭력	아니오	114	45.60	136	54.40	250	100	.008
	예	33	66.00	17	34.00	50	100	
7. 무기로 공격당함	아니오	135	48.39	144	51.61	279	100	.501
	예	12	57.14	9	42.86	21	100	
8. 성폭력	아니오	133	48.19	143	51.81	276	100	.398
	예	14	58.33	10	41.67	24	100	
9. 기타 원하지 않거나 불편한 성적 경험	아니오	132	47.83	144	52.17	276	100	.203
	예	15	62.50	9	37.50	24	100	
10. 전투나 전쟁터에 노출	아니오	147	49.00	153	51.00	300	100	N/A
	예	N/A						
11. 감금	아니오	114	46.15	133	53.85	247	100	.033
	예	33	62.26	20	37.74	53	100	
12. 목숨이 좌우될 정도의 질병이나 부상	아니오	137	47.57	151	52.43	288	100	.018
	예	10	83.33	2	16.67	12	100	
13. 심각한 인간적 고난	아니오	137	48.41	146	51.59	283	100	.460
	예	10	58.82	7	41.18	17	100	
14. 급작스러운 변사	아니오	145	48.99	151	51.01	296	100	.999
	예	2	50.00	2	50.00	4	100	
15. 급작스러운 사고사	아니오	144	49.15	149	50.85	293	100	.999
	예	3	42.86	4	57.14	7	100	
16. 나 자신 때문에 발생했던 다른 사람의 심각한 부상, 상해 또는 사망	아니오	146	48.83	153	51.17	299	100	.490
	예	1	100.00	0	0.00	1	100	
합계		147	49.00	153	51.00	300	100	

3) 남한에서 트라우마 경험에 따른 차이

북한이탈주민이 남한에서 경험한 트라우마 여부에 따른 외상 후 스트레스 증상 특성을 알아보기 위하여 외상 후 스트레스 장애 체크리스트(PCL-5 with a item of PCL-IV) 총점에 차이가 있는지를 분석한 결과는 다음과 같다.

직장이나 집 또는 여가 활동 중 심각한 사고를 직접 겪은 대상자의 총점 평균은 61.75±13.67점으로 비경험자의 총점 평균인 35.50±22.83점에 비해 유의하게 약 74% 높게 나타났다.

자연재난, 교통사고, 신체폭력, 무기로 공격당함, 기타 원하지 않거나 불편한 성적 경험, 목숨이 좌우될 정도의 질병이나 부상, 급작스러운 변사의 목격, 급작스러운 사고사의 목격에 따라서는 트라우마 경험 여부에 따른 외상 후 스트레스 장애 체크리스트(PCL-5 with a item of PCL-IV) 총점이 통계적으로 유의한 수준의 차이를 나타내지 않았다.

화재 또는 폭발, 독성 물질에 노출, 성폭력, 전투나 전쟁터에 노출, 감금, 심각한 인간적 고난, 나 자신 때문에 발생했던 다른 사람의 심각한 부상이나 상해 또는 사망 목격의 경우에는 통계적 검정을 수행하는 데 필요한 최소한의 트라우마 경험자 수가 미달하여 분석에서 제외하였다.

남한에서 겪은 트라우마 경험 차이에 따른 북한이탈주민의 삶의 질 차이는 다음과 같다. 북한이탈주민이 남한에서 경험한 트라우마 여부에 따른 북한이탈주민의 건강관련 삶의 질 특성을 알아보기 위하여 SF-8(Short Form-8 Health survey) 총점에 차이가 있는지를 분석하고, 이를 유의하게 차이가 컸던 순으로 살펴보면 다음과 같다. 직장이나 집 또는 여가 활동 중 심각한 사고를 직접 겪은 대상자의 총점 평균은 32.75±2.63점으로 비경험자의 총점 평균인 26.69±8.10점에 비해 유의하게 약 23% 높게 나타났다. 이는 해당 트라우마 경험자가 비경험자에 비해 더 낮은 삶의 질 수준을 나타냄을 시사한다. 반면에, 자연재난, 교통사고, 신체폭력, 무기로 공격당함, 기타 원하지 않거나 불편한 성적 경험, 목숨이 좌우될 정도의 질병이나 부상, 급작스러운 변사의 목격, 급작스러운 사고사의 목격에 따라서는 트라우마 경험 여부에 따른 건강관련 삶의 질 총점이 통계적으로 유의한 수준의 차이를 나타내지 않았다. 화재 또는 폭발, 독성 물질에 노출, 성폭력, 전투나 전쟁터에 노출, 감금, 심각한 인간적 고난, 나 자신 때문에 발생했던 다른 사람의 심각한 부상이나 상해 또는 사망 목격의 경우에는 통계적 검정을 수행하는 데 필요한 최소한의 트라우마 경험자 수가 미달하여 분석에서 제외하였다.

<표 4-89> 남한에서 겪은 트라우마 경험 차이에 따른 북한이탈주민의 외상 후 스트레스 증상

요인	트라우마 경험	평균	표준편차	95% 신뢰구간		유의확률
1. 자연재난	아니오	35.90	22.98	33.26	38.54	.829
	예	34.00	21.62	14.01	53.99	
2. 화재 또는 폭발	아니오	N/A				
	예					
3. 교통사고	아니오	35.71	23.19	32.92	38.49	.744
	예	37.13	20.70	29.53	44.72	
4. 직장, 집, 또는 여가 활동 중 심각한 사고	아니오	35.50	22.83	32.89	38.12	.023
	예	61.75	13.67	40.00	83.50	
5. 독성 물질에 노출	아니오	N/A				
	예					
6. 신체폭력	아니오	35.63	22.97	32.98	38.27	.302
	예	44.13	20.60	26.90	61.35	
7. 무기로 공격당함	아니오	35.77	23.02	33.14	38.40	.590
	예	42.00	14.28	19.27	64.73	
8. 성폭력	아니오	N/A				
	예					
9. 기타 원하지 않거나 불편한 성적 경험	아니오	35.88	22.91	33.27	38.50	.829
	예	33.00	28.48	-37.74	103.74	
10. 전투나 전쟁터에 노출	아니오	N/A				
	예					
11. 감금	아니오	N/A				
	예					
12. 목숨이 좌우될 정도의 질병이나 부상	아니오	35.72	22.60	33.10	38.35	.649
	예	38.69	30.16	20.46	56.92	
13. 심각한 인간적 고난	아니오	N/A				
	예					
14. 급작스러운 변사	아니오	36.02	22.92	33.40	38.65	.323
	예	25.80	22.52	-2.16	53.76	
15. 급작스러운 사고사	아니오	35.67	22.92	33.05	38.29	.169
	예	54.00	15.72	14.96	93.04	
16. 나 자신 때문에 발생했던 다른 사람의 심각한 부상, 상해 또는 사망	아니오	N/A				
	예					

<표 4-90> 남한에서 겪은 트라우마 경험 차이에 따른 북한이탈주민의 삶의 질 특성

요인	트라우마 경험	평균*	표준편차	95% 신뢰구간		유의확률
1. 자연재난	아니오	26.74	8.05	25.82	27.67	.651
	예	28.14	10.02	18.87	37.41	
2. 화재 또는 폭발	아니오	N/A				
	예					
3. 교통사고	아니오	26.69	8.10	25.72	27.66	.606
	예	27.48	7.99	24.55	30.41	
4. 직장, 집, 또는 여가 활동 중 심각한 사고	아니오	26.69	8.10	25.77	27.62	.014
	예	32.75	2.63	28.57	36.93	
5. 독성 물질에 노출	아니오	N/A				
	예					
6. 신체폭력	아니오	26.67	8.08	25.74	27.60	.172
	예	30.63	7.56	24.31	36.94	
7. 무기로 공격당함	아니오	26.83	8.10	25.91	27.76	.261
	예	22.25	6.24	12.32	32.18	
8. 성폭력	아니오	N/A				
	예					
9. 기타 원하지 않거나 불편한 성적 경험	아니오	26.77	8.05	25.85	27.69	.961
	예	27.00	13.45	-6.42	60.42	
10. 전투나 전쟁터에 노출	아니오	N/A				
	예					
11. 감금	아니오	N/A				
	예					
12. 목숨이 좌우될 정도의 질병이나 부상	아니오	26.67	8.01	25.74	27.60	.294
	예	29.08	9.58	23.29	34.87	
13. 심각한 인간적 고난	아니오	N/A				
	예					
14. 급작스러운 변사	아니오	26.80	8.06	25.87	27.72	.702
	예	25.40	10.57	12.27	38.53	
15. 급작스러운 사고사	아니오	26.76	8.10	25.83	27.68	.738
	예	28.33	6.43	12.36	44.30	
16. 나 자신 때문에 발생했던 다른 사람의 심각한 부상, 상해 또는 사망	아니오	N/A				
	예					

*점수가 높을수록 장애가 높고 삶의 질이 낮음을 의미

남한에서 겪은 트라우마 경험 차이에 따른 북한이탈주민의 취업여부 차이는 다음과 같다. 북한이탈주민이 남한에서 동안 경험한 트라우마 여부와 취업여부 간의 연관성을 알아보기 위하여 피어슨의 카이제곱 검정을 수행하였다. 분석 결과, 자동차 사고, 선박 사고, 기차 사고 및 비행기 추락과 같은 교통사고를 직접 겪은 대상자 31명 중 취업자는 22명(70.97%)으로 비경험자 269명 중 취업자 125명(46.47%)에 비해 유의하게 취업자에 해당한 비율이 더 높았다. 자연재난, 화재 또는 폭발, 교통사고, 직장, 집, 또는 여가 활동 중 심각한 사고, 신체폭력, 무기로 공격당함, 기타 원하지 않거나 불편한 성적 경험, 목숨이 좌우될 정도의 질병이나 부상, 심각한 인간적 고난, 급작스러운 변사, 급작스러운 사고사, 나 자신 때문에 발생했던 다른 사람의 심각한 부상, 상해, 또는 사망에 따라서는 유의한 연관성을 나타내지 않았다. 독성 물질에 노출, 성폭력, 전투나 전쟁터에 노출, 감금의 경우에는 통계적 검정을 수행하는 데 필요한 최소한의 트라우마 경험자 수가 미달하여 분석에서 제외하였다.

〈표 4-91〉 남한에서 겪은 트라우마 경험 차이에 따른 북한이탈주민의 취업여부 특성

요인	트라우마 경험	취업		미취업		합계		유의확률
		빈도 (회)	백분율 (%)	빈도 (회)	백분율 (%)	빈도 (회)	백분율 (%)	
1. 자연재난	아니오	142	48.46	151	51.54	293	100	.275
	예	5	71.43	2	28.57	7	100	
2. 화재 또는 폭발	아니오	147	49.16	152	50.84	299	100	1.000
	예	0	0.00	1	100.00	1	100	
3. 교통사고	아니오	125	46.47	144	53.53	269	100	.013
	예	22	70.97	9	29.03	31	100	
4. 직장, 집, 또는 여가 활동 중 심각한 사고	아니오	143	48.31	153	51.69	296	100	.056
	예	4	100.00	0	0.00	4	100	
5. 독성 물질에 노출	아니오	147	49.00	153	51.00	300	100	N/A
	예	N/A						
6. 신체폭력	아니오	142	48.63	150	51.37	292	100	.494
	예	5	62.50	3	37.50	8	100	
7. 무기로 공격당함	아니오	143	48.31	153	51.69	296	100	.056
	예	4	100.00	0	0.00	4	100	
8. 성폭력	아니오	147	49.00	153	51.00	300	100	N/A
	예	N/A						
9. 기타 원하지 않거나 불편한 성적 경험	아니오	146	49.16	151	50.84	297	100	1.000
	예	1	33.33	2	66.67	3	100	
10. 전투나 전쟁터에 노출	아니오	147	49.00	153	51.00	300	100	N/A
	예	N/A						
11. 감금	아니오	147	49.00	153	51.00	300	100	N/A
	예	N/A						
12. 목숨이 좌우될 정도의 질병이나 부상	아니오	138	48.08	149	51.92	287	100	.163
	예	9	69.23	4	30.77	13	100	
13. 심각한 인간적 고난	아니오	146	48.83	153	51.17	299	100	.490
	예	1	100.00	0	0.00	1	100	
14. 급작스러운 변사	아니오	143	48.47	152	51.53	295	100	.207
	예	4	80.00	1	20.00	5	100	
15. 급작스러운 사고사	아니오	145	48.82	152	51.18	297	100	.616
	예	2	66.67	1	33.33	3	100	
16. 나 자신 때문에 발생했던 다른 사람의 심각한 부상, 상해 또는 사망	아니오	146	48.83	153	51.17	299	100	.490
	예	1	100.00	0	0.00	1	100	
합계		147	49.00	153	51.00	300	100	

5. 심층 면접 내용 분석 결과

북한이탈주민 20명을 대상으로 심층 면접을 시행하고 요약한 후 영역 및 범주 별로 내용을 분석한 결과를 정리하면 다음과 같았다.

가. 참여자 특성

심층 면접에 참여한 북한이탈주민 20명의 인구학적 정보는 〈표 4-92〉와 같다. 여성이 17명(85%)이고, 평균 나이는 53.35세이고 35~83세로 분포되어 있었다. 배우자나 동거인과 함께 사는 경우가 7명(35%)이고 취업하고 있는 자가 8명(40%)이며 중국 등 제3국 거주했던 경험이 있는 경우가 14명(70%)이었다.

심층면접의 방법 및 절차는 제2장 연구방법에 구체적으로 기술되어 있다.

〈표 4-92〉 심층 면접 참여자의 일반적 특성

특징	참여자(n=20)
여자	17(85%)
나이	평균 53.35 세 (35~83)
기혼 혹은 동거	7(35%)
취업	8(40%)
제3국 거주경험	14(70%)

나. 내용 분석 결과

자료는 3가지 영역으로 분류되었다. ① 인권침해 트라우마 경험, ②인권침해 트라우마 경험이 한국 정착에 미치는 영향, ③ 탈북민이 생각하는 심리적 안정을 돕기 위한 방안 등이었다. 그리고 인권침해 트라우마 경험이 한국 정착에 미치는 영향은 대인관계, 직장생활, 일상생활 세가지 범주로 나뉘었다.

1) 인권침해 트라우마 경험

이 영역은 탈북민들이 생각하는 그동안 경험했던 대표적인 인권침해 트라우마 경험 중 기억나는 것에 대해 이야기 한 것이다. 그 주된 내용들은 8가지 범주로 나눌 수 있었다. 각 범주 별로 탈북민들 인터뷰 내용을 구체적으로 요약 정리하면 다음과 같다.

가) 심한 굶주림 또는 질병, 죽음

- ❍ 가족들 모두가 1997년 시기에는 굶게 되면서, 어지럽고, 의식이 혼탁해 지는 경험을 하게 되었다. 당시, 내가 이렇게 살다가 가족들까지 모두 죽을 것 같아서, 두려웠다. 북한의 가족이 굶어 돌아가신 일을 목격하였다.
- ❍ 고난의 행군 시절(1999년), 길바닥, 도랑에 굶주림으로 죽은 시체가 널려있었다. 치우지 않아 벌레들이 꼬이는 상황을 많이 목격하였다.
- ❍ 김일성 사망 이후, 경제 불황이 시작되었고, 배급이 사라지며 길거리에 굶주림으로 죽어가는 사람들을 많이 목격하였다. 두려움과 함께 나의 미래라는 생각이 들었다. 꽃제비가 굶어서 길가에서 쓰러지는데 옥수수 국수를 줬는데 힘이 없어서 먹지도 못했다.

나) 정치범 수용소 잔혹성

- ❍ 복역 생활 동안, 식사가 제대로 공급되지 못해 체중이 죽을 정도로 빠지기도 하였다. 강냉이를 주었는데 쥐똥, 머리카락, 털 등이 섞여 있어 제대로 먹을 수 없었고, 간을 하지 않아 늘 힘이 빠진 상태로 지냈다.

○ 낮에는 노동을 하였는데 감자를 캐는데 힘이 없어 땅을 깊이 파지 못해 맞기도 하였다. 이로 인해 이가 다 빠져 틀니를 하게 되었고, 얼굴에 흉터가 많다. 감옥에 들어가면, 인간대우를 못 받고, 때리고, 발로 차고,,, 이로 인한 분노가 많이 생기고…

○ 밤이면 구호를 하는데 하루는 함께 지내는 사람이 구호를 틀렸고, 이로 인해 다 같이 잠을 못 자게 되자 화가 난 사람들이 그 사람을 구타하였고 다음날 죽었다.

○ 고문하는 모습을 목격함, 발을 때려서 피가 나고 잠을 하루종일 재우지 않고 하루 종일 거울을 쳐다보게 하여 환시를 유발하였다.

○ 죄수들이 사는 집이 사람이 머리를 둘 수 없을 정도로 작고, “하늘을 영원히 볼 수 없다” 등의 협박을 죄수들에게 하고, 형편없는 거주환경을 제공하는 것을 듣고 보게 됨. 예를 들어, 인분을 끓이는 가마와 밥을 먹는 가마를 같이 끓이는 냄새 등의 위생적으로 있을 수 없는 환경이었다.

○ 여자들은 돈을 자궁 안에 넣어 몰래 가지고 다녔는데 집결소에서 남자가 동의 없이 불쑥 자궁 속으로 손을 넣어 검사하며 수치감을 느꼈다.

다) 공개 처형

○ 공개처형을 목격하기도 하였는데 억울하게 죽어가는 것에 대한 화와 불만이 있었다. 말하나만 잘못해도 죽는 경우가 있었다. 총살, 교수형을 목격했었는데 총이 머리를 통과해서 머리에서 피가 나오는 모습이 지금도 생각이 난다.

라) 국가기관/군대에서 매질

○ 국경근처 살았고 경비대가 무고한 사람들 괴롭히고 구타하였다. 팔 꺽이고 총구로 머리 맞아 터지는 모습을 목격하였다. 가족이 중국에 넘어가다 잡혀 매질을 당하였다.

○ 가족이 북한을 탈북 후, 남은 가족들을 심하게 매질을 하면서, 욕하고, 발로 차는 등의 폭력적 행위를 당하였다.

○ 강을 건너다가 물에 떠내려가는데 국경 경비대에 잡혀서 참나무 장작으로 맞았다. 중국 간 이유를 대라고 하면서 때렸다.

마) 토대가 나쁘다는 이유로 인한 차별

○ 북한에서 생활 당시, 기독교라는 이유로 토대가 안 좋다고 하여 교육의 기회를 제한받았다.
○ 어려서부터 토대가 나쁘다는 이유로 지내던 평양에서 쫓겨나서 생활하였고, 공부를 잘했으나 대학에 가지 못하였다. 시집갈 때도 토대 나쁜 사람에게 갔다.
○ 부모가 처벌을 받으면, 중학교 의무교육만 받고, 노동만 해야 하고, 발언을 잘못만 해도, 처벌의 수준이 달라져 감옥으로 가게 된다.

바) 공개적인 자아비판

○ 공개적인 자아비판을 받은 적 있고, 마음이 힘들고, 억울하다.

사) 통신 검열 또는 록화기 단속

○ 친구가 한국 영화를 보는데 장군의 아들, 가을동화 등을 봤다고 들었고 그러면 공개재판을 한다고 들었다. 거기서 주범은 총살을 한다고 들었다. 내 친구는 한국 영화를 봐서 7년형을 받은 친구도 있었다.

아) 탈북 기간 동안

○ 중국에 사는 동안 신분증이 없어 늘 잡힌다는 긴장과 불안 속에 살았다.
○ 중국에 건너가 인신매매 당하여 한 집에서 생활하였고, 일거수일투족을 감시당하며 생활하였고, 외출도 불가능하였다. 화장실 갈 때까지도 쫓아왔었다. 이 때, 심한 우울증을 경험하였다.
○ 탈출을 감행하다 잡혀 하루 종일 몰매를 당하였고, 죽을 뻔 하였는데 이웃에게 목격되어 겨우 살게 되었다.

2) 인권침해 트라우마 경험이 한국 정착에 미치는 영향

이 영역은 탈북민들이 생각하는 그동안 경험했던 인권침해 트라우마 경험들이 현재 한국에 정착하여 생활하는데 어떤 영향을 미치는가에 대한 것이다. 그 주요 내용들은 3가지 범주로 나눌 수 있었다. 각 범주 별로 탈북민들 인터뷰 내용을 구체적으로 요약 정리하면 다음과 같다.

가) 대인관계에 미치는 영향

- ❍ 사람들에게 맞았던 기억, 다들 살기 위해 예민해있던 기억 때문에 사람이 무섭다. 밖을 나갈 수가 없다. 계속 사람을 의심하게 된다.
- ❍ 사람들을 대할 때 감정적으로 불안정하다. 상대가 아무 뜻 없이 하는 말에도 짜증이 나고 화가 난다. 욱하는 감정이 먼저 올라와서 사람들을 대하기가 어렵다. 예민해져서 쉽게 싸우고 때리고 싶다. 그래서 상대방도 나를 피한다.
- ❍ 한국에 와서, 회사일 하면서 지속되는 차별과 불합리함으로 스트레스 받았고, 혼자서 자녀들을 양육해야하는 환경에 큰 스트레스를 받으며 더 힘들어졌다.

나) 직장생활에 미치는 영향

- ❍ 온몸이 아프고, 밤에 내내 불안하고 악몽에 시달려 일상생활이 힘들어 일도 하지 못한다.
- ❍ 직장생활을 하다보면 밥을 먹어야 하는 자리가 생기는데 가기가 두렵고 이로 인한 오해를 많이 산다.
- ❍ 바깥에 나가는 게 두렵기 때문에 일을 할 수가 없다. 힘들었던 기억들 때문에 괴로워서 일을 할 생각조차 못한다.

다) 일상생활에 미치는 영향

- ❍ 교화소 경험들이 자꾸 떠오르고, 힘들었던 기억들이 떠오르고, 끔찍한 꿈(성폭행 당한 꿈, 도망 다니던 꿈, 엄마 꿈 등등)을 꾸고 잠을 깊게 못잔다.
- ❍ 집에 있는데도 혼자 있어도 불안하여 계속 문을 확인하게 된다. 소스라치게 놀란다.

❍ 머리가 쥐어짜듯이 아프다. 부풀어오르듯이 아프다. 머리를 열어두는게 낫다 싶을 정도다.

❍ 온몸이 아프다 보니까 고달프고 힘이 든다.

❍ 우울하고 무기력하고 일상생활이 잘되지 않는다. 외출을 못하고 나가기도 싫다.

❍ 지하철을 못 탄다. 밀폐된 공간이 감옥 같은 느낌에 들어가지 못하고, 네모난 책상만 봐도 두렵다. 터널 들어갈 때(답답한 곳) 숨을 쉬기가 어렵다.

3) 탈북민의 심리적 안정을 돕기 위한 제안

이 영역은 탈북민들이 그동안 경험했던 인권침해 트라우마 경험으로 인한 심리적 어려움을 돕기 위해서 어떤 것들이 필요할지 스스로 생각하는 방안에 대해 이야기 한 것이다. 그 주된 내용들은 4가지 범주로 나눌 수 있었다. 각 범주 별로 탈북민들 인터뷰 내용을 구체적으로 요약 정리하면 다음과 같다.

가) 금전적 지원

❍ 돈을 벌고 먹고 생활을 할 수 있는 것. 금전적인 지원이 충분했으면 한다.

나) 남한 사회의 편견 차별 보호 장치

❍ 회사 내 차별과 모멸이 심한데 이를 보호해줄 수 있는 장치가 있으면 좋겠다. 북한의 실상을 남한 사람들이 잘 모르게 되는 경우가 많다. 남한사람들의 생각이 점점 북한사람들이 별로 어렵게 남한으로 온 것 같지도 않고, 지원에 대하여서도, 불만이 있다고 생각을 하는 것 같고, 북한 이탈주민들에게 지원하는 것을 부정적으로 보는 경우가 있는 것 같다.

다) 남한 사회 정착이후에도 맞춤형 실질적 교육

❍ 하나원을 졸업해서 나갈 때, 한국사회에 대한 깊이를 잘 모르고 나오게 된다. 하나원, 하나센터에 있을 때보다는 하나센터 교육 후에 도움을 받을 수 있는 프로그램들이 있으면 좋겠다. 실질적 경험들이 같이 있으면 좋겠다. 먼저 사회를 맞부딪힐 수 있는 기회가 있고 난 후에야 정착과 직업 관련된 교육이 되지 않을까 한다. 대인관계에 대한 방법, 접근 등에 대한 것이 더 기본적인 교육이지 않을까

한다.

- ❍ 직장에서 살아남을 수 있는 개개인의 실력에 따른 국가의 정착지원 도움이 필요하다. 정착 프로그램에서 모두 무상으로 남한에서 교육을 해 주는데, 북한이탈주민들이 자신의 실력을 잘 모르고, 자신의 실력과 상관없이 일을 하려하는 욕심을 부리게 되고, 이로 인해 북한 이탈 주민들의 나쁜 이미지를 한국에 심어 준다고 생각된다.

라) 의료지원 및 정신과 치료 연계

- ❍ 병원진료를 안정적으로 받을 수 있도록 의료지원을 잘 해줬으면 좋겠다. 병원에 가려고 하면 돈이 많이 나올까봐도 겁나고 절차 등이 익숙하지 못하다.
- ❍ 정신과 치료로 인한 편견이나 불이익이 없으면 좋겠다. 사람들이 예전에 심리적인 충격으로 문제가 있다는 것을 모르고, 병을 모르기 때문에 치료 받으러 가기가 힘들다고 한다. 그런 것들을 알려주면 좋겠다. 함께 이야기를 나눌 수 있는 사람, 이야기를 서로 들어주고 공감해줄 수 있는 사람들, 속마음을 터놓고 이야기를 할 수 있는 프로그램이 있으면 좋겠다. 탈북과정에서 많이 고생하고 와서, 힘들어 하는 것 같다. 하지만, 아직도 어디를 어떻게 가서 도움을 받을 수 있는지 잘 모르고 있어서, 많이 알려지면 좋겠다.

심층 면담 내용을 분석한 결과 북한이탈주민은 일반 사람들이 평생에 한번 겪기도 힘든 **심각한 인권침해 트라우마 경험**을 다양한 유형으로 때로는 장기간 반복적으로 겪음을 알 수 있었다. 그리고 이러한 경험들이 실질적으로 남한에 와서 사람을 만나고, 직장생활을 잘 하는데 **어떤 걸림돌로 작용하는지 구체적으로 이해**할 수 있었고 심한 경우에 일상생활에서도 **그 후유증으로 고통받고 있음**이 드러났다. 그들은 그 대책으로 **금전적 지원**에서부터 일반 남한 국민들의 탈북민에 대한 **인식개선,** 개개인의 실력이나 특성에 맞는 **맞춤형 직업교육**의 지속까지 다양한 제안을 내놓고 있었고 무엇보다 **아플 때 잘 치료 받을 수 있도록** 지원을 원하고 특히 **정신과 치료를 어떨 때 받는지 몰라서, 어디에서 받을 수 있는지 몰라서 홍보가 필요하고 그들에게 맞는 치료프로그램에 대한 갈망과 불이익 없이 치료 받을 수 있기를 희망**하고 있어 이에 대한 대책이 필요함을 알 수 있었다.

6. 소결

이번 실태조사 결과 북한이탈주민은 북한과 탈북기간의 인권침해 트라우마 경험이 높으며 그러한 경험들은 PTSD 등 정신건강 문제 수준을 유의하게 높이고 삶의 질을 의미있게 저하시키는 것으로 나타나 이에 대한 근본적 대책이 필요함을 알 수 있었다.

인권침해 경험으로는 북한이탈주민이 경험한 다빈도 인권침해 사건은 북한에서 공개적인 자아비판을 직접 경험한 경우가 247명(82.33%)으로 가장 많았고, 북한에서 심한 굶주림 또는 질병을 직접 경험한 경우가 188명(62.67%), 북한에서 이웃과 당원의 감시와 고발을 직접 경험한 경우가 171명(57.00%) 등의 순으로 나타났다. 그 밖에 심각한 인권침해 경험으로는 탈북기간 중 여성 또는 아동 인신매매를 직접 경험한 경우가 65명(21.67%, 여성 중 26.53%), 북한에서 여성에 대한 성폭력을 직접 경험한 경우가 22명(여성 중 9.0%), 공개처형 목격이 246명(82.0%)으로 분석되었다.

트라우마 경험도 심각하다. 북한에서 직접 겪은 사건은 강제적인 노동이나 지속적인 굶주림 또는 식량 부족, 지속적인 노숙 상태, 고문과 같은 심각한 인간적 고난이 192명(64.00%)에 달해 가장 많았고, 그다음으로는 공격, 가격(손, 주먹, 몽둥이로 공격), 따귀를 맞음, 또는 발로 채이거나 두들겨 맞는 등의 신체폭력 경험이 139명(46.33%)으로 나타났으며, 홍수나 태풍, 폭풍, 지진과 같은 자연재난 133명(44.33%) 순으로 나타났다. 탈북 기간에 직접 겪은 사건은 납치, 유괴, 인질 및 전쟁 포로 등으로 인해 감금된 경험이 51명(27.57%)에 달해 가장 많았으며, 그다음으로는 공격, 가격(손, 주먹, 몽둥이로 공격), 따귀를 맞음, 또는 발로 채이거나 두들겨 맞는 등의 신체폭력 경험이 49명(26.49%), 성폭행 시도 또는 완력이나 위협 하에 성적 행위 경험 23명(12.43%) 순으로 나타났다. 남한에서 직접 겪은 사건으로는 자동차 사고, 선박 사고, 기차 사고 및 비행기 추락과 같은 교통사고 경험 31명(10.33%), 목숨이 좌우될 정도의 질병이나 부상 경험 13명(4.33%) 순이었다.

스노우볼샘플링 특성 상 일반화 할 수는 없다는 제한점은 있으나, **PTSD 임상군이 북한이탈주민의 56%에 달하는데,** 보건복지부(2016) 정신건강 실태조사결과에서 일반 국민의 PTDS 평생유병율이 1.5%(남1.3%, 여1.8%)이고 일년유병율이 0.5%(남0.2%, 여0.8%)인 것을 고려하면, **탈북민에서 PTSD 유병율이 월등히 높음을 알 수 있다. 그 외 우울, 불안, 불면도 50~60%가 임상군으로 나타났고, 자살고위험군도 약 25%에 달한다.**

특히, **중국에서 거주 경험자가** 남한 직행자에 비해 PTSD 등 정신건강문제가 더 심각하였고 (불면증 제외) 삶의 질은 더 낮다는 것을 알 수 있었는데 이러한 문제가 정착기간이 오래된다고 의미 있는 호전을 보이지는 않아서 자연적으로 해결되기 어렵고 근본적 대책이 필요함을 알 수 있다. 반면, 취업자 비율은 중국거주경험자가 더 높았다. 북한이탈주민들이 취업 후 직장을 그만 둔 이유는 건강상 이유가 61%, 스트레스와 부적응이 15% 순이었다.

인권침해 경험 유형별로는 북한에서 '심한 굶주림 또는 질병을 경험', '정치범 수용소의 잔혹한 경험', '공개처형', '탈북으로 인한 처벌', '여성에 대한 성폭력' 경험을 했을 때 높은 PTSD 증상 수준을 나타내었다. 다만, 탈북 기간 중에는 '여성에 대한 성폭력' 경험은 집단 간 PTSD 증상 수준의 의미 있는 차이는 아니었지만 '여성 또는 아동 인신 매매'은 집단 간 PTSD 증상 정도의 차이가 있었다. 삶의 질에 있어서는 '공개처형', '탈북으로 인한 처벌', '북한에서의 성폭력', '탈북 중 인신매매' 경험을 직접 경험 혹은 목격한 경우 유의하게 낮은 것으로 나타났다. 취업 여부는 '탈북 중 인신매매'를 경험하거나 목격한 경우 오히려 취업자가 더 많아 중국 거주가 교란변수로 작용했을 것으로 보인다.

트라우마 경험 유형별 PTSD 수준을 분석한 결과는 다음과 같아. 트라우마 사건 별 직접 경한 경우와 그렇지 않은 경우 집단간 차이를 분석하였을 때, 북**한에서와 탈북기간에는 공통적으로,** 공격, 가격(손, 주먹, 몽둥이로 공격), 따귀를 맞음, 또는 발로 채이거나 두들겨 맞는 등의 신체폭력', '총이나 칼에 맞거나, 칼, 총, 폭탄으로 위협 당하는 등과 같이 무기에 의한 공격', '목숨이 좌우될 정도의 질병이나 부상', '홍수나 태풍, 폭풍, 지진과 같은 자연재난', 을 경험한 집단이 그렇지 않은 집단에 비해 각각 더 높은 PTSD 증상 수준을 나타내었다. **추가적으로 북한에서는** ' 나 자신 때문에 발생했던 다른 사람의 심각한 부상, 상해 또는 사망을 목격', '납치, 유괴, 인질 및 전쟁 포로 등으로 인한 감금', '타인이 살인이나 자살 등의 급작스러운 변사를 당하는 것을 목격'을 경험한 집단에서도 그렇지 않은 집단에 비해 각각 더 높은 PTSD 증상 수준을 나타내었다. 추**가적으로 탈북기간 동안에는**, '강제적인 노동이나 지속적인 굶주림 또는 식량 부족, 지속적인 노숙 상태, 고문과 같은 심각한 인간적 고난'을 경험한 집단도 그렇지 않은 집단에 비해 각각 더 높은 PTSD 증상 수준을 나타내었다. 남한에서 경험한 집단이 그렇지 않은 집단에 비해 각각 유의하게 더 높은 PTSD 증상 수준을 나타내었던 트라우마 경험은, '직장이나 집 또는 여가 활동 중 심각한 사고'만으로 밝혀졌다.

트라우마 경험 유형별 삶의 질 수준은 트라우마 사건 별 직접 경험한 경우와 그렇지 않은 경우 집단간 차이를 분석하였을 때, **북한과 탈북 기간 공통적으로** '자

연재난', '신체폭력', '무기로 공격당함', '목숨이 좌우될 정도의 질병이나 부상'을 직접 경험한 경우 그렇지 않은 경우보다 삶의 질이 유의하게 낮게 나타났다. **추가적으로 북한**에서는 '성폭력', '기타 원하지 않거나 불편한 성적 경험 공격', '감금', '급작스러운 변사 목격', '나 때문에 발생했던 다른 사람의 심각한 부상, 상해 또는 사망 목격'을 직접 경험한 경우 삶의 질이 유의하게 낮았다. 한편, **탈북기간**에는 추가적으로 '심각한 인간적 고난'을 경험한 경우 삶의 질 유의하게 낮았다. **남한**에서는 직접 경험했을 때 유의하게 삶의 질이 낮는 것은 '직장이나 집 또는 여가 활동 중 심각한 사고'만으로 나타났다.

북한에서의 트라우마 경험은 **취업 유무**에 유의한 영향을 주지 못하였고. **탈북기간의** '자연재난', '신체폭력', '감금', '목숨이 좌우될 정도의 질병이나 부상'을 경험한 경우 오히려 취업자 비율이 높았는데 중국 거주가 교란 변수로 작용한 것으로 해석된다. **남한에서는** '자동차 사고, 선박 사고, 기차 사고 및 비행기 추락과 같은 교통사고' 경험이 있는 경우 오히려 취업자 비율이 높았는데 이는 취업 경험이 결과라기보다 원인으로 해석된다.

심층면접 조사에서는 그들의 **인권침해 트라우마 경험으로 인한 후유증상이 대인관계, 직장생활, 일상생활에 어떠한 부정적 영향을 주는 지** 구체적으로 분석되어있다. 즉, **대인관계 영역에서** '사람이 무섭다.', '사람을 의심한다.', '욱해서 싸운다.', '사람을 피한다.'는 등의 부정적 영향을 호소하였고, **직장 생활 영역**에서는 '온몸이 아프다', '회식 자리를 가기 싫다.', '바깥에 나가는 게 두려워 일을 할 수 없다'는 등의 부정적 영향을 호소하였다. **일상생활에** 악몽, 불면, 불안, 우울, 두통, 무기력 등을 포함한 트라우마 관련 재경험, 특정 상황 회피 반응, 과각성, 부정적 기분 인지 등 다양한 후유 증상을 호소하였다. **탈북민의 심리적 안정을 돕기 위한 제안으로는** 금전적 지원, 남한 회사나 사회에서 탈북민을 향한 차별과 부정적 시선으로부터 보호 받을 수 있는 방법, 병원 진료를 안정적으로 받을 수 있는 의료지원, 정신과 치료에 대한 편견이나 불이익이 없으면 좋겠고 홍보가 필요, 하나원 졸업 후 실제 사회생활을 하면서 받을 수 있는 개개인의 능력에 맞고 실질적 도움이 되는 교육 등 다양한 의견을 제안하였다.

제5장 정책적 제언

1. 현행제도

가. 하나원의 정신건강 관리

탈북민들은 국내 입국 후 합동신문센터에서 일정 기간 조사를 받은 후 하나원에 입소하게 된다. 하나원은 탈북민을 위한 사회정착교육기관으로 안성에 위치한 여성과 노인, 아동을 위한 교육기관인 하나원과 화천에 위치한 남성과 정착 탈북민을 위한 교육기관인 제2하나원으로 나누어져 있다.

(1) 하나원의 정신건강 관련 인력

하나원에서는 사회적응교육과 함께 신체적 및 정신적 건강 관리가 이루어진다. 우선 안성 하나원에는 정신건강의학과 의사 1인과 함께 임상심리사 1인, 상담심리사 1인이 근무하고 있다. 심리수련요원을 합치면 의사 1명과 심리상담사 4명이 근무하고 있으며, 이들은 입소 교육생들을 위한 정신건강 스크리닝과 상담, 심리 프로그램, 진료 및 약물 치료를 담당하고 있으며, 퇴소 시 지속적인 진료가 필요한 탈북민들이 지역사회에서 지속적으로 치료받을 수 있도록 연계하는 역할도 한다. 화천 하나원 역시 심리상담사 1인이 근무하고 있으며, 정신건강의학과 전문의가 방문하여 상담을 하고 있다. 안성과 마찬가지로 지속적인 진료가 상담이 필요한 탈북민의 경우 지역사회에서 지속적으로 치료받을 수 있도록 연계하는 역할을 담당한다.

정신건강의학과 문제뿐만 아니라 신체 증상에 대한 치료를 위해 하나원에는 내과 의사, 치과 의사, 산부인과 의사 등의 의료진과 간호 인력이 구성되어 있다. 하나원에서의 진료는 주로 개인의원급의 진료가 이루어지기 때문에, 입원이나 평가가 더 필요한 경우 안성은 안성 의료원, 춘천 인성병원 등의 2차 의료기관, 단국대 병원, 이나 단국대학교 병원, 분당서울대학교병원, 강원대학교 병원 등의 3차 의료기관을 이용하며, 정신건강의학과의 경우 국립춘천병원, 국립정신건강센터 등에서 입원이나 평가를 시행

하기도 한다.

(2) 하나원의 정신건강서비스

하나원에서 시행하는 탈북민을 위한 정신건강 서비스는 크게 몇 가지로 구분할 수 있다.

우선 탈북민들에 대한 스크리닝이다. 입소 탈북민들에 대한 정신건강 문제를 알아보기 위하여 정신건강평가척도를 통한 스크리닝이 이루어지며, 이에 대한 임상심리사와 정신건강의학과 의사의 초기 평가가 이루어진다.

둘째 탈북민들에 대한 상담 및 진료이다. 입소 초기의 평가 결과 및 합동신문 당시 정신건강문제가 있었던 탈북민들을 중심으로 상담 및 진료가 이루어진다.

셋째 탈북민들에 대한 심리안정 프로그램 운영이다. 이들을 위한 심리안정 프로그램은 주로 하나원의 교육 과정 중 초기에 집중되어 있다. 프로그램의 변화가 있지만 대체적으로 탈북민들을 마음챙김 명상에 기반한 정서안정 프로그램, 숲 체험을 통한 스트레스 관리 프로그램, 대인관계 훈련 등 스트레스 감소나 교육, 인지행동치료 등을 기반으로 한 프로그램으로 구성되어 있다.

나. 정착지원제도의 기본개념

현행 북한이탈주민 지원정책은 2005년부터 인센티브제도를 수용하면서 '수혜적 보호'에서'자립·자활' 중심의 정착지원으로 기본 개념이 변화하였다. 특히 2010년 북한이탈주민지원재단을 설치하고 지역적응센터(하나센터)와 연계하여 북한이탈주민의 정착지원 서비스의 종합적 지원기능을 확충하였다. 지역차원에서 하나센터 전문상담사, 정착도우미 제도 등 민간의 지원체계간 연계를 통해 정착지원 역량을 강화하는 것으로 나타나고 있다. <그림5-1> 은 통일부의 현행 정착지원제도를 간략하게 표시한 것이다.

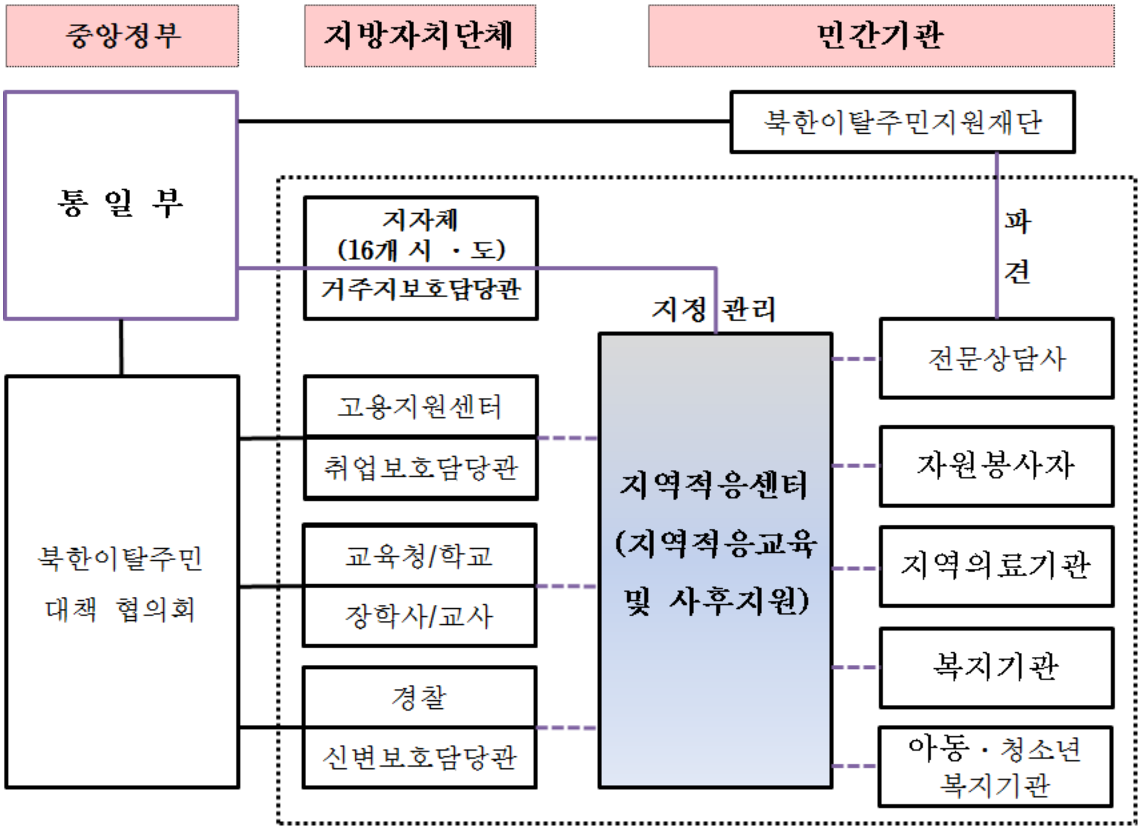

<그림 5-1> 통일부의 현행 정착지원제도 개요: 북한이탈주민 정착지원 체계도

다. 전문상담사 제도

북한이탈주민의 정신건강 문제와 관련하여 운영되고 있는 제도는 전문상담사 제도이다.

현행 법규의 내용을 살펴보면, 우선 전체적으로 건강권 보장과 관련하여 <북한이탈주민의 보호 및 정착지원에 관한법률> 제4조의 3항은 통일부 장관이 다음과 같은 내용에 대한 기본계획을 매3년마다 작성하도록 규정하고 있다.

> ② 기본계획에는 다음 각 호의 사항이 포함되어야 한다. <개정 2014.5.28.>
> 1. 보호대상자의 보호 및 정착에 필요한 교육에 관한 사항
> 2. 보호대상자의 직업훈련, 고용촉진 및 고용유지에 관한 사항
> 3. 보호대상자에 대한 정착지원시설의 설치 · 운영 및 주거지원에 관한 사항
> 4. 보호대상자에 대한 의료지원 및 생활보호 등에 관한 사항

5. 보호대상자의 사회통합 및 인식개선에 관한 사항

6. 그 밖에 보호대상자의 보호, 정착지원 및 고용촉진 등을 위하여 통일부장관이 필요하다고 인정하는 사항

③ 통일부장관은 관계 중앙행정기관의 장과 협의하여 기본계획에 따른 연도별 시행계획(이하 “시행계획“이라 한다)을 수립 · 시행하여야 한다.

④ 통일부장관은 기본계획 및 시행계획을 수립하고자 할 경우에 제22조제3항에 따른 실태조사의 결과를 반영하여야 한다.

위 법령의 내용 속에는 북한이탈주민의 의료지원 및 생활보호 등에 관한 사항이 포함되어 있으며 북한이탈주민의 정신건강문제도 당연히 이에 포함되어 있다. 그러나 북한이탈주민의 정신건강에 대한 별도의 내용은 전혀 없으며 다음과 같은 전문상담사 제도 규정 항목이 전부인 것으로 나타난다. 즉 “정신건강 검사 등 전문적 서비스를 제공” 할 수 있는 전문상담사 제도를 운영한다는 것이다.

제22조의2(전문상담사제도 운영) ① 통일부장관은 거주지에 전입한 북한이탈주민에 대한 정신건강 검사 등 전문적 상담서비스를 제공할 수 있는 북한이탈주민 전문상담사제도를 운영할 수 있다. <개정 2014.5.28.>

② 통일부장관은 제1항에 따른 전문상담사의 자질 향상을 위한 보수교육을 실시할 수 있다.

③ 제1항 및 제2항에 따른 전문상담사의 운영방법 및 절차 등에 관한 사항은 통일부령으로 정한다.

현재 전국적으로 약 100여명의 전문 상담사가 지역적응센터(하나센터)에서 활동하고 있으며 전문상담사가 제공하고 있는 서비스는 다음과 같다.

❍ 북한이탈주민이 정착과정에서 겪는 다양한 문제점과 어려움에 대한 깊은 이해를 바탕으로 전문상담사는 심리, 취업, 의료, 교육, 복지 등의 분야에서 종합 상담서비스

❍ 북한이탈주민에게 ‘찾아가는 상담 서비스’ 를 제공하여 거주지 편입 이후 북한이탈주민의 빠른 지역사회 안착을 유도

라. 정신건강 지원 서비스 현황

현행 제도 가운데 건강지원 서비스와 관련한 내용을 요약하면, 일단 의료비 지원과 의료서비스 지원으로 나누어 살펴볼 수 있다. 2017년 현재 북한이탈주민에 대한 각종 의료비 제원혜택은 전체적으로 증가된 것으로 나타나고 있다(아래 표 5-1 참조).

〈표 5-1〉 2017년 의료비지원 대비표

구분	2016년(기존)		2017(현행)	비고
입원기간	2일 이상		3일 이상	
본인부담 최소납부액	10만원 이상		30만원 이상	
치과(완전틀니)	상·하약 최대 70만원, 편척(1약) 35만원		상·하약 최대 100만원, 편척(1약)50만원	연 1회<br>일반·중증 중복 가능
출산의료	본인부담금의 30% 지원		본인부담금의 50% 지원	일반, 연 1회
불임치료<br>(인공수정,<br>시험관아기)	-		본인부담금의 50%	일반, 연1회(당일 건)
지원 제외항목	-		입원기간 중 질병치료와 관련 없는 검사비	MRI 등
입국년도 지원율	일반질환		일반질환	만성·중증 및 희귀성 50%, 한방치료 30%
	2001~2010	2011~2016	2002~2017	
	30%	40%	30%	

출처: 남북하나재단 홈페이지

위 표에서 나타나듯이 본인부담 최소납부액, 치과진료, 출산의료 및 불임치료 등에서 북한이탈주민에 대한 의료비 지원은 늘어났다. 한편 북한이탈주민에 대한 정신건강의학과 의료비 지원 현황을 살펴보면 정신건강의학과 진료 후에 진료비 지원이 필요한 경우는 남북하나재단 생활안정부에서 지원하는 의료비 지원 프로그램을 이용할 수 있다(표 5-2 참조).

〈표 5-2〉 남북하나재단 공공의료체계 의료비 지원

■ 대상 : 2002년 1월 1일 이후 입국자 *비보호자도 포함<br>■ 질병범위 : F00-F99<br>■ 지원금액 : 정신질환 및 만성중증질환 지원금액 합산하여 최대 700만원 이내 지원<br>■ 기타 :<br>- 의료급여(1종,2종)는 본인 부담금 전액<br>- 건강보험(직장, 지역)가입자는 본인 부담금50%<br><br>* 비보호자 : 「북한이탈주민정착지원법」의 적용을 받지 않는 북한이탈주민

또한 통일부 하나원에서 의료협약을 체결한 협력병원에서 정신건강의학과 진료를 받는 경우에 본인 부담금 일부(특진비 무료 및 비급여 진료비 20-80%) 지원이 가능하다. 다음 <표 5-3>은 정신건강의학과 진료 과목을 운영 중인 협약 병원 목록이다.

〈표 5-3〉 북한이탈주민 의료지원 협약병원 현황 (17.8.3 기준): 정신건강의학과 진료 과목 운영 병원

번호	단체명	지역	지원현황	비고
1	국립중앙의료원	서울시 중구	북한이탈주민 의료비 지원(80%) 북한이탈주민 상담실 운영	(사)새조위 상담실 지원
2	분당서울대학교병원	경기도 성남시	하나원 교육생 진료비 전액 무료 진료	
3	국립정신건강센터	서울시 중랑구	북한이탈주민 진료 지원 센터 운영	
4	가톨릭 의정부 성모병원	경기도 의정부시	의정부, 양주 지역 의료지원 특진비 무료, 비급여 20%	
5	충남대학교병원	대전광역시 중구	대전, 충남, 충북지역 북한이탈주민 의료지원, 특진비 무료, 비급여 30% 지원, 새터민 상담실 운영	(사)새조위와 공동 협약
6	서울특별시 서울의료원	서울특별시 중랑구	북한이탈주민 전염병관리 및 산부인과 특화진료, 80% 의료비 지원 상담실 운영	(사)새조위와 공동 협약
7	관동대학교 일산명지병원	경기도 고양시	경기지역 북한이탈주민 의료지원, 특진비 무료, 비급여 30% 지원	(사)새조위와 공동 협약
8	부산대학병원	부산시 서구	부산지역 북한이탈주민 의료지원, 특진비 무료, 비급여 20% 지원	(사)새조위와 공동 협약
9	전북대학교병원	전북 전주시 덕진구	전북지역 북한이탈주민 의료지원, 특진비 무료, 비급여 20% 지원	(사)새조위와 공동 협약
10	인천적십자병원	인천시 연수구	인천지역 북한이탈주민 의료지원, 비급여 50%지원, 새터민 상담실 운영	(사)새조위와 공동 협약
11	강원대학교병원	강원도 춘천시	강원지역 북한이탈주민 의료지원, 특진비 무료, 비급여 30%지원	(사)새조위와 공동 협약
12	경상대학교병원	경남 진주시	경남지역 북한이탈주민 의료지원, 특진비 무료, 비급여 20% 지원	
13	국립춘천병원	강원도 춘천시	하나원 정신과 방문진료, 프로그램 운영 협력, 북한이탈주민 진료 지원 센터 운영	
14	조선대학교병원	광주광역시	광주. 전남지역 북한이탈주민 의료지원, 비급여 20% 지원	
15	부산의료원	부산광역시	부산지역 북한이탈주민 의료지원 공공의료사업 연계 가능	
16	대구의료원	대구광역시	대구지역 북한이탈주민 의료지원 공공의료사업 연계 가능	

번호	단체명	지역	지원현황	비고
17	인천의료원	인천광역시	인천지역 북한이탈주민 의료지원<br>공공의료사업 연계 가능	
18	수원의료원	경기도 수원시	경기지역 북한이탈주민 의료지원<br>공공의료사업 연계 가능	
19	원주의료원	강원도 원주시	강원지역 북한이탈주민 의료지원<br>공공의료사업 연계 가능	
20	속초의료원	강원도 속초시	강원지역 북한이탈주민 의료지원<br>공공의료사업 연계 가능	
21	삼척의료원	강원도 삼척시	강원지역 북한이탈주민 의료지원<br>공공의료사업 연계 가능	
22	청주의료원	충북 청주시	충북지역 북한이탈주민 의료지원<br>공공의료사업 연계 가능	
23	홍성의료원	충남 홍성군	충남지역 북한이탈주민 의료지원<br>공공의료사업 연계 가능	
24	군산의료원	전북 군산시	전북지역 북한이탈주민 의료지원<br>공공의료사업 연계 가능	
25	남원의료원	전북 남원시	전북지역 북한이탈주민 의료지원<br>공공의료사업 연계 가능	
26	순천의료원	전남 순천시	전남지역 북한이탈주민 의료지원<br>공공의료사업 연계 가능	
27	목포의료원	전남 목포시	전남지역 북한이탈주민 의료지원<br>공공의료사업 연계 가능	
28	포항의료원	경북 포항시	경북지역 북한이탈주민 의료지원<br>공공의료사업 연계 가능	
29	안동의료원	경북 안동시	경북지역 북한이탈주민 의료지원<br>공공의료사업 연계 가능	
30	제주의료원	제주특별자치도 제주시	제주지역 북한이탈주민 의료지원<br>공공의료사업 연계 가능	
31	서귀포 의료원	제주특별자치도 서귀포시	제주지역 북한이탈주민 의료지원<br>공공의료사업 연계 가능	
32	경기의료원 의정부병원	경기도 의정부시	경기지역 북한이탈주민 의료지원<br>공공의료사업 연계 가능	
33	단국대학교병원	충남 천안시	하나원 교육생 의료비 지원<br>특진비 무료 등	

2. 문제점

가. 북한이탈주민의 인권침해 트리우마 경험으로 인한 정신건강문제의 심각성

국내 정착하는 북한이탈주민의 정신건강 문제가 안정적인 정착을 위협할 정도로 심각한 상태에 있는 것으로 보인다. 이번 실태조사 결과 스노우볼샘플링 특성상 일반화 할 수는 없다는 제한점은 있으나, **북한이탈주민 북한과 탈북기간의 심각한 인권침해 트라우마 경험이 높으며 그러한 경험들은 PTSD 수준을 유의하게 높이는 것으로 나타났다.** 또한, PTSD는 사회적응에 부정적 영향을 주는 것으로 여러 선행연구결과에서 보고되었고, 이번 조사에서도 탈북민의 트라우마 경험들이 삶의 질 저하와 관련성이 있는 것으로 나타났다. 심층면접 조사에서는 그들의 인권침해 트라우마 경험으로 인한 후유증상이 대인관계, 직장생활, 일상생활에 어떠한 부정적 영향을 주는지 구체적으로 분석되어있다.

<표 5-4>에서 나타나듯이 **하나원 교육생 진료 현황을 살펴볼 때도 신체적인 질병뿐만 아니라 정신적 건강이 크게 악화되어 있는 것으로 나타났다**. 2007년 이후 2016년 7월까지 하나원에 입소한 북한이탈주민은 총 10만3,348건의 내과 관련 진료를 받았으며, 이 시기 입국한 북한이탈주민의 숫자가 약 2만명 임을 감안할 때 하나원 입소기간 중 1인당 평균 5건 이상의 내과진료를 받은 것으로 조사되었다.

한편 하나원 교육생의 건강과 관련해서 주목할 점은 북한이탈주민들이 겉으로 드러나는 신체적인 질병 뿐 아니라 정신적인 측면에서의 건강이 심각한 수준에 있다는 점이다. 즉 북한이탈주민의 진료 건수 변화추이를 보면 정신적 외상으로 인한 정신과 진료 건수는 2007년의 110건에서 2009년 12,979건, 2011년 14,292건으로 100배 이상 증가한 것으로 나타나고 있다. 2007년부터 2016년 7월까지 하나원 교육생 정신과 진료 누적 건수는 56,530건이며 이는 지난 10년동안 하나원 입소 교육생 1인당 누적 평균 2.8회 정도 정신과 진료를 받았음을 의미한다.

한편 2012년 이후 최근 정신과 진료 건수는 감소되는 추세로 나타나고 있다. 2015년 진료통계로 살펴본다면, 전체적으로 입국자 수가 50% 정도 규모로 줄어드는 가운데 정신과 진료건수는 2011년 14,292건 대비 1,062건(7.4%)대로 감소되어 내과 25.4%, 한방과 52.2% 등과 비교할 때 특이한 점을 나타내고 있다.

〈표 5-4〉 하나원 교육생 진료현황 통계

(단위: 건)

구분	원내진료										외부진료	합계
	내과	한방과	정신과	치과		비뇨기과(자원봉사)	산부인과	소아청소년과	신경과	소계		
				원내	자원봉사							
'99-'00	1,388	-	-	-	314	-	-	-	-	1,702	262	1,964
'01	1,692	-	-	-	442	-	-	-	-	2,134	306	2,440
'02	4,692	-	-	-	1,068	-	-	-	-	5,760	419	6,179
'03	7,373	-	456	-	1,325	-	549	-	-	9,703	324	10,027
'04	9,452	1,774	1,091	-	1,314	-	1260	-	-	14,891	1,213	16,104
'05	6,396	1,822	875	-	1,564	-	495	-	-	11,152	760	11,912
'06	6,199	2,006	152	-	2,895	-	873	-	-	12,125	946	13,071
'07	11,360	3,860	110	1,108	2,800	-	664	-	-	19,902	774	20,676
'08	11,051	1,345	2,266	2,193	2,011	-	1,432	-	-	20,298	1,463	21,761
'09	10,314	1,543	12,979	3,644	1,140	-	1,633	-	-	31,253	1,774	33,027
'10	19,509	1,968	11,165	1,938	2,913	-	2,644	-	-	40,137	2,459	42,596
'11	21,721	2,444	14,292	3,782	3,127	-	4,138	-	-	49,504	3,181	52,685
'12	9,020	1,649	8,031	4,405	2,018	-	1,592	1,058	-	27,773	2,997	30,770
'13	5,013	921	3,597	5,789	1,698	-	1,088	1,030	-	19,136	1,682	20,818
'14	6,122	1,151	2,509	6,107	1,561	-	1,251	1,335	-	20,036	2,051	22,087
'15	5,534	1,277	1,062	4,892	1,273	12	935	403	-	15,388	1,532	16,920
'16	3,704	771	519	2,582	823	15	1,000	-	95	9,509	1,080	10,589

출처: 통일부 홈페이지

나. 의료기관 내 의료 상담실의 별도 운영체제

현재 **통일부는 북한이탈주민 의료 상담실 운영을 민간에게 위탁하는 형태**를 띄고 있다. 북한이탈주민을 위한 의료 상담실은 의료 수요가 있는 북한이탈주민을 치료 지원이 가능한 의료기관과 연결하거나 **의료기관을 방문한 북한이탈주민의 접수, 진료, 수납 등 일련의 의료기관 이용 절차를 도와주는 기능을 하는 것이므로 당연히 의료기관에서 운영할 수 있도록 위탁하는 것이 합리적일 것이다.** 그것을 민간에 위탁하는 것은 비효율성과 서비스 전달체제의 분리 등 여러 문제를 야기 시킨다.

첫째, 탈북민 의료 상담사의 질을 관련 의료기관에서 관리하고 주기적 보수교육이나 문제 사례에 대한 슈퍼비전이 절대적으로 필요한데 운영 주체가 별도로 되고 있으므로 그것이 불가능하다. 북한이탈주민 의료상담실에서 근무하는 상담사는 주로 탈북민으로서 민간에서 일정 시간의 집단 강의 교육을 이수하면 자체에서 심사하여 자격증을 주는 데, 탈북민을 위한 문화 브로커로서의 기능은 충분할지 모르지만 살제 의료 상담사로서의 전문성은 일반 의료기관의 상담 직원에 못 미치는 것이 현실이다. 그런 전문성이 부족한 의료상담사가 의료기관을 찾은 환자를 직접 응대하고 도와주는 일을 하는데 전문가가 아닌 민간에서 자격증을 주고 관리를 하고 있으므로 만약 문제가 생겨도 의료기관은 권한도 책임도 없는 무력한 상황이 되는 것이다.

둘째, 북한이탈주민 의료 상담실은 별도의 체계 하에 운영되기 때문에 정보 흐름과 서비스 관리의 단절로 인한 불합리성과 비효율성이 야기된다. 일반적으로 의료기관 고객 상담실에서 민원 발생 시 즉각적으로 관련 의료진이나 직원과 공유하여 해결하거나, QI(Quality Improvement) 팀에서 시행하는 만족도 조사 등의 주기적 설문조사를 통해 의료 서비스 개선점을 찾는 것이 가능하다. 그러나 북한이탈주민, 의료상담실은 민간이 운영하고 있고, 한편 의료기관은 개인정보보호법의 적용을 받으므로 환자 관련 상담내용, 만족도나 민원 파악 시 원활한 정보 공유에 걸림돌로 작용하는 것이다.

북한이탈주민을 우리 사회의 보편적인 사회안전망, 보편적인 사회복지 및 보건의료 시스템 속에서 그들을 위해 특화된 서비스를 추가 지원하는 것이 바람직할 것이다. 별도의 주체로 운영 관리하는 것은 특히 정신건강 및 의료 서비스와 같은 전문적 영역에서는 북한이탈주민에게 오히려 불리하게 작용하여 또 하나의 차별이 될 뿐이다.

다. 서비스 전달 체계의 분리

현행 북한이탈주민 정착지원법에 근거한 통일부 정착지원정책의 핵심은 **북한이탈주민을 '특수신분계층'으로 분리하고 대상자에 대한 특례적 보호와 보편적인 국민 사회보장체계와 별도의 탈북자 대상 복지지원 전달체계를 구축**하는 것이다. 즉 대북정책 부서인 통일부가 처음부터 끝까지 북한이탈주민 관리보호와 정착지원을 책임지는 시스템인 것이다. 현행 정착지원 체계는 주무부처인 통일부를 중심으로 산하기관인 북한이탈주민지원재단과 정부의 3종 보호담당관, 그리고 하나센터와 지역협의회 등 주로 정부중심 민간보조형의 지원체계를 갖추고 있다. 이처럼 통일부 주도의 분리형 지원서비스 체계는 20년이 넘어서는 장기적인 북한이탈주민 정착추세와 다양한 욕구체계의 분출 등으로 더 이상 유효하지 않다. 이제 남한 사회 정착이 장기화되고 있는 현상을 주목하여 북한이탈주민에 대한 적극적인 지역사회통합형 거버넌스 체제 구축을 고민해야 할 것이다.

북한이탈주민을 일반국민을 대상으로 하는 보편적 보건의료 지원제도와 분리하여 별도의 서비스전달체계를 만들어서 지원하는 것이 장기적인 안목에서 사회통합에 바람직한가에 대한 의문이 제기된다. '북한이탈주민 만의 별도의 서비스 제공'을 강조하는 현행제도는 우리사회 보편적 복지시스템과 단절된 또 하나의 제도를 분리하는 결과를 가져올 수 있다. 현재 대한민국의 사회복지 안전망과 사회보장 시스템은 생애주기별, 대상별로 다양한 맞춤형 서비스가 제공되고 있다. 특히 보건인력의 전문성과 치료 및 지원의 지속성을 주목할 때 초기지원에 집중되어 있는 통일부 주도 시스템은 정신건강 지원과 관련하여 다음과 같은 한계점이 지적된다.

첫째 전문성 문제이다. 현재 지역적응지원센터에는 전문상담사 제도가 실행되고 있지만, 북한이탈주민이 겪은 외상경험의 심각성과 증상의 심각성에 비해서 볼 때 약간의 기초적인 상담지식 정도를 갖추고 일하는 상담사들이 이들에 대한 전문적인 치료와 관리 기능을 수행하기에는 근본적인 한계점이 있다. 2016년 7월 현재 북한이탈주민지원재단이 운영하는 전문상담사는 86명이며 전문상담사 운영예산은 38억 3천300만원이 소요되고 있다. 2017년 남북하나재단은 신규 전문상담사 채용기준을 강화하여 학사학위 이상자를 필수요건으로 상담, 임상심리, 간호 등 전문직 관련 자격증을 필수요건으로 제시하고 있으며 필기시험을 도입하였다(남북하나재단 홈페이지). 그러나 현직 전문상담사 가운데 15명(17.4%)는 사회복지사 자격증만 가지고 있는 상태이다.

둘째 의료자원에 대한 접근성 문제이다. 현재 우리나라의 정신건강 지원제도와 자원수준을 살펴볼 때 과도한 수도권 집중현상이 나타나고 있다. 또한 수도권

집중을 회피하고 전국적으로 정착인구를 분산시키는 현행 북한이탈주민 주거지원 정책에 따라서 이들에 대한 집중적인 거점 지원도 불가능하다. 현재 23개 지역적응센터에서 활동하는 전문상담사의 지역배치 현황을 살펴보면 대전지역은 상담사 1인이 177명을 담당하고 있으나 경기동부의 경우 663명, 충청북도 507명 등 전문상담사의 지역배치도 불합리한 측면이 있다.

수도권 지역에서는 전문적 치료시설들과 자원인력 자원들이 집중되어 있어서 그나마 중증치료 대상자에 대한 협력 치료가 어느 정도 가능할지 모르지만 지방 거주 북한이탈주민들은 그러한 혜택에서도 소외될 수밖에 없다. 정신건강학과 전문의가 있는 지방대학병원이나 거점 지원 종합병원들도 대도시 거주인구 만이 혜택을 받을 수 있다. 장기치료와 지속적인 관찰이 필요한 정신건강 문제의 특성 상 접근성 제한과 정신건강자원의 대도시 집중문제는 현행 북한이탈주민 정신건강 지원제도의 중대한 한계점이다.

셋째, 의료비 지원제도의 한계이다. 하나센터와 의료기관들이 의료비 지원 제도를 운영하고 있지만, **북한이탈주민의 심각한 인권침해 트라우마 후유증상을 치료하기에는 단순 의료비 지원만으로는 현 보건의료환경에서는 인프라가 부족하다**. 우선, 국내 건강의료보험 체계 내에서 **트라우마 전문 치료센터나 프로그램이 마련되어있지 않고, 그것을 실행할만한 인력도 준비되어 있지 않다.** 특히 의료급여 1,2종 북한이탈주민의 경우 약물치료와 간단한 지지요법을 받는 것에 그치게 된다. 그러나, 다양한 인권침해와 트라우마의 후유증으로 발생하는 복합 PTSD는 인간의 삶을 황폐화시키고, 만성화되고, 다른 공존질환율이 높은 심각한 질환으로 그들을 위한 특화된 북한이탈주민 트라우마 치료를 위한 프로그램, 인력, 시설 등 인프라 구축이 필요하다.

라. 전문상담사 제도의 문제점

현재 지역 하나센터에서 운영되고 있는 전문상담사 제도는 정신건강에 대한 지원 서비스라기보다 심리, 생활, 취업 등 종합적인 상담을 관리하는 내용으로 운영되고 있다. 우선 2017년 새로 바뀐 전문상담사 채용기준을 살펴보면 <표 5-5>와 같다.

<표 5-5> 전문상담사 채용기준

<table>
<tr><th colspan="2">분야</th><th>구분</th><th>근무지</th><th>지원자격</th></tr>
<tr><td colspan="2">공통사항</td><td>-</td><td>-</td><td>▪ 학사학위 이상 취득자
▪ 북한이탈주민의 경우 거주지편입 5년 이상자
(※ 2012년 7월 이전 보호결정 받은 자)</td></tr>
<tr><td rowspan="2">전문 상담사</td><td>심리 생활</td><td>일반경쟁</td><td>하나센터</td><td>▪ 상담, 임상심리, 간호 등 관련분야 국가자격증 소지자
▪ 한국상담학회, 한국상담심리학회에서 발급하는 전문상담사, 상담심리사 자격증 소지자</td></tr>
<tr><td>취업</td><td>제한경쟁</td><td>하나센터</td><td>▪ 장애인복지법 상 장애인으로 등록된 자
▪ 직업상담 관련 국가자격증 소지자로 취업상담 알선 업무경력 1년 이상자</td></tr>
</table>

출처: 남북하나재단 홈페이지

2016년'전문상담사 관리·운영예산'은 38억 3,300만원으로 남북하나재단 사업 전체예산 중 약 19.9%를 차지하고 있으며, 콜센터와 전국 지역적응센터로 파견 된 총 89명의 전문상담사에게(2016년 9월 기준) 지급되는'인건비'는 약 27억 3,400만원으로'전문상담사 관리·운영예산' 중 약 71.2%인 상황이다.

2016년도 국회 예산 정책처 '북한이탈주민 보호 및 정착지원 사업 평가' 자료에 따르면 2010년부터 2016년 3월까지 선발된 162명 전문상담사 가운데 24명(14.8%)는 상담·사회복지·정착지원 활동 경력이 전혀 없으며, 24명 중 17명은 사회경력이 전혀 없었다. 이는 '북한이탈주민의 보호 및 정착지원에 관한 법률' 22조의 2(전문상담사제도 운영)에서 규정하는 '정신건강 검사 등 전문적 상담 서비스를 제공할 수 있는 자'로 볼 수 있는지 의문이다. 실제로 2016년 10월 국회의 통일부 감사과정에서 전문상담사 제도에 대한 문제제기가 있었다(포커스 경제, 2016).

3. 정책대안

가. 의료기관 기반의 북한이탈주민 트라우마 치료 센터 구축

실태 조사 결과에서 나타났듯이 북한이탈주민은 상당수가 심각한 트라우마와 스트레스를 경험하고 그 후유증에 시달리고 있으므로 그들이 우리 사회의 일원으로 잘 통합되고 정착하여 살 수 있도록 돕기 위해서는 **북한이탈주민을 위한 특화된 트라우마 치료 센터**가 마련되어야 한다. 그리하여 북한이탈주민 트라우마 치료는 물론이고 인력 양성, 교육, 연구, 정책 기능을 할 수 있도록 하여야한다. 탈북민 정신건강과 보건의료복지 측면에서 이 부분은 매우 중요하고 시급한 실정이다. 현재 통일부는 20개가 넘는 지역의료기관과 개별적으로 협력체계를 구축하여 이 문제를 해결하려고 시도하고 있다. 그러나 **의료급여 특례나 단순 의료비 지원만으로는** 북한, 탈북과정에서 겪는 북한이탈주민의 독특하고 심각한 트라우마와 남한 정착 과정에서 겪는 그들의 고충을 현 의료 환경에서 제대로 이해하고 치유하기에는 **인프라가 부족하여 한계점이 많다.** 특히 **북한이탈주민들이 정신과 진료에 대해서 가지고 있는 편견 및 보건의료 상식의 부족과 신체화 되어 나타나는 증상**들로 인해 자발적으로 전문적인 트라우마 기관을 찾아서 치료를 받으러 가기도 어려운 실정이다. 또한 전문 상담사 제도는 정신건강 및 트라우마 치료 문제를 해결할 수 있는 대안이 전혀 되지 못한다. 이 문제를 중장기적으로 해결하기 위해서는 보건복지부의 국민정신건강증진 시스템과의 연동하여 부서간에 정보공유와 정책적 협치가 절대적으로 필요하다.

현재 전국적으로 260여개 국공립 의료기관이 있으므로 북**한이탈주민 트라우마 치료센터 시범 사업을 의료기관을 기반으로 시행한 후 거점 센터로 확대 운영**하는 것을 검토할 수 있을 것으로 판단된다. 북한이탈주민의 트라우마 치료 프로그램, 인력, 시설 인프라가 마련되지 않은 채 자원과 환경이 너무나 다른 지역단위 수준에서 지역정착지원 센터와 지역의료기관과의 협정이나 협력지원 협약체결만으로 이들에 대한 전문적인 관리가 이루어지기 어렵다. 북한이탈주민은 의료 수요가 높은 만큼 그 특성상 보건의료적 관점에서 정신건강을 지원하는 것이 더 효율적일 것이다.

나. 북한이탈주민 의료 상담실 운영을 해당 의료기관에 위탁하여 통합 운영

북한이탈주민을 위한 특화된 트라우마 치료센터가 마련되는 것이 이상적이기는 하나 단기간에 구축이 어렵다면, **통일부는 최소한 탈북민 의료 상담실 운영을 민간에게 위탁할 것이 아니라 탈북민 치료 지원을 하고 있는 의료기관에서 운영할 수 있도록 위탁하는 것이 바람직할 것이다.**

보건복지부는 응급실을 방문하는 자살시도자의 재시도 예방을 위한 사업을 의료기관에 위탁하여 예산을 지원하고 있다. 여성가족부는 성폭력 혹은 가정폭력 피해자를 위한 법적, 치료적 다양한 서비스 지원 센터(해바라기센터)를 의료기관에 위탁하여 원스탑으로 설치 운영하고 있다. 이렇듯 의료 서비스 영역을 해당 의료기관에 위탁하는 것은 사용자의 편의와 효율성, 전문성 측면에서 적절하고 합리적인 것이다.

만약 통일부 차원에서 보건복지부와 거버넌스 체제를 구축할 의지가 있다면 북한이탈주민 정신건강 및 보건의료 지원 사업을 보건복지부에 위탁 운영하는 것도 대안이 될 수 있다. 그 예로 서울의료원 북한이탈주민 치과진료 사업을 들 수 있다(표 5-6). 그렇게 하면 **보건복지부는 지역 북한이탈주민 진료센터를 운영하는 의료기관에 이들을 관리하는 전문 인력을 배치하고 하나원으로부터 지역정착단계 등 주요 대상자에 대한 모든 정신건강 및 보건의료 관련 정보를 연계 받아 지역 사회에서도 연속적이고 체계적으로 관리유지하고 지역 보건의료 자원과 공유할 수 있다.** 이들에 대한 생애주기별, 대상특성별 맞춤형 치료프로그램을 전문성을 갖춘 보건의료 인력이 기존의 지역의료 자원과 시설을 활용해서 체계적으로 운영할 수 있다.

앞에서 서술하였듯이 북한이탈주민 만을 대상화한 정신건강 지원제도는 분리와 편견을 만들어 내는 부정적인 결과를 가져올 수 있다. 이 문제에 대한 해법은 하루빨리 북한이탈주민들을 주변화된 특수관리 대상에서 벗어나서 **일반 국민을 위한 보건복지부의 보편적 정신건강 및 보건의료 시스템 속에 북한이탈주민을 위한 전문 치료 인프라를 편입시키는** 것이다.

<표 5-6> 서울의료원 북한이탈주민 치과진료 사업

사업 목적	▪ 탈북 과정에서 발생한 의료공백을 채워 새로운 환경에 적응할 수 있는 기반 구축 및 치료를 받고 싶어도 비용문제로 치료를 포기하는 치과진료에 대한 지원 체계를 마련하여 북한이탈주민의 건강취약 질환인 구강질환에 대한 보건 기능을 강화하고자 함.
사업내용	▪ 서울시 자치행정과의 보조금사업으로 서울의료원 공공의료팀에서 수행하는 사업으로 2015년부터 진행 중임.<br>▪ 대상은 서울시 주민등록이 되어있는 만 12세 이상의 북한이탈주민들을 대상으로 가장 북한이탈주민들의 요구도가 높은 구강진료(치과: 틀니, 보철)를 제공함.<br>▪ 일부 의료비 지원을 하고 있으며, 필요시, 본인부담금을 지원하고 있음. 보조금은 병원을 통해 지원되며, 병원 내 진료를 담당할 의료 인력 및 행정인력에 대한 인건비 및 의료비 지원이 포함됨.
사업 의의	▪ **직접적 병원으로의 지원과 이와 관련된 공공병원의 의료인력을 포함한 지원체계를 통해**, 지역사회의 하나센터와 연계하여, 구강치료가 필요한 대상군을 선별하고, 이를 체계적으로 지원함.

출처: 서울의료원 내부자료

다. 맞춤형 지원체제: 유입경로별, 성별, 생애주기별

3만 명이 넘어서는 북한이탈주민들은 북한사회의 다양한 지역과 사회계층에서 다양한 요인에 의해서 탈출을 결심하고 남한사회에 정착하고 있다. 1990년대 말부터 시작된 북한주민들의 탈출은 초기에는 주로 국가배급체계 붕괴로 인하여 생명을 위협하는 식량난을 극복하기 위한 불가피한 선택이었다. 그러나 적어도 100만명 이상이 희생된 것으로 알려진 식량위기는 이제 만성화 단계로 진행되고 있다. 북한주민들은 이제 만성화된 북한경제난에 다양한 방법으로 적응하였으며 대체로 400개가 넘는 공식적인 장마당과 비공식적인 지하경제를 중심으로 자생적인 생계유지 체계가 정착된 것으로 알려지고 있다.

최근에 유입되는 북한이탈주민들은 생존을 위협하는 식량위기보다는 가족결합을 위한 목적이나 새로운 기회와 자유를 찾아서 넘어오고 있다. 특히 중국에 장기 체류했던 여성들이나 먼저 정착한 가족들의 도움으로 북한에서 직접 넘어오는 사례(남한직행)가 눈에 띄게 증가하고 있다. 이처럼 지난 20년 동안 북한이탈주민의 인구학적 구성과 탈출배경 및 탈출경로 등은 많은 변화를 나타내고 있다. 북한이탈주민에 대한 정착지원체계도 이러한 북한이탈주민의 인구학적 사회적 경제적 지위에 따른 차이점을 감안하여 대상집단에 대한 맞춤형 지원체제를 구축할 필요성이 제기되고 있다.

맞춤형 지원제도의 중요성과 필요성에 대해서는 이미 많은 선행연구자들이 지적하고 있다. 대표적으로 윤인진과 채정민(2010)은 북한이탈주민의 삶의 조건에 따라 고위험군과 저위험군으로 구분하여 각각 극복형, 좌절형, 발전형, 과소성취형으로 유형화를 시도했다. 이를 통해 앞으로 북한이탈주민 정착지원을 위한 사회통합 정책은 수요자 중심으로 보다 세밀하게 구분될 필요성이 있음을 지적한다. 북한이탈주민의 정체성에 따른 범주화, 정체성 재구성 필요, 고위험군에 대한 적극적 접근이 필요하다. 다시 말하자면 ‘맞춤형’ 정책 공급이 필요하다는 의미이다.

본 보고서에서는 다음과 같은 세 가지 인구집단별 맞춤형 지원제도 구축과 시행의 필요성을 제안하고자 한다.

1) 유입경로별 맞춤형 지원제도

첫째 유입경로별 맞춤형 지원제도이다. 이번 조사연구에서 밝혀졌듯이 중국체류를 거쳐서 유입되는 인구와 직접 북한에서 가족의 도움으로 남한에 정착한 인구

집단(남한직행) 사이에는 정신건강 면에서 여러 가지 뚜렷한 차이가 나타나고 있다. 장기간 중국체류 인구집단들은 어느 정도 자본주의사회의 제도와 남한사회에 대한 정보를 가지고 있다. 그러나 중국에 두고 온 남편이나 자식들과의 관계로 인하여 정신적으로 많은 어려움을 겪고 있는 것으로 알려진다. 또한 중국체류 기간 중에 중국공안에 체포되어 북한으로 강제북송을 겪은 북한이탈주민의 경우 상당한 정도의 정신적 트라우마 증상을 나타내고 있다. 본 조사에서는 중국체류 응답자들 가운데 약 33% 정도가 강제북송을 체험한 것으로 나타나고 있다.

반면에 북한에서 직접 남한으로 건너온 인구집단들의 경우에는 남한사회의 제도와 자본주의 시스템에 대한 정보가 상대적으로 적고 적응태도에 있어서도 차이가 나타난다고 보고되고 있다. 예를 들어 본 조사에서 외상 후 스트레스 장애(PTSD) 수준에 있어서도 중국체류 인구집단의 평균(38.92)과 북한에서 직접 들어온 인구집단의 평균(30.45)간에 뚜렷한 차이점이 나타나고 있다. 자살경향성 수준에 있어서도 중국을 경유한 집단의 평균(8.48)과 남한으로 직행한 집단의 평균(4.68) 사이에도 통계적으로 의미있는 차이가 나타나고 있다. 나머지 우울수준, 불면증 등 정신건강 특성에서도 마찬가지 차이점이 나타나고 있다. 일반적으로 볼 때 중국체류 인구집단에서 정신적 문제가 보다 많은 것으로 조사되었다.

이러한 특성을 감안할 때 중국체류를 거쳐서 입국하는 북한이탈주민에 대한 세밀한 검사와 지속적인 정신건강 관리가 필요하다.

2) 성별 맞춤형 지원제도

둘째 성별 맞춤형지원제도의 필요성이다. 북한이탈주민 가운데 여성인구의 압도적 비율이다. 현재 남한사회에 정착한 북한이탈주민의 성비는 2016년 12월 기준 여성인구가 71%이상이며 유입기간에 따라 80%대를 넘어선 경우도 있었다. 남한에 정착한 북한이탈주민 여성인구 가운데 20대와 30대 비율이 약 60%대에 달하고 있다. 즉 북한이탈주민의 대다수는 여성들이며 정신적 유병률이나 정신건강, 트라우마 수준에 있어서도 이와 같은 성별 특성을 중시해야 할 것이다. 일반적으로 난민집단을 대상으로 한 선행연구에서 여성들의 외상 후 스트레스 장애나 트라우마 수준이 남성들보다 심각한 것으로 보고되고 있다.

특히 북한이탈주민 여성들은 인신매매, 성폭력 등에 노출되는 빈도수가 다른 지역 난민여성보다 심각한 상태인 점을 감안한다면 북한이탈주민 가운데 여성들에 대한 맞춤형 정신건강지원체제가 특히 필요하다. 이번 조사에서 응답자 가운데 북

한탈출 과정에서 제3국에서 성폭력을 직접 체험한 사례 수가 12.43%이며 타인의 피해현장을 직접 목격한 경우까지 합하여 전체 14.59%나 된다는 사실은 매우 심각한 현실을 반영하고 있다. 이것은 남한에서 동일한 경험을 보고한 사례비율 1%와 비교할 때 15배가 높은 수치이다. 또한 응답자들 가운데 북한사회에 살고 있을 당시 성폭력을 당했던 사례는 전체 응답자 가운데 5.67%이며 다른 이가 겪는 것을 목격한 경우를 합산하면 전체 12.34%로 이 역시 상당히 높은 수치이다. 이 두 응답율의 중복사례를 감안해서 볼 때 전체적으로 15% 이상의 응답자들이 성폭력을 직접 체험했거나 목격한 것으로 추정할 수 있다. 이처럼 북한이탈주민 여성들의 경우 정상적인 사회의 경우와는 비교할 수 없을 정도로 심각한 인권유린의 피해자들이 포함되어 있다는 사실을 중시할 필요성이 있다.

이러한 북한이탈주민 여성의 특성을 감안하여 북한이탈주민 정신건강 지원체제는 일반적인 우울이나 자존감 회복을 위한 상시지원체제 이외에 심각한 인권피해, 특히 여성들에 대한 젠더폭력의 피해자들에 대한 체계적이고 전문적인 진단 및 치료, 상담시스템을 구축할 필요성이 있다. 현재 23개 지역적응센터에서는 전문상담사 제도와 각종 의료지원 시스템을 운영 중이나 이와 같은 심각한 트라우마에 대한 지원으로서는 매우 미흡한 것이 사실이다. 여성, 특히 젠더폭력 피해자를 위한 정신건강 지원을 위해서는 정신과 전문의, 성폭력 상담 전문가, 여성인권 전문가를 포함한 체계적인 통합시스템 구축이 시급하다.

통일부는 북한이탈주민지원에 있어서 2015년도부터 성인지적 관점을 적용한다고 하였으나 사업내용을 구체적으로 살펴보면 여성가족부와 고용노동부의 경력단절여성지원제도와 여성특화형 취업지원제도에 국한하고 있다. 적어도 전체 탈북여성들 가운데 최소 15% 이상에 달하는 젠더폭력 피해자 및 목격자들의 외상 후 스트레스장애가 이들의 건강한 생활과 성공적인 남한사회 정착을 가로막고 있는 숨겨진 또 하나의 정신적 요인이 아닌지 성찰해 볼 필요가 있다.

3) 생애주기별 맞춤형 지원제도

셋째 생애주기별 맞춤형지원제도이다. 여기서 말하는 생애주기별 맞춤형 지원제도에는 물론 인구학적 연령별 심리적 건강욕구의 변화에 따른 당연한 대응체제도 포함되어 있지만, 보다 중요한 것은 심각한 심리적 외상체험 집단군에 대한 특화된 전문적 생애지속 관리체제를 의미할 수 있다.

먼저 연령대별 심리욕구 대응체제 구축과 관련해서 북한인구의 지속적인 유

입추세를 지적할 수 있다. 북한이탈주민의 남한유입은 1990년대 말 이후 20년간 진행되고 있다. 앞서 지적하였듯이 이들의 인구학적 연령대별 차이와 욕구도 다양하게 변화하고 있다. 남한사회 고령화 속도를 고려할 때 북한이탈주민의 고령화도 피할 수 없는 문제이다. 북한이탈주민 고령층들은 정신적으로 더욱 심각한 고립감과 외로움, 죄책감을 가지고 살아가고 있는 것으로 알려지고 있다. 또한 이번 조사에서는 제외되어 있지만 북한이탈 청소년들의 정신건강 문제도 심각한 수준인 것으로 알려지고 있다. 청소년기에 극심한 스트레스를 체험한 북한이탈 청소년들은 남한의 교육과 사회제도에 적응하는 과정에서 많은 문제를 나타내고 있다. 이는 특히 다문화가정 청소년들의 부적응 사례와 매우 유사한 특성을 가진 것으로 알려지고 있다. 최근 북한이탈 청소년들의 학교 중도이탈율은 개선된 것으로 지표상으로 나타나고 있으나 이는 단지 학력인증을 허가한 대안학교 제도를 통해서 흡수한 것에 지나지 않는다. 문제는 대안학교를 통해서 통계적으로 정상화된 것으로 보고된 북한이탈 청소년들은 대부분 남한의 정상적인 청소년들과 새로운 친구관계를 맺고 주류사회에 진출할 수 있는 기회가 원천적으로 배제된다는 점이다. 북한이탈청소년들의 남한 대학 중도이탈율은 재학생대비 졸업율 평균 7.8% 정도로서 일반 대학생의 3배 이상으로 매우 높은 것으로 알려지고 있다.

특히 전체 북한이탈주민 여성 가운데 30% 정도가 남한사회에서 임신과 출산을 경험하고 있으며 북한에서 데려온 청소년과 남한에서 출생한 청소년사이 또 중국에 있는 자녀문제 등으로 인한 가족 스트레스 문제, 북한이탈주민 가족의 높은 가정폭력 수준, 이혼과 중혼 등으로 인한 심한 정신적 스트레스, 북한에 두고 온 가족에 대한 죄책감 등 북한이탈주민은 남한주민들과 다른 특이한 정신적 복합적 스트레스를 안고 살아가고 있다. 이러한 문제들을 반영하여 청소년기(학령기), 중장년기, 노년기 대상별 북한이탈주민의 다양한 욕구체계에 대응하는 지속가능한 중장기 지원제도 구축이 필요하다. 현재 초기 정착지원에 집중되어 있는 제도를 손질하여 보편적인 국민돌봄 제도 속에 편입시키는 발상의 전환과 정부부처 간 협치시스템이 필요한 대목이다.

또 하나의 맞춤형 지원제도로서 심각한 심리적 외상경험 집단군에 대한 임상적 식별척도 개발 및 이들에 대한 별도의 맞춤형 중장기 관리대책과 치료시스템 구축이 시급하다.[4] 당장 가해자 식별 및 처벌이 불가능한 상태에서 북한이탈주민에 대한 인권침해 피해문제는 온전히 본인이 평생을 짊어지고 가야할 정신적 심리적 위해요인이 되어 버리고 말았다. 정신건강 측면이나 인권피해자 구제 측면에서도

4) 이미 2011년 11월 24일 국가인권위원회는 북한인권침해신고센터 운영결과 데이터를 근거로 하여 통일부측에 심각한 인권피해자들을 대상으로 외상 후 스트레스경감 정신건강프로그램을 권고한 바 있다.

북한이탈주민 가운데 심각한 트라우마증상이나 특이한 증상이 나타난 북한이탈주민 대상군에 대한 맞춤형 대응매뉴얼 구축이 필요하다. 이 가운데 북한체류 중, 혹은 제3국 체류 중 발생한 외상사건에 대한 체험유무는 이들의 남한사회 심리적 적응, 남한에서의 행복한 가정생활에 있어서 매우 중요한 변수이다. 향후 이들 대상군에 대한 생애주기별 정신건강 특성과 스트레스 행동특성 등을 세밀하게 조사하여 통계적으로 유형화할 필요성이 있다. 이미 선진국에서는 이민자 가정 및 난민가정의 정주, 정착, 적응과정에 대한 학문적 연구가 많이 축적되어 지원제도 구축과 시행과정에서 활용되고 있다. 이러한 선행연구를 참조하면서 북한이탈주민의 생애주기별, 정착단계별 정신건강 위협요인, 특히 심각한 인권침해로 인한 정신적 외상유무, 주요 스트레스 관련 검사지표들을 지속적으로 축적, 관리하여 생애주기별 정착과정 전체에 대한 중장기 정신건강 지원체제맵과 지원매뉴얼을 만들 것을 제안하고자 한다.

라. 정착지원법 개정(안)

무엇보다 정신건강 문제의 심각성을 인식하고 이 문제를 해결하기 위한 법적 기반을 마련하는 것이 필요하다. 현행 <북한이탈주민의 보호 및 정착지원에 관한법률> 내용 가운데 다음과 같이 북한이탈주민을 위한 트라우마 치료센터 구축을 위한 조항 삽입이 필요하다.

개정안(신설)
제22조의 3(트라우마치료센터 설치운영) ① 통일부장관은 거주지에 전입한 북한이탈주민에 대한 정신적 트라우마 후유증의 진단 및 치료를 위한 전문적 치료센터를 설치 운영할 수 있다<br>② 통일부장관은 제1항에 따른 트라우마 치료센터 설치 및 운영을 위해서 보건복지부의 관련기관과 협의할 수 있다.<br>③ 제1항 및 제2항에 따른 트라우마 치료센터의 설치운영 등에 관한 사항은 통일부령으로 정한다.

4. 결론 : 분리/지원정책에서 지역화/통합정책으로

대다수 인권피해자들에 있어서 중대한 스트레스 요인은 자신들을 향한 부정적 시선과 낙인효과이다. 북한이탈주민들이 남한 사회 정착과정에서 매우 어려운 심리적 요인이 남한 주민들의 차가운 시선과 문화적 차이라는 점은 여러 연구자들이 강조하고 있다. 이미 많은 선행연구들은 남한국민들 사이에서 북한이탈주민의 유입 증가를 우리 사회의 부담으로 인식하는 변화가 최근 두드러지게 나타나고 있다(박상수, 2010; 박명 등, 2012) 즉 우리 국민들은 북한이탈주민의 유입이 '국민의 세금부담을 증가시킨다.'(49.7%)고 바라보는 시선이 과반수에 달하고 있다. 또한 만약 북한에서 대량난민이 발생 하게 된다면, '전원수용하기 보다 선별적 수용을 해야한다'(56.2%)는 응답이 과반수를 넘어서고 있다. 우리 국민들은 북한이탈주민을 비록 의도적으로 차별하려는 생각은 없지만, 동등하게 대우하려는 태도도 없는 것이다. 이들과 관계 맺기를 꺼려하고 있으며 결국 북한이탈주민은 우리 사회의 주변인으로 고착되는 결과를 가져올 수 있을 것이 우려된다.

이번 조사결과에서 나타나듯이 상당수 북한이탈주민들은 극심한 빈곤과 정치체제의 가혹한 탄압, 중국과 북한의 교화소 및 구금시설의 끔찍한 경험을 겪어온 바 있다. 이러한 외상적 체험들은 남한사회 정착과정에서 북한이탈주민 개인들에게는 감추고 싶은 개인사적 비밀로 인식될 수 있다. 반면에 남한 주민들에게 있어서는 북한이탈주민 대다수가 겪은 것으로 알려진 끔찍한 보편적 외상경험들은 자신들과는 상관이 없는 매우 미개한 나라에서 벌어진 흥밋거리 정도로 여겨질 수 있다. 이러한 인식체계는 더 나아가 북한, 북한사람, 북한이탈주민 전체에 대한 편견과 고정관념을 고착시키는 낙인효과를 가져올 수 있다. 실제로 대다수 북한이탈주민들은 남한사회 정착과정에서 자신들에게 향해지는 무언의 편견과 무시와 더불어 동정의 시선조차도 거북하고 싫다고 응답하고 있다.

이러한 문제점을 극복하기 위해서 현행 정책지원과정에 대한 검토와 보완도 필요하지만 무엇보다 북한이탈주민을 분리하여 대상화하는 제도구축의 근본 철학을 개선할 필요성이 있다. 즉 현재 통일부(중앙정부)가 주도하는 북한이탈주민 지원제도를 수정하여 그들의 **인권침해 트라우마 후유증을 전문적으로 치료해줄 수 있는 치료 인프라를 의료기관에 구축하는 방안이다. 또한, 북한이탈주민의료상담실을 해당 의료기관에 위탁운영하는 것이 필요하다**. 이를 위해서는 관련법의 개정이나 수정이 필요하며 보건복지부, 여성가족부, 행정안전부와의 협치체제 구축에 대한 치밀한 연구와 거시적인 정책조정도 필요하다. 북한이탈주민을 주변화하지 않기 위해서는 무엇보다 일반 국민들을 대상으로 하는 보편적인 차별철폐교육, 인권교육, 인식

개선 노력이 광범위하게 설계되어 시행되어야 한다. 이를 위해서는 통일부 차원의 노력만으로는 부족하다. 즉 이 문제는 보다 거시적인 차원에서 거버넌스 문제로 풀어갈 수도 있다. 교육부와 행정안전부, 국정홍보처 등 정부기관과 기업, 학교, 언론을 포함한 시민사회 전문가 집단의 협력과 거버넌스 체계 구축이 필요하다.

이 조사를 통해서 많은 북한이탈주민들, 특히 여성들이 심각한 인권침해로 인한 심리적 정서적 어려움을 겪고 있는 것을 확인할 수 있다. 이것은 이들이 보편적인 우리 사회의 정신건강증진 시스템 속에서 그들을 위한 특화된 치료 센터를 구축하여 보다 양질의 전문적인 치료와 보호를 받아야 할 정당성을 다시 한 번 확인시켜 준다. 또한 의료 상담실을 의료기관에서 운영할 수 있게 함으로써 의료기관의 고객 상담실처럼 질관리와 만족도 개선이 이루어지도록 함이 합당할 것이다. 또한 우리 정부와 국민들이 보다 따뜻한 시선으로 이들에 대한 관심과 동포애를 증진시켜야 할 이유를 구성한다. 무엇보다 편견과 부정적 시선 극복을 위한 남한사회의 인식개선, 사회통합노력이 더욱 필요하다.

참 고 문 헌

강성록. (2000). 탈북자들의 외상척도 개발 연구. 연세대학교 석사학위논문. 연세대학교 대학원 석사학위 논문.

김병창. (2010). 북한이탈주민의 정신건강. 정신건강정책포럼. 4(1), 19-37.

김현아, 전명남. (2003). MMPI에 나타난 북한이탈주민의 개인차 특성. 통일연구. 7(2), 129-160.

김현아. (2004). 북한이탈주민의 품행장애 행동과 성격적 특성에 관한 연구. 사회과학연구, 12(1), 150-168.

김현아, 윤여상, 한선영. (2007). 북한이탈주민의 외상 후 스트레스 증상 척도개발 및 타당화 연구. 한국심리학회, 19(3) :693-718.

곽해룡. (2005). 탈북자; 정책분석: 중국의 탈북자 정책연구. 민족연구, 14, 76-96.

국가인권위원회. (2009). 북한인권 관련 국가, 국제기구 및 INGO의 동향 분석. 국가인권위원회.

국가인권위원회. (2009). 유엔 국가별 정례인권검토(PUR)에 대한 북한의 국가인권보고서 및 우리정부, NGO, INGO 관련 자료집. 국가인권위원회.

국가인권위원회. (2012). 북한 인권침해 신고센터 1주년 보고회. 국가인권위원회.

국가인권위원회. (2016). 북한인권 국제 심포지엄 : 유엔인권메커니즘과 북한여성의 인권. 국가인권위원회.

국립중앙의료원. (2017). 북한이탈주민의 진료 현황 분석 및 질병 관리 방안 제안. 국립중앙의료원.

남궁찬, 김예란. (2016). 여성 탈북민에 대한 감정경제: 여성 탈북민 출연 프로그램의 생산 및 수용의 회로를 중심으로. 서울대학교 언론정보연구, 53(1), 105-148.

박명, 강원택, 김병로, 송영훈, 이수정, 장용석. (2012). 통일의식조사 서울: 서울대학교 통일평화연구원

박상수. (2010). 아세아연구 139 고려대학교 아세아문제연구소.

박순성, 고유환, 소라미, 이임하, 이희영, 전미영, 홍민. (2009). 탈북여성의 탈북 및 정착과정에서의 인권침해 실태조사. 국가인권위원회.

박인호. (2008). 재외탈북자의 실태와 인권현황. 북한인권대화모임 발표논문, 한국인권재단.

보건복지부, 대한정신건강재단 재난정신건강위원회. (2014), 해외 방문 현황 조사를 통한 (가칭)정신건강트라우마센터 모형 및 운영체계 개발. 보건복지부.

보건복지부, 삼성서울병원. (2016). 2016년도 정신질환실태조사. 보건복지부.

서주연. (2006). 북한이탈주민의 외상 후 스트레스장애(PTSD)와 남한사회적응에 관한 탐색적 연구. 이화여자대학교 석사학위논문.

윤여상. (2004). 재외탈북자 인권현황. (사) 기독교윤리실천운동, 북한이탈주민 인권 및 선교 현황과 방향 토론회 자료집.

윤여상, 김현아, 한선영. (2007). 북한이탈주민의 외상 후 스트레스 증상 척도개발 및 타당화 연구. 한국심리학회지: 상담 및 심리치료, 19(3), 693-718.

윤인진 · 채정민. (2010),『북한이탈주민의 정체성과 남한주민과의 상호인식』, 북한이탈주민지원재단.

이금순. (1999). 탈북자문제 해결방안. 통일연구원.

이대인. 북한이탈주민지원재단 전문상담사제도 허술해. 포커스 경제. (2016.10.14.). http://www.gungsireong.com/news/articleView.html?idxno=7182.

이숙영. (2006). 북한이탈주민의 외상경험과 남한 내 결혼만족도와의 관계. 고려대학교 석사학위논문.

이영렬. (2014). 북한이탈주민 정신건강지원대책 수립을 위한 예비연구. 국립부곡병원, 임상연구논문집.

전우택, 김연희, 조영아. (2010). 북한이탈주민 정신건강 문제 유병율과 영향요인 : 2007년 입국자 중심으로. 통일정책연구, 19(2), 141-174.

정정애. (2013). 북한이탈주민의 탈북동기와 남한사회 적응에 관한 연구. 통일정책연구. 제22권 2호.

조영아, 전우택. (2005). 탈북 여성들의 남한 사회 적응 문제: 결혼 경험자를 중심으로. 사회심리학지, 10(1): 17-35.

좋은벗들. (1999). 두만강을 넘은 사람들: 중국 동북부지역 2,479개 마을 북한 '식량난민'실태조사. 서울: 정토출판

최성근. (2012). 논단: 북한이탈주민 정착 실태와 개선방안. 통일경제, 34, 66-67.

최현옥, 손정락. (2011). 한국형 마음챙김에 기반한 스트레스 감소 (K-MBSR) 프로그램이 북한이탈주민의 외상 후 스트레스 증상, 경험회피 및 수치심에 미치는 효과. 한국심리학회지: 건강, 16(3), 469-482.

탈북청소년의 진로실태와 정책과제 토론회. (2013). 이주배경청소년지원제단 무지개청소년센터.

통일부. (2016). 북한이탈주민의 국내 입국 현황. 통일부.

하현선. (2016). 북한이탈주민 보호 및 정착지원 사업 평가. 국회예산정책처.

한나영, 이소희, 유소영, 김석주, 전진용, 원성두, 신미녀. (2015). 북한이탈주민진료센터 정신건강의학과를 내원한 북한이탈주민에서 외상 후 스트레스장애와 사회 적응 및 삶의 질 관계. 신경정신의학, 54(1), 105-111.

홍창형, 유정자, 조영아, 엄진섭, 구현지, 서승원, 전우택. (2005). 북한이탈주민의 외상 후 스트레스 장애에 대한 3 년 추적연구. 연세대학교 석사학위논문.

Acarturk, C., Konuk, E., Cetinkaya, M., Senay, I., Sijbrandij, M., Cuijpers, P., & Aker, T. (2015). EMDR for Syrian refugees with posttraumatic stress disorder symptoms: Results of a pilot randomized controlled trial. European Journal of Psychotraumatology, 6(1), 27414.

Bogic, M., Njoku, A., & Priebe, S. (2015). Long-term mental health of war-refugees: a systematic literature review. BMC international health and human rights, 15(1), 29.

Bong-Hee Jeon, Moon-Doo Kim, Seong-Chul Hong, Na-Ri Kang, Chang-In Lee, Young-Sook Kwak, Joon-Hyuk Park, Jaehwan Chung, Hanul Chong, Eun-Kyung Jwa, Min-Ho Bae, Sanghee Kim, Bora Yoo, Jun-Hwa Lee, Mi-Yeul Hyun, Mi-Jeong Yang, & Duk-Soo Kim. (2009). Prevalence and Correlates of Depressive Symptoms among North Korean Defectors Living in South Korea for More than One Year. Psychiatry Invest, 6, 122-130.

Boris D. (2015). Challenges in treatment of posttraumatic stress disorder in refugees: towards integration of evidence-based treatments with contextual and culture-sensitive perspectives. European Journal of Psychotraumatology, 6, 247-250.

Buhman, C., Mortensen, E. L., Lundstrøm, S., Ryberg, J., Nordentoft, M., & Ekstrøm, M. (2013). Symptoms, quality of life and level of functioning of traumatized refugees at psychiatric trauma clinic in Copenhagen. Torture: quarterly journal on rehabilitation of torture victims and prevention of torture, 24(1), 25-39.

Buhmann, C., Andersen, I., Mortensen, E. L., Ryberg, J., Nordentoft, M., & Ekstrøm, M. (2015a). Cognitive behavioral psychotherapeutic treatment at a psychiatric trauma clinic for Refugees: description and evaluation. Torture, 25(1), 17-32.

Buhmann, C., Mortensen, E. L., Nordentoft, M., Ryberg, J., & Ekstrøm, M. (2015b). Follow-up study of the treatment outcomes at a psychiatric trauma clinic for refugees. Torture, 25(1), 1-16.

Buhmann, C. B., Nordentoft, M., Ekstroem, M., Carlsson, J., & Mortensen, E. L. (2016). The effect of flexible cognitive–behavioural therapy and medical treatment, including antidepressants on post-traumatic stress disorder and depression in traumatised refugees: pragmatic randomised controlled clinical trial. The British Journal of Psychiatry, 208(3), 252-259.

Cohen, J. (1969). Statistical power analysis for the behavioural sciences. New York: Academic Press.

Cho, Y. A., Kim, Y. H., & You, S. E. (2009). Predictors of mental health among North Korean defectors residing in South Korea over 7 years. Korean Journal of Counseling and Psychotherapy, 21(1), 329-348.

Faul, F., Erdfelder, E., Lang, A. G., & Buchner, A. (2007). G* Power 3: A flexible statistical power analysis program for the social, behavioral, and biomedical sciences. Behavior research methods, 39(2), 175-191.

Jeon, W., Hong, C., Lee, C., Kim, D. K., Han, M., & Min, S. (2005). Correlation between traumatic events and posttraumatic stress disorder among North Korean defectors in South Korea. Journal of traumatic stress, 18(2), 147-154.

Jeon, W. T., Yu, S. E., Cho, Y. A., & Eom, J. S. (2008). Traumatic experiences and mental health of North Korean refugees in South Korea. Psychiatry investigation, 5(4), 213-220.

Knipscheer, J. W., Sleijpen, M., Mooren, T., Ter Heide, F. J. J., & van der Aa, N. (2015). Trauma exposure and refugee status as predictors of mental health outcomes in treatment-seeking refugees. BJPsych Bull, 39(4), 178-182.

Kang, C. (2006). The relationship between stress coping style and mental health among female North Korean refugees in China. Korean Journal of Women's Psychology, 10(1), 61-80.

Kartal, D., & Kiropoulos, L. (2016). Effects of acculturative stress on PTSD, depressive, and anxiety symptoms among refugees resettled in Australia and Austria. European Journal of Psychotraumatology, 7(1), 28711.

Lee, Y., Lee, M. K., Chun, K. H., Lee, Y. K., & Yoon, S. J. (2001). Trauma experience of North Korean refugees in China. American journal of preventive medicine, 20(3), 225-229.

Lee, S. Y. (2006). Correlations Between Traumatic Experience and Marital Satisfaction Scale of an Escapee from North Korea-Centered on Dwelling in North Korea, Escaping from North Korea, Adapting to South Korea. Seoul: Korea University.

Lindert, J., von Ehrenstein, O. S., Priebe, S., Mielck, A., & Brähler, E. (2009). Depression and anxiety in labor migrants and refugees–a systematic review and meta-analysis. Social science & medicine, 69(2), 246-257.

Opaas, M., &Varvin, S. (2015). Relationships of childhood adverse experiences with mental health and quality of life at treatment start for adult refugees traumatized by pre-flight experiences of war and human rights violations. The Journal of nervous and mental disease, 203(9), 684.

PoKempner, D., Baik TU, & Jendrzejczyk (2002). The invisible exodus: North Koreans in the Peoples Republic of China. Human Rights Report, 14(8).

Pumariega, A. J., Rothe, E., & Pumariega, J. B. (2005). Mental health of immigrants and refugees. Community mental health journal, 41(5), 581-597.

Schick, M., Zumwald, A., Knöpfli, B., Nickerson, A., Bryant, R. A., Schnyder, U., ... & Morina, N. (2016). Challenging future, challenging past: The relationship of social integration and psychological impairment in traumatized refugees. European journal of psychotraumatology, 7, 28057.

Shin, C. H., & Go, M. H. (2014). Beyond the UN COI Report on Human Rights in North Korea. The ASAN Institute for Policy Studies, 30.

Shin, H. Y., Lee, H., & Park, S. M. (2016). Mental Health and Its Associated Factors Among North Korean Defectors Living in South Korea: A Case-Control Study. Asia Pacific Journal of Public Health, 28(7), 592-600.

Spiller, T. R., Schick, M., Schnyder, U., Bryant, R. A., Nickerson, A., & Morina, N. (2016). Somatisation and anger are associated with symptom severity of posttraumatic stress disorder in severely traumatised refugees and asylum seekers. Swiss Med Wkly, 146, w14311.

Shevlin, M., Armour, C., Murphy, J., Houston, J. E., & Adamson, G. (2011). Evidence for a psychotic posttraumatic stress disorder subtype based on the National Comorbidity Survey. Social psychiatry and psychiatric epidemiology, 46(11), 1069-1078.

Teodorescu, D. S., Heir, T., Siqveland, J., Hauff, E., Wentzel-Larsen, T., & Lien, L. (2015). Chronic pain in multi-traumatized outpatients with a refugee background resettled in Norway: a cross-sectional study. BMC psychology, 3(1), 7.

UNHCR. (2014). “UNHCR global trends, 2013: War’s human cost”, United Nations, Geneva.

Woo-Teak Jeon, Shi-Eun Yu, Young-A Cho, & Jin-Sup Eom. (2000). Traumatic Experiences and Mental Health of North Korean Refugees in South Korea. Psychiatry Investig 5:213-220.

Yu, S., Brackbill, R. M., Locke, S., Stellman, S. D., & Gargano, L. M. (2016). Impact of 9/11-related chronic conditions and PTSD comorbidity on early retirement and job loss among World Trade Center disaster rescue and recovery workers. American journal of industrial medicine, 59(9), 731-741.

홈페이지

국가인권위원회(http://www.humanrights.go.kr)

남북하나재단(https://www.koreahana.or.kr/index.jsp)

통일부(http://www.unikorea.go.kr/unikorea/)

〈부록 1〉 인권침해 조사표

※ 평가자가 작성하는 항목입니다(북한N, 탈북기간E, 남한S).

설문항목	①	②	③	④	⑤
	내가 직접 경험하였다.	가족들이 당하는 것을 경험하였다.	주변사람들이 당하는 것을 목격하였다	소문을 들었다	전혀 겪거나 들은 바 없다
① 심한 굶주림 또는 질병					
② 정치범수용소의 잔혹성					
③ 공개처형					
④ 국가기관/군대에서 매질					
⑤ 잘못 없이 국가기관에 끌려감					
⑥ 이웃과 당원의 감시와 고발					
⑦ 토대가 나쁘다는 이유로 인한 차별					
⑧ 공개적인 자아비판					
⑨ 통신검열 또는 록화기 단속					
⑩ 굶주림으로 인한 죽음					
⑪ 탈북으로 인한 처벌(강제송환)					
⑫ 여성에 대한 성폭력					
⑬ 여성 또는 아동 인신매매					

<부록 2> 스트레스 사건 목록 (Life Events Checklist, LEC-5)

아래 열거된 것들은 힘들거나 스트레스가 되는 일들로 누구에게나 일어날 수 있습니다.
각 사건마다 한 개 또는 그 이상 해당하는 것에 ○표를 하시기 바랍니다.

(a) 당신이 그 일을 직접 겪은 경우, (b) 다른 사람에게 일어난 일을 목격한 경우

아래의 사건들에 대하여, **(성인 시절뿐만 아니라 어릴 때도 포함)** 당신의 인생 전체를 되돌아보며 응답해주시기 바랍니다.		북한		탈북기간		남한	
		직접 겪음	목격함	직접 겪음	목격함	직접 겪음	목격함
1	자연재난 (예: 홍수, 태풍, 폭풍, 지진)	a	b	a	b	a	b
2	화재 또는 폭발	a	b	a	b	a	b
3	교통사고 (예: 자동차 사고, 선박 사고, 기차 사고, 비행기 추락)	a	b	a	b	a	b
4	직장, 집, 또는 여가 활동 중 심각한 사고	a	b	a	b	a	b
5	독성 물질에 노출 (예: 유독성 화학물질, 방사능)	a	b	a	b	a	b
6	신체폭력 (예: 공격당하거나, 가격(손, 주먹, 몽둥이로 공격)당하거나, 따귀를 맞거나, 발로 채이거나, 두들겨 맞음)	a	b	a	b	a	b
7	무기로 공격당함 (예: 총이나 칼에 맞거나, 칼, 총, 폭탄으로 위협 당함)	a	b	a	b	a	b
8	성폭력 (성폭행, 성폭행 시도, 완력이나 위협 하에 성적 행위에 해당하는 것을 하게 됨)	a	b	a	b	a	b
9	기타 원하지 않거나 불편한 성적 경험	a	b	a	b	a	b
10	전투나 전쟁터에 노출 (군 근무 또는 민간인으로서)	a	b	a	b	a	b
11	감금 (예: 납치, 유괴, 인질, 전쟁 포로, 중국에서 남편이나 시댁식구들로부터)	a	b	a	b	a	b
12	목숨이 좌우될 정도의 질병이나 부상	a	b	a	b	a	b
13	심각한 인간적 고난 (예: 강제적인 노동, 지속적인 굶주림 또는 식량 부족, 지속적인 노숙 상태, 고문)	a	b	a	b	a	b
14	급작스러운 변사 (예: 살인, 자살)	a	b	a	b	a	b
15	급작스러운 사고사	a	b	a	b	a	b
16	나 자신 때문에 발생했던 다른 사람의 심각한 부상, 상해 또는 사망	a	b	a	b	a	b
17	그 밖의 매우 심각한 스트레스 사건이나 경험	a	b	a	b	a	b

생활사건점검표 (Life Events Checklist for DSM-5:LEC-5)

〈부록 3〉 탈북민 트라우마 평가 설문지

◎ 우울

<u>지난 2주 동안</u> 아래 나열되는 증상들에 **<u>얼마나 자주</u>** 시달렸습니까?

문 항	없음	2일 이상	1주일 이상	거의 매일
1. 어떤 일에도 관심이나 재미가 없음	ⓞ	①	②	③
2. 처지는 느낌, 우울감 혹은 절망감	ⓞ	①	②	③
3. 잠들기 어려움, 깨지 않고 쭉 자기 어려움, 혹은 너무 많이 잠	ⓞ	①	②	③
4. 피곤함 혹은 기운 없음	ⓞ	①	②	③
5. 식욕(입맛) 저하 혹은 과식	ⓞ	①	②	③
6. 나 자신을 못마땅하게 여김<br>- 나 자신이 실패자 같음, 혹은 나 자신이나 가족을 실망시킨 것 같음	ⓞ	①	②	③
7. 신문 읽기나 텔레비전 시청과 같은 일상적인 일에도 집중하기 어려움	ⓞ	①	②	③
8. 움직이는 것이나 말하는 것이 너무 느려서 남들이 알아챌 정도임.<br>혹은 너무 불안해서 평소보다 안절부절 못함.	ⓞ	①	②	③
9. 차라리 죽는 것이 낫겠다는 생각<br>혹은 나 자신을 스스로(내 절로) 해칠지도 모른다는 생각	ⓞ	①	②	③

한 개 증상이라도 1점 이상으로 체크(표시)를 하신 분만 보세요.

위의 해당 증상 때문에 **<u>직장이나 집안 일을 할 때 혹은 주변 사람들과의 관계에서</u>** 어려움을 겪으셨다면 해당 번호에 ○표를 하시기 바랍니다.

① 전혀 없었다. ② 조금 어려웠다. ③ 많이 어려웠다. ④ 아주 어려웠다.

건강질문지-9 (Patient Health Questionnaire, PHQ-9)

◎ K-MINI

	**지난 1개월간 당신은:**		
1	차라리 죽는 것이 더 낫다고 생각하거나 죽기를 바랐던 적이 있습니까?	☐ 아니오	☐ 예
2	자해(본인에게 상처를 내는 등 해를 끼치는)를 하고 싶었던 적이 있습니까?	☐ 아니오	☐ 예
3	자살에 대해서 생각했던 적이 있습니까?	☐ 아니오	☐ 예
4	자살을 계획했었습니까?	☐ 아니오	☐ 예
5	자살을 시도했습니까?	☐ 아니오	☐ 예
	**일생동안:**		
6	한 차례라도 자살 시도를 한 적이 있습니까?	☐ 아니오	☐ 예

자살경향성 척도 K-MINI

◎ **외상 후 스트레스 장애**

다음은 매우 심한 스트레스(정신적, 신체적 피로감이 있는 상태)를 경험한 사람들에게 때때로 발생할 수 있는 문제들입니다. 각 문항을 주의 깊게 읽고, **지난 1달 동안** 다음과 같은 문제로 얼마나 고통스러웠는지를 해당하는 것에 ○표를 하시기 바랍니다.

지난 1달 동안,<br>아래의 문제로 인해 얼마나 고통스러웠습니까?		전혀 아님	약간	보통	많이	매우 많이
1	그 스트레스 경험에 대한 괴롭고 원하지 않는 기억이 반복적으로 떠오른다.	0	1	2	3	4
2	그 스트레스 경험과 관련된 악몽을 반복해서 꾼다.	0	1	2	3	4
3	갑자기 그 스트레스 경험을 실제로 다시 체험하는 것처럼 느끼거나 행동하게 된다.	0	1	2	3	4
4	무엇인가 그 스트레스 경험을 떠오르게 하면 마음이 매우 힘들어진다.	0	1	2	3	4
5	무엇인가 그 스트레스 경험을 떠오르게 하면 강렬한 신체 증상이 나타난다.<br>(예를 들면, 두근거림, 호흡곤란, 식은땀)	0	1	2	3	4
6	그 스트레스 경험과 관련된 기억, 생각 혹은 감정에서 벗어나려고 한다.	0	1	2	3	4
7	그 스트레스 경험을 떠오르게 하는 어떤 외부의 자극을 피한다.<br>(예를 들면, 사람, 장소, 대화, 활동, 물건, 상황)	0	1	2	3	4
8	그 스트레스 경험과 관련된 어떤 중요한 부분을 기억해내기 어렵다.	0	1	2	3	4
9	나 자신, 다른 사람, 혹은 세상에 대해 절대로 안 된다고 생각하는 신념(신심)을 가지고 있다. (예를 들면, 나는 나쁘다, 나에게는 뭔가 심각하게 잘못된 것이 있다, 아무도 믿을 수 없다, 세상은 어디든 위험하다, 내게는 늘 안 좋은 일만 생긴다.)	0	1	2	3	4
10	그 스트레스 경험이나 그 이후에 일어난 일들에 대해 나 자신이나 누군가를 탓한다.	0	1	2	3	4
11	두려움, 공포, 분노, 죄책감, 수치심과 같은 강력한 부정적인 감정을 가지고 있다.	0	1	2	3	4
12	예전에는 즐거운 마음으로 하던 일이 이제는 다 재미없어졌다.	0	1	2	3	4
13	다른 사람들과의 관계가 멀어지거나 끊어졌다고 느낀다.	0	1	2	3	4
14	긍정적인 감정을 느끼는데 어려움이 있다. (예를 들면, 행복감을 느낄 수 없거나 친밀한 사람들에게 애정을 느낄 수 없는 것)	0	1	2	3	4
15	신경질, 분노폭발 혹은 공격적인 행동을 한다.	0	1	2	3	4
16	매우 위험한 행동을 하거나 자신에게 해가 될 수 있는 행동을 한다.	0	1	2	3	4
17	매우 예민하게 늘 주변을 살피고 경계하게 된다.	0	1	2	3	4
18	작은 일에도 쉽게 깜짝깜짝 놀라게 된다.	0	1	2	3	4
19	집중하기가 어렵다.	0	1	2	3	4
20	잠들기가 어렵거나 깨지 않고 쭉 자기가 어렵다.	0	1	2	3	4
21	왠지 나 자신의 미래가 갑자기 끝날 것 같은 느낌이 든다.	0	1	2	3	4

외상 후 스트레스 장애 체크리스트 (PCL-5 with a item of PCL-IV)

◎ 불안증상

**<u>지난 2주 동안</u>** 다음의 어려움으로 인해<br><u>얼마나 자주</u> 신경이 쓰였습니까?	없음	2일 이상	1주일<br>이상	거의<br>매일
1. 초조, 불안, 조마조마함.	0	1	2	3
2. 걱정하는 것을 멈추거나 조절할 수 없음.	0	1	2	3
3. 이런저런 다양한 걱정을 너무 많이 함.	0	1	2	3
4. 진정하기 어려움.	0	1	2	3
5. 가만히 있지 못할 정도로 안절부절 못함.	0	1	2	3
6. 쉽게 짜증이 남.	0	1	2	3
7. 끔찍한 일이 생길 것 같은 두려움이 생김.	0	1	2	3

불안장애선별검사 (Generalized Anxiety Disorder Scale, GAD-7)

◎ 불면증

**<u>현재 (지난 2주 동안)</u>** 불면 문제들의 심각도를 평가하십시오.					
1. 잠들기 어려움	⓪ 없음	① 약간	② 중간	③ 심함	④ 매우
2. 깊은 잠을 자기 어려움	⓪ 없음	① 약간	② 중간	③ 심함	④ 매우
3. 너무 일찍 깨는 문제	⓪ 없음	① 약간	② 중간	③ 심함	④ 매우
4. 현재 당신의 수면 패턴(틀, 규칙)에 대해 얼마나 만족하고 있습니까?					
⓪ 매우 만족	① 만족	② 중간	③ 불만족	④ 매우 불만족	
5. 불면증이 일상생활 (예: 주간피로(낮에 피로), 업무 혹은 일상적 가사 능력, 집중력, 기억력, 기분 등) 을 어느 정도 방해한다고 생각합니까?					
⓪ 전혀 방해되지 않음	① 조금 방해됨	② 다소 방해됨			
③ 많이 방해됨	④ 매우 많이 방해됨				
6. 불면증으로 인해 제대로 생활을 할 수 없는 당신을 다른 사람들은 어떻게 볼 거라고 생각합니까?					
⓪ 전혀 알아보지 못할 정도	① 조금 알아보는 정도	② 다소 알아보는 정도			
③ 많이 알아보는 정도	④ 매우 많이 알아보는 정도				
7. 현재 불면증에 관하여 얼마나 걱정하고 있습니까?					
⓪ 전혀	① 조금	② 다소	③ 많이	④ 매우 많이	

불면증 심각도 척도 (Insomnia Severity Index, ISI)

◎ **삶의 질**

당신의 건강 상태에 대하여 당신의 의견을 묻는 것입니다. 당신의 응답은 본인의 건강 상태에 대해서 어떻게 느끼고 일상적인 활동을 어느 정도 잘 할 수 있는가를 알아보는데 도움이 될 것입니다.
[당신의 건강 상태를 가장 잘 표현한 것에 √로 표시하여 주십시오.]

1. 전반적으로 지난 한 달 동안 당신의 건강 상태는 어떠하였습니까?

① 최고로 좋았다	② 상당히 좋았다	③ 좋았다	④ 그저 그랬다	⑤ 나빴다	⑥ 아주 나빴다

2. 지난 한 달 동안 당신은 신체적인 건강 문제 때문에 일상적인 신체활동 (예: 걷는 것 혹은 계단을 걸어 올라가는 것)에 어느 정도의 제한을 받았습니까?

① 전혀 받지 않았다	② 거의 받지 않았다	③ 어느 정도 받았다	④ 상당히 많이 받았다	⑤ 신체 활동을 할 수 없었다

3. 지난 한 달 동안 당신은 신체적인 건강 때문에 집에서나 밖에서 일상적인 일 (예: 집안일과 직장에서의 업무)을 하는데 얼마나 어려움이 있었습니까?

① 전혀 없었다	② 조금 있었다	③ 어느 정도 있었다	④ 상당히 많이 있었다	⑤ 일상적인 일을 할 수 없었다

4. 지난 한 달 동안 당신의 몸에는 통증이 얼마나 있었습니까?

① 전혀 없었다	② 아주 가벼운 통증이 있었다	③ 가벼운 통증이 있었다	④ 어느 정도의 통증이 있었다	⑤ 심한 통증이 있었다	⑥ 매우 심한 통증이 있었다

5. 지난 한 달 동안 당신은 어느 정도의 활력 (기운 혹은 스태미나)이 있었습니까?

① 굉장히 많이 있었다	② 꽤 많이 있었다	③ 어느 정도 있었다	④ 아주 조금 있었다	⑤ 전혀 없었다

6. 지난 한 달 동안 당신은 신체적인 건강 혹은 정신적인 문제 때문에 친척이나 친구들을 만나거나 모임에 참석하는데 어느 정도의 제한을 받았습니까?

① 전혀 받지 않았다	② 거의 받지 않았다	③ 어느 정도 받았다	④ 상당히 많이 받았다	⑤ 교제나 모임에 참석할 수 없었다

7. 지난 한 달 동안 당신은 정신적인 문제 (예: 불안감, 우울 증상, 신경과민) 때문에 얼마나 괴로움을 당했습니까?

① 전혀 괴로움이 없었다	② 아주 조금 괴로웠다	③ 어느 정도 괴로웠다	④ 꽤 많이 괴로웠다	⑤ 아주 심하게 괴로웠다

8. 지난 한 달 동안 개인적인 또는 정신적인 문제 때문에 당신이 일상적인 활동 (예: 직장 업무, 집안일, 학업 혹은 다른 평상시 하는 일)을 하는데 얼마나 지장을 받았습니까?

① 전혀 받지 않았다	② 거의 받지 않았다	③ 어느 정도 받았다	④ 상당히 많이 받았다	⑤일상적인 일을 할 수 없었다

건강관련 삶의 질 평가(Short Form-8 Health survey; SF-8)

<평가자가 작성하는 항목입니다>

○ 인구사회학적 특성

**북한에서**	
주요 거주지: 도	
직업	□유 □무 □장사
결혼상태	□기혼(결혼상태) □미혼(결혼하지 않음) □동거(결혼신고 없이 같이 생활) □이혼<br>□별거(부부가 떨어져 지냄) □사별(남편 혹은 아내 사망) □기타

**탈북기간 동안**					
최초탈북 연도: 년			하나원 퇴소 연도: 년		
제3거주국가	□직행 □중국 □러시아 □기타				
중국공안 체포	□예(회)<br>□아니오	북송경험	□예(회)<br>□아니오	교화소, 구치소	□예(회)<br>□아니오

**남한에서**				
취업경험	□예 □아니오	직업:	현재 지속여부 □예 □아니오	
	그만 둔 이유:			
남한결혼상태	□기혼 □동거 □미혼 □이혼 □별거 □사별 □기타			
	배우자 출신	□탈북민 □조선족 □한족 □남한 □기타		
북한 잔류가족	□예 (관계:) (명)		□아니오	
자녀양육	□예 (□13세 이하 □동거 □기숙사)		□아니오	
	출산국가	□북한	부 국적 (북한)	
		□중국	부 국적 (북한 / 한족 / 조선족)	
		□남한	부 국적 (북한 / 한족 / 조선족 / 남한)	
Medical/Psychiatric Hx				
진단받은 질병	진단명:	진단당시 치료 □예 □아니오	치료지속여부 □예 □아니오	
	치료지속하지 못한 이유:			
문화적응 스트레스	전혀 없다	조금 어렵다	매우 어렵다	구체적으로
① 남한 언어 이해 어려움				
② 남한 사람들과 어울리는데 어려움				
③ 남한 사람들에게 무시당하는 느낌, 경험				
④ 이웃과 어울리기, 도움 주고받기 어려움				
⑤ 그 외 남한에서 살면서 힘든 점 :				

◎ 음주

1. (최근 1년간) 얼마나 자주 술을 마십니까?
ⓞ 전혀 안마심 ① 월 1회 미만 ② 월 2~4회 ③ 주 2~3회 ④ 주 4회 이상
2. 술을 마시면 한 번에 몇 잔 정도 마십니까?(소주잔, 맥주잔 구분 없이)
ⓞ 한 두잔 ① 3~4잔 ② 5~6잔 ③ 7~9잔 ④ 10잔 이상
3. 한 번에 소주 1병 이상 혹은 맥주 4병 이상 마시는 경우는 얼마나 자주 있습니까?
ⓞ 전혀 없다 ① 월 1회 미만 ② 월 1회 ③ 주 1회 ④ 거의 매일

◎ 흡연 : 현재 흡연을 하고 계신다면 내용을 작성하여 주십시오.

몇 년째 담배를 피우시고 계십니까?<br>(**중간에 금연하였던 기간이 있는 경우 그 기간 제외**)	총 ______년
평균 하루 흡연량은 몇 개비였습니까?	__________개비

<수고하셨습니다>

색 인

- **건강관련 삶의 질 평가**(Short Form-8 Health survey, SF-8) : 질병, 건강 관련하여 삶의 질을 평가하는 설문지로서 총 8문항이고 각 문항 당 1~5점으로 답하도록 되어 있다. 총합으로 계산되고 점수가 높을수록 삶의 질이 저하되어 있음을 의미한다.

- **건강질문지-9**(Patient Health Questionnaire-9, PHQ-9) : PHQ는 1999년 Spitzer가 일차 진료기관에서 볼 수 있는 정신 질환을 진단하기 위해 자가보고 형식으로 개발한 도구이고, PHQ-9은 이 중 우울증을 평가하는 도구이다. 총 문항은 9문항으로 각 문항은 0~3점으로 계산하여 중등도 우울의 절단점은 10점이다.

- **난민** : 인종, 종교 또는 정치적, 사상적 차이로 인한 박해를 피해 외국이나 다른 지방으로 탈출하는 사람

- **망명자** : 자기 나라에서 정치적인 이유에 의해서 박해를 받게 될 근거가 있으므로 외국으로 도피하는 자, 또는 현재 외국에 있으면서 귀국하면 박해를 받을 근거가 있어서 귀국하지 않으려는 자를 말하며, 정치적 난민이라고도 한다.

- **불면증 심각도 지수**(Insomnia Severity Index, ISI) : 불면증의 정도를 측정하기 위해 개발된 자기보고식 척도이고 5문항으로 구성되어 있다. 점수는 0~28점까지 가능하며 국내 중등도 불면증의 절단점은 16점이다.

- **불안척도-7**(7 items anxiety scale, GAD-7) : 전반적 불안장애를 선별하기 위해 자기보고식으로 개발된 설문지이다. 총 7문항으로 구성되어있고 각 문항은 0~3점으로 계산하며, 중등도 불안의 절단점은 10점이다.

▪ **신체화(Somatization)** : 욕동, 방어 그리고 그것들 사이의 갈등과 같은 다양한 자극에 신체적으로 반응하는 경향성을 말함. 즉 정신 에너지가 신체적 증상으로 표현되는 것을 일컫는다.

▪ **외상 후 스트레스 장애(Posttraumatic Stress Disorder, 이하 PTSD)** : 생명을 위협할 정도의 극심한 스트레스 혹은 충격적이거나 두려운 사건을 당하거나 목격(트라우마)을 경험하고 나서 발생하는 심리적 반응을 말한다. 이러한 외상들은 대부분 갑작스럽게 일어나며 경험하는 사람에게 심한 고통을 주고 일반적인 스트레스 대응 능력을 압도한다.

▪ **이주민** : 다른 곳으로 옮겨 가서 사는 사람. 또는 다른 지역에서 옮겨 와서 사는 사람.

▪ **생활사건 조사표(Life Events Checklist, LEC-5)** : 개인이 일생동안 겪은 트라우마 생활 사건을 평가하기 위해 개발된 자기보고형 척도로서 총 17문항으로 구성되어 있다. 점수화된 척도가 아닌 트라우마(정신적 외상) 경험 유무만을 평정한다.

▪ **스노우볼 샘플링(snowball sampling)** : 첫 단계에서는 무작위로 표본이 선택되고 이후에는 다양한 요인에 의해 표본이 선택된다. 소개의 소개를 받아 계속 연구대상이 표본으로 선택되는 비확률적 샘플링으로서, 모집단에 속하는 연구대상을 찾기 어려울 때, 유용하다.

▪ **자살경향성 척도(K-MINI)** : 정신장애진단 및 통계편람(Diagnostic and Statistical Manual of Mental disorders, DSM) 진단 기준에 따라 고안된 간략한 구조화된 면접도구로서 그 중 자살경향성을 평가하기 위한 영역이다. 총 5문항이고 문항마다 가중치가 다르게 채점되어 자살 위험성이 거의 없음, 낮음, 높음 3단계로 나뉜다.

▪ **트라우마(trauma)** : 생명을 위협할 정도의 극심한 스트레스 혹은 충격적이거나 두려운 사건을 의미한다. 정신적 외상이라고도 부른다.

▪ PTSD 체크리스트(PTSD check List-5, PCL-5) : 정신장애진단 및 통계편람(Diagnostic and Statistical Manual of Mental disorders, DSM)의 PTSD 증상에 따라 PTSD를 진단하기 위해 Weathers 등이 개발한 자가보고 척도이다. 총 20개의 문항으로, 각 문항은 0-4점으로 총점의 범위는 0에서 80점이며 38점 이상일 경우 PTSD를 의심할 수 있다.

▪ The Alcohol Use Disorders Identification Test(AUDIT-C) : 위험 음주나 알코올 사용 장애를 선별하기 위해 고안된 자기보고식 척도로서 총 3문항으로 구성되어 있고 각 문항은 0~4점으로 답하도록 되어 있다. 음주 문제의 절단점 기준은 남자는 4점, 여자는 3점이다.

북한이탈주민 인권피해 트라우마 실태조사

인쇄일 : 2017년 10월 27일
발행일 : 2017년 10월 27일
발행처 : 국가인권위원회
주　소 : 04551 서울시 중구 삼일대로 340
(저동 1가) 나라키움 저동빌딩
전　화 : (02) 2125-9842 (북한인권팀)
팩　스 : (02) 2125-0918 (운영지원과)
이메일 : nanuhh@korea.kr
제　작 : 현대아트컴

ISBN 978-89-6114-576-3 93340 (비매품)
